# Yersiniabactin-Producing Adherent/Invasive Escherichia coli Promotes Inflammation-Associated Fibrosis in Gnotobiotic *Il10^−/−^* Mice

**DOI:** 10.1128/IAI.00587-19

**Published:** 2019-10-18

**Authors:** Melissa Ellermann, Raad Z. Gharaibeh, Laura Fulbright, Belgin Dogan, Lyndsey N. Moore, Christopher A. Broberg, Lacey R. Lopez, Aaron M. Rothemich, Jeremy W. Herzog, Allison Rogala, Ilyssa O. Gordon, Florian Rieder, Cory R. Brouwer, Kenneth W. Simpson, Christian Jobin, R. Balfour Sartor, Janelle C. Arthur

**Affiliations:** a Department of Microbiology and Immunology, University of North Carolina at Chapel Hill, Chapel Hill, North Carolina, USA; b Center for Gastrointestinal Biology and Disease, University of North Carolina at Chapel Hill, Chapel Hill, North Carolina, USA; c Lineberger Comprehensive Cancer Center, University of North Carolina at Chapel Hill, Chapel Hill, North Carolina, USA; d Department of Medicine, University of Florida, Gainesville, Florida, USA; e Department of Infectious Diseases and Pathology, University of Florida, Gainesville, Florida, USA; f Department of Clinical Sciences, College of Veterinary Medicine, Cornell University, Ithaca, New York, USA; g Department of Pathology, Robert J. Tomsich Pathology and Laboratory Medicine Institute, Cleveland Clinic Foundation, Cleveland, Ohio, USA; h Department of Gastroenterology, Hepatology and Nutrition, Digestive Diseases and Surgery Institute, Cleveland Clinic Foundation, Cleveland, Ohio, USA; i Department of Inflammation and Immunity, Lerner Research Institute, Cleveland Clinic Foundation, Cleveland, Ohio, USA; j Department of Bioinformatics and Genomics, University of North Carolina at Charlotte, Charlotte, North Carolina, USA; Georgia Institute of Technology School of Biological Sciences

**Keywords:** fibrosis, AIEC, Crohn’s disease, colitis, microbiome, yersiniabactin, intestinal inflammation, microbiota

## Abstract

Fibrosis is a significant complication of intestinal disorders associated with microbial dysbiosis and pathobiont expansion, notably Crohn’s disease (CD). Mechanisms that favor fibrosis are not well understood, and therapeutic strategies are limited. Here we demonstrate that colitis-susceptible *Il10*-deficient mice develop inflammation-associated fibrosis when monoassociated with adherent/invasive Escherichia coli (AIEC) that harbors the yersiniabactin (Ybt) pathogenicity island. Inactivation of Ybt siderophore production in AIEC nearly abrogated fibrosis development in inflamed mice. In contrast, inactivation of Ybt import through its cognate receptor FyuA enhanced fibrosis severity. This corresponded with increased colonic expression of profibrogenic genes prior to the development of histological disease, therefore suggesting causality. *fyuA*-deficient AIEC also exhibited greater localization within subepithelial tissues and fibrotic lesions that was dependent on Ybt biosynthesis and corresponded with increased fibroblast activation *in vitro*. Together, these findings suggest that Ybt establishes a profibrotic environment in the host in the absence of binding to its cognate receptor and indicate a direct link between intestinal AIEC and the induction of inflammation-associated fibrosis.

## Abstract (precis)

Fibrosis is a significant complication of intestinal disorders associated with microbial dysbiosis and pathobiont expansion, notably Crohn’s disease (CD). Mechanisms that favor fibrosis are not well understood, and therapeutic strategies are limited. Here we demonstrate that colitis-susceptible *Il10*-deficient mice develop inflammation-associated fibrosis when monoassociated with adherent/invasive Escherichia coli (AIEC) that harbors the yersiniabactin (Ybt) pathogenicity island.

## INTRODUCTION

Inflammatory bowel diseases (IBD), including Crohn’s disease (CD), are characterized by chronic intestinal inflammation that develops as a result of prolonged and inappropriate mucosal immune responses to luminal antigens in genetically susceptible individuals (1). The chronic and relapsing nature of IBD, in conjunction with the lack of curative therapies for many patients, enhances risk for inflammation-associated comorbidities, including intestinal fibrosis (2). Approximately 30% of CD patients develop fibrotic disease that can result in intestinal strictures and bowel obstructions (2–4). Current treatments for intestinal fibrosis are inadequate and rely on anti-inflammatory therapies (which are often ineffective) and surgical interventions (3). Fibrosis is recurrent in large proportions of the population with CD (4), thus necessitating the development of specific antifibrotic therapeutics.

Fibrosis is characterized by excess accumulation of extracellular matrix (ECM) components that results in the pathological remodeling of tissues and consequent organ dysfunction. Mesenchymal cells such as fibroblasts, myofibroblasts, and smooth muscle cells become highly activated in response to transmural injury or inflammation and hypersecrete ECM components and profibrogenic factors that further propagate fibrotic processes. The tissue microenvironment also plays an important role in modulating the activity of mesenchymal cells, where host-derived signals such as cytokines and growth factors serve as additional fibrogenic or antifibrotic mediators (3, 4). Activation of mesenchymal cells is also subject to regulation by microbial factors (5, 6). Fibrosis can occur in bacterium-induced models of acute colitis, including mice chronically colonized with the enteric pathogen Salmonella enterica or with a CD-associated Escherichia coli pathobiont (7, 8). Importantly, progression from intestinal inflammation to inflammation-associated fibrosis is incompletely penetrant in bacterium-induced colitis models and in clinical populations with microbe-driven diseases like IBD. It remains unclear which microbiota-derived signals favor the establishment of a profibrogenic microenvironment.

Members of the intestinal microbiota are key modulators of mucosal immunity under homeostatic conditions and in numerous inflammatory pathologies, including IBD (1). A subset of resident intestinal E. coli organisms known as adherent/invasive E. coli (AIEC) is enriched in CD patients (9–11). AIEC breaches the intestinal epithelium and induces inflammation in various rodent models of experimental colitis (12–15). Colonization of germfree, inflammation-prone *Il10*^−/−^ mice with AIEC induces aggressive, transmural intestinal inflammation driven by bacterial antigen-specific T-helper 1 (Th1) and Th17 immune responses (13, 16). Studies with germfree *Il10^−/−^* mice individually colonized with AIEC have led to the identification of several bacterial factors that augment or diminish the colitis-inducing and procarcinogenic capabilities of AIEC (17–20).

Comparative phylogenetic studies have demonstrated that the yersiniabactin (Ybt) high-pathogenicity island (HPI) is overrepresented in human, canine, and murine AIEC strains (21). The Ybt HPI encodes enzymatic machinery required for the biosynthesis of the siderophore Ybt (22). Once Ybt is released from bacterial cells, it sequesters extracellular metals, including iron, zinc, and copper. The Ybt-metal chelate is subsequently imported through its cognate outer membrane receptor FyuA for bacterial use (22–24). The Ybt HPI is harbored by numerous *Enterobacteriaceae* pathogens and contributes to *in vivo* fitness, niche formation, and virulence (25–27). However, the contribution of the Ybt HPI to the proinflammatory potential of resident intestinal E. coli such as AIEC has not been explored, despite its prevalence in this population. We therefore utilized our gnotobiotic *Il10^−/−^* mouse model to investigate whether inactivation of the Ybt system in AIEC modulates immune-mediated colitis. While abrogation of Ybt biosynthesis in AIEC delayed colitis onset, colonization of mice with Ybt-positive AIEC was associated with the development of inflammation-associated fibrosis. Severity of fibrosis was enhanced in mice colonized with the Ybt-positive transport mutant (Δ*fyuA*), which corresponded with increased profibrogenic gene signatures in the colon and in cultured fibroblasts and enhanced AIEC subepithelial localization within fibrotic lesions. Abrogation of Ybt biosynthesis in the Δ*fyuA* mutant attenuated fibrosis in inflamed mice, restored AIEC localization to the epithelium, and reduced fibroblast activation. Collectively, our findings introduce a noncanonical role for Ybt in mediating fibrosis development independent of its established function in delivering iron to bacteria through FyuA. More broadly, we introduced a novel microbe-driven, immune-mediated model of inflammation-associated fibrosis that recapitulates key histopathological features of fibrotic disease in human CD.

## RESULTS

### Inactivation of Ybt biosynthesis, but not Ybt transport, in AIEC delays progression of colitis.

**FIG 1:**
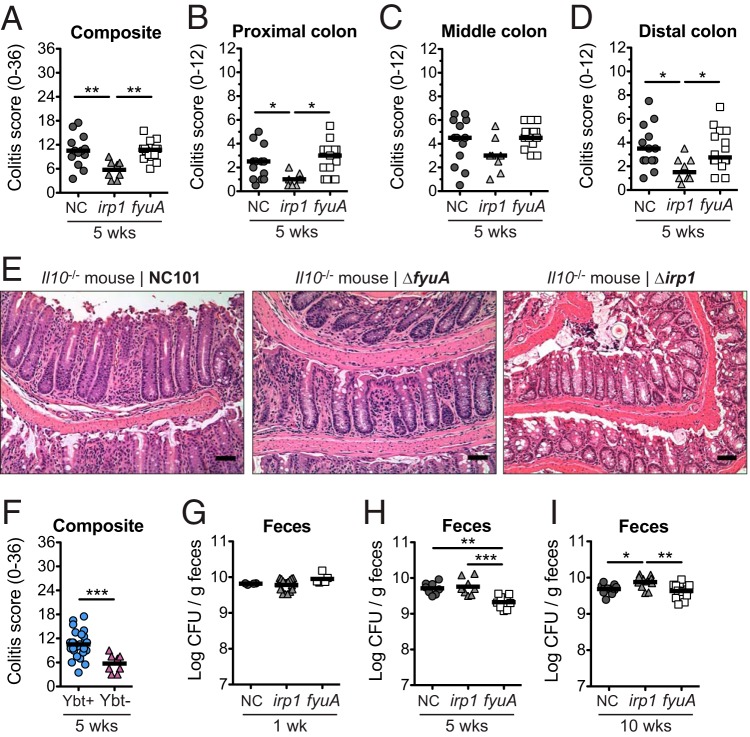
Yersiniabactin enhances the proinflammatory potential of AIEC in gnotobiotic *Il10*^−/−^ mice. Germfree *Il10*^−/−^ mice were monoassociated with the AIEC strain E. coli NC101 (NC) or the Δ*fyuA* or Δ*irp1* mutant for 5 weeks. (A to D) Composite (A) and regional (B to D) histopathology colitis scores. (E) Representative H&E histology of the colon. Scale bar, 50 μm. (F) Composite histopathology colitis scores of *Il10*^−/−^ mice colonized with yersiniabactin (Ybt)-positive or Ybt-deficient NC101. Lines are at the medians. *P* values were determined by Kruskal-Wallis or Mann-Whitney test. (G to I) Quantitative bacterial culture from feces at 1 week (G), 5 weeks (H), or 10 weeks (I) postcolonization. Lines are at the means. *P* values were determined by one-way analysis of variance (ANOVA). Each symbol represents an individual mouse (*n* = 8 to 14). *, *P* < 0.05; **, *P* < 0.01; ***, *P* < 0.001.

The siderophore Ybt and its cognate receptor FyuA mediate bacterial metal acquisition in pathogenic *Enterobacteriaceae*. Because the Ybt HPI is also harbored by many IBD-associated AIEC strains, we hypothesized that like its pathogenic counterparts, the Ybt HPI enhances the proinflammatory potential of AIEC. To determine whether an intact Ybt siderophore system in AIEC contributes to colitis development, we inactivated Ybt biosynthesis or import by creating isogenic mutants unable to import Ybt-metal chelates (Δ*fyuA*) or unable to synthesize Ybt (Δ*irp1*) in the AIEC strain NC101 (which also harbors the enterobactin and salmochelin siderophore systems). We colonized germfree, inflammation-susceptible *Il10^−/−^* mice with NC101 or the Δ*fyuA* or Δ*irp1* mutant and compared the severities of colitis induction. At 5 weeks, colitis histopathology was significantly attenuated in mice colonized with the Δ*irp1* mutant compared with Ybt^+^ NC101 and the Δ*fyuA* mutant (Fig. 1A to E), an attenuation that was no longer apparent by 10 weeks (see Fig. S1 in the supplemental material). In contrast, colitis development did not differ in mice colonized with NC101 versus the Δ*fyuA* mutant. Colitis scores differences did not correlate with altered expression of proinflammatory cytokines known to correlate with disease in this model (Fig. S1) (13, 16). The reduced colitis potential of the Δ*irp1* mutant did not correspond with diminished luminal growth in the gut (Fig. 1G to I) or *in vitro* growth defects under iron-replete or -limiting conditions (Fig. S2). While the Δ*fyuA* mutant exhibited a growth defect at 5 weeks, its attenuated growth was not sustained throughout colitis development and did not correlate with colitis severity (Fig. 1G to I). Together, these findings demonstrate that Ybt enhances the proinflammatory potential of AIEC in gnotobiotc, inflammation-susceptible hosts.

### Ybt-positive AIEC promotes fibrosis development in inflamed *Il10^−/−^* mice.

**FIG 2:**
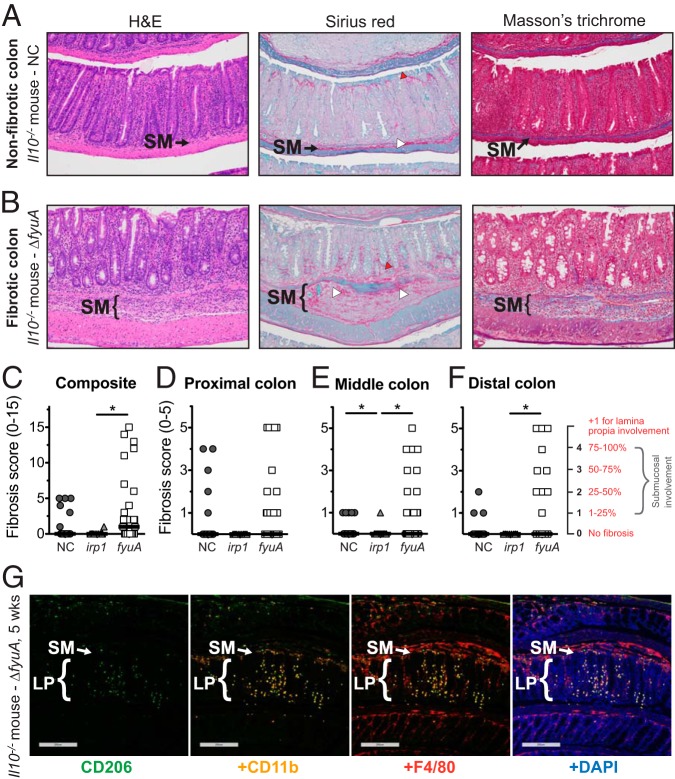
Ybt^+^ AIEC promotes fibrosis development in colitic *Il10*^−/−^ mice. Germfree *Il10*^−/−^ mice were monoassociated with the Ybt^+^ AIEC strain NC and the Δ*fyuA* mutant or the Ybt^−^ Δ*irp1* strain for 10 weeks. (A and B) Representative colonic histology of gnotobiotic *Il10*^−/−^ mice colonized with NC (A) or the Δ*fyuA* mutant (B). Colon sections were stained with H&E, Sirius red/fast green, or Masson’s trichrome. Regions of Sirius red binding are indicated by white arrowheads in the submucosa and red arrowheads in the lamina propria. (C to F) Composite (C) and regional (D to F) fibrosis histology scores. Each symbol represents an individual mouse (*n* = 11 to 29). Lines are at the medians. *P* values were determined by Kruskal-Wallis test. *, *P* < 0.05. (G) Representative colonic histology from gnotobiotic *Il10*^−/−^ mice colonized with the Δ*fyuA* mutant for 5 weeks. Colonic sections were stained with antibodies against the established macrophage cell surface markers CD206, CD11b, and F4/80 and were counterstained with the DNA stain 4′,6-diamidino-2-phenylindole (DAPI). Scale bars, 200 μm. SM, submucosa; LP, lamina propria.

In a subset of NC101- and Δ*fyuA* mutant-colonized inflamed *Il10^−/−^* mice, but rarely in Δ*irp1* mutant-colonized *Il10^−/−^* mice, pathological remodeling of the colonic submucosa was observed in hematoxylin and eosin (H&E)-stained colon sections (Fig. 2 and Fig. S3). Histological features consistent with fibrosis, including marked expansion of the submucosa with excessive deposition of lightly eosinophilic, fibrillar substances, characterized the pathology. Positive staining with Masson’s trichrome and Sirius red confirmed the presence of collagen fibers as part of the expanded ECM in fibrotic mice (Fig. 2B). Lamina propria collagen localization was also altered in fibrotic mice, exhibiting a basal predilection. In contrast, in nonfibrotic AIEC-colonized *Il10^−/−^* mice, the submucosal ECM was structured and organized and stained collagen fibrils in the lamina propria exhibited an apical propensity (Fig. 2A). Taken together, our results show that a subset of AIEC-colonized *Il10*^−/−^ mice developed histopathological lesions that are consistent with fibrosis.

**FIG 3:**
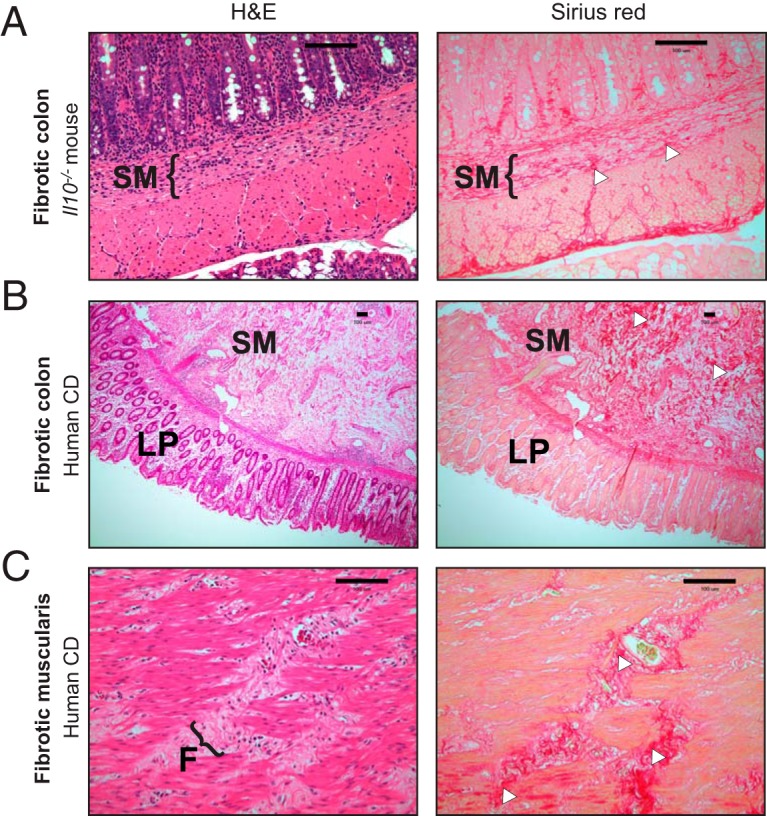
Fibrosis development in AIEC-colonized *Il10*^−/−^ mice recapitulates histopathological features of fibrosis in human Crohn’s disease. (A) Representative colonic histology of Δ*fyuA* mutant-colonized fibrotic *Il10*^−/−^ mice. (B and C) Representative histology of full-thickness colon cross sections from fibrotic Crohn’s disease patients, representative of 3 per group. (C) Magnification of the muscularis serosa. Colon sections were stained with H&E or Sirius red. Regions of Sirius red binding are indicated with white arrowheads. F, fibrotic lesion. Scale bars, 100 μm.

Because fibrosis incidences seemed to differ between NC101-, Δ*fyuA* mutant-, and Δ*irp1* mutant-colonized *Il10^−/−^* mice, we next utilized a fibrosis pathology scoring system to determine whether the Ybt system in AIEC impacts inflammation-associated fibrosis (28, 29) (see Materials and Methods). The most severe fibrosis pathology in all regions of the colon were observed in Δ*fyuA* mutant-colonized *Il10^−/−^* mice, which corresponded with higher incidence of severe disease. In contrast, moderate to severe fibrosis in NC101-colonized mice was mostly restricted to the proximal colon (Fig. 2C to F). These differences in fibrosis severity and incidence were associated with altered cellular populations infiltrating the submucosa, with immunologically defined macrophages (CD206^+^, CD11b^+^, and/or F4/80^+^ cells) observed in Δ*fyuA* mutant-colonized fibrotic mice (Fig. 2G) versus the inflammatory lymphocytes consistently observed in NC101-colonized, nonfibrotic mice (20). Inflamed *Il10^−/−^* mice colonized with the Ybt-deficient Δ*irp1* mutant did not develop moderate to severe fibrotic lesions and rarely exhibited mild disease (Fig. 2A to D), suggesting a role for Ybt in inducing and exacerbating this pathology. To validate that the histopathology in our mouse model is consistent with inflammation-associated fibrosis in human CD, we evaluated H&E and Sirius red staining of full-thickness colon resection tissues from fibrotic CD, ulcerative colitis, diverticulitis, and healthy margins of colorectal cancer resections (Fig. 3 and Fig. S5). Fibrotic CD tissues exhibited remarkable similarity to our mouse model, with transmural inflammation, expansion of the submucosa, thick collagen fibrils, and disruption of the muscularis by collagen and infiltrating cells (Fig. 3). Fibrosis was not evident by H&E, Sirius red, or Masson’s trichrome or at 5 weeks in *Il10^−/−^* mice (data not shown). Collectively, these observations demonstrate that Ybt^+^ AIEC promotes the development of fibrotic disease in an experimental model of pathobiont-induced colitis.

Because fibrosis occurs in response to tissue injury instigated by inflammation, we next determined whether fibrosis severity positively correlates with inflammation. Linear regression analysis revealed a significant negative correlation between fibrosis and colitis histopathology in the middle colon and no significant correlations in the proximal and distal colon (Fig. S4). Moreover, NC101- and Δ*fyuA* mutant-colonized mice exhibited similar levels of colitis histopathology despite the exacerbated fibrosis observed in Δ*fyuA* mutant-colonized mice (Fig. 1 and 2). Nonetheless, as previously reported (4), inflammation is required for the profibrotic activities of NC101 and Δ*fyuA* given that fibrosis was not observed in uninflamed wild-type (WT) mice colonized with either strain (Fig. S3). These results demonstrate that while inflammation is required for fibrosis development, Ybt^+^ AIEC exacerbates inflammation-associated fibrosis independent of effects on the proinflammatory potential of AIEC.

### Fibrosis development corresponds with enhanced subepithelial invasion of *fyuA*-deficient AIEC.

**FIG 4:**
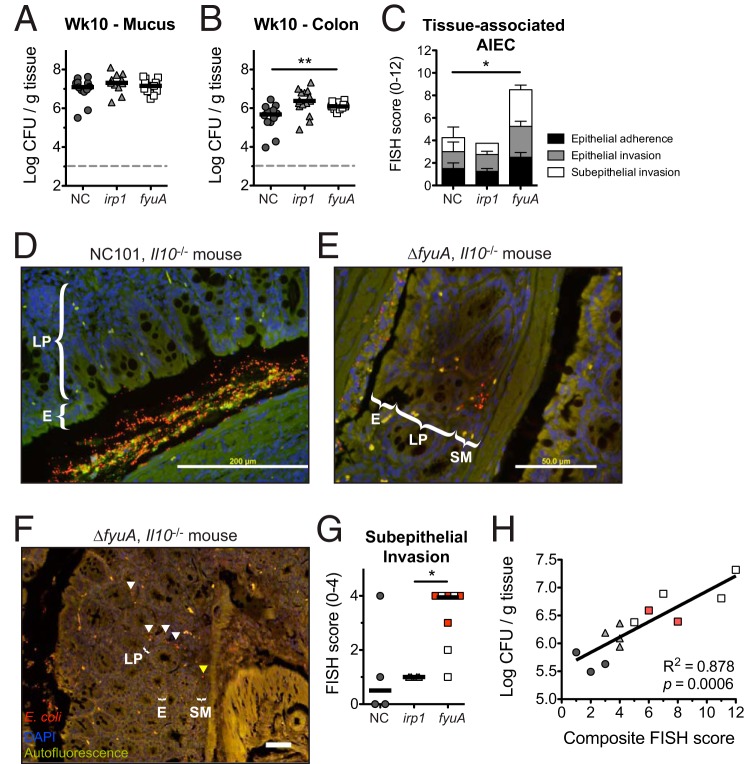
Inactivation of yersiniabactin transport enhances AIEC mucosal invasion. Germfree *Il10*^−/−^ mice were monoassociated with NC or the Δ*fyuA* or Δ*irp1* mutant for 10 weeks. (A and B) Quantitative bacterial cultures of colonic mucus (A) and colonic (B) tissues. Each symbol represents an individual mouse (*n* = 11 to 15). Lines are at the medians. *P* values were determined by Kruskal-Wallis test. (C) FISH analysis of proximal colons (*n* = 4 to 8). *P* values were determined by Kruskal-Wallis test. (D to F) Representative FISH images of the proximal colon. Red, E. coli; blue, DAPI. Arrowheads in panel F indicate E. coli localized within the lamina propria (white) and submucosa (yellow). E, epithelium. Scale bars, 200 μm. (G) Subepithelial AIEC invasion scores as assessed by FISH in panel C. Red squares represent fibrotic mice as assessed by histopathology. Lines are at the medians. *P* values were determined by Kruskal-Wallis test. (H) Linear regression analysis of quantitative bacterial culture versus FISH score from colonic tissues. Red squares represent fibrotic mice as assessed by histopathology. *, *P* < 0.05; **, *P* < 0.01.

We next determined whether the profibrogenic potential of Ybt^+^ AIEC corresponds with altered bacterial localization within the intestines. While colonic mucus colonization did not differ between the strains, colonic tissue loads of AIEC were significantly increased in Δ*fyuA* mutant-colonized mice at 10 weeks (Fig. 4A and B). In contrast, colonic tissue colonization did not differ between NC101 and the Δ*irp1* mutant. Colonic mucus and tissue loads were also comparable at 5 weeks (Fig. S6A and B). Because AIEC is functionally characterized by epithelial invasiveness, intramacrophagic survival, and robust biofilm formation, we performed standard *in vitro* assays commonly utilized to distinguish AIEC strains (9). While iron availability altered AIEC epithelial invasion, no differences in epithelial adherence or invasion were observed between NC101 and the Δ*irp1* and Δ*fyuA* mutants under iron-replete or -limiting conditions (Fig. S6C and D). Similarly, genetic ablation of Ybt transport did not alter macrophage phagocytosis or intracellular survival of AIEC (Fig. S6E to G) and had no effect on AIEC biofilm formation (Fig. S6H). Thus, while Ybt transport and biosynthesis did not impact defining *in vitro* characteristics of AIEC, deletion of *fyuA* enhanced AIEC colonic tissue colonization, suggesting that FyuA may be important in modulating bacterial localization within the intestines.

To further assess how FyuA impacts AIEC localization in the gut, we employed a more sensitive approach—E. coli 16S fluorescence *in situ* hybridization (FISH)—to visualize tissue-associated AIEC. FISH analysis revealed an overall increase in the tissue-associated Δ*fyuA* mutant relative to NC101 and the Δ*irp1* mutant (Fig. 4C). This difference was primarly driven by enhanced subepithelial (lamina propria and submucosa) localization of Δ*fyuA* (Fig. 4D to G). Moreover, Δ*fyuA* was observed within submucosal fibrotic lesions, demonstrating its colocalization with diseased tissue (Fig. 4F, arrowheads). Importantly, tissue bacteria loads assessed by quantitative bacterial culture and FISH analysis were positively correlated (Fig. 4H). Together, these results suggest that inactivation of *fyuA* enhances the subepithelial localization of AIEC, which may contribute to its profibrogenic potential.

### Inactivation of Ybt-mediated metal acquisition does not alter AIEC iron sensing.

The canonical function of Ybt is to scavenge extracellular metals for bacterial use (22, 30). Because the most severe fibrosis occurred in mice colonized with the Δ*fyuA* mutant, we first assessed whether Ybt functionality was altered in this mutant. The extents of Ybt secretion were comparable between NC101 and the Δ*fyuA* mutant, and as expected, Ybt secretion was not detected in the Δ*irp1* Ybt biosynthesis mutant (Fig. S7a). We next confirmed the functionality of Ybt produced by NC101 and the Δ*fyuA* mutant. To accomplish this, we assessed whether Ybt produced by these strains can restore the growth of siderophore-deficient Klebsiella pneumoniae (Δ*entB irp1*) cultivated under iron-limiting conditions. In contrast to the Δ*irp1* mutant, both Ybt^+^ NC101 and the Δ*fyuA* mutant rescued K. pneumoniae Δ*entB irp1* mutant growth (Fig. S7b). Taken together, these data suggest that altered Ybt functionality does not correspond with the increased profibrogenic potential of the Δ*fyuA* mutant.

Mutants lacking FyuA are unable to import Ybt-iron chelates and may therefore be unable to satisfy their iron requirements. Thus, the enhanced profibrogenic potential of the Δ*fyuA* mutant may be the result of altered bacterial function mediated through disrupted bacterial iron homeostasis. To test this idea, we first compared *in vivo* expressions of iron-responsive genes in NC101 and the Δ*fyuA* and Δ*irp1* mutants. Transcript levels of several iron-responsive genes did not differ between strains (Fig. S8A and B), suggesting that NC101 iron homeostasis is not perturbed upon inactivation of Ybt transport or biosynthesis in the intestines. Similarly, *in vitro* iron depletion with the iron chelator 2,2′-bipyridyl (BPD) did not alter transcription of iron-responsive genes (Fig. S8C).

To corroborate these results, we performed transcriptional reporter assays utilizing vectors harboring *gfp* fused to the iron-responsive promoter P*_tonB_*. To first validate this approach, the NC101 reporter strain was cultivated under iron-replete and -limiting conditions, and as expected, iron depletion enhanced *gfp* activity driven by the *tonB* promoter (Fig. S9a). We next assessed whether NC101, Δ*fyuA*, and Δ*irp1* iron-sensing reporters respond differently to iron depletion. In agreement with our transcriptional results, levels of *gfp* expression were comparable between NC101 and the Δ*fyuA* mutant (Fig. S9a), suggesting that inactivation of FyuA does not impact AIEC iron sensing. Because Ybt can also bind other metals, including zinc (22, 31; our own observations) and copper (our own observations) (23, 24; our own observations), we performed similar assays with the zinc-responsive promoter P*_znuA_* and the copper-responsive promoter P*_cusC_*. As with the iron-sensing reporters, altering zinc and copper availability did not alter sensing of the respective metals in the Δ*fyuA* mutant relative to that in NC101 (Fig. S9b and c). In contrast, the activities of iron- and zinc-responsive promoters were significantly increased in the Δ*irp1* mutant (Fig. S9a and b), suggesting that metal starvation is enhanced in this mutant under iron- and zinc-limiting conditions. Taken together, these data suggest that while metal sensing in AIEC is not altered with disruption of Ybt transport, metal homeostasis appears to be disrupted in the Ybt-negative Δ*irp1* mutant.

### Deletion of *fyuA* in AIEC promotes the establishment of a profibrotic colonic environment that precedes fibrosis development.

**FIG 5:**
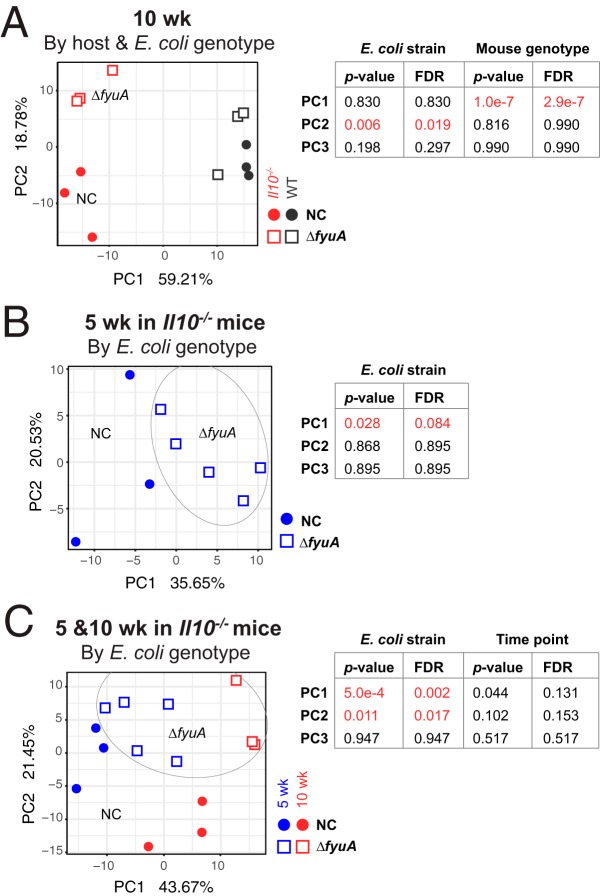
Deletion of *fyuA* in AIEC promotes transcriptome-wide changes in the colons of *Il10*^−/−^ mice. Shown are the results of principal-component analysis of transcriptome-wide changes in the colons of NC- versus Δ*fyuA* mutant-colonized WT or *Il10^−/−^* mice after 10 weeks (A), NC- versus Δ*fyuA* mutant-colonized *Il10^−/−^* mice after 5 weeks (B), and NC- versus Δ*fyuA* mutant-colonized *Il10^−/−^* mice after 5 and 10 weeks (C).

The increased incidence of fibrosis in Δ*fyuA* mutant-colonized *Il10^−/−^* mice may in part be driven by differential host responses to *fyuA*-expressing versus *fyuA*-deficient AIEC. To test this idea, we utilized high-throughput RNA sequencing (RNA-seq) to determine whether global differences are apparent in the colonic transcriptomes of inflamed *Il10^−/−^* mice and noninflamed WT mice colonized with NC101 or Δ*fyuA*. Principal-coordinate analysis (PCoA) revealed significant differences in the colonic transcriptomes of NC101-colonized nonfibrotic versus Δ*fyuA* mutant-colonized fibrotic *Il10^−/−^* mice at 10 weeks, when fibrosis is apparent (Fig. 5A). This corresponded with 2,692 genes and 71 Kyoto Encyclopedia of Genes and Genomes (KEGG) pathways that were differentially expressed between Δ*fyuA* mutant- versus NC101-colonized *Il10^−/−^* mice (Tables S3 and S4). In contrast, the transcriptomes of NC101- versus Δ*fyuA* mutant-colonized WT mice clustered together (Fig. 5A), suggesting that differences in the host transcriptional responses to either strain predominantly occur in *Il10^−/−^* mice. To determine whether the differing host responses precede histological evidence of fibrosis, we also compared the colonic transcriptomes of NC101-colonized versus Δ*fyuA* mutant-colonized *Il10^−/−^* mice at 5 weeks. RNA-seq analysis revealed that 169 genes and 116 KEGG pathways were differentially expressed in NC101-colonized versus Δ*fyuA* mutant-colonized *Il10^−/−^* mice (Tables S1 and S2), many of which were differentially regulated at both 5 and 10 weeks. However, testing overall community composition did not show statistical significance after false-discovery rate (FDR) correction (*P* < 0.084) (Fig. 5B). Thus, the presence of *fyuA* in AIEC significantly altered host transcriptional responses in the inflamed colon prior to and throughout the development of fibrosis.

**FIG 6:**
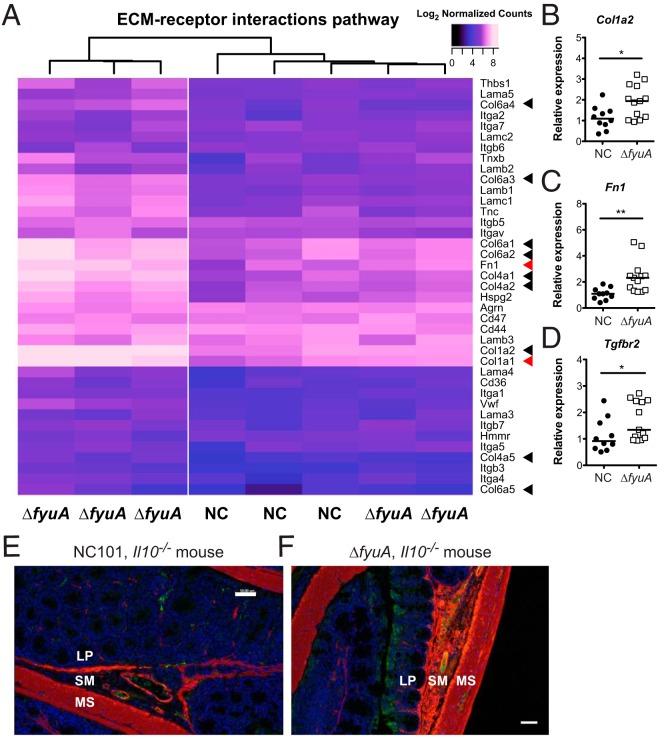
Deletion of *fyuA* in AIEC promotes profibrotic host responses preceding fibrosis development. (A) Heat map of log2 normalized counts of genes in the ECM-receptor interaction KEGG pathway in NC- and Δ*fyuA* mutant-colonized *Il10^−/−^* mice at 5 weeks. (B to D) Relative colonic transcript levels of *Col1a1* (B), *Fn1* (C), and *Tgfbr2* (D) in *Il10*^−/−^ mice monoassociated with NC or the Δ*fyuA* mutant for 5 weeks. Each symbol represents an individual mouse (*n* = 10 to 13). Lines are at the medians. *P* values were determined by Mann-Whitney test. *, *P* < 0.05; **, *P* < 0.01. (E and F) Proximal colons from *Il10*^−/−^ mice colonized with NC (E) or the Δ*fyuA* mutant (F) for 10 weeks were stained with α-SMA (red), CD31 (green), or DAPI. MS, muscularis serosa. Scale bars, 50 μm.

Transcriptomic analysis of a prospectively followed inception cohort of pediatric CD patients revealed high expression of profibrogentic genes and pathways prior to the development of stricturing fibrotic disease (32). This included ECM structural constituents and collagen binding pathways (32). In agreement with these results, the ECM-receptor interaction KEGG pathway is significantly upregulated in Δ*fyuA* mutant-colonized mice during (10 weeks) and prior to (5 weeks) histological evidence of fibrosis (Tables S2 and S4). We generated a heat map to visualize expression of individual genes in this KEGG pathway between individual NC101- versus Δ*fyuA* mutant-colonized *Il10^−/−^* mice (Fig. 6A). Phylogenetic clustering of the 5-week samples demonstrated that three of the Δ*fyuA* mutant-colonized mice clustered together and exhibited increased expression of numerous ECM genes, including type I, IV, and VI collagens and fibronectin (arrowheads, Fig. 6A). Careful histological observation by a pathologist blinded to the treatment groups revealed early evidence of fibrosis in these three Δ*fyuA* mutant-colonized mice but not in the remaining Δ*fyuA* mutant-colonized mice that clustered with the NC101-colonized mice and exhibited lower expression of ECM genes. These unbiased molecular findings are consistent with our observation that a subset, and not 100%, of Δ*fyuA* mutant-colonized mice develop fibrosis. These findings were confirmed by targeted quantitative PCR analysis, in which transcript levels of *col1a2* (type 1 collagen) and *fn1* (fibronectin) were significantly increased in *fyuA* mutant- versus NC101-colonized *Il10^−/−^* mice (Fig. 6B and C). This corresponded with increased positivity of α-SMA (smooth muscle actin), a common feature of fibrosis, in Δ*fyuA* mutant-colonized *Il10*^−/−^ mice (Fig. 6E and F). Taken together, these findings demonstrate that ECM components are upregulated in prefibrotic *Il10*^−/−^ mice colonized with the Δ*fyuA* mutant prior to the development of fibrotic disease.

Transforming growth factor β (TGF-β) signaling represents the canonical profibrotic activation pathway. Therefore, to further confirm the presence of a profibrotic gene signature in fibrotic mice, we evaluated the expression of genes within the TGF-β pathway. RNA-seq analysis detected colonic expression of the three TGF-β and TGF-β receptor isoforms. Prefibrotic (5 weeks) Δ*fyuA* mutant- versus NC101-colonized *Il10^−/−^* mice trended toward elevated expression of TGF-β1 and TGF-β3 and TGF-β receptor isoforms 1 and 2 (Fig. S10A). Increased expression of the TGF-β2 receptor was confirmed by quantitative PCR (Fig. 6D). Similarly, a significant increase in TGF-β1 to -β3 and TGF-β receptor isoform 2 and 3 expression was observed at 10 weeks in fibrotic Δ*fyuA* mutant-colonized *Il10^−/−^* mice (Fig. S10b). Together, these data further support our hypothesis that deletion of *fyuA* in AIEC promotes a profibrogenic environment in inflammation-susceptible hosts, which occurs at an early phase of the inflammatory response prior to onset of fibrosis.

### Ybt-dependent fibrosis is not associated with altered host systemic iron homeostasis.

Membrane-permeative siderophores like Ybt disrupt host iron homeostasis and modulate iron-sensitive host responses, which includes the induction of *Ndrg1* (33, 34). Because deletion of *fyuA* does not alter Ybt secretion, colonization with the Δ*fyuA* mutant may instead increase Ybt internalization by host cells in the absence of bacterial import and alter host iron homeostasis to promote fibrosis. To address this possibility, we determined whether colonization with the Δ*fyuA* mutant versus NC101 or the Δ*irp1* mutant alters systemic iron homeostasis in *Il10^−/−^* mice. At 2 weeks (prior to histological inflammation or fibrosis) and at 10 weeks (when colitis and fibrosis are evident in affected animals), plasma hemoglobin levels did not differ (Fig. S11A and B). Similarly, Prussian blue staining did not reveal differences in splenic iron stores at 10 weeks (Fig. S11C). To determine whether local iron homeostasis was altered in the colon, we utilized our RNA-seq data to assess whether established host iron-responsive genes were differentially expressed in mice colonized with NC101 or the Δ*fyuA* mutant (Table S5) (35–38). Of the 15 canonical iron-responsive genes investigated, three were differentially regulated in *Il10^−/−^* mice, including *Ndrg1* and *Tfrc* (transferrin receptor), and two were differentially regulated in WT mice, including *Tfrc*, at 10 weeks. At 5 weeks, *Epas1* was the only iron-responsive gene that was altered between NC101- versus Δ*fyuA* mutant-colonized *Il10^−/−^* mice, a change not observed at 10 weeks. Together, these findings suggest that the Δ*fyuA* mutant does not profoundly alter systemic or colonic iron homeostasis in the host and may not be a driving factor for fibrosis induction.

### Yersiniabactin biosynthesis is required for AIEC-mediated fibrosis induction.

**FIG 7:**
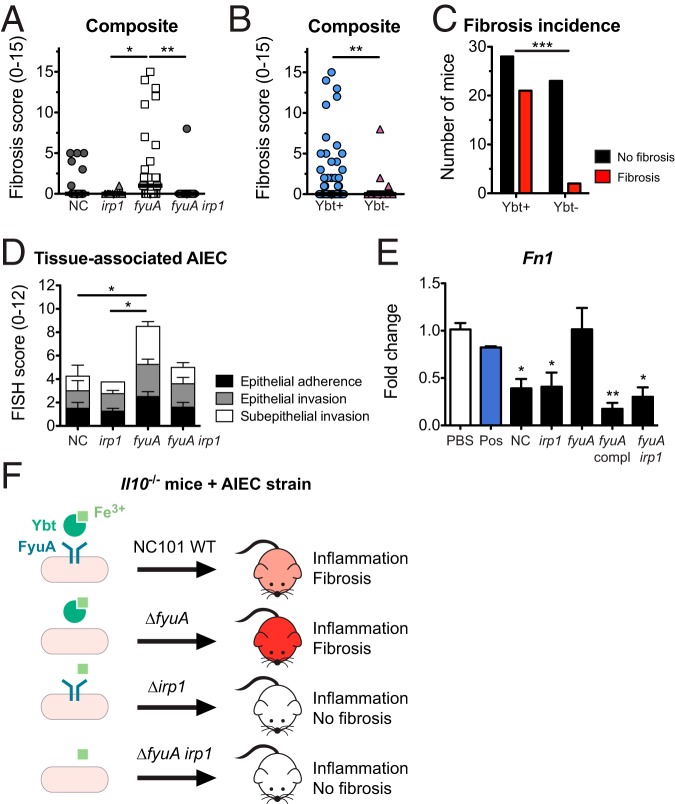
Yersiniabactin biosynthesis promotes fibrosis in AIEC-driven colitis. Germfree *Il10*^−/−^ mice were monoassociated with the AIEC strain NC or the Δ*fyuA*, Δ*irp1*, or Δ*fyuA irp1* mutant for 10 weeks. (A) Composite fibrosis histology scores. Each symbol represents an individual mouse (*n* = 10 to 29). Lines are at the medians. *P* values were determined by Kruskal-Wallis test. (B) Composite histopathology colitis scores of *Il10*^−/−^ mice colonized with Ybt-positive or Ybt-deficient AIEC. Lines are at the medians. *P* values were determined by Mann-Whitney test. (C) Fibrosis incidence rates of *Il10*^−/−^ mice colonized with Ybt-positive or Ybt-deficient AIEC as assessed by H&E histology. *P* values were determined by Fisher’s exact test. (D) FISH analysis of proximal colons (*n* = 4 to 8). *P* values were determined by one-way ANOVA. (E) Swiss 3T3 fibroblasts were cocultured with NC or the Δ*irp1*, Δ*fyuA*, Δ*fyuA* chromosomally complemented with *fyuA* under its native promoter, or Δ*fyuA irp1* mutant. Fibroblasts stimulated with TGF-β served as a positive control (Pos). Data are represented as the means ± SEMs. *P* values were determined by Kruskal-Wallis test. (F) Working model. Data for NC-, Δ*fyuA* mutant-, and Δ*irp1* mutant-colonized mice are also presented in Fig. 2 and 4. *, *P* < 0.05; **, *P* < 0.01; ***, *P* < 0.001.

Abrogation of Ybt transport in AIEC had opposing effects on fibrosis induction in *Il10^−/−^* mice compared to the inactivation of Ybt biosynthesis (Fig. 2). Because fibrosis development was minimal in *Il10^−/−^* mice colonized with the Δ*irp1* mutant, we next determined whether Ybt biosynthesis is required for the fibrosis-inducing potential of the Δ*fyuA* mutant. Genetic inactivation of Ybt biosynthesis in the Δ*fyuA* mutant (Δ*fyuA irp1*) significantly reduced fibrosis incidence in *Il10^−/−^* mice (Fig. 7A to C). Moreover, when comparing fibrosis incidence in mice colonized with Ybt-positive versus Ybt-negative AIEC, we noted that 22 out of 51 mice colonized with Ybt-positive AIEC developed fibrotic disease, whereas 3 out of 26 mice colonized with Ybt-deficient AIEC exhibited histological evidence of fibrosis (Fig. 7B). Inactivation of Ybt production in the Δ*fyuA* mutant also reduced its subepithelial invasiveness, resulting in a pattern of tissue localization similar to that of NC101 (Fig. 7D). This further reinforces the link between increased mucosal invasiveness and the enhanced profibrogenic potential of the Δ*fyuA* mutant. Importantly, colitis severities at 10 weeks were comparable between the Δ*fyuA* and Δ*fyuA irp1* mutants (Fig. S12), suggesting that differences in inflammation were not driving fibrosis severity.

To further demonstrate the profibrogenic potential of the Δ*fyuA* mutant, we next determined whether inactivation of Ybt transport in AIEC enhances the activation of cultured fibroblasts *in vitro*. Fibroblasts that were cultured with the Δ*fyuA* mutant expressed significantly higher levels of the fibroblast activation marker *Fn1* than fibroblasts cultured with the parental and Ybt-deficient strains (Fig. 7E). This corresponded with our *in vivo* observation that *Fn1* transcripts were elevated in Δ*fyuA* mutant-colonized *Il10^−/−^* mice (Fig. 6C). Together, these results demonstrate that inactivation of the Ybt siderophore system in AIEC in two distinct manners (i.e., Ybt transport versus Ybt biosynthesis) does not have similar effects on colitis induction and fibrosis development in genetically susceptible hosts. More broadly, in addition to its role in bacterial iron acquisition, our findings collectively introduce a novel, noncanonical role of Ybt in establishing a profibrogenic microenvironment in inflammation-susceptible hosts.

## DISCUSSION

Siderophore biosynthetic gene clusters are abundant in the gut microbiota, with 232 putative clusters identified from metagenomes in the Human Microbiome Project study (39). Given that IBD-associated AIEC strains also harbor many of these siderophore systems (21), it is conceivable their siderophores may contribute to AIEC-associated intestinal disease. Indeed, here we introduce the siderophore Ybt as a novel bacterial factor that promotes profibrogenic host responses in the inflamed intestinal environment. Our findings demonstrate that AIEC is profibrogenic and that inactivation of Ybt transport in a colitogenic AIEC strain enhances fibrosis development in inflammation-susceptible mice. Inactivation of Ybt biosynthesis in both the Ybt transport mutant and the parental strain abrogates their fibrosis-inducing potential, suggesting that Ybt promotes fibrosis development even in the absence of uptake through its canonical receptor. Profibrogenic transcriptional signatures are evident in the colon prior to histological presentation of disease, suggesting a causative role for Ybt-mediated induction of fibrosis. Together, our findings introduce a specific microbiota-derived factor that promotes the development of inflammation-associated fibrosis.

The canonical function of the Ybt siderophore system is to import extracellular iron sequestered by Ybt through FyuA for bacterial use. Thus, inactivation of FyuA may enhance the profibrogenic potential of AIEC by perturbing bacterial iron homeostasis and subsequently modulating bacterial function. However, levels of luminal expression of highly sensitive, iron-responsive genes (30, 40) were comparable between the NC101 parental strain, the Δ*fyuA* transport mutant, and the Δ*irp1* Ybt-deficient mutant. This indicates a lack of strain-specific differences in iron sensing. Similarly, while the functional outcome of iron starvation in bacteria is a fitness disadvantage (30, 41), we observed no prolonged differences in luminal colonization between NC101 and the Δ*fyuA* and Δ*irp1* mutants in the noninflamed intestines or during the course of inflammation and fibrosis development. This is likely the result of additional iron scavenging systems in NC101 that play compensatory roles in the Ybt-deficient and transport mutants (21, 42). Indeed, in other *Enterobacteriaceae* strains, inactivation of multiple iron acquisition systems is required to attenuate *in vivo* fitness (25, 30). Most importantly, if the enhanced profibrogenic potential of the Δ*fyuA* mutant was the result of dysregulated bacterial iron homeostasis and consequent effects on Ybt-independent functions, we would expect levels of fibrosis induction mediated by the Δ*irp1* and Δ*fyuA* mutants to be comparable, as both mutants cannot scavenge iron through Ybt (43). Instead, fibrosis induction was further attenuated in mice colonized with Ybt-deficient AIEC strains. Collectively, our findings support a model in which Ybt stimulates host profibrogenic responses through a mechanism independent of its role in importing iron through FyuA.

While the Ybt system did not impact overall AIEC intestinal fitness, inactivation of Ybt transport altered the distribution of AIEC colonization within colonic tissues. This may contribute to AIEC-driven fibrosis by activating myofibroblasts and mesenchymal cells either directly via bacterial recognition receptors (i.e., Toll-like receptors [TLRs]) or indirectly by activating intestinal immune cells that modulate profibrogenic cellular responses (5, 6). The Δ*fyuA* mutant was more abundant than the parental strain within the colonic subepithelium and colocalized with fibrotic lesions in *Il10^−/−^* mice at 10 weeks. In contrast, inactivation of Ybt biosynthesis did not alter tissue localization of AIEC, further uncoupling the effects of Ybt biosynthesis and Ybt transport on bacterial function. Instead, inactivation of Ybt biosynthesis in the Δ*fyuA* mutant restored tissue colonization patterns exhibited by the parental strain, suggesting that Ybt mediates the mislocalization of the Δ*fyuA* mutant to the subepithelium independent of its role in importing iron through FyuA. Consistent with our findings, several studies have also reported altered tissue localization of *Enterobacteriaceae* pathogens with inactivation of the Ybt system in extraintestinal mucosal environments (26, 44, 45). Finally, it should be noted that while we observed a statistically significant decrease in fecal colonization of the Δ*fyuA* mutant at 5 weeks, it remains unclear whether a <0.5-log difference in bacterial burdens in a monocolonized mouse can impart any meaningful effects on the host, especially as this decrease was also not observed at 1 or 10 weeks postcolonization. Collectively, these findings highlight one putative noncanonical function of Ybt that may enhance the profibrogenic potential of AIEC. The precise mechanisms by which inactivation of FyuA enhances fibrosis development in susceptible hosts will be the subject of future studies.

Because Ybt is a secreted bacterial product that permeates mammalian membranes, Ybt may also promote profibrogenic host responses by perturbing cellular iron homeostasis in the host. Indeed, the membrane-permeative siderophores enterobactin and yersiniabactin stimulate epithelial proinflammatory responses by decreasing intracellular iron pools, an effect that is reversed with the addition of iron (33, 34). Ybt disruption of local iron homeostasis may similarly drive fibrosis development by stimulating profibrogenic responses in epithelial, mesenchymal, and immune cells. While our host transcriptomics analyses demonstrated similar colonic expression profiles of numerous iron responsive host genes in NC101- versus Δ*fyuA* mutant-colonized mice (Table S5), differences in the canonical iron response genes *Ndrg1* and *Tfrc* were uniquely observed in *Il10^−/−^* mice. This suggests that local and/or cell-specific alterations in host iron homeostasis may contribute to the progression of fibrosis. However, as these changes were observed at 10 weeks but not 5 weeks postcolonization, the initiation of fibrosis and early profibrotic gene signatures cannot be attributed to major alterations in host iron homeostasis.

In addition to iron, Ybt is capable of binding other metals, including nickel, cobalt, chromium, gallium, and copper (46). This raises the possibility that its profibrogenic potential is the result of interactions with other metals present in the colonic environment. For example, when complexed with copper, Ybt acts to limit the lethal effects of macrophage reactive oxygen species (47). Ybt-copper chelates have been detected in urine samples from patients infected with uropathogenic E. coli (UPEC), demonstrating that Ybt binds copper *in vivo* (23). Bacterial cells can also import Ybt-copper chelates through FyuA (24, 31). Together, these findings introduce two putative mechanisms by which Ybt promotes fibrosis development: (i) through chelation of host sources of metals other than iron and/or (ii) by modulating the transcriptome and metabolome of AIEC by altering the flux of micronutrients into the bacterial cell. Finally, because the Ybt enzymatic machinery produces additional secreted metabolites that remain uncharacterized (48), it is intriguing to speculate that these Ybt precursors and Ybt-like molecules may also play a role in inflammation-associated fibrosis.

Fibrosis complicates many inflammatory intestinal disorders associated with microbial dysbiosis; however, profibrotic mechanisms remain incompletely understood and limit therapeutic strategies. This has been hampered by the lack of rodent models that recapitulate the complex interactions between host genetics and microbial factors important for inflammation and fibrosis development. Here we introduce a new model for inflammation-associated fibrosis driven by a pathobiont-derived small molecule produced from the Ybt pathogenicity island. Consistent with human CD (32), profibrogenic pathways are upregulated prior to histological presentation of fibrosis and mirror the incidence rate of fibrotic disease in our model. Moreover, our model recapitulates key histological and transcriptomic aspects of fibrotic disease in human CD. More broadly, our findings demonstrate that manipulating the same pathogenicity island in different ways can result in distinct consequences for disease development. This highlights an important difference in targeting siderophore biosynthesis versus the cognate receptors as putative bacterial targets in microbe-driven diseases such as CD. Furthermore, other siderophore and metallophore systems of the gut microbiota may induce similar responses and contribute to fibrosis. Given the prevalence of AIEC among the population with CD, the presence of the Ybt siderophore system could serve as a useful prognostic tool in identifying patient subsets susceptible to fibrotic disease.

## MATERIALS AND METHODS

### Bacterial strains.

The fecal isolate E. coli NC101 was isolated from WT mice (13). The λ-red recombinase system was utilized to generate mutants (49) (Table S7). Bacterial strains and plasmids are listed in Table S6.

### Mice.

Germfree *Il10*^−/−^ and WT 129S6/SvEV mice were maintained at the National Gnotobiotic Rodent Resource Center at the University of North Carolina at Chapel Hill (UNC-CH). Absence of isolator contamination was confirmed by Gram stain and fecal culture. Eight- to 12-week-old mice were inoculated via oral and rectal swabs with E. coli following overnight growth in LB broth (13). Colonization was confirmed by fecal plating. Five cohorts of *Il10*^−/−^ mice were colonized with the NC101 WT or the Δ*fyuA* mutant, and two cohorts of *Il10*^−/−^ mice were colonized with the Δ*irp1* or Δ*fyuA irp1* mutant. Animal protocols were approved by the UNC-CH Institutional Animal Care and Use Committee.

### Quantification of bacteria.

E. coli CFU in feces were quantified by serial dilutions and plating on LB plates. Mucus- and tissue-associated bacteria were enumerated as described previously (10).

### Colitis histopathology.

At necropsy, tissues were fixed in 10% neutral buffered formalin. Colon sections were stained with H&E, Masson’s trichrome, or Sirius red; spleens were stained with Prussian blue. Colitis scores (0 to 12) of Swiss-rolled colons were blindly assessed as described previously (13, 20). Composite scores (0 to 36) are the sums of proximal, middle, and distal colon scores.

### Fibrosis histopathology.

Fibrosis was blindly assessed on colonic H&E sections and validated by Sirius red. Severity of fibrosis (0 to 5) was evaluated using a validated scoring system (28, 29) assessing the extent of submucosal involvement: no fibrosis (score of 0) or fibrosis in <25% (score of 1), 26 to 50% (score of 2), 51 to 75% (score of 3), or 76 to 100% (score of 4) of the colon section. One point was added for lamina propria involvement. Composite scores (0 to 15) are the sums of the proximal, middle, and distal colon scores. A score of 0 was considered nonfibrotic, 1 to 3 represented mild fibrosis, and 4+ represented moderate/severe fibrosis. Histopathology was blindly confirmed by a small-animal veterinarian specializing in gastrointestinal histopathology.

### Human intestinal samples.

Formalin-fixed, paraffin-embedded tissue blocks from routine diagnostic surgical resections were transferred to UNC-CH under an approved Institutional Review Board protocol of the Cleveland Clinic. Sections were H&E and Sirius red stained from three individuals per disease category: CD, ulcerative colitis (UC), diverticulitis, and noninflamed controls (healthy margins of colorectal cancer patients).

### FISH.

Colons were washed in phosphate-buffered saline (PBS) to remove contents and loosely adhered bacteria. Formalin-fixed, paraffin-embedded sections were mounted on charged glass slides and incubated with an oligonucleotide probe directed against E. coli (Cy3-E. coli/*Shigella* probe) and an antisense probe (6-carboxyfluorescein [FAM]-non-EUB338) (50). Hybridized samples were washed in PBS, air dried, and mounted with ProLong antifade (Molecular Probes Inc.). Sections were examined on a BX51 epifluorescence microscope with an Olympus DP-7 camera. Fluorescent in situ hybridization (FISH) analysis was performed in a blind fashion by two independent investigators as follows: to assess bacterial colonization, we enumerated individual bacterial cells adhered to epithelial cells (epithelial attachment), localized within epithelial cells (epithelial invasion), and translocated across the epithelium (subepithelial invasion). The quantity of bacteria per colon Swiss roll was converted to a FISH score of 0 to 4 (Table 1).

**TABLE 1:** FISH scoring

FISH score	No. of bacteria demonstrating:		
	Epithelial attachment	Epithelial invasion	Subepithelial invasion
0	None	None	None
1	1–50	1–10	1–5
2	51–150	11–20	6–10
3	151–250	21–30	11–15
4	251+	31+	16+

### RNA-seq analysis.

RNA-seq reads were quality filtered at Q20 and trimmed to remove remaining adaptors using Trimmomatic (51) version 0.35. Resulting reads were aligned to the Illumina iGenome Mus musculus GRCm38 reference genome using Tophat (52) version 2.1.0 utilizing Bowtie2 (53) version 2.2.5. Resulting alignments were processed using Cufflinks (54) version 2.2.1 along with the Illumina iGenome Mus musculus GRCm38 Gene transfer format file, after masking rRNA features as described previously (55). Transcripts were quantified using cuffquant, and gene counts were exported to text files and then imported to edger (56) version 3.12.1 (running inside R version 3.2.3) for detecting differentially expressed genes. A gene was considered for differential-expression testing if present in at least three samples. We considered a gene differentially expressed if its edgeR FDR-adjusted *P* value was <0.05. Parallel analysis using featureCounts (57) from the subread package version 1.4.6 for transcript quantification showed similar results.

Pathway analysis was conducted using GAGE (58) version 2.20.1 using Mus musculus (mmu) Kyoto Encyclopedia of Genes and Genomes (KEGG) (59) pathways, and genes were mapped to KEGG pathways using Pathview version 1.10.1 (60). Pathways were considered significant if the GAGE q-value was <0.05. ECM-receptor interaction pathway genes (Fig. 6) are based on KEGG pathway mmu04512. We tested the effect of sequencing run and lane on the clustering of the samples and found both to be insignificant (*P* value > 0.05) for PC1 and PC2 in Fig. 5.

### Statistical analysis.

*P* values were calculated using the nonparametric Mann-Whitney test when 2 experimental groups were compared or Kruskal-Wallis test with Dunn’s posttest when ≥3 experimental groups were compared. Data from quantitative bacterial cultures were log transformed for normalization. *P* values of <0.05 were considered significant.

Additional methods are described in the supplemental material.

## Supplementary Material

Supplemental file 1
