# Supplementary material for: Yersiniabactin-Producing Adherent/Invasive Escherichia coli Promotes Inflammation-Associated Fibrosis in Gnotobiotic Il10−/− Mice
Source: Infect Immun. 2019 Oct 18;87(11):e00587-19. doi: 10.1128/IAI.00587-19 (PMC6803345; doi:10.1128/IAI.00587-19)
Supplement: Supplemental file 1 [file IAI.00587-19-s0001.pdf]

Supplementary materials and methods

Bacteria growth conditions.

Bacteria were grown at 250 rpm at 37°C in Luria broth (LB) unless otherwise indicated. For growth under experimental conditions, bacteria were grown in M9 minimal medium, LB, or Minimal Essential Medium (Gibco) with 10% heat-inactivated fetal bovine serum (Gibco). The iron chelator 2,2'-bipyridyl (BPD) or the iron/zinc chelator diethylenetriaminepentaacetic acid (DTPA) was added to the cultures where indicated. Media was supplemented with 50 μg/mL kanamycin or 100 μg/mL carbenicillin as appropriate.

Construction of isogenic mutants, chromosomally and functionally complemented strains and green fluorescent protein (*gfp*) reporters.

Isogenic deletion mutants were created using the *λ-red* recombinase system and resolved using the pCP20 plasmid as previously described (1) (2). Deletion mutants were chromosomally complemented using the pMCL2868 plasmid (kind gift from M. Chelsea Lane), a mini-Tn7 vector, as previously described (3). To functionally complement the *fyuA* isogenic mutant, we used the *λ-red* recombinase system to generate the *irp1* isogenic mutant in the *fyuA*-deficient background, thus abrograting Ybt production. Any off-target effects/mutations in the *fyuA* mutant would therefore be carried over to the *fyuAirp1* double mutant. Transcriptional *gfp* reporters for *tonB*, *cusC* or *znuA* were purchased from the *E. coli* promoter collection from Dharmacon. Reporter

plasmids were transformed into the indicated strains by electroporation. Transformed strains were grown with 50 μg/mL kanamycin to maintain the plasmid.

RNA isolation from colon tissues, cecal contents or bacterial *in vitro* cultures.

RNA was isolated from colon tissues, cecal contents or bacterial cultures using the Qiagen RNAeasy kit per the manufacturers' instructions. Bacterial RNA was harvested using Qiagen RNA Protect per the manufacturer's instructions. RNA isolated from contents was subjected to two different DNase treatments - TurboDNase (Ambion) and Baseline-Zero DNase (Epicenter) – prior to cDNA synthesis. For all samples, superscript II reverse transcriptase (Invitrogen) was utilized to synthesize cDNA from RNA templates per the manufacturers' instructions.

Quantitative real-time PCR.

Targeted qRT-PCR reactions were run using the Sensifast SYBR No-ROX Kit.(Bioline) with the following conditions: 95°C for 2 minutes, followed by 40 cycles at 95°C for 5 seconds, 60°C for 10 seconds and 72°C for 20 seconds. Melting curves were assessed to ensure specificity of the PCR products. Oligonucleotides are listed in Table S7.

Adhesion/invasion assays.

Epithelial adhesion and invasion assays with AIEC were performed as previously described (8). Briefly, Caco2 cells were seeded in 24-well plates and grown to confluency. Bacteria were pretreated with 250 μg/mL BPD or the appropriate vehicle until cultures reached an OD600 of 1.0. Bacteria were washed and then added to the

Caco2 monolayers at a multiplicity of infection (MOI) of 15. Following 3 hours of co-culture, gentamicin-laden medium (MEM with 1% sodium pyruvate, 1% non-essential amino acids and 100 μg/mL gentamicin) was added to eliminate extracellular bacteria to assess bacterial invasion. After 1 hour, Caco2 cells were lysed with 1% Triton X-100 and bacteria were quantified via serial dilution plating. To assess total bacterial association (adhesion + invasion) with Caco2 cells, co-cultures were washed with PBS and then Caco2 cells were immediately lysed without prior incubation with gentamicin.

Isolation of bone marrow derived macrophages.

Bone marrow cells were isolated as previously described (4). Conditioned medium from the murine fibroblast cell line L929 served as a source of M-CSF for macrophage differentiation (5). During all experiments unless otherwise indicated, bone marrow-derived macrophages were maintained in RPMI 1640 medium (Gibco) with 10% heat-inactivated fetal bovine serum (Gibco) and 1% penicillin/streptomycin/antimycotic (Gibco) at 37°C, 5% CO_2.

Macrophage survival assays.

Gentamicin protection assays were performed as previous described (6). Briefly, bone marrow-derived macrophages were seeded in 24-well plates and bacteria were added at a MOI of 10. Following 1-hour incubation, gentamicin-laden medium (RPMI 1640 with 100 μg/mL gentamicin) was added to eliminate extracellular bacteria. Macrophages were lysed with 1% Triton X-100 at the indicated time points to release surviving intracellular bacteria and to enable quantification via serial dilution plating.

Quantification of Ybt.

Culturing. Bacteria were grown in 50mL of culture media (M9 + 0.2% casamino acids + 0.4% glycerol, buffered at pH 6 with 0.1M MES and supplemented with 1mM salicylate) at 37°C in a 125mL flask, with shaking at 200rpm, and protected from light. After 24 hrs, the OD_{600} and pH of cultures were evaluated. Cultures were centrifuged and supernatants were filtered through a 0.2μM-pore-sized filter. Supernatants were stored at -20°C until processing.

Instrumentation. Filtered culture supernatants were thawed at 4°C and acidified (pH 2-3) with trifluoroacetic acid (TFA). Supernatants were passed over a C18 reversed phase column via gravity flow and eluted in 1.25mL of 80% acetonitrile (ACN) + 0.1% TFA. Samples were protected from light and stored at -20°C until analysis via Waters Acquity H-class ultra-performance liquid chromatography with in-line mass spectrometry (UPLC-MS). Samples (3μL) were injected onto a Waters Acquity UPLC BEH C18 column (2.1 x 50 mm, 1.7 μm particle size). Gradient elution conditions were set at 100 % water with 0.1% formic acid (A), ramped linearly over 9.8 mins to 95% acetonitrile with 0.1% formic acid (B), and held until 10.2 mins. At 10.21 mins the gradient was switched back to 100% A and allowed to re-equilibrate until 11.25 mins. UPLC effluent was directed to the ThermoScientific Q Exactive HF-X mass spectrometer through a heated electrospray source (HESI) at a flow rate of 0.6 mL/min. Electrospray source conditions

were set as: spray voltage 3.0 kV, sheath gas (nitrogen) 60 arb, auxillary gas (nitrogen) 20 arb, sweep gas (nitrogen) 0 arb, nebulizer temperature 375 degrees C, capillary temperature 380 degrees C, RF funnel 45 V. The mass range was set to 150-2000 m/z. All measurements were recorded at a resolution setting of 120,000.

Analysis. A 5pt standard curve was generated using 0.1µg/mL – 1mg/mL of commercially available Ybt (YER, *EMC microcollections*). Using XCalibur data acquisition and processing software, extracted ion chromatograms were binned by mass 481-483, with apo-Ybt $[M+H]^+$ detected at 482.12 *m/z*. Then, user-defined integration components were applied to peaks and used to determine response peak area for each sample. Response peak area for component 1 (RT 5.68min +/- 30s) and component 2 (RT 6.00min +/- 30s) were added together and µg/mL Ybt was determined based on the integration of the standard curve. Average µg/mL Ybt and standard deviation were calculated for each sample elute. Proper integration was manually checked for all samples and all LC-MS runs included an external Ybt control, to ensure there was no shift in spectra across days. Samples included two technical replicates per two biological replicates for each strain.

Detection of metal-bound Ybt

Cultures were grown and processed via LC-MS, as described above. For analysis, extracted ion chromatograms were binned by mass as follows: 534-536 (Fe^{3+}-Ybt $[2H]^+$ = 535.03 *m/z*), 543-545 (Zn^{2+}-Ybt $[H]^+$ = 544.04 *m/z*), and 542-544 (Cu^{2+}-Ybt $[H]^+$ =

543.04 *m/z*). Peak detection was performed via visual inspection. No quantification was performed.

Ybt crossfeeding assay.

Bacteria were aerobically grown in LB with 200 μM BPD for 6 hours at 37°C. Bacterial cells were then washed and equilibrated to similar OD600 values. Lawns of the siderophore biosynthesis mutant, *Klebsiella pneumoniae* Δ*entB ybtS*, were plated onto LB agar plates containing 150 μM BPD – a concentration of iron chelator that does not support growth of this mutant. Then, 5-μL inocula of the siderophore feeder strains (NC101 WT, Δ*irp1*, Δ*fyuA*, Δ*fyuA* + *fyuA* or the positive control *K. pneumonia* WT) were applied onto the *K. pneumoniae* Δ*entB ybtS* lawns. Halos of *K. pneumoniae* Δ*entB ybtS* growth surrounding the inocula were assessed after 24-48 hours by measuring the total diameter of growth and subtracting the diameter of the feeder strain inocula/growth.

Biofilm assays.

AIEC biofilm formation in LB medium on polystyrene surfaces (Costar #3370) was assessed using the crystal violet biofilm staining assay as previously described (7).

Bacteria stimulation of fibroblasts.

Swiss 3T3 fibroblast cells were maintained in Dulbecco's Modified Eagle Medium (DMEM) supplemented with 10% FBS and 1% penicillin-streptomycin and passaged at ~80% confluency. Six-well plates were seeded with Swiss 3T3 cells at 3.0 x 10^5 cells/well. Cells were serum starved for 24 hours and then stimulated with the indicated

AIEC strains (MOI 100) for 24- 48 hours. Bacteria were killed after 4 hours by replacing wells with serum starved medium supplemented with 200 μg/mL gentamycin. Cells were stimulated with 50 ng/μL TGF-β as a positive control. At the indicated time points, cells were harvested for RNA isolation using TriZol reagent.

Transcriptional *gfp* reporter fusions

Transformed bacteria harboring the *gfp* reporter plasmids were aerobically grown at 37°C in a 96-well format in M9 minimal medium with 50 μg/mL kanamycin for 3-5 hours. Where indicated, the following was also added: 50 μM DTPA metal chelator, 50 μM Fe^{2+}, 50 μM Zn^{2+} or 50 μM Cu^{2+}. Bacteria transformed with promoterless *gfp* reporters served as negative controls. Fluorescence was normalized to bacterial growth as measured by spectrophotometry (OD600).

Supplemental References

1. **Datsenko KA**, **Wanner BL**. 2000. One-step inactivation of chromosomal genes in Escherichia coli K-12 using PCR products. Proc Natl Acad Sci USA **97**:6640–6645.

2. **Baba T**, **Ara T**, **Hasegawa M**, **Takai Y**, **Okumura Y**, **Baba M**, **Datsenko KA**, **Tomita M**, **Wanner BL**, **Mori H**. 2006. Construction of Escherichia coli K-12 in-frame, single-gene knockout mutants: the Keio collection. Mol Syst Biol **2**:473–11.

3. **Choi K-H**, **Gaynor JB**, **White KG**, **Lopez C**, **Bosio CM**, **Karkhoff-Schweizer RR**, **Schweizer HP**. 2005. A Tn7-based broad-range bacterial cloning and expression system. Nat Meth **2**:443–448.

4. **Lutz MB**, **Kukutsch N**, **Ogilvie AL**, **Rössner S**, **Koch F**, **Romani N**, **Schuler G**. 1999. An advanced culture method for generating large quantities of highly pure dendritic cells from mouse bone marrow. J Immunol Methods **223**:77–92.

5. **Stanley ER**, **Heard PM**. 1977. Factors regulating macrophage production and growth. Purification and some properties of the colony stimulating factor from medium conditioned by mouse L cells. J Biol Chem **252**:4305–4312.

6. **Ellermann M**, **Huh EY**, **Liu B**, **Carroll IM**, **Tamayo R**, **Sartor RB**. 2015. Adherent-Invasive Escherichia coli Production of Cellulose Influences Iron-Induced Bacterial Aggregation, Phagocytosis, and Induction of Colitis. Infect Immun **83**:4068–4080.

7. **Martinez-Medina M**, **Naves P**, **Blanco J**, **Aldeguer X**, **Blanco JE**, **Blanco M**, **Ponte C**, **Soriano F**, **Darfeuille-Michaud A**, **Garcia-Gil LJ**. 2009. Biofilm formation as a novel phenotypic feature of adherent-invasive Escherichia coli (AIEC). BMC Microbiol **9**:202.

8. **Dogan B**, **Suzuki H**, **Herlekar D**, **Sartor RB, Campbell BJ**, **Roberts CL**, **Stewart K**, **Scherl EJ**, **Araz Y**, **Bitar PP**, **Lefebure** T, **Chandler B**, **Schukken YH**, **Stanhope MJ**, **Simpson KW**. 2014. Inflammation-associated adherent-invasive Escherichia coli are enriched in pathways for use of propanediol and iron and M-cell translocation. Inflamm Bowel Dis **20**:1919-1932.

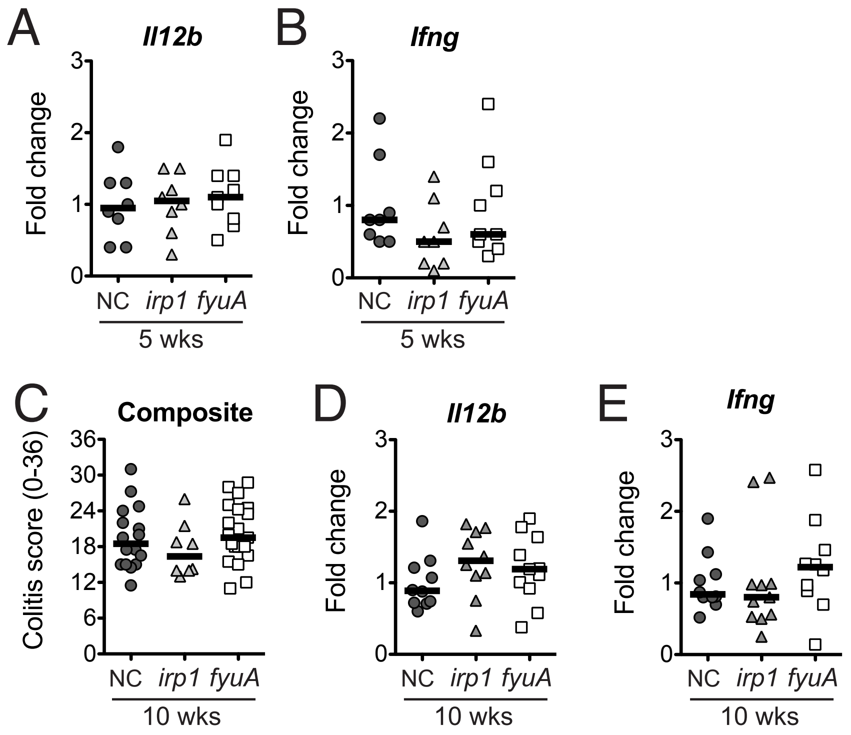

Figure S1. Related to Figure 1. Yersiniabactin-mediated acceleration of colitis development in *Il10*$^{-/-}$ mice is no longer apparent by 10 weeks.

Germ free *Il10*$^{-/-}$ mice were mono-associated with *E. coli* NC101 (NC), Δ*fyuA* or Δ*irp1* for A-B) 5 weeks or C-D) 10 weeks. A-B) Relative colonic transcript levels of A) *Il12b* or B) *Ifng* at 5 weeks. C) Composite regional histopathology colitis scores at 10 weeks. D-E) Relative colonic transcript levels of D) *Il12b* or E) *Ifng* at 10 weeks. Lines are at the median. *P*-values were determined by Kruskal-Wallis.

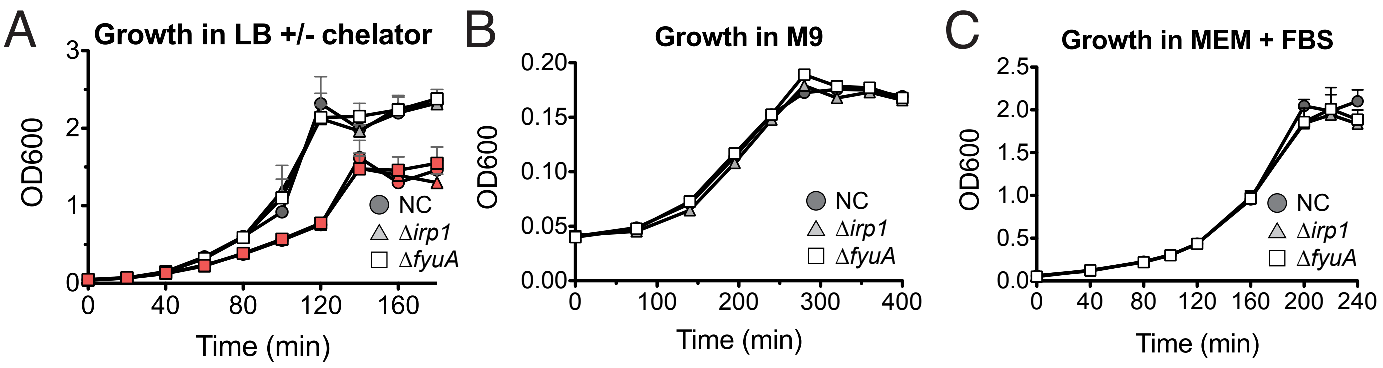

Figure S2. Related to Figure 1. Inactivation of Ybt biosynthesis or transport does not alter fitness under iron sufficient and iron limiting conditions *in vitro*.

In vitro growth curves of *E. coli* NC101 (NC), Δ*fyuA* or Δ*irp1* in A) LB medium (white, grey or black) or in LB medium with the iron chelator BPD (in red), B) minimal M9 medium or C) MEM medium with 10% FBS. Data are represented as the mean ± SEM of at least three independent experiments.

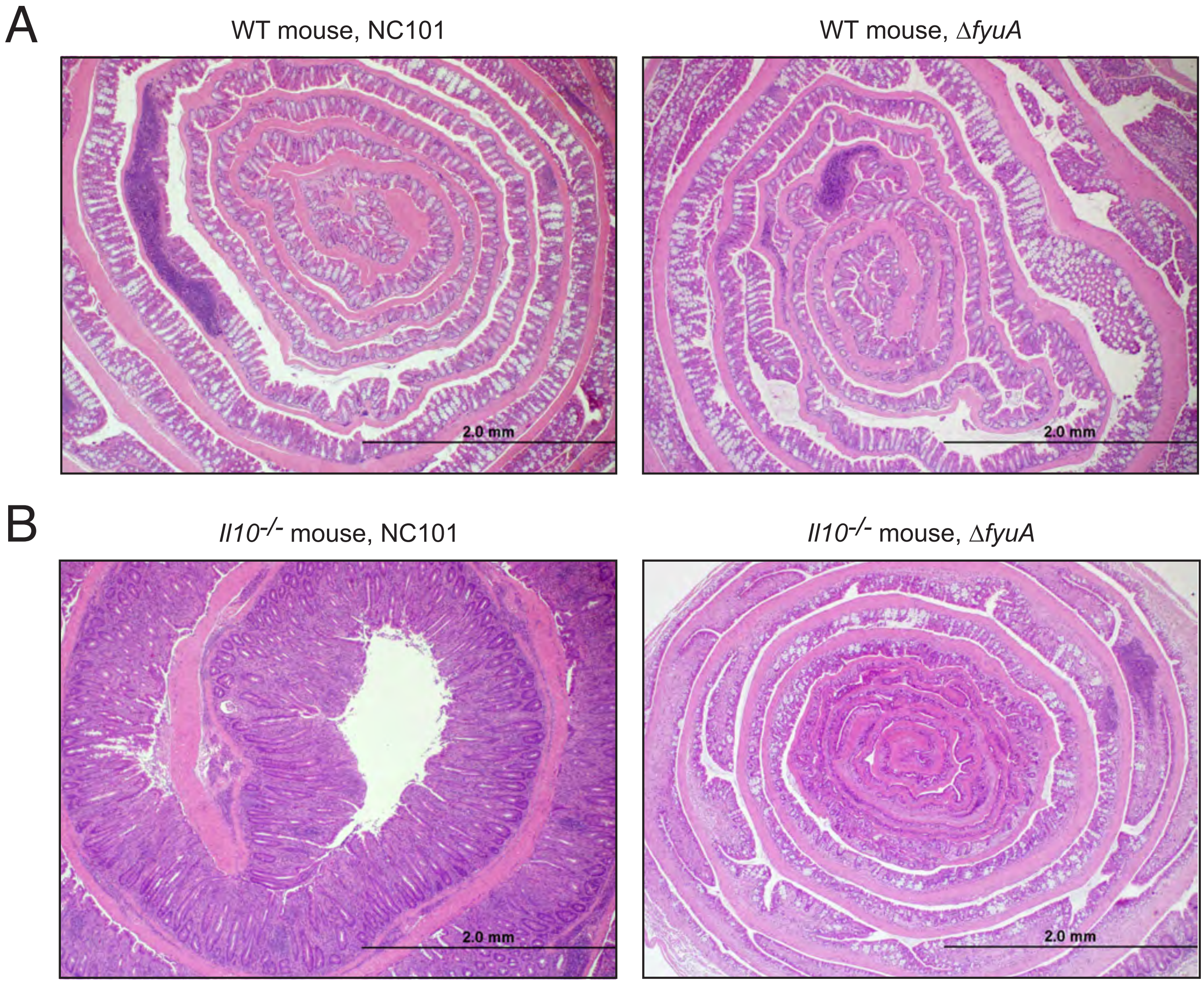

Figure S3. Related to Figure 2. Fibrosis histopathology is more severe and widespread in the colons of *Il10*$^{-/-}$ mice colonized with *fyuA*-deficient AIEC.

Representative histology of Swiss rolled colons from A) WT mice or B) *Il10*$^{-/-}$ mice mono-associated with *E. coli* NC101 or Δ*fyuA* for 10 weeks. White arrowheads indicate fibrotic lesions. Scale bar, 2 mm.

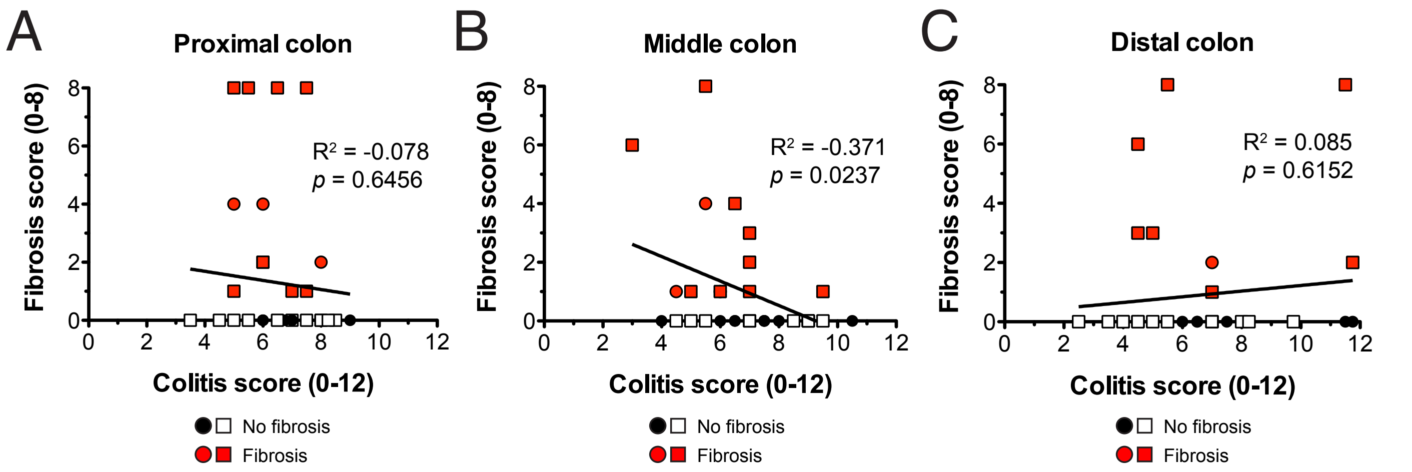

Figure S4. Related to Figure 2. Fibrosis development in *Il10*$^{-/-}$ mice colonized with AIEC does not correlate with colitis severity.

Germ free *Il10*$^{-/-}$ mice were monoassociated with *E. coli* NC101 (NC) (circles) or Δ*fyuA* (squares) for 10 weeks. Correlations of fibrosis versus colitis histopathology scores were generated for the A) proximal, B) middle and C) distal colon.

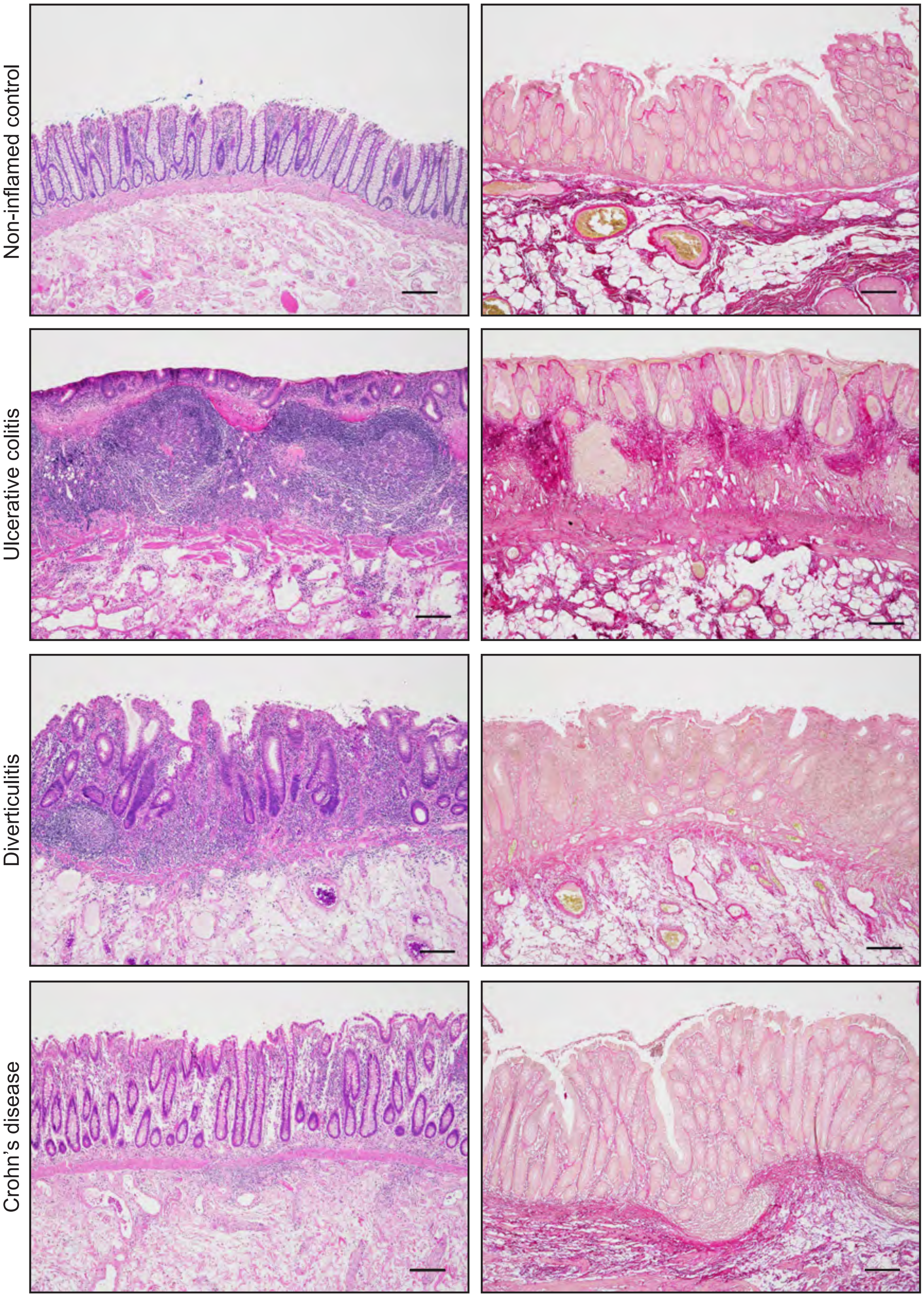

Figure S5. Related to Figure 3. Histology images of colonic full thickness tissue sections from human IBD patients or non-inflamed controls.

Histology of colonic full thickness tissue sections from ulcerative colitis, diverticulitis patients, fibrotic Crohn's disease patients or non-inflamed controls (healthy margins of colorectal cancer patients). Colon sections were stained with H&E or Sirius red. Scale bar, 200 μm.

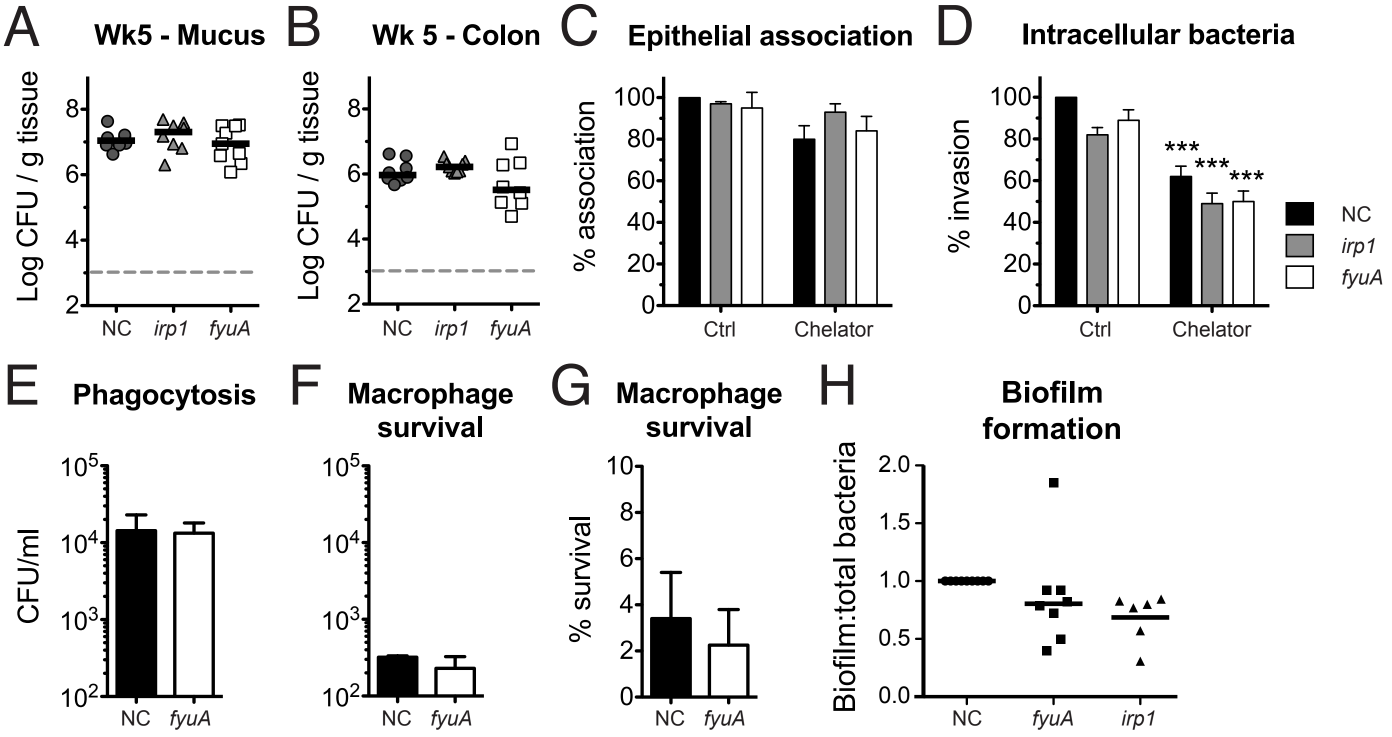

Figure S6. Related to Figure 4. Impact of the yersiniabactin pathogenicity island on AIEC intestinal localization.

Germ free *Il10*$^{-/-}$ mice were mono-associated with the AIEC strain *E. coli* NC101 (NC), Δ*fyuA* or Δ*irp1* for 5 weeks. Quantitative bacteria culture of A) colonic mucus or B) colonic tissues. Each symbol represents an individual mouse. Lines are at the median. *P*-values were determined by Kruskal-Wallis. C-D) Caco-2 cells were co-cultured NC, Δ*fyuA* or Δ*irp1* ± the iron chelator BPD. The percentage of C) epithelial associated and D) intracellular bacteria was determined by quantitative bacteria culture. Data are represented as the mean ± SEM from 4 independent experiments. *P*-values were determined by one-way ANOVA. E-G) Bone marrow derived macrophages from WT mice were co-cultured with NC or Δ*fyuA*. Intracellular bacteria at E) 1 hr and F) 4 hr post infection was determined by quantitative bacteria culture. G) The ratio of intracellular bacteria between 4 hr and 1 hr. Data are represented as the mean ± SEM from 2 independent experiments. H) NC strains were grown statically in LB for 24 hours in 96-well plates. Biofilm formation was assessed as a ratio of the OD570 (biofilm cells) to the OD600 (total bacteria). Data are represented as the mean ± SEM from 2 independent experiments with 16 technical replicates per experiment. *** $p < 0.001$.

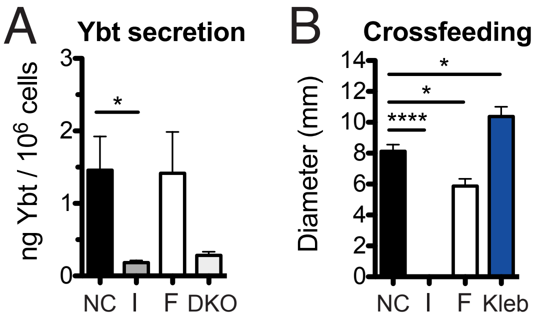

Figure S7. Related to Figure 4. Yersiniabactin secretion and functionality in AIEC.
A) NC, Δ*fyuA*, Δ*irp1* or Δ*fyuA irp1* were cultured in M9 minimal medium. Supernatants were collected for quantification of Ybt by LC-MS. B) NC, Δ*fyuA*, Δ*irp1* or *Klebsiella pneumoniae* were spotted onto a lawn of the *K. pneumoniae* enterobactin (Ent) and Ybt deficient mutant on iron limiting media (LB + BPD). Diameters of *K. pneumoniae* Ybt^- Ent^- growth were measured. Data are represented as the mean ± SEM of three independent experiments. *P*-values were determined by one-way ANOVA. * $p < 0.05$, **** $p < 0.0001$

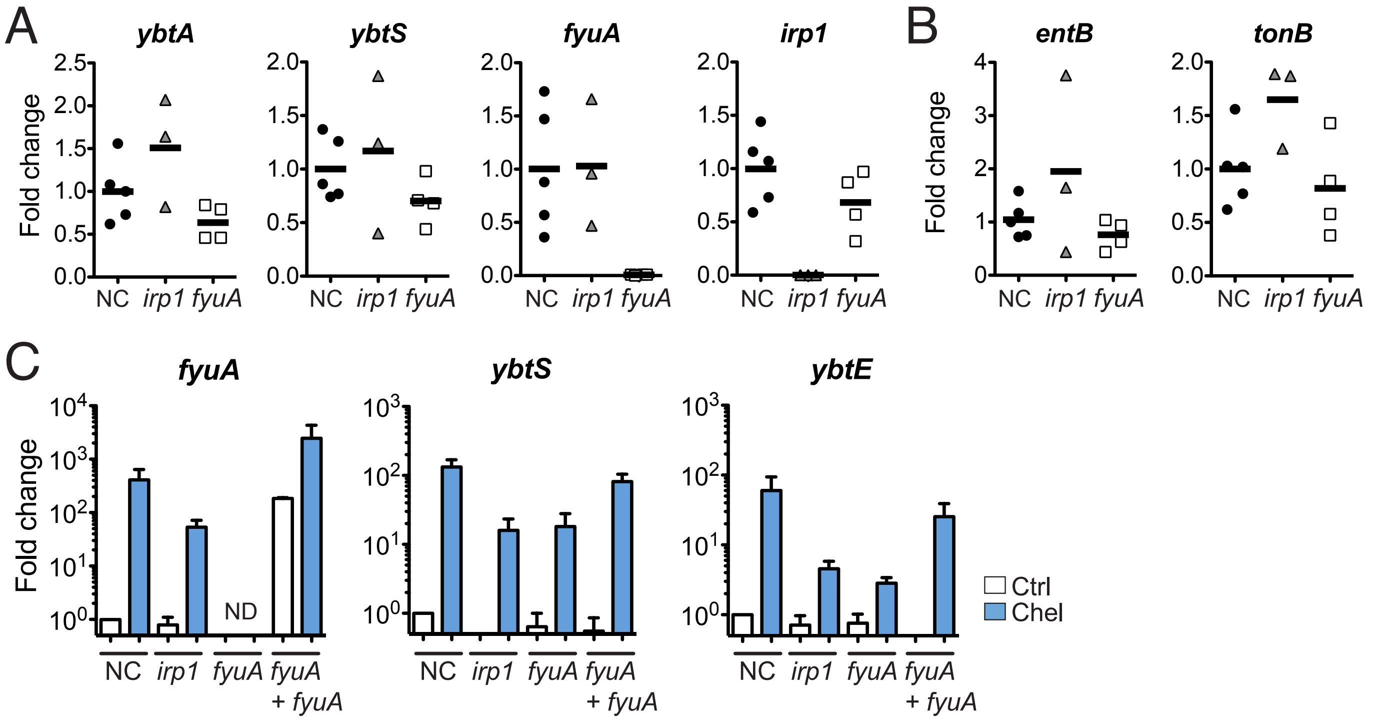

Figure S8. Related to Figure 4. Impact of the yersiniabactin pathogenicity island on iron responsive genes.

A-B) Germ free WT mice were mono-associated with the NC, Δ*fyuA* or Δ*irp1*. A) Transcript levels of A) Ybt-encoded genes *ybtA*, *ybtS*, *fyuA* and *irp1* and B) iron responsive genes *entB* and *tonB* in bacteria recovered from colon contents. C) NC, Δ*irp1,* Δ*fyuA* or Δ*fyuA + fyuA* were cultured in LB medium (Ctrl) ± the iron chelator BPD (Chel). Transcript levels of Ybt-encoded genes *fyuA*, *ybtS* and *ybtE* were assessed. Data are represented as the mean ± SEM of 2-3 independent experiments. *P*-values were determined by one-way ANOVA.

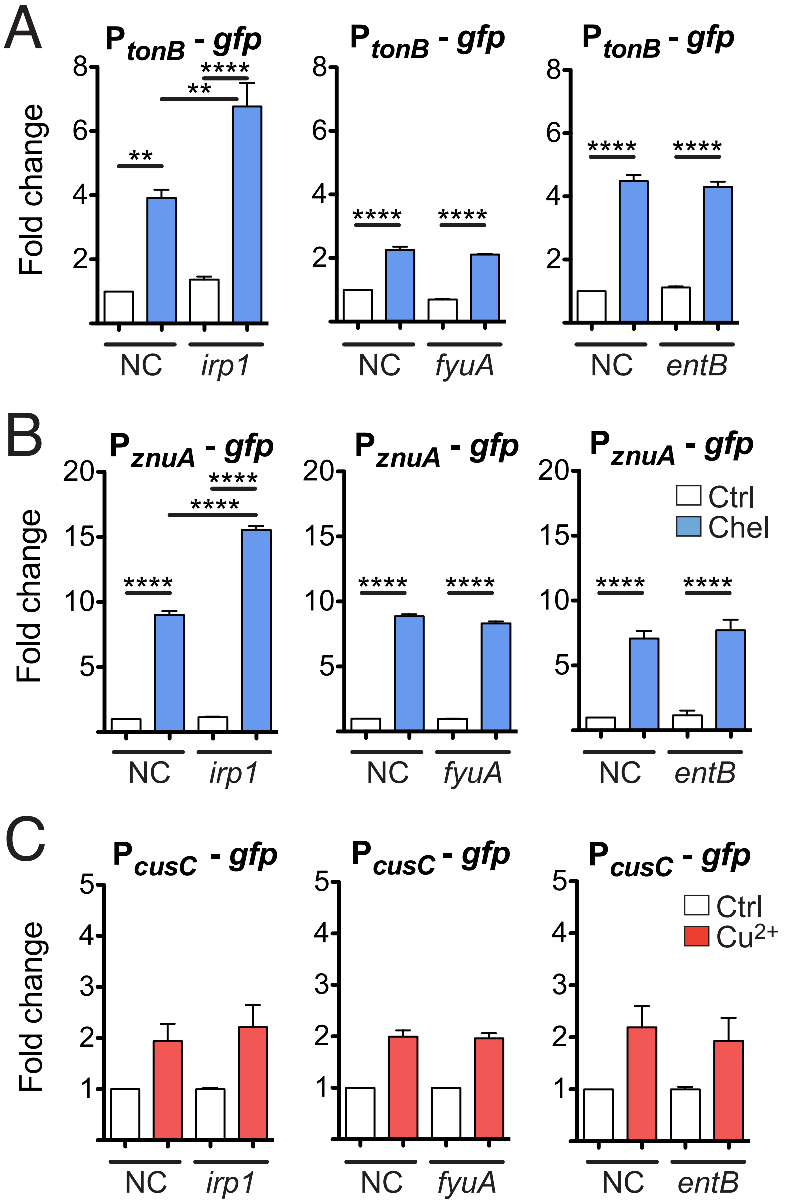

Figure S9. Related to Figure 4. Impact of the yersiniabactin pathogenicity island on AIEC metal sensing.

NC, Δ*fyuA*, Δ*irp1* or Δ*entB* were cultured in M9 minimal medium (Ctrl) with the iron chelator DTPA (Chel) or with added copper (Cu^{2+}). Strains were transformed with a plasmid containing *gfp* under regulation of the A) iron responsive *tonB* promoter, B) zinc responsive *znuA* promoter and C) copper responsive *cusC* promoter. Data are represented as fold change of arbitrary fluorescence units from three independent experiments. *P*-values were determined by one-way ANOVA. ** $p < 0.01$, **** $p < 0.0001$.

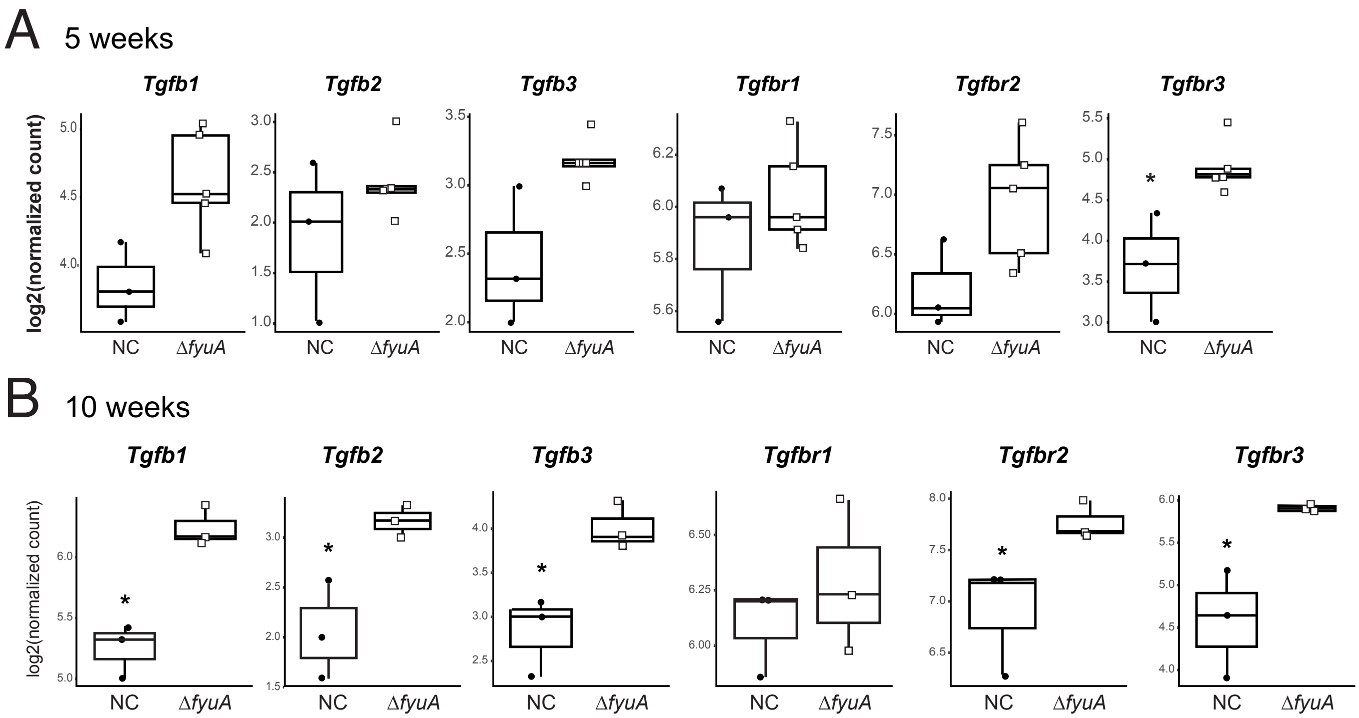

Figure S10. Related to Figure 6. RNAseq reveals enhanced potential for TGF-β signaling in Δ*fyuA*-colonized *Il10$^{-/-}$* mice.

Log2 normalized counts of *Tgfb1*, *Tgfb2*, *Tgfb3*, *Tgfbr1*, *Tgfbr2* and *Tgfbr3* in Δ*fyuA* versus NC-colonized *Il10$^{-/-}$* mice at A) 5 weeks and B) 10 weeks. * FDR-corrected $p < 0.05$. Data from RNAseq analysis.

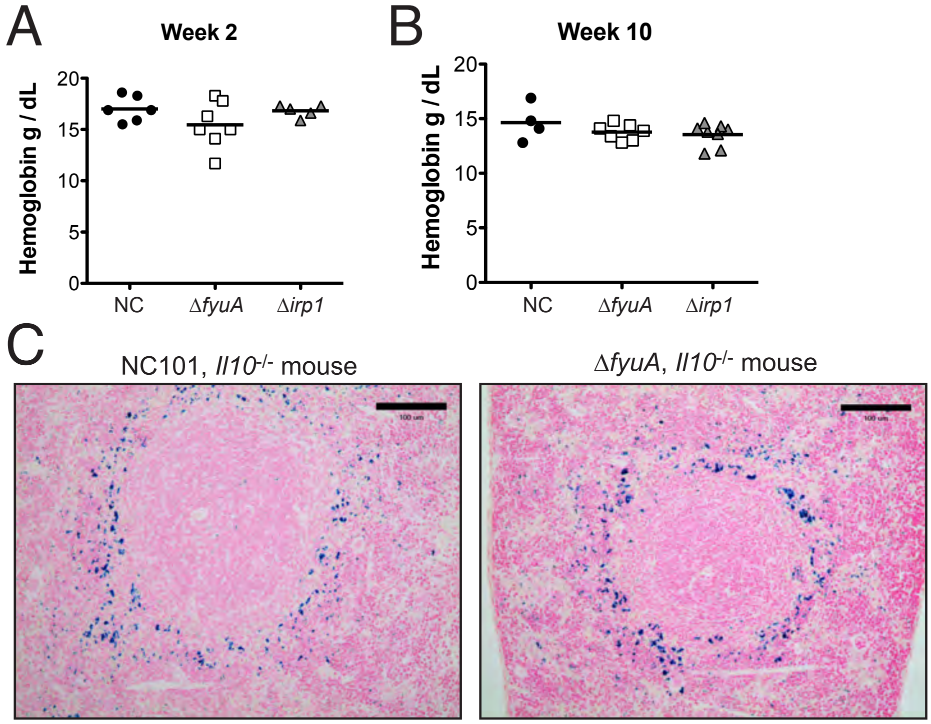

Figure S11. Related to Figure 7. Fibrosis development in Δ*fyuA*-colonized *Il10*$^{-/-}$ mice is not associated with altered bacterial or host systemic iron homeostasis.

A-B) Plasma hemoglobin levels in *Il10*$^{-/-}$ mice mono-associated with NC, Δ*fyuA* or Δ*irp1* for A) 2 weeks or B) 10 weeks. Each symbol represents an individual mouse. Lines are at the median. *P*-values were determined by Kruskal-Wallis. C) Representative histology of spleens from *Il10*$^{-/-}$ mice mono-associated with NC or Δ*fyuA* for 10 weeks. Splenic sections were stained with Prussian blue to visualize iron stores and counter stained with nuclear fast red. Scale bar, 100 μm.

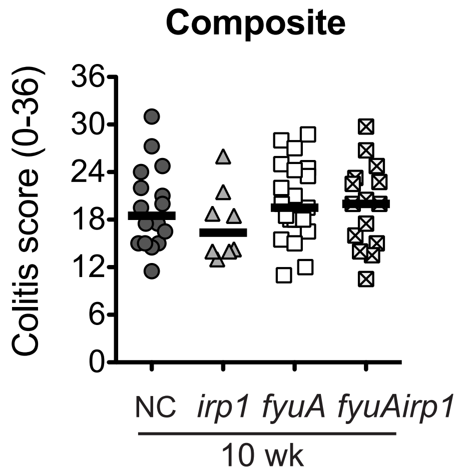

Figure S12. Related to Figure 7. Impact of the yersiniabactin pathogenicity island on AIEC colitis induction.

Composite histopathology colitis scores at 10 weeks post-colonization of germ free *Il10*$^{-/-}$ mice mono-associated with NC, Δ*fyuA*, Δ*irp1* or Δ*fyuA irp1*. Each symbol represents an individual mouse (*n* = 8-21). Lines are at the median. *P*-values were determined by Kruskal-Wallis. Data shown for NC, Δ*fyuA* and Δ*irp1* were also presented in Figure 1.

Table S1: List of differentially expressed genes between colon tissue of Il10-/- mice colonized with either NC101 or △fyuA for 5 weeks

LogFC values are positive if NC101> fyuA

Gene	logFC	FDR	Description
Ighv4-1	3.59	1.9E-05	immunoglobulin heavy variable 4-1 [Source:MGI Symbol;Acc:MGI:4439536]
Cps1	2.35	2.1E-02	carbamoyl-phosphate synthetase 1 [Source:MGI Symbol;Acc:MGI:891996]
Muc16	1.92	1.6E-04	mucin 16 [Source:MGI Symbol;Acc:MGI:1920982]
Krt27	1.77	2.4E-03	keratin 27 [Source:MGI Symbol;Acc:MGI:1339999]
Reg4	1.51	1.7E-06	regenerating islet-derived family, member 4 [Source:MGI Symbol;Acc:MGI:1914959]
St3gal5	1.48	1.2E-04	ST3 beta-galactoside alpha-2,3-sialyltransferase 5 [Source:MGI Symbol;Acc:MGI:1339963]
Adcy8	1.42	4.0E-02	adenylate cyclase 8 [Source:MGI Symbol;Acc:MGI:1341110]
Gsdmc4	1.35	1.5E-04	gasdermin C4 [Source:MGI Symbol;Acc:MGI:1921798]
Gsdmc2	1.30	5.6E-03	gasdermin C2 [Source:MGI Symbol;Acc:MGI:2146102]
Gsdmc	1.28	2.6E-02	gasdermin C [Source:MGI Symbol;Acc:MGI:1933176]
Atp12a	1.27	1.9E-02	ATPase, H+/K+ transporting, nongastric, alpha polypeptide [Source:MGI Symbol;Acc:MGI:1926943]
Slc16a9	1.23	2.1E-03	solute carrier family 16 (monocarboxylic acid transporters), member 9 [Source:MGI Symbol;Acc:MGI:1914109]
Rnf152	1.23	2.2E-02	ring finger protein 152 [Source:MGI Symbol;Acc:MGI:2443787]
Trpv3	1.22	3.5E-02	transient receptor potential cation channel, subfamily V, member 3 [Source:MGI Symbol;Acc:MGI:2181407]
Cmtm8	1.21	3.2E-02	CKLF-like MARVEL transmembrane domain containing 8 [Source:MGI Symbol;Acc:MGI:2447167]
Slc20a1	1.17	6.4E-05	solute carrier family 20, member 1 [Source:MGI Symbol;Acc:MGI:108392]
9830144P21Rik	1.14	1.8E-02	RIKEN cDNA 9830144P21 gene [Source:MGI Symbol;Acc:MGI:3041171]
Cyp2c55	1.14	2.4E-02	cytochrome P450, family 2, subfamily c, polypeptide 55 [Source:MGI Symbol;Acc:MGI:1919332]
Gsdmc3	1.12	4.1E-02	gasdermin C3 [Source:MGI Symbol;Acc:MGI:3580656]
Spink4	1.07	4.0E-04	serine peptidase inhibitor, Kazal type 4 [Source:MGI Symbol;Acc:MGI:1341848]
Ces1g	1.04	1.6E-02	carboxylesterase 1G [Source:MGI Symbol;Acc:MGI:88378]
Cyp2c66	0.88	3.3E-02	cytochrome P450, family 2, subfamily c, polypeptide 66 [Source:MGI Symbol;Acc:MGI:1917138]
Pmp22	0.84	1.2E-03	peripheral myelin protein 22 [Source:MGI Symbol;Acc:MGI:97631]
Ankrd37	0.82	3.3E-02	ankyrin repeat domain 37 [Source:MGI Symbol;Acc:MGI:3603344]
Akr1c19	0.71	1.3E-02	aldo-keto reductase family 1, member C19 [Source:MGI Symbol;Acc:MGI:2653678]
Mettl7a1	0.69	2.6E-02	methyltransferase like 7A1 [Source:MGI Symbol;Acc:MGI:1916523]
Sult1d1	0.69	1.9E-02	sulfotransferase family 1D, member 1 [Source:MGI Symbol;Acc:MGI:1926341]
Slc22a1	0.69	3.6E-02	solute carrier family 22 (organic cation transporter), member 1 [Source:MGI Symbol;Acc:MGI:108111]
Maoa	0.68	1.4E-02	monoamine oxidase A [Source:MGI Symbol;Acc:MGI:96915]
Tff3	0.65	4.2E-02	trefoil factor 3, intestinal [Source:MGI Symbol;Acc:MGI:104638]
Nqo1	0.62	3.8E-02	NAD(P)H dehydrogenase, quinone 1 [Source:MGI Symbol;Acc:MGI:103187]
Emp1	0.61	4.1E-02	epithelial membrane protein 1 [Source:MGI Symbol;Acc:MGI:107941]
Abcg2	0.57	3.0E-02	ATP-binding cassette, sub-family G (WHITE), member 2 [Source:MGI Symbol;Acc:MGI:1347061]
Cd97	-0.62	5.0E-02	CD97 antigen [Source:MGI Symbol;Acc:MGI:1347095]
Gna14	-0.64	1.8E-02	guanine nucleotide binding protein, alpha 14 [Source:MGI Symbol;Acc:MGI:95769]
Lsp1	-0.69	3.3E-02	lymphocyte specific 1 [Source:MGI Symbol;Acc:MGI:96832]
Fry	-0.71	4.7E-02	furry homolog (Drosophila) [Source:MGI Symbol;Acc:MGI:2443895]
Anxa6	-0.74	3.3E-02	annexin A6 [Source:MGI Symbol;Acc:MGI:88255]
Itpkb	-0.74	4.1E-02	inositol 1,4,5-trisphosphate 3-kinase B [Source:MGI Symbol;Acc:MGI:109235]
Tacc1	-0.77	3.4E-02	transforming, acidic coiled-coil containing protein 1 [Source:MGI Symbol;Acc:MGI:2443510]
Mmp14	-0.77	1.8E-02	matrix metallopeptidase 14 (membrane-inserted) [Source:MGI Symbol;Acc:MGI:101900]
Rac2	-0.78	4.0E-02	RAS-related C3 botulinum substrate 2 [Source:MGI Symbol;Acc:MGI:97846]

Ppp1r18	-0.78	3.3E-02	protein phosphatase 1, regulatory subunit 18 [Source:MGI Symbol;Acc:MGI:1923698]
Thy1	-0.79	1.8E-02	thymus cell antigen 1, theta [Source:MGI Symbol;Acc:MGI:98747]
Fxyd5	-0.79	4.0E-02	FXYD domain-containing ion transport regulator 5 [Source:MGI Symbol;Acc:MGI:1201785]
Rcsd1	-0.79	4.0E-02	RCSD domain containing 1 [Source:MGI Symbol;Acc:MGI:2676394]
Itga1	-0.80	4.2E-02	integrin alpha 1 [Source:MGI Symbol;Acc:MGI:96599]
Vim	-0.81	1.4E-02	vimentin [Source:MGI Symbol;Acc:MGI:98932]
Ifi203	-0.81	1.8E-02	interferon activated gene 203 [Source:MGI Symbol;Acc:MGI:96428]
Gpx3	-0.81	2.6E-02	glutathione peroxidase 3 [Source:MGI Symbol;Acc:MGI:105102]
Igfbp7	-0.82	3.8E-02	insulin-like growth factor binding protein 7 [Source:MGI Symbol;Acc:MGI:1352480]
C1qa	-0.82	3.6E-02	complement component 1, q subcomponent, alpha polypeptide [Source:MGI Symbol;Acc:MGI:88223]
B3galt5	-0.83	9.2E-03	UDP-Gal:betaGlcNAc beta 1,3-galactosyltransferase, polypeptide 5 [Source:MGI Symbol;Acc:MGI:2136878]
Fmnl1	-0.83	4.7E-02	formin-like 1 [Source:MGI Symbol;Acc:MGI:1888994]
Mafb	-0.83	1.9E-02	v-maf musculoaponeurotic fibrosarcoma oncogene family, protein B (avian) [Source:MGI Symbol;Acc:MGI:104555]
Apoe	-0.84	3.3E-02	apolipoprotein E [Source:MGI Symbol;Acc:MGI:88057]
Selplg	-0.86	1.1E-02	selectin, platelet (p-selectin) ligand [Source:MGI Symbol;Acc:MGI:106689]
Hspg2	-0.86	2.5E-02	perlecan (heparan sulfate proteoglycan 2) [Source:MGI Symbol;Acc:MGI:96257]
Dkk3	-0.86	3.3E-02	dickkopf homolog 3 (Xenopus laevis) [Source:MGI Symbol;Acc:MGI:1354952]
Rassf2	-0.86	4.8E-02	Ras association (RalGDS/AF-6) domain family member 2 [Source:MGI Symbol;Acc:MGI:2442060]
Msn	-0.86	2.8E-02	moesin [Source:MGI Symbol;Acc:MGI:97167]
Postn	-0.87	3.4E-02	periostin, osteoblast specific factor [Source:MGI Symbol;Acc:MGI:1926321]
Hip1	-0.87	4.1E-02	huntingtin interacting protein 1 [Source:MGI Symbol;Acc:MGI:1099804]
Slc43a2	-0.88	1.4E-02	solute carrier family 43, member 2 [Source:MGI Symbol;Acc:MGI:2442746]
Entpd1	-0.90	3.9E-02	ectonucleoside triphosphate diphosphohydrolase 1 [Source:MGI Symbol;Acc:MGI:102805]
Cygb	-0.90	4.0E-02	cytoglobin [Source:MGI Symbol;Acc:MGI:2149481]
Plxnd1	-0.92	2.5E-02	plexin D1 [Source:MGI Symbol;Acc:MGI:2154244]
Fscn1	-0.93	2.4E-02	fascin homolog 1, actin bundling protein (Strongylocentrotus purpuratus) [Source:MGI Symbol;Acc:MGI:1352745]
Enpp2	-0.96	4.2E-02	ectonucleotide pyrophosphatase/phosphodiesterase 2 [Source:MGI Symbol;Acc:MGI:1321390]
Plekho2	-0.96	3.3E-02	pleckstrin homology domain containing, family O member 2 [Source:MGI Symbol;Acc:MGI:2143132]
Ppap2b	-0.97	4.1E-02	phosphatidic acid phosphatase type 2B [Source:MGI Symbol;Acc:MGI:1915166]
4632428N05Rik	-0.97	1.1E-02	RIKEN cDNA 4632428N05 gene [Source:MGI Symbol;Acc:MGI:1921298]
Ptpn14	-0.97	3.5E-02	protein tyrosine phosphatase, non-receptor type 14 [Source:MGI Symbol;Acc:MGI:102467]
Eng	-0.98	4.0E-02	endoglin [Source:MGI Symbol;Acc:MGI:95392]
Derl3	-0.98	4.7E-02	Der1-like domain family, member 3 [Source:MGI Symbol;Acc:MGI:1917627]
Nrp1	-0.98	4.1E-02	neuropilin 1 [Source:MGI Symbol;Acc:MGI:106206]
Slfn5	-0.98	4.7E-02	schlafen 5 [Source:MGI Symbol;Acc:MGI:1329004]
Slc43a3	-0.99	1.4E-02	solute carrier family 43, member 3 [Source:MGI Symbol;Acc:MGI:1931054]
C1qb	-1.01	6.8E-03	complement component 1, q subcomponent, beta polypeptide [Source:MGI Symbol;Acc:MGI:88224]
Axl	-1.01	1.8E-02	AXL receptor tyrosine kinase [Source:MGI Symbol;Acc:MGI:1347244]
Laptm5	-1.03	1.5E-02	lysosomal-associated protein transmembrane 5 [Source:MGI Symbol;Acc:MGI:108046]
Fcgr2b	-1.04	4.8E-02	Fc receptor, IgG, low affinity IIb [Source:MGI Symbol;Acc:MGI:95499]
Itgb2	-1.04	3.3E-02	integrin beta 2 [Source:MGI Symbol;Acc:MGI:96611]
Aldh1a3	-1.05	4.5E-02	aldehyde dehydrogenase family 1, subfamily A3 [Source:MGI Symbol;Acc:MGI:1861722]
Pltp	-1.08	3.5E-02	phospholipid transfer protein [Source:MGI Symbol;Acc:MGI:103151]
Bgn	-1.08	5.6E-03	biglycan [Source:MGI Symbol;Acc:MGI:88158]
Nid1	-1.08	4.1E-02	nidogen 1 [Source:MGI Symbol;Acc:MGI:97342]
Lamc1	-1.08	4.0E-02	laminin, gamma 1 [Source:MGI Symbol;Acc:MGI:99914]

C1ra	-1.09	1.4E-02	complement component 1, r subcomponent A [Source:MGI Symbol;Acc:MGI:1355313]
Igha	-1.09	3.2E-02	immunoglobulin heavy constant alpha [Source:MGI Symbol;Acc:MGI:96444]
Lama4	-1.09	4.9E-02	laminin, alpha 4 [Source:MGI Symbol;Acc:MGI:109321]
Serping1	-1.09	1.8E-02	serine (or cysteine) peptidase inhibitor, clade G, member 1 [Source:MGI Symbol;Acc:MGI:894696]
Col1a2	-1.10	4.0E-02	collagen, type I, alpha 2 [Source:MGI Symbol;Acc:MGI:88468]
Dock2	-1.12	2.1E-02	dedicator of cyto-kinesis 2 [Source:MGI Symbol;Acc:MGI:2149010]
C1qc	-1.12	1.5E-03	complement component 1, q subcomponent, C chain [Source:MGI Symbol;Acc:MGI:88225]
Itgal	-1.12	3.2E-02	integrin alpha L [Source:MGI Symbol;Acc:MGI:96606]
Fn1	-1.13	1.1E-02	fibronectin 1 [Source:MGI Symbol;Acc:MGI:95566]
Sirpa	-1.13	3.6E-03	signal-regulatory protein alpha [Source:MGI Symbol;Acc:MGI:108563]
Pid1	-1.14	3.5E-02	phosphotyrosine interaction domain containing 1 [Source:MGI Symbol;Acc:MGI:2138391]
Il6ra	-1.14	4.5E-02	interleukin 6 receptor, alpha [Source:MGI Symbol;Acc:MGI:105304]
Cxcl12	-1.15	1.1E-02	chemokine (C-X-C motif) ligand 12 [Source:MGI Symbol;Acc:MGI:103556]
Stab1	-1.15	7.1E-04	stabilin 1 [Source:MGI Symbol;Acc:MGI:2178742]
Sod3	-1.19	5.0E-02	superoxide dismutase 3, extracellular [Source:MGI Symbol;Acc:MGI:103181]
Tgfbr3	-1.19	1.4E-02	transforming growth factor, beta receptor III [Source:MGI Symbol;Acc:MGI:104637]
Scube1	-1.19	1.8E-02	signal peptide, CUB domain, EGF-like 1 [Source:MGI Symbol;Acc:MGI:1890616]
Cmklr1	-1.21	4.6E-02	chemokine-like receptor 1 [Source:MGI Symbol;Acc:MGI:109603]
Gpr65	-1.21	3.6E-02	G-protein coupled receptor 65 [Source:MGI Symbol;Acc:MGI:108031]
Abca1	-1.23	1.3E-02	ATP-binding cassette, sub-family A (ABC1), member 1 [Source:MGI Symbol;Acc:MGI:99607]
Col1a1	-1.23	1.8E-02	collagen, type I, alpha 1 [Source:MGI Symbol;Acc:MGI:88467]
Fbn1	-1.23	4.4E-02	fibrillin 1 [Source:MGI Symbol;Acc:MGI:95489]
Flt4	-1.24	4.0E-02	FMS-like tyrosine kinase 4 [Source:MGI Symbol;Acc:MGI:95561]
Lox	-1.25	3.2E-02	lysyl oxidase [Source:MGI Symbol;Acc:MGI:96817]
Ltbp4	-1.26	2.1E-02	latent transforming growth factor beta binding protein 4 [Source:MGI Symbol;Acc:MGI:1321395]
Csf1r	-1.26	3.6E-03	colony stimulating factor 1 receptor [Source:MGI Symbol;Acc:MGI:1339758]
Cp	-1.27	2.2E-02	ceruloplasmin [Source:MGI Symbol;Acc:MGI:88476]
Mrc1	-1.28	2.3E-02	mannose receptor, C type 1 [Source:MGI Symbol;Acc:MGI:97142]
Cybb	-1.28	1.9E-02	cytochrome b-245, beta polypeptide [Source:MGI Symbol;Acc:MGI:88574]
Itgam	-1.30	3.1E-03	integrin alpha M [Source:MGI Symbol;Acc:MGI:96607]
Slc11a1	-1.30	4.0E-02	solute carrier family 11 (proton-coupled divalent metal ion transporters), member 1 [Source:MGI Symbol;Acc:MGI:1345275]
Igfbp5	-1.30	3.2E-02	insulin-like growth factor binding protein 5 [Source:MGI Symbol;Acc:MGI:96440]
Prelp	-1.31	3.3E-02	proline arginine-rich end leucine-rich repeat [Source:MGI Symbol;Acc:MGI:2151110]
C4b	-1.31	1.5E-02	complement component 4B (Chido blood group) [Source:MGI Symbol;Acc:MGI:88228]
Ptafr	-1.31	4.1E-02	platelet-activating factor receptor [Source:MGI Symbol;Acc:MGI:106066]
Eln	-1.32	1.8E-02	elastin [Source:MGI Symbol;Acc:MGI:95317]
Fmo2	-1.33	4.0E-02	flavin containing monooxygenase 2 [Source:MGI Symbol;Acc:MGI:1916776]
Fbln1	-1.33	2.2E-03	fibulin 1 [Source:MGI Symbol;Acc:MGI:95487]
Adamts2	-1.41	4.1E-02	a disintegrin-like and metallopeptidase (reprolysin type) with thrombospondin type 1 motif, 2 [Source:MGI Symbol;Acc:MGI:1347356]
Htra3	-1.41	2.4E-03	HtrA serine peptidase 3 [Source:MGI Symbol;Acc:MGI:1925808]
Spon1	-1.45	1.9E-02	spondin 1, (f-spondin) extracellular matrix protein [Source:MGI Symbol;Acc:MGI:2385287]
Thbs2	-1.45	2.1E-02	thrombospondin 2 [Source:MGI Symbol;Acc:MGI:98738]
St6galnac5	-1.47	3.3E-02	ST6 (alpha-N-acetyl-neuraminyl-2,3-beta-galactosyl-1,3)-N-acetylgalactosaminide alpha-2,6-sialyltransferase 5 [Source:MGI Symbol;Acc:MGI:1349471]
C1qtnf3	-1.48	2.1E-02	C1q and tumor necrosis factor related protein 3 [Source:MGI Symbol;Acc:MGI:1932136]
Lum	-1.48	4.1E-02	lumican [Source:MGI Symbol;Acc:MGI:109347]
Serpina3n	-1.49	2.1E-03	serine (or cysteine) peptidase inhibitor, clade A, member 3N [Source:MGI Symbol;Acc:MGI:105045]
Aoah	-1.50	4.1E-02	acyloxyacyl hydrolase [Source:MGI Symbol;Acc:MGI:1350928]

Abca9	-1.54	1.2E-02	ATP-binding cassette, sub-family A (ABC1), member 9 [Source:MGI Symbol;Acc:MGI:2386796]
Vcam1	-1.54	7.1E-04	vascular cell adhesion molecule 1 [Source:MGI Symbol;Acc:MGI:98926]
Fndc1	-1.56	3.9E-02	fibronectin type III domain containing 1 [Source:MGI Symbol;Acc:MGI:1915905]
Igkv3-2	-1.58	2.1E-02	immunoglobulin kappa variable 3-2 [Source:MGI Symbol;Acc:MGI:1330850]
Vpreb3	-1.59	3.3E-02	pre-B lymphocyte gene 3 [Source:MGI Symbol;Acc:MGI:98938]
Svep1	-1.60	1.8E-02	sushi, von Willebrand factor type A, EGF and pentraxin domain containing 1 [Source:MGI Symbol;Acc:MGI:1928849]
Serpina3e-ps	-1.61	2.4E-02	serine (or cysteine) peptidase inhibitor, clade A, member 3E, pseudogene [Source:MGI Symbol;Acc:MGI:2182837]
Emr1	-1.67	1.2E-02	EGF-like module containing, mucin-like, hormone receptor-like sequence 1 [Source:MGI Symbol;Acc:MGI:106912]
Mfap4	-1.68	8.8E-05	microfibrillar-associated protein 4 [Source:MGI Symbol;Acc:MGI:1342276]
Lama2	-1.70	2.4E-02	laminin, alpha 2 [Source:MGI Symbol;Acc:MGI:99912]
C3	-1.75	2.2E-03	complement component 3 [Source:MGI Symbol;Acc:MGI:88227]
F10	-1.82	2.7E-02	coagulation factor X [Source:MGI Symbol;Acc:MGI:103107]
Ighv1-26	-1.83	4.2E-02	immunoglobulin heavy variable 1-26 [Source:MGI Symbol;Acc:MGI:4439641]
Col14a1	-1.84	3.6E-02	collagen, type XIV, alpha 1 [Source:MGI Symbol;Acc:MGI:1341272]
Gm14548	-1.88	2.8E-02	predicted gene 14548 [Source:MGI Symbol;Acc:MGI:3709645]
Serpina3m	-1.91	1.4E-02	serine (or cysteine) peptidase inhibitor, clade A, member 3M [Source:MGI Symbol;Acc:MGI:98378]
Ighg2c	-1.94	3.2E-02	immunoglobulin heavy constant gamma 2C [Source:MGI Symbol;Acc:MGI:2686979]
Cd300lh	-1.97	3.5E-02	CD300 antigen like family member H [Source:MGI Symbol;Acc:MGI:2687214]
Chil1	-2.07	1.2E-02	chitinase-like 1 [Source:MGI Symbol;Acc:MGI:1340899]
Igkv4-68	-2.20	3.3E-02	immunoglobulin kappa variable 4-68 [Source:MGI Symbol;Acc:MGI:2686265]
Ighv10-1	-2.22	4.8E-02	immunoglobulin heavy variable 10-1 [Source:MGI Symbol;Acc:MGI:4439620]
Fcrls	-2.23	5.0E-02	Fc receptor-like S, scavenger receptor [Source:MGI Symbol;Acc:MGI:1933397]
Slit3	-2.30	4.0E-02	slit homolog 3 (Drosophila) [Source:MGI Symbol;Acc:MGI:1315202]
Ighg1	-2.32	1.8E-02	immunoglobulin heavy constant gamma 1 (G1m marker) [Source:MGI Symbol;Acc:MGI:96446]
Marco	-3.33	1.1E-02	macrophage receptor with collagenous structure [Source:MGI Symbol;Acc:MGI:1309998]
Igkv5-48	-3.39	1.1E-04	immunoglobulin kappa variable 5-48 [Source:MGI Symbol;Acc:MGI:3642817]
Ighv1-9	-3.87	1.5E-03	immunoglobulin heavy variable V1-9 [Source:MGI Symbol;Acc:MGI:4439621]
Iglc2	-4.37	1.7E-06	immunoglobulin lambda constant 2 [Source:MGI Symbol;Acc:MGI:99547]
Ighv1-54	-4.64	1.6E-04	immunoglobulin heavy variable V1-54 [Source:MGI Symbol;Acc:MGI:3647133]
Ighv1-12	-4.84	5.7E-11	immunoglobulin heavy variable V1-12 [Source:MGI Symbol;Acc:MGI:3646284]
Ighv3-8	-4.93	7.1E-10	immunoglobulin heavy variable V3-8 [Source:MGI Symbol;Acc:MGI:3645298]
#N/A	-5.66	2.8E-10	#N/A
Iglv2	-6.21	2.8E-06	immunoglobulin lambda variable 2 [Source:MGI Symbol;Acc:MGI:99548]

Table S2: List of differentially expressed KEGG pathways between colon tissue of Il10-/- mice colonized with either NC101 or △fyuA for 5 weeks

KEGG Pathways increased in fyuA v NC	Magnitude of perturbation	FDR
mmu04512 ECM-receptor interaction	4.46	4.3E-19
mmu04510 Focal adhesion	4.29	4.3E-19
mmu04151 PI3K-Akt signaling pathway	3.78	2.5E-15
mmu04060 Cytokine-cytokine receptor interaction	3.81	2.5E-15
mmu04514 Cell adhesion molecules (CAMs)	3.69	1.4E-14
mmu04610 Complement and coagulation cascades	3.49	2.7E-12
mmu04015 Rap1 signaling pathway	3.13	7.5E-11
mmu04640 Hematopoietic cell lineage	2.98	1.3E-09
mmu04080 Neuroactive ligand-receptor interaction	2.95	1.3E-09
mmu04974 Protein digestion and absorption	3.03	1.3E-09
mmu04360 Axon guidance	2.89	1.5E-09
mmu04062 Chemokine signaling pathway	2.71	2.0E-08
mmu04670 Leukocyte transendothelial migration	2.69	2.3E-08
mmu04810 Regulation of actin cytoskeleton	2.66	2.8E-08
mmu04380 Osteoclast differentiation	2.60	8.2E-08
mmu04020 Calcium signaling pathway	2.54	1.2E-07
mmu04024 cAMP signaling pathway	2.48	2.5E-07
mmu04611 Platelet activation	2.44	4.1E-07
mmu04145 Phagosome	2.24	3.9E-06
mmu04022 cGMP-PKG signaling pathway	2.23	4.5E-06
mmu04970 Salivary secretion	2.23	4.8E-06
mmu04014 Ras signaling pathway	2.17	6.7E-06
mmu04713 Circadian entrainment	2.12	1.4E-05
mmu04310 Wnt signaling pathway	1.97	5.6E-05
mmu04270 Vascular smooth muscle contraction	1.97	5.7E-05
mmu04010 MAPK signaling pathway	1.93	6.5E-05
mmu04630 Jak-STAT signaling pathway	1.94	7.3E-05
mmu04672 Intestinal immune network for IgA production	1.94	1.2E-04
mmu04662 B cell receptor signaling pathway	1.89	1.3E-04
mmu04911 Insulin secretion	1.87	1.4E-04
mmu04261 Adrenergic signaling in cardiomyocytes	1.85	1.4E-04
mmu04725 Cholinergic synapse	1.82	1.8E-04
mmu04064 NF-kappa B signaling pathway	1.81	2.4E-04
mmu04650 Natural killer cell mediated cytotoxicity	1.69	6.6E-04
mmu04390 Hippo signaling pathway	1.63	8.6E-04
mmu04660 T cell receptor signaling pathway	1.65	8.6E-04
mmu00601 Glycosphingolipid biosynthesis - lacto and neolacto series	1.64	1.1E-03
mmu04921 Oxytocin signaling pathway	1.60	1.1E-03
mmu04340 Hedgehog signaling pathway	1.53	2.0E-03
mmu04724 Glutamatergic synapse	1.52	2.1E-03
mmu04540 Gap junction	1.49	2.6E-03
mmu04919 Thyroid hormone signaling pathway	1.48	2.6E-03
mmu04550 Signaling pathways regulating pluripotency of stem cells	1.42	3.8E-03
mmu04330 Notch signaling pathway	1.43	3.8E-03
mmu00532 Glycosaminoglycan biosynthesis - chondroitin sulfate / dermatan sulfate	1.44	4.3E-03

mmu04666 Fc gamma R-mediated phagocytosis	1.39	4.8E-03
mmu02010 ABC transporters	1.28	1.1E-02
mmu04973 Carbohydrate digestion and absorption	1.26	1.2E-02
mmu00603 Glycosphingolipid biosynthesis - globo series	1.25	1.4E-02
mmu04144 Endocytosis	1.20	1.5E-02
mmu04620 Toll-like receptor signaling pathway	1.20	1.7E-02
mmu04520 Adherens junction	1.19	1.7E-02
mmu04971 Gastric acid secretion	1.16	2.0E-02
mmu04727 GABAergic synapse	1.14	2.3E-02
mmu04668 TNF signaling pathway	1.12	2.5E-02
mmu00500 Starch and sucrose metabolism	1.09	3.1E-02
mmu04961 Endocrine and other factor-regulated calcium reabsorption	1.08	3.2E-02
mmu04066 HIF-1 signaling pathway	1.07	3.2E-02
mmu04740 Olfactory transduction	1.06	3.3E-02
mmu04142 Lysosome	1.04	3.5E-02

**KEGG Pathways decreased in *fyuA* v NC**	**Magnitude of perturbation**	**FDR**
mmu03010 Ribosome	-5.82	5.0E-28
mmu00190 Oxidative phosphorylation	-4.80	5.2E-22
mmu01200 Carbon metabolism	-3.10	3.1E-10
mmu03050 Proteasome	-3.25	6.4E-10
mmu03040 Spliceosome	-3.01	7.5E-10
mmu03013 RNA transport	-2.61	1.1E-07
mmu00240 Pyrimidine metabolism	-2.61	1.1E-07
mmu04146 Peroxisome	-2.61	1.1E-07
mmu03008 Ribosome biogenesis in eukaryotes	-2.65	1.1E-07
mmu00983 Drug metabolism - other enzymes	-2.49	8.4E-07
mmu00830 Retinol metabolism	-2.23	1.2E-05
mmu00280 Valine, leucine and isoleucine degradation	-2.22	1.2E-05
mmu04110 Cell cycle	-2.06	4.5E-05
mmu03030 DNA replication	-2.16	4.6E-05
mmu00970 Aminoacyl-tRNA biosynthesis	-2.07	5.0E-05
mmu00920 Sulfur metabolism	-2.20	5.4E-05
mmu00140 Steroid hormone biosynthesis	-2.05	5.6E-05
mmu00640 Propanoate metabolism	-1.98	1.1E-04
mmu00040 Pentose and glucuronate interconversions	-1.96	1.1E-04
mmu00053 Ascorbate and aldarate metabolism	-1.98	1.5E-04
mmu03420 Nucleotide excision repair	-1.89	1.8E-04
mmu00630 Glyoxylate and dicarboxylate metabolism	-1.90	1.9E-04
mmu03020 RNA polymerase	-1.88	2.0E-04
mmu03060 Protein export	-1.88	2.6E-04
mmu01230 Biosynthesis of amino acids	-1.79	3.0E-04
mmu00020 Citrate cycle (TCA cycle)	-1.79	3.5E-04
mmu00982 Drug metabolism - cytochrome P450	-1.79	3.5E-04
mmu00980 Metabolism of xenobiotics by cytochrome P450	-1.78	3.5E-04
mmu03022 Basal transcription factors	-1.77	3.8E-04
mmu00860 Porphyrin and chlorophyll metabolism	-1.76	4.2E-04
mmu00071 Fatty acid degradation	-1.76	4.2E-04

mmu00650 Butanoate metabolism	-1.63	1.3E-03
mmu03015 mRNA surveillance pathway	-1.58	1.4E-03
mmu01212 Fatty acid metabolism	-1.57	1.6E-03
mmu00900 Terpenoid backbone biosynthesis	-1.62	1.6E-03
mmu03018 RNA degradation	-1.52	2.2E-03
mmu00561 Glycerolipid metabolism	-1.47	3.2E-03
mmu03440 Homologous recombination	-1.47	3.8E-03
mmu04120 Ubiquitin mediated proteolysis	-1.40	4.9E-03
mmu00270 Cysteine and methionine metabolism	-1.40	4.9E-03
mmu04141 Protein processing in endoplasmic reticulum	-1.37	5.7E-03
mmu03430 Mismatch repair	-1.41	6.1E-03
mmu00480 Glutathione metabolism	-1.36	6.3E-03
mmu00062 Fatty acid elongation	-1.33	8.0E-03
mmu03460 Fanconi anemia pathway	-1.33	8.0E-03
mmu03410 Base excision repair	-1.34	8.0E-03
mmu00620 Pyruvate metabolism	-1.32	8.3E-03
mmu00563 Glycosylphosphatidylinositol(GPI)-anchor biosynthesis	-1.32	9.1E-03
mmu00450 Selenocompound metabolism	-1.31	9.5E-03
mmu00910 Nitrogen metabolism	-1.28	1.4E-02
mmu00130 Ubiquinone and other terpenoid-quinone biosynthesis	-1.21	2.0E-02
mmu00380 Tryptophan metabolism	-1.09	3.1E-02
mmu00030 Pentose phosphate pathway	-1.10	3.1E-02
mmu01040 Biosynthesis of unsaturated fatty acids	-1.09	3.2E-02
mmu01210 2-Oxocarboxylic acid metabolism	-1.09	3.3E-02
mmu00260 Glycine, serine and threonine metabolism	-1.01	4.6E-02

Table S3: List of differentially expressed genes between colon tissue of Il10-/- mice colonized with either NC101 or △fyuA for 10 weeks

LogFC values are positive if △fyuA > NC101

Gene	logFC	FDR	Description
Cd5l	9.65	6.2E-19	CD5 antigen-like [Source:MGI Symbol;Acc:MGI:1334419]
Spocd1	8.32	3.0E-29	SPOC domain containing 1 [Source:MGI Symbol;Acc:MGI:3652045]
Cd209f	8.03	3.4E-29	CD209f antigen [Source:MGI Symbol;Acc:MGI:1916392]
Cd209g	7.61	2.9E-13	CD209g antigen [Source:MGI Symbol;Acc:MGI:1917442]
Fcrls	7.53	6.1E-18	Fc receptor-like S, scavenger receptor [Source:MGI Symbol;Acc:MGI:1933397]
Slc13a3	7.51	2.3E-12	solute carrier family 13 (sodium-dependent dicarboxylate transporter), member 3 [Source:MGI Symbol;Acc:MGI:2149635]
1700003M07Rik	6.98	3.3E-27	RIKEN cDNA 1700003M07 gene [Source:MGI Symbol;Acc:MGI:1919475]
Pnoc	6.82	6.0E-19	prepronociceptin [Source:MGI Symbol;Acc:MGI:105308]
Marco	6.68	3.7E-08	macrophage receptor with collagenous structure [Source:MGI Symbol;Acc:MGI:1309998]
Fcrl5	6.29	6.7E-17	Fc receptor-like 5 [Source:MGI Symbol;Acc:MGI:3053558]
Alpk3	6.20	4.5E-22	alpha-kinase 3 [Source:MGI Symbol;Acc:MGI:2151224]
Nrxn2	5.59	9.0E-29	neurexin II [Source:MGI Symbol;Acc:MGI:1096362]
Tmem63c	5.35	9.1E-15	transmembrane protein 63c [Source:MGI Symbol;Acc:MGI:2444386]
Kcnk12	5.18	3.7E-13	potassium channel, subfamily K, member 12 [Source:MGI Symbol;Acc:MGI:2684043]
Ttc21a	4.95	2.6E-28	tetratricopeptide repeat domain 21A [Source:MGI Symbol;Acc:MGI:1921302]
Cd300e	4.70	1.1E-22	CD300e antigen [Source:MGI Symbol;Acc:MGI:2387602]
Raver2	4.69	3.7E-42	ribonucleoprotein, PTB-binding 2 [Source:MGI Symbol;Acc:MGI:2443623]
Bai2	4.69	6.9E-22	brain-specific angiogenesis inhibitor 2 [Source:MGI Symbol;Acc:MGI:2451244]
Mfsd7c	4.65	2.5E-21	major facilitator superfamily domain containing 7C [Source:MGI Symbol;Acc:MGI:2384974]
Slamf9	4.64	1.5E-54	SLAM family member 9 [Source:MGI Symbol;Acc:MGI:1923692]
Ak8	4.60	7.4E-12	adenylate kinase 8 [Source:MGI Symbol;Acc:MGI:1916120]
Clec4b1	4.57	6.0E-23	C-type lectin domain family 4, member b1 [Source:MGI Symbol;Acc:MGI:1917060]
Gm1720	4.49	6.2E-12	predicted gene 1720 [Source:MGI Symbol;Acc:MGI:2686566]
Upk3b	4.34	8.9E-03	uroplakin 3B [Source:MGI Symbol;Acc:MGI:2140882]
Siglece	4.12	1.2E-38	sialic acid binding Ig-like lectin E [Source:MGI Symbol;Acc:MGI:1932475]
Emr1	4.11	4.2E-33	EGF-like module containing, mucin-like, hormone receptor-like sequence 1 [Source:MGI Symbol;Acc:MGI:106912]
Ip6k3	4.08	2.6E-14	inositol hexaphosphate kinase 3 [Source:MGI Symbol;Acc:MGI:3045325]
Igfn1	4.06	3.2E-09	immunoglobulin-like and fibronectin type III domain containing 1 [Source:MGI Symbol;Acc:MGI:3045352]
Gpr31b	4.05	6.9E-15	G protein-coupled receptor 31, D17Leh66b region [Source:MGI Symbol;Acc:MGI:1354372]
Rgag4	4.03	3.2E-34	retrotransposon gag domain containing 4 [Source:MGI Symbol;Acc:MGI:3045324]
Rasgrf1	4.00	1.1E-17	RAS protein-specific guanine nucleotide-releasing factor 1 [Source:MGI Symbol;Acc:MGI:99694]
Gfi1b	3.98	8.0E-17	growth factor independent 1B [Source:MGI Symbol;Acc:MGI:1276578]
Fpr1	3.97	5.2E-13	formyl peptide receptor 1 [Source:MGI Symbol;Acc:MGI:107443]
1700026L06Rik	3.95	9.6E-17	RIKEN cDNA 1700026L06 gene [Source:MGI Symbol;Acc:MGI:1917237]
Cyp2f2	3.85	1.1E-06	cytochrome P450, family 2, subfamily f, polypeptide 2 [Source:MGI Symbol;Acc:MGI:88608]
Inmt	3.78	2.7E-08	indolethylamine N-methyltransferase [Source:MGI Symbol;Acc:MGI:102963]
Sez6l2	3.77	4.0E-18	seizure related 6 homolog like 2 [Source:MGI Symbol;Acc:MGI:2385295]
Ebi3	3.71	3.5E-22	Epstein-Barr virus induced gene 3 [Source:MGI Symbol;Acc:MGI:1354171]
Zfp811	3.63	3.7E-13	zinc finger protein 811 [Source:MGI Symbol;Acc:MGI:2682944]
Nkx3-2	3.61	3.6E-04	NK3 homeobox 2 [Source:MGI Symbol;Acc:MGI:108015]
2900011O08Rik	3.57	1.5E-06	RIKEN cDNA 2900011O08 gene [Source:MGI Symbol;Acc:MGI:1914504]
Grin3a	3.57	2.2E-04	glutamate receptor ionotropic, NMDA3A [Source:MGI Symbol;Acc:MGI:1933206]
Ly6g6f	3.56	1.4E-07	lymphocyte antigen 6 complex, locus G6F [Source:MGI Symbol;Acc:MGI:3616082]
Gpr152	3.54	4.6E-11	G protein-coupled receptor 152 [Source:MGI Symbol;Acc:MGI:2685519]
Cd72	3.42	2.9E-20	CD72 antigen [Source:MGI Symbol;Acc:MGI:88345]
Rnase2a	3.39	1.9E-04	ribonuclease, RNase A family, 2A (liver, eosinophil-derived neurotoxin) [Source:MGI Symbol;Acc:MGI:1890465]
Pik3ap1	3.34	2.0E-23	phosphoinositide-3-kinase adaptor protein 1 [Source:MGI Symbol;Acc:MGI:1933177]
Adam23	3.33	5.0E-31	a disintegrin and metallopeptidase domain 23 [Source:MGI Symbol;Acc:MGI:1345162]

Gm16712	3.33	3.2E-10	predicted gene, 16712 [Source:MGI Symbol;Acc:MGI:4439636]
Prkcb	3.32	1.8E-25	protein kinase C, beta [Source:MGI Symbol;Acc:MGI:97596]
Clec4a2	3.29	4.1E-29	C-type lectin domain family 4, member a2 [Source:MGI Symbol;Acc:MGI:1349412]
Kif3c	3.20	6.7E-17	kinesin family member 3C [Source:MGI Symbol;Acc:MGI:107979]
Iglv2	3.19	8.5E-03	immunoglobulin lambda variable 2 [Source:MGI Symbol;Acc:MGI:99548]
Lst1	3.17	2.1E-17	leukocyte specific transcript 1 [Source:MGI Symbol;Acc:MGI:1096324]
Pltp	3.15	1.5E-32	phospholipid transfer protein [Source:MGI Symbol;Acc:MGI:103151]
Pou2f2	3.15	6.8E-23	POU domain, class 2, transcription factor 2 [Source:MGI Symbol;Acc:MGI:101897]
Cyp2c69	3.13	3.9E-03	cytochrome P450, family 2, subfamily c, polypeptide 69 [Source:MGI Symbol;Acc:MGI:3721049]
Spic	3.08	4.1E-12	Spi-C transcription factor (Spi-1/PU.1 related) [Source:MGI Symbol;Acc:MGI:1341168]
Iyd	3.06	4.0E-04	iodotyrosine deiodinase [Source:MGI Symbol;Acc:MGI:1917587]
Glt8d2	3.05	2.7E-09	glycosyltransferase 8 domain containing 2 [Source:MGI Symbol;Acc:MGI:1922032]
Susd1	3.05	5.8E-15	sushi domain containing 1 [Source:MGI Symbol;Acc:MGI:3651543]
#N/A	3.00	7.6E-20	#N/A
Grm4	2.97	3.0E-08	glutamate receptor, metabotropic 4 [Source:MGI Symbol;Acc:MGI:1351341]
Kcna2	2.97	1.7E-08	potassium voltage-gated channel, shaker-related subfamily, member 2 [Source:MGI Symbol;Acc:MGI:96659]
Gm13710	2.95	9.9E-08	predicted gene 13710 [Source:MGI Symbol;Acc:MGI:3650894]
Lmo2	2.92	2.4E-30	LIM domain only 2 [Source:MGI Symbol;Acc:MGI:102811]
Gpr126	2.88	6.8E-12	G protein-coupled receptor 126 [Source:MGI Symbol;Acc:MGI:1916151]
Lppr4	2.86	2.0E-05	lipid phosphate phosphatase-related protein type 4 [Source:MGI Symbol;Acc:MGI:106530]
Pgbd5	2.86	1.2E-07	piggyBac transposable element derived 5 [Source:MGI Symbol;Acc:MGI:2429955]
C3ar1	2.85	3.7E-21	complement component 3a receptor 1 [Source:MGI Symbol;Acc:MGI:1097680]
Ighv1-12	2.85	1.4E-04	immunoglobulin heavy variable V1-12 [Source:MGI Symbol;Acc:MGI:3646284]
Fcgr4	2.85	1.4E-13	Fc receptor, IgG, low affinity IV [Source:MGI Symbol;Acc:MGI:2179523]
Msln	2.84	9.4E-04	mesothelin [Source:MGI Symbol;Acc:MGI:1888992]
Hebp1	2.83	1.5E-24	heme binding protein 1 [Source:MGI Symbol;Acc:MGI:1333880]
Cd300lh	2.83	2.2E-05	CD300 antigen like family member H [Source:MGI Symbol;Acc:MGI:2687214]
Gria1	2.82	9.7E-06	glutamate receptor, ionotropic, AMPA1 (alpha 1) [Source:MGI Symbol;Acc:MGI:95808]
Zmat4	2.81	7.4E-03	zinc finger, matrin type 4 [Source:MGI Symbol;Acc:MGI:2443497]
Cacna1g	2.80	3.8E-08	calcium channel, voltage-dependent, T type, alpha 1G subunit [Source:MGI Symbol;Acc:MGI:1201678]
#N/A	2.80	2.9E-14	#N/A
Fgf11	2.77	2.1E-21	fibroblast growth factor 11 [Source:MGI Symbol;Acc:MGI:109167]
Clec4a1	2.76	6.0E-18	C-type lectin domain family 4, member a1 [Source:MGI Symbol;Acc:MGI:3036291]
Afp	2.75	1.0E-06	alpha fetoprotein [Source:MGI Symbol;Acc:MGI:87951]
Ptprz1	2.72	1.5E-05	protein tyrosine phosphatase, receptor type Z, polypeptide 1 [Source:MGI Symbol;Acc:MGI:97816]
Fam150b	2.72	9.9E-04	family with sequence similarity 150, member B [Source:MGI Symbol;Acc:MGI:3697448]
Pdcd1lg2	2.72	1.7E-12	programmed cell death 1 ligand 2 [Source:MGI Symbol;Acc:MGI:1930125]
A630033H20Rik	2.72	5.8E-10	RIKEN cDNA A630033H20 gene [Source:MGI Symbol;Acc:MGI:2441814]
B3galnt1	2.69	7.8E-13	UDP-GalNAc:betaGlcNAc beta 1,3-galactosaminyltransferase, polypeptide 1 [Source:MGI Symbol;Acc:MGI:1349405]
Cckar	2.69	2.7E-03	cholecystokinin A receptor [Source:MGI Symbol;Acc:MGI:99478]
Csrnp3	2.68	3.1E-04	cysteine-serine-rich nuclear protein 3 [Source:MGI Symbol;Acc:MGI:1925021]
Cd180	2.66	1.0E-17	CD180 antigen [Source:MGI Symbol;Acc:MGI:1194924]
Ptprm	2.65	1.7E-12	protein tyrosine phosphatase, receptor type, M [Source:MGI Symbol;Acc:MGI:102694]
Esr2	2.63	7.1E-03	estrogen receptor 2 (beta) [Source:MGI Symbol;Acc:MGI:109392]
Clec12a	2.60	6.4E-12	C-type lectin domain family 12, member a [Source:MGI Symbol;Acc:MGI:3040968]
Ighm	2.60	1.2E-31	immunoglobulin heavy constant mu [Source:MGI Symbol;Acc:MGI:96448]
Dbh	2.55	8.6E-04	dopamine beta hydroxylase [Source:MGI Symbol;Acc:MGI:94864]
C7	2.55	1.4E-02	complement component 7 [Source:MGI Symbol;Acc:MGI:88235]
Rgs18	2.55	1.9E-08	regulator of G-protein signaling 18 [Source:MGI Symbol;Acc:MGI:1927498]
Avil	2.54	1.5E-17	advillin [Source:MGI Symbol;Acc:MGI:1333798]
Ugt8a	2.54	3.9E-04	UDP galactosyltransferase 8A [Source:MGI Symbol;Acc:MGI:109522]
A630001G21Rik	2.54	8.1E-19	RIKEN cDNA A630001G21 gene [Source:MGI Symbol;Acc:MGI:2443131]
Plxnb3	2.54	6.4E-07	plexin B3 [Source:MGI Symbol;Acc:MGI:2154240]
Gm17705	2.53	2.5E-11	predicted gene, 17705 [Source:MGI Symbol;Acc:MGI:4937339]
1700011H14Rik	2.52	8.4E-03	RIKEN cDNA 1700011H14 gene [Source:MGI Symbol;Acc:MGI:1914332]
Aif1	2.51	1.3E-36	allograft inflammatory factor 1 [Source:MGI Symbol;Acc:MGI:1343098]

Ugt2b1	2.50	2.9E-03	UDP glucuronosyltransferase 2 family, polypeptide B1 [Source:MGI Symbol;Acc:MGI:1919023]
R74862	2.46	2.3E-05	expressed sequence R74862 [Source:MGI Symbol;Acc:MGI:2142382]
Itgam	2.45	2.4E-18	integrin alpha M [Source:MGI Symbol;Acc:MGI:96607]
Neurl2	2.45	1.4E-06	neuralized-like 2 (Drosophila) [Source:MGI Symbol;Acc:MGI:3043305]
Gm13012	2.44	5.4E-07	predicted gene 13012 [Source:MGI Symbol;Acc:MGI:3652270]
Abcg1	2.43	2.7E-22	ATP-binding cassette, sub-family G (WHITE), member 1 [Source:MGI Symbol;Acc:MGI:107704]
Fcer1g	2.43	2.2E-25	Fc receptor, IgE, high affinity I, gamma polypeptide [Source:MGI Symbol;Acc:MGI:95496]
Klhdc7a	2.43	3.8E-05	kelch domain containing 7A [Source:MGI Symbol;Acc:MGI:2444612]
Cdk14	2.42	1.7E-12	cyclin-dependent kinase 14 [Source:MGI Symbol;Acc:MGI:894318]
Rac2	2.41	1.5E-32	RAS-related C3 botulinum substrate 2 [Source:MGI Symbol;Acc:MGI:97846]
Rims3	2.40	2.2E-09	regulating synaptic membrane exocytosis 3 [Source:MGI Symbol;Acc:MGI:2443331]
Clps	2.39	5.5E-03	colipase, pancreatic [Source:MGI Symbol;Acc:MGI:88421]
Asb2	2.39	7.8E-21	ankyrin repeat and SOCS box-containing 2 [Source:MGI Symbol;Acc:MGI:1929743]
Sult1c2	2.39	4.0E-04	sulfotransferase family, cytosolic, 1C, member 2 [Source:MGI Symbol;Acc:MGI:1916333]
Batf3	2.38	7.0E-17	basic leucine zipper transcription factor, ATF-like 3 [Source:MGI Symbol;Acc:MGI:1925491]
Fat2	2.38	3.0E-04	FAT tumor suppressor homolog 2 (Drosophila) [Source:MGI Symbol;Acc:MGI:2685369]
Myom2	2.37	4.6E-03	myomesin 2 [Source:MGI Symbol;Acc:MGI:1328358]
Dbp	2.37	1.5E-04	D site albumin promoter binding protein [Source:MGI Symbol;Acc:MGI:94866]
Srpk3	2.36	8.9E-08	serine/arginine-rich protein specific kinase 3 [Source:MGI Symbol;Acc:MGI:1891338]
Fgd2	2.33	6.4E-24	FYVE, RhoGEF and PH domain containing 2 [Source:MGI Symbol;Acc:MGI:1347084]
Fmn2	2.32	2.4E-05	formin 2 [Source:MGI Symbol;Acc:MGI:1859252]
Krt84	2.32	2.8E-02	keratin 84 [Source:MGI Symbol;Acc:MGI:96700]
Matn4	2.31	1.3E-04	matrilin 4 [Source:MGI Symbol;Acc:MGI:1328314]
Ang4	2.31	4.3E-03	angiogenin, ribonuclease A family, member 4 [Source:MGI Symbol;Acc:MGI:2656551]
Lrrc25	2.30	5.2E-16	leucine rich repeat containing 25 [Source:MGI Symbol;Acc:MGI:2445284]
Thbs2	2.28	2.9E-05	thrombospondin 2 [Source:MGI Symbol;Acc:MGI:98738]
Iglc2	2.28	2.1E-02	immunoglobulin lambda constant 2 [Source:MGI Symbol;Acc:MGI:99547]
Tenm2	2.27	1.6E-03	teneurin transmembrane protein 2 [Source:MGI Symbol;Acc:MGI:1345184]
Rin3	2.27	1.4E-17	Ras and Rab interactor 3 [Source:MGI Symbol;Acc:MGI:2385708]
Arhgap4	2.26	1.0E-21	Rho GTPase activating protein 4 [Source:MGI Symbol;Acc:MGI:2159577]
Kcnn3	2.26	2.5E-03	potassium intermediate/small conductance calcium-activated channel, subfamily N, member 3 [Source:MGI Symbol;Acc:MGI:2153183]
Hck	2.26	1.8E-25	hemopoietic cell kinase [Source:MGI Symbol;Acc:MGI:96052]
Myrf	2.26	9.0E-04	myelin regulatory factor [Source:MGI Symbol;Acc:MGI:2684944]
Gpr85	2.24	1.1E-04	G protein-coupled receptor 85 [Source:MGI Symbol;Acc:MGI:1927851]
Slc14a1	2.23	1.6E-08	solute carrier family 14 (urea transporter), member 1 [Source:MGI Symbol;Acc:MGI:1351654]
Shh	2.23	8.7E-04	sonic hedgehog [Source:MGI Symbol;Acc:MGI:98297]
Sh3bp5	2.23	1.6E-20	SH3-domain binding protein 5 (BTK-associated) [Source:MGI Symbol;Acc:MGI:1344391]
Irak3	2.22	3.0E-13	interleukin-1 receptor-associated kinase 3 [Source:MGI Symbol;Acc:MGI:1921164]
Mchr1	2.21	3.4E-04	melanin-concentrating hormone receptor 1 [Source:MGI Symbol;Acc:MGI:2180756]
Lingo1	2.20	1.4E-03	leucine rich repeat and Ig domain containing 1 [Source:MGI Symbol;Acc:MGI:1915522]
Kcnd1	2.20	7.9E-09	potassium voltage-gated channel, Shal-related family, member 1 [Source:MGI Symbol;Acc:MGI:96671]
Trappc2	2.20	2.8E-14	trafficking protein particle complex 2 [Source:MGI Symbol;Acc:MGI:1913476]
Adrb2	2.19	4.7E-11	adrenergic receptor, beta 2 [Source:MGI Symbol;Acc:MGI:87938]
Tg	2.19	1.3E-03	thyroglobulin [Source:MGI Symbol;Acc:MGI:98733]
Cntn3	2.19	1.2E-08	contactin 3 [Source:MGI Symbol;Acc:MGI:99534]
Sfrp2	2.18	3.5E-03	secreted frizzled-related protein 2 [Source:MGI Symbol;Acc:MGI:108078]
AI593442	2.18	2.9E-02	expressed sequence AI593442 [Source:MGI Symbol;Acc:MGI:2143099]
Gstt1	2.18	3.6E-11	glutathione S-transferase, theta 1 [Source:MGI Symbol;Acc:MGI:107379]

Abca8a	2.17	2.5E-11	ATP-binding cassette, sub-family A (ABC1), member 8a [Source:MGI Symbol;Acc:MGI:2386846]
Cyp4b1	2.16	6.6E-10	cytochrome P450, family 4, subfamily b, polypeptide 1 [Source:MGI Symbol;Acc:MGI:103225]
Gpc3	2.16	2.1E-05	glypican 3 [Source:MGI Symbol;Acc:MGI:104903]
Abca1	2.16	2.7E-19	ATP-binding cassette, sub-family A (ABC1), member 1 [Source:MGI Symbol;Acc:MGI:99607]
Sorcs3	2.16	2.2E-02	sortilin-related VPS10 domain containing receptor 3 [Source:MGI Symbol;Acc:MGI:1913923]
Gdf10	2.16	3.5E-05	growth differentiation factor 10 [Source:MGI Symbol;Acc:MGI:95684]
Car14	2.16	1.4E-03	carbonic anhydrase 14 [Source:MGI Symbol;Acc:MGI:1344341]
BC067074	2.15	2.2E-03	cDNA sequence BC067074 [Source:MGI Symbol;Acc:MGI:3040697]
Cib2	2.15	1.6E-09	calcium and integrin binding family member 2 [Source:MGI Symbol;Acc:MGI:1929293]
Ankrd13d	2.15	2.3E-08	ankyrin repeat domain 13 family, member D [Source:MGI Symbol;Acc:MGI:1915673]
Ccr7	2.15	2.3E-06	chemokine (C-C motif) receptor 7 [Source:MGI Symbol;Acc:MGI:103011]
Cfp	2.14	2.3E-30	complement factor properdin [Source:MGI Symbol;Acc:MGI:97545]
Fyb	2.14	4.1E-18	FYN binding protein [Source:MGI Symbol;Acc:MGI:1346327]
Cyp2d9	2.14	4.9E-03	cytochrome P450, family 2, subfamily d, polypeptide 9 [Source:MGI Symbol;Acc:MGI:88606]
Tmc3	2.13	1.2E-07	transmembrane channel-like gene family 3 [Source:MGI Symbol;Acc:MGI:2669033]
Slc11a1	2.12	1.2E-11	solute carrier family 11 (proton-coupled divalent metal ion transporters), member 1 [Source:MGI Symbol;Acc:MGI:1345275]
Osbp2	2.12	5.9E-05	oxysterol binding protein 2 [Source:MGI Symbol;Acc:MGI:1921559]
Atp2b3	2.12	1.1E-02	ATPase, Ca++ transporting, plasma membrane 3 [Source:MGI Symbol;Acc:MGI:1347353]
Fads6	2.11	2.2E-05	fatty acid desaturase domain family, member 6 [Source:MGI Symbol;Acc:MGI:3039592]
Fhl3	2.11	1.6E-13	four and a half LIM domains 3 [Source:MGI Symbol;Acc:MGI:1341092]
Cd200r1	2.11	3.4E-05	CD200 receptor 1 [Source:MGI Symbol;Acc:MGI:1889024]
Hk3	2.10	1.4E-11	hexokinase 3 [Source:MGI Symbol;Acc:MGI:2670962]
Galnt15	2.10	1.1E-09	UDP-N-acetyl-alpha-D-galactosamine:polypeptide N-acetylgalactosaminyltransferase 15 [Source:MGI Symbol;Acc:MGI:1926004]
A430093F15Rik	2.10	9.4E-06	RIKEN cDNA A430093F15 gene [Source:MGI Symbol;Acc:MGI:2685520]
Slc45a1	2.09	5.4E-03	solute carrier family 45, member 1 [Source:MGI Symbol;Acc:MGI:2653235]
Cd36	2.09	7.4E-09	CD36 antigen [Source:MGI Symbol;Acc:MGI:107899]
F13a1	2.08	4.3E-04	coagulation factor XIII, A1 subunit [Source:MGI Symbol;Acc:MGI:1921395]
Oprk1	2.08	3.9E-02	opioid receptor, kappa 1 [Source:MGI Symbol;Acc:MGI:97439]
Ighv1-9	2.08	3.2E-02	immunoglobulin heavy variable V1-9 [Source:MGI Symbol;Acc:MGI:4439621]
Hpn	2.07	2.8E-05	hepsin [Source:MGI Symbol;Acc:MGI:1196620]
Ncam2	2.07	1.7E-03	neural cell adhesion molecule 2 [Source:MGI Symbol;Acc:MGI:97282]
Enpp3	2.06	2.2E-05	ectonucleotide pyrophosphatase/phosphodiesterase 3 [Source:MGI Symbol;Acc:MGI:2143702]
AB124611	2.06	7.9E-07	cDNA sequence AB124611 [Source:MGI Symbol;Acc:MGI:3043001]
Lonrf2	2.06	6.8E-04	LON peptidase N-terminal domain and ring finger 2 [Source:MGI Symbol;Acc:MGI:1920209]
St18	2.06	1.4E-04	suppression of tumorigenicity 18 [Source:MGI Symbol;Acc:MGI:2446700]
Tlr9	2.05	1.2E-08	toll-like receptor 9 [Source:MGI Symbol;Acc:MGI:1932389]
Cyp4f15	2.05	5.5E-03	cytochrome P450, family 4, subfamily f, polypeptide 15 [Source:MGI Symbol;Acc:MGI:2146921]
Pim2	2.05	6.9E-22	proviral integration site 2 [Source:MGI Symbol;Acc:MGI:97587]
Dct	2.04	2.8E-02	dopachrome tautomerase [Source:MGI Symbol;Acc:MGI:102563]
Basp1	2.04	1.7E-16	brain abundant, membrane attached signal protein 1 [Source:MGI Symbol;Acc:MGI:1917600]
Ticam2	2.04	9.8E-06	toll-like receptor adaptor molecule 2 [Source:MGI Symbol;Acc:MGI:3040056]
Gal	2.03	3.6E-03	galanin [Source:MGI Symbol;Acc:MGI:95637]
Cd244	2.02	5.4E-08	CD244 natural killer cell receptor 2B4 [Source:MGI Symbol;Acc:MGI:109294]
Cyth4	2.02	2.6E-28	cytohesin 4 [Source:MGI Symbol;Acc:MGI:2441702]
Hif3a	2.01	2.0E-05	hypoxia inducible factor 3, alpha subunit [Source:MGI Symbol;Acc:MGI:1859778]
Clec4a3	2.01	3.7E-08	C-type lectin domain family 4, member a3 [Source:MGI Symbol;Acc:MGI:1920399]
Sv2c	2.01	5.7E-04	synaptic vesicle glycoprotein 2c [Source:MGI Symbol;Acc:MGI:1922459]
Cabp4	2.00	9.1E-08	calcium binding protein 4 [Source:MGI Symbol;Acc:MGI:1920910]
Wfdc2	1.99	1.7E-02	WAP four-disulfide core domain 2 [Source:MGI Symbol;Acc:MGI:1914951]
Angptl7	1.99	1.0E-03	angiopoietin-like 7 [Source:MGI Symbol;Acc:MGI:3605801]

Pik3r5	1.98	1.2E-12	phosphoinositide-3-kinase, regulatory subunit 5, p101 [Source:MGI Symbol;Acc:MGI:2443588]
Dok3	1.98	7.6E-13	docking protein 3 [Source:MGI Symbol;Acc:MGI:1351490]
Gabrp	1.97	3.2E-03	gamma-aminobutyric acid (GABA) A receptor, pi [Source:MGI Symbol;Acc:MGI:2387597]
Omg	1.97	1.2E-02	oligodendrocyte myelin glycoprotein [Source:MGI Symbol;Acc:MGI:106586]
Smpx	1.97	2.7E-03	small muscle protein, X-linked [Source:MGI Symbol;Acc:MGI:1913356]
Klk1b4	1.97	3.4E-03	kallikrein 1-related pepidase b4 [Source:MGI Symbol;Acc:MGI:97320]
Reln	1.97	1.2E-06	reelin [Source:MGI Symbol;Acc:MGI:103022]
Fmod	1.97	5.3E-03	fibromodulin [Source:MGI Symbol;Acc:MGI:1328364]
Gm15675	1.97	6.0E-09	predicted gene 15675 [Source:MGI Symbol;Acc:MGI:3783117]
Tspan32	1.97	1.1E-10	tetraspanin 32 [Source:MGI Symbol;Acc:MGI:1350360]
Tspyl3	1.97	2.1E-11	TSPY-like 3 [Source:MGI Symbol;Acc:MGI:2139328]
Frmpd4	1.96	4.8E-03	FERM and PDZ domain containing 4 [Source:MGI Symbol;Acc:MGI:3042378]
Syt6	1.96	8.0E-04	synaptotagmin VI [Source:MGI Symbol;Acc:MGI:1859544]
Il9r	1.96	1.3E-05	interleukin 9 receptor [Source:MGI Symbol;Acc:MGI:96564]
Masp1	1.95	1.8E-03	mannan-binding lectin serine peptidase 1 [Source:MGI Symbol;Acc:MGI:88492]
Fmo2	1.95	2.2E-07	flavin containing monooxygenase 2 [Source:MGI Symbol;Acc:MGI:1916776]
Slc6a15	1.95	1.5E-03	solute carrier family 6 (neurotransmitter transporter), member 15 [Source:MGI Symbol;Acc:MGI:2143484]
Folr1	1.95	3.1E-02	folate receptor 1 (adult) [Source:MGI Symbol;Acc:MGI:95568]
Nr2e3	1.95	1.2E-02	nuclear receptor subfamily 2, group E, member 3 [Source:MGI Symbol;Acc:MGI:1346317]
Grap	1.94	1.7E-12	GRB2-related adaptor protein [Source:MGI Symbol;Acc:MGI:1918770]
Batf	1.94	3.0E-11	basic leucine zipper transcription factor, ATF-like [Source:MGI Symbol;Acc:MGI:1859147]
Acsm3	1.94	5.5E-03	acyl-CoA synthetase medium-chain family member 3 [Source:MGI Symbol;Acc:MGI:99538]
Ces1d	1.93	7.5E-08	carboxylesterase 1D [Source:MGI Symbol;Acc:MGI:2148202]
Pygm	1.93	2.2E-05	muscle glycogen phosphorylase [Source:MGI Symbol;Acc:MGI:97830]
Cyp2d12	1.93	1.3E-02	cytochrome P450, family 2, subfamily d, polypeptide 12 [Source:MGI Symbol;Acc:MGI:88604]
Sema3d	1.93	1.0E-04	sema domain, immunoglobulin domain (Ig), short basic domain, secreted, (semaphorin) 3D [Source:MGI Symbol;Acc:MGI:1860118]
Ano3	1.92	1.4E-02	anoctamin 3 [Source:MGI Symbol;Acc:MGI:3613666]
Slitrk3	1.92	1.6E-02	SLIT and NTRK-like family, member 3 [Source:MGI Symbol;Acc:MGI:2679447]
Atp8b5	1.92	2.0E-03	ATPase, class I, type 8B, member 5 [Source:MGI Symbol;Acc:MGI:2444287]
Sstr4	1.92	1.4E-02	somatostatin receptor 4 [Source:MGI Symbol;Acc:MGI:105372]
Mn1	1.92	3.0E-05	meningioma 1 [Source:MGI Symbol;Acc:MGI:1261813]
Rgs17	1.91	1.1E-02	regulator of G-protein signaling 17 [Source:MGI Symbol;Acc:MGI:1927469]
Nes	1.91	2.0E-04	nestin [Source:MGI Symbol;Acc:MGI:101784]
Ms4a6c	1.91	4.4E-13	membrane-spanning 4-domains, subfamily A, member 6C [Source:MGI Symbol;Acc:MGI:2385644]
Adamtsl4	1.91	7.0E-14	ADAMTS-like 4 [Source:MGI Symbol;Acc:MGI:2389008]
Fam198a	1.90	4.3E-03	family with sequence similarity 198, member A [Source:MGI Symbol;Acc:MGI:3041196]
Kcnc4	1.90	3.5E-02	potassium voltage gated channel, Shaw-related subfamily, member 4 [Source:MGI Symbol;Acc:MGI:96670]
Tns1	1.90	1.3E-09	tensin 1 [Source:MGI Symbol;Acc:MGI:104552]
Kcnq3	1.89	9.8E-03	potassium voltage-gated channel, subfamily Q, member 3 [Source:MGI Symbol;Acc:MGI:1336181]
Garnl3	1.89	6.2E-04	GTPase activating RANGAP domain-like 3 [Source:MGI Symbol;Acc:MGI:2139309]
Kcnc2	1.89	2.8E-02	potassium voltage gated channel, Shaw-related subfamily, member 2 [Source:MGI Symbol;Acc:MGI:96668]
Dkk2	1.89	1.2E-02	dickkopf homolog 2 (Xenopus laevis) [Source:MGI Symbol;Acc:MGI:1890663]
Asic2	1.88	3.1E-03	acid-sensing (proton-gated) ion channel 2 [Source:MGI Symbol;Acc:MGI:1100867]
Pik3cg	1.88	1.3E-08	phosphoinositide-3-kinase, catalytic, gamma polypeptide [Source:MGI Symbol;Acc:MGI:1353576]
Kcng2	1.87	3.6E-03	potassium voltage-gated channel, subfamily G, member 2 [Source:MGI Symbol;Acc:MGI:3694646]
Ric3	1.87	9.4E-06	resistance to inhibitors of cholinesterase 3 homolog (C. elegans) [Source:MGI Symbol;Acc:MGI:2443887]
Aebp1	1.87	5.1E-05	AE binding protein 1 [Source:MGI Symbol;Acc:MGI:1197012]
Vstm2a	1.87	2.0E-02	V-set and transmembrane domain containing 2A [Source:MGI Symbol;Acc:MGI:2384826]

Unc13c	1.87	9.2E-03	unc-13 homolog C (C. elegans) [Source:MGI Symbol;Acc:MGI:2149021]
Adcyap1r1	1.87	4.4E-04	adenylate cyclase activating polypeptide 1 receptor 1 [Source:MGI Symbol;Acc:MGI:108449]
Slc38a4	1.87	6.1E-03	solute carrier family 38, member 4 [Source:MGI Symbol;Acc:MGI:1916604]
Wisp1	1.87	7.6E-07	WNT1 inducible signaling pathway protein 1 [Source:MGI Symbol;Acc:MGI:1197008]
Mmrn1	1.87	4.5E-05	multimerin 1 [Source:MGI Symbol;Acc:MGI:1918195]
Slit3	1.86	1.1E-03	slit homolog 3 (Drosophila) [Source:MGI Symbol;Acc:MGI:1315202]
Gm684	1.86	2.4E-03	predicted gene 684 [Source:MGI Symbol;Acc:MGI:2685530]
Kirrel3	1.86	4.8E-03	kin of IRRE like 3 (Drosophila) [Source:MGI Symbol;Acc:MGI:1914953]
Lrrc4b	1.86	6.0E-04	leucine rich repeat containing 4B [Source:MGI Symbol;Acc:MGI:3027390]
Itgal	1.86	1.3E-12	integrin alpha L [Source:MGI Symbol;Acc:MGI:96606]
Csdc2	1.86	2.2E-03	cold shock domain containing C2, RNA binding [Source:MGI Symbol;Acc:MGI:2146027]
Adamts18	1.85	3.2E-02	a disintegrin-like and metallopeptidase (reprolysin type) with thrombospondin type 1 motif, 18 [Source:MGI Symbol;Acc:MGI:2442600]
Edil3	1.85	4.6E-04	EGF-like repeats and discoidin I-like domains 3 [Source:MGI Symbol;Acc:MGI:1329025]
Grp	1.85	7.8E-03	gastrin releasing peptide [Source:MGI Symbol;Acc:MGI:95833]
Pbld1	1.85	7.9E-03	phenazine biosynthesis-like protein domain containing 1 [Source:MGI Symbol;Acc:MGI:1915621]
Pitpnc1	1.85	2.2E-13	phosphatidylinositol transfer protein, cytoplasmic 1 [Source:MGI Symbol;Acc:MGI:1919045]
Trpm3	1.85	2.6E-03	transient receptor potential cation channel, subfamily M, member 3 [Source:MGI Symbol;Acc:MGI:2443101]
Rgl1	1.84	6.2E-13	ral guanine nucleotide dissociation stimulator,-like 1 [Source:MGI Symbol;Acc:MGI:107484]
Fzd9	1.84	2.2E-03	frizzled homolog 9 (Drosophila) [Source:MGI Symbol;Acc:MGI:1313278]
Vav1	1.84	4.4E-19	vav 1 oncogene [Source:MGI Symbol;Acc:MGI:98923]
Tnxb	1.84	1.2E-07	tenascin XB [Source:MGI Symbol;Acc:MGI:1932137]
Fhod3	1.84	8.7E-04	formin homology 2 domain containing 3 [Source:MGI Symbol;Acc:MGI:1925847]
Pld4	1.83	6.5E-19	phospholipase D family, member 4 [Source:MGI Symbol;Acc:MGI:2144765]
Slitrk1	1.83	9.5E-03	SLIT and NTRK-like family, member 1 [Source:MGI Symbol;Acc:MGI:2679446]
Arhgap44	1.83	2.0E-04	Rho GTPase activating protein 44 [Source:MGI Symbol;Acc:MGI:2144423]
Rcan2	1.83	1.2E-05	regulator of calcineurin 2 [Source:MGI Symbol;Acc:MGI:1858219]
Stag3	1.82	1.8E-04	stromal antigen 3 [Source:MGI Symbol;Acc:MGI:1355311]
Gypc	1.82	2.2E-12	glycophorin C [Source:MGI Symbol;Acc:MGI:1098566]
Hlf	1.82	6.5E-07	hepatic leukemia factor [Source:MGI Symbol;Acc:MGI:96108]
#N/A	1.82	2.3E-03	#N/A
Wnt2b	1.81	1.4E-04	wingless-type MMTV integration site family, member 2B [Source:MGI Symbol;Acc:MGI:1261834]
Mfap3l	1.81	2.0E-04	microfibrillar-associated protein 3-like [Source:MGI Symbol;Acc:MGI:1918556]
Lrrn1	1.81	1.9E-02	leucine rich repeat protein 1, neuronal [Source:MGI Symbol;Acc:MGI:106038]
Hs3st2	1.81	2.9E-02	heparan sulfate (glucosamine) 3-O-sulfotransferase 2 [Source:MGI Symbol;Acc:MGI:1333802]
Fat3	1.81	2.2E-02	FAT tumor suppressor homolog 3 (Drosophila) [Source:MGI Symbol;Acc:MGI:2444314]
Chrm3	1.81	2.5E-04	cholinergic receptor, muscarinic 3, cardiac [Source:MGI Symbol;Acc:MGI:88398]
Col25a1	1.80	1.0E-02	collagen, type XXV, alpha 1 [Source:MGI Symbol;Acc:MGI:1924268]
Slc15a3	1.80	8.7E-14	solute carrier family 15, member 3 [Source:MGI Symbol;Acc:MGI:1929691]
Gm14964	1.80	1.3E-03	predicted gene 14964 [Source:MGI Symbol;Acc:MGI:3641621]
Arhgdib	1.80	9.0E-19	Rho, GDP dissociation inhibitor (GDI) beta [Source:MGI Symbol;Acc:MGI:101940]
Fabp3	1.80	1.2E-02	fatty acid binding protein 3, muscle and heart [Source:MGI Symbol;Acc:MGI:95476]
Cntfr	1.80	8.2E-03	ciliary neurotrophic factor receptor [Source:MGI Symbol;Acc:MGI:99605]
Kcnt1	1.79	9.9E-03	potassium channel, subfamily T, member 1 [Source:MGI Symbol;Acc:MGI:1924627]
Arhgap20	1.79	2.3E-03	Rho GTPase activating protein 20 [Source:MGI Symbol;Acc:MGI:2445175]
Cd52	1.79	1.7E-19	CD52 antigen [Source:MGI Symbol;Acc:MGI:1346088]
Ccl27a	1.79	9.8E-03	chemokine (C-C motif) ligand 27A [Source:MGI Symbol;Acc:MGI:1343459]
Lama2	1.79	4.2E-05	laminin, alpha 2 [Source:MGI Symbol;Acc:MGI:99912]
Lpo	1.79	3.1E-02	lactoperoxidase [Source:MGI Symbol;Acc:MGI:1923363]
Slc4a8	1.79	4.7E-06	solute carrier family 4 (anion exchanger), member 8 [Source:MGI Symbol;Acc:MGI:1928745]
Scube2	1.79	6.8E-04	signal peptide, CUB domain, EGF-like 2 [Source:MGI Symbol;Acc:MGI:1928765]
Gas1	1.78	1.8E-04	growth arrest specific 1 [Source:MGI Symbol;Acc:MGI:95655]
Hepacam	1.78	4.5E-03	hepatocyte cell adhesion molecule [Source:MGI Symbol;Acc:MGI:1920177]

Vit	1.78	5.7E-05	vitrin [Source:MGI Symbol;Acc:MGI:1921449]
Stap1	1.77	9.0E-06	signal transducing adaptor family member 1 [Source:MGI Symbol;Acc:MGI:1926193]
Prkg1	1.77	6.7E-04	protein kinase, cGMP-dependent, type I [Source:MGI Symbol;Acc:MGI:108174]
Pcolce2	1.77	8.7E-04	procollagen C-endopeptidase enhancer 2 [Source:MGI Symbol;Acc:MGI:1923727]
Pcsk2	1.77	1.8E-02	proprotein convertase subtilisin/kexin type 2 [Source:MGI Symbol;Acc:MGI:97512]
Gfra1	1.77	2.4E-03	glial cell line derived neurotrophic factor family receptor alpha 1 [Source:MGI Symbol;Acc:MGI:1100842]
Shisa3	1.76	7.5E-03	shisa family member 3 [Source:MGI Symbol;Acc:MGI:3041225]
Kynu	1.76	1.1E-06	kynureninase (L-kynurenine hydrolase) [Source:MGI Symbol;Acc:MGI:1918039]
Gas7	1.76	4.5E-22	growth arrest specific 7 [Source:MGI Symbol;Acc:MGI:1202388]
Slc10a4	1.76	1.9E-02	solute carrier family 10 (sodium/bile acid cotransporter family), member 4 [Source:MGI Symbol;Acc:MGI:3606480]
Zfp804a	1.76	3.4E-02	zinc finger protein 804A [Source:MGI Symbol;Acc:MGI:2442949]
Caly	1.76	1.1E-02	calcyon neuron-specific vesicular protein [Source:MGI Symbol;Acc:MGI:1915816]
Prokr1	1.76	1.3E-04	prokineticin receptor 1 [Source:MGI Symbol;Acc:MGI:1929676]
Diras1	1.75	2.9E-02	DIRAS family, GTP-binding RAS-like 1 [Source:MGI Symbol;Acc:MGI:2183442]
Htr2b	1.75	1.6E-02	5-hydroxytryptamine (serotonin) receptor 2B [Source:MGI Symbol;Acc:MGI:109323]
Klhl30	1.75	7.7E-03	kelch-like 30 [Source:MGI Symbol;Acc:MGI:1918038]
Ly6h	1.75	2.6E-02	lymphocyte antigen 6 complex, locus H [Source:MGI Symbol;Acc:MGI:1346030]
Sik1	1.75	4.4E-12	salt inducible kinase 1 [Source:MGI Symbol;Acc:MGI:104754]
Chst7	1.75	1.5E-04	carbohydrate (N-acetylglucosamino) sulfotransferase 7 [Source:MGI Symbol;Acc:MGI:1891767]
Rab39	1.75	2.5E-05	RAB39, member RAS oncogene family [Source:MGI Symbol;Acc:MGI:2442855]
Cpne4	1.75	7.4E-04	copine IV [Source:MGI Symbol;Acc:MGI:1921270]
Pkhd1l1	1.75	2.3E-03	polycystic kidney and hepatic disease 1-like 1 [Source:MGI Symbol;Acc:MGI:2183153]
Grem2	1.75	6.7E-04	gremlin 2 homolog, cysteine knot superfamily (Xenopus laevis) [Source:MGI Symbol;Acc:MGI:1344367]
Slc2a12	1.75	7.3E-03	solute carrier family 2 (facilitated glucose transporter), member 12 [Source:MGI Symbol;Acc:MGI:3052471]
Svep1	1.74	2.7E-08	sushi, von Willebrand factor type A, EGF and pentraxin domain containing 1 [Source:MGI Symbol;Acc:MGI:1928849]
Flrt2	1.74	1.6E-04	fibronectin leucine rich transmembrane protein 2 [Source:MGI Symbol;Acc:MGI:3603594]
D130040H23Rik	1.74	7.9E-04	RIKEN cDNA D130040H23 gene [Source:MGI Symbol;Acc:MGI:2444324]
B3gnt6	1.74	1.5E-02	UDP-GlcNAc:betaGal beta-1,3-N-acetylglucosaminyltransferase 6 (core 3 synthase) [Source:MGI Symbol;Acc:MGI:3039603]
Tm6sf1	1.74	6.5E-07	transmembrane 6 superfamily member 1 [Source:MGI Symbol;Acc:MGI:1933209]
Fam188b	1.74	2.0E-04	family with sequence similarity 188, member B [Source:MGI Symbol;Acc:MGI:3583959]
Vat1l	1.74	3.1E-03	vesicle amine transport protein 1 homolog-like (T. californica) [Source:MGI Symbol;Acc:MGI:2142534]
Frrs1l	1.74	2.4E-02	ferric-chelate reductase 1 like [Source:MGI Symbol;Acc:MGI:2442704]
Scn2a1	1.73	5.4E-03	sodium channel, voltage-gated, type II, alpha 1 [Source:MGI Symbol;Acc:MGI:98248]
Rab38	1.73	5.5E-05	RAB38, member RAS oncogene family [Source:MGI Symbol;Acc:MGI:1919683]
Nrxn1	1.73	3.8E-03	neurexin I [Source:MGI Symbol;Acc:MGI:1096391]
Rbfox1	1.73	1.6E-02	RNA binding protein, fox-1 homolog (C. elegans) 1 [Source:MGI Symbol;Acc:MGI:1926224]
Emilin2	1.73	3.8E-10	elastin microfibril interfacer 2 [Source:MGI Symbol;Acc:MGI:2389136]
Sfmbt2	1.73	9.5E-04	Scm-like with four mbt domains 2 [Source:MGI Symbol;Acc:MGI:2447794]
Gm26947	1.72	1.5E-05	predicted gene, 26947 [Source:MGI Symbol;Acc:MGI:5504062]
C1qc	1.72	9.9E-19	complement component 1, q subcomponent, C chain [Source:MGI Symbol;Acc:MGI:88225]
Tmem229a	1.72	1.0E-02	transmembrane protein 229A [Source:MGI Symbol;Acc:MGI:2442812]
Kcnj5	1.72	8.4E-03	potassium inwardly-rectifying channel, subfamily J, member 5 [Source:MGI Symbol;Acc:MGI:104755]
Pip4k2a	1.72	1.2E-17	phosphatidylinositol-5-phosphate 4-kinase, type II, alpha [Source:MGI Symbol;Acc:MGI:1298206]
Asb16	1.71	1.1E-03	ankyrin repeat and SOCS box-containing 16 [Source:MGI Symbol;Acc:MGI:2654437]
Milr1	1.71	2.2E-08	mast cell immunoglobulin like receptor 1 [Source:MGI Symbol;Acc:MGI:2685731]
Tpd52l1	1.71	2.1E-02	tumor protein D52-like 1 [Source:MGI Symbol;Acc:MGI:1298386]
Glrb	1.70	3.6E-03	glycine receptor, beta subunit [Source:MGI Symbol;Acc:MGI:95751]

Prkar2b	1.70	2.8E-05	protein kinase, cAMP dependent regulatory, type II beta [Source:MGI Symbol;Acc:MGI:97760]
Casq2	1.70	2.4E-03	calsequestrin 2 [Source:MGI Symbol;Acc:MGI:1309469]
Vip	1.70	1.3E-04	vasoactive intestinal polypeptide [Source:MGI Symbol;Acc:MGI:98933]
Chadl	1.70	9.8E-04	chondroadherin-like [Source:MGI Symbol;Acc:MGI:3036284]
C1qb	1.70	2.4E-13	complement component 1, q subcomponent, beta polypeptide [Source:MGI Symbol;Acc:MGI:88224]
Bdh2	1.70	1.6E-02	3-hydroxybutyrate dehydrogenase, type 2 [Source:MGI Symbol;Acc:MGI:1917022]
Ecm2	1.69	3.0E-05	extracellular matrix protein 2, female organ and adipocyte specific [Source:MGI Symbol;Acc:MGI:3039578]
Adamtsl3	1.69	2.7E-03	ADAMTS-like 3 [Source:MGI Symbol;Acc:MGI:3028499]
Pygl	1.69	4.3E-07	liver glycogen phosphorylase [Source:MGI Symbol;Acc:MGI:97829]
BC035044	1.68	5.0E-06	cDNA sequence BC035044 [Source:MGI Symbol;Acc:MGI:2448540]
Kcnk2	1.68	4.0E-04	potassium channel, subfamily K, member 2 [Source:MGI Symbol;Acc:MGI:109366]
Sstr2	1.68	6.9E-03	somatostatin receptor 2 [Source:MGI Symbol;Acc:MGI:98328]
Gsta4	1.68	1.6E-03	glutathione S-transferase, alpha 4 [Source:MGI Symbol;Acc:MGI:1309515]
Scara5	1.68	6.9E-04	scavenger receptor class A, member 5 (putative) [Source:MGI Symbol;Acc:MGI:1918395]
Klhdc9	1.67	4.4E-02	kelch domain containing 9 [Source:MGI Symbol;Acc:MGI:1916124]
Dlg2	1.67	2.4E-02	discs, large homolog 2 (Drosophila) [Source:MGI Symbol;Acc:MGI:1344351]
Fermt3	1.67	1.4E-15	fermitin family homolog 3 (Drosophila) [Source:MGI Symbol;Acc:MGI:2147790]
Pik3r6	1.67	3.7E-08	phosphoinositide-3-kinase, regulatory subunit 6 [Source:MGI Symbol;Acc:MGI:2144613]
Scn1b	1.66	2.4E-10	sodium channel, voltage-gated, type I, beta [Source:MGI Symbol;Acc:MGI:98247]
Actr3b	1.65	1.1E-03	ARP3 actin-related protein 3B [Source:MGI Symbol;Acc:MGI:2661120]
Dpysl4	1.65	1.4E-02	dihydropyrimidinase-like 4 [Source:MGI Symbol;Acc:MGI:1349764]
Kctd12	1.65	8.0E-17	potassium channel tetramerisation domain containing 12 [Source:MGI Symbol;Acc:MGI:2145823]
Vsig2	1.65	6.0E-04	V-set and immunoglobulin domain containing 2 [Source:MGI Symbol;Acc:MGI:1928009]
Zscan18	1.65	4.6E-04	zinc finger and SCAN domain containing 18 [Source:MGI Symbol;Acc:MGI:3643810]
Podxl2	1.65	9.9E-04	podocalyxin-like 2 [Source:MGI Symbol;Acc:MGI:2442488]
Slc16a7	1.64	6.5E-04	solute carrier family 16 (monocarboxylic acid transporters), member 7 [Source:MGI Symbol;Acc:MGI:1330284]
Lyve1	1.64	9.0E-06	lymphatic vessel endothelial hyaluronan receptor 1 [Source:MGI Symbol;Acc:MGI:2136348]
Angptl2	1.64	1.1E-05	angiopoietin-like 2 [Source:MGI Symbol;Acc:MGI:1347002]
Zfp365	1.64	7.0E-03	zinc finger protein 365 [Source:MGI Symbol;Acc:MGI:2143676]
Nhsl2	1.64	4.7E-05	NHS-like 2 [Source:MGI Symbol;Acc:MGI:3645090]
Cyp2d10	1.64	1.8E-02	cytochrome P450, family 2, subfamily d, polypeptide 10 [Source:MGI Symbol;Acc:MGI:88602]
Unc80	1.64	1.7E-02	unc-80 homolog (C. elegans) [Source:MGI Symbol;Acc:MGI:2652882]
Spi1	1.63	2.4E-18	spleen focus forming virus (SFFV) proviral integration oncogene [Source:MGI Symbol;Acc:MGI:98282]
Lamc3	1.63	9.7E-04	laminin gamma 3 [Source:MGI Symbol;Acc:MGI:1344394]
Adamtsl1	1.63	5.5E-04	ADAMTS-like 1 [Source:MGI Symbol;Acc:MGI:1924989]
Srrm4	1.63	2.4E-02	serine/arginine repetitive matrix 4 [Source:MGI Symbol;Acc:MGI:1916205]
Zcchc12	1.63	5.5E-03	zinc finger, CCHC domain containing 12 [Source:MGI Symbol;Acc:MGI:1919943]
Ckm	1.63	1.2E-03	creatine kinase, muscle [Source:MGI Symbol;Acc:MGI:88413]
Rassf4	1.63	1.9E-18	Ras association (RalGDS/AF-6) domain family member 4 [Source:MGI Symbol;Acc:MGI:2386853]
Slc31a2	1.63	6.2E-17	solute carrier family 31, member 2 [Source:MGI Symbol;Acc:MGI:1333844]
Sla	1.63	1.1E-16	src-like adaptor [Source:MGI Symbol;Acc:MGI:104295]
4930578C19Rik	1.63	2.1E-03	RIKEN cDNA 4930578C19 gene [Source:MGI Symbol;Acc:MGI:1923155]
Sgcd	1.62	1.0E-03	sarcoglycan, delta (dystrophin-associated glycoprotein) [Source:MGI Symbol;Acc:MGI:1346525]
Cldn8	1.62	1.5E-02	claudin 8 [Source:MGI Symbol;Acc:MGI:1859286]
Syn1	1.62	2.8E-03	synapsin I [Source:MGI Symbol;Acc:MGI:98460]
Btk	1.62	4.5E-08	Bruton agammaglobulinemia tyrosine kinase [Source:MGI Symbol;Acc:MGI:88216]
Gabrg3	1.62	3.6E-02	gamma-aminobutyric acid (GABA) A receptor, subunit gamma 3 [Source:MGI Symbol;Acc:MGI:95624]
Htra3	1.62	1.0E-08	HtrA serine peptidase 3 [Source:MGI Symbol;Acc:MGI:1925808]
Tacr2	1.62	3.8E-03	tachykinin receptor 2 [Source:MGI Symbol;Acc:MGI:98477]
Moxd1	1.61	6.1E-03	monooxygenase, DBH-like 1 [Source:MGI Symbol;Acc:MGI:1921582]
Tmod1	1.61	3.6E-03	tropomodulin 1 [Source:MGI Symbol;Acc:MGI:98775]

Fstl5	1.61	3.5E-02	follistatin-like 5 [Source:MGI Symbol;Acc:MGI:2442179]
Bche	1.61	2.4E-04	butyrylcholinesterase [Source:MGI Symbol;Acc:MGI:894278]
Adap2	1.61	6.0E-07	ArfGAP with dual PH domains 2 [Source:MGI Symbol;Acc:MGI:2663075]
Ms4a7	1.61	2.7E-08	membrane-spanning 4-domains, subfamily A, member 7 [Source:MGI Symbol;Acc:MGI:1918846]
Scn9a	1.61	2.4E-02	sodium channel, voltage-gated, type IX, alpha [Source:MGI Symbol;Acc:MGI:107636]
Map3k8	1.60	3.0E-06	mitogen-activated protein kinase kinase kinase 8 [Source:MGI Symbol;Acc:MGI:1346878]
Ptgis	1.60	4.6E-03	prostaglandin I2 (prostacyclin) synthase [Source:MGI Symbol;Acc:MGI:1097156]
Astn1	1.60	2.3E-03	astrotactin 1 [Source:MGI Symbol;Acc:MGI:1098567]
1810041L15Rik	1.60	1.5E-02	RIKEN cDNA 1810041L15 gene [Source:MGI Symbol;Acc:MGI:1919551]
Megf6	1.60	1.5E-04	multiple EGF-like-domains 6 [Source:MGI Symbol;Acc:MGI:1919351]
Adcy1	1.60	9.8E-03	adenylate cyclase 1 [Source:MGI Symbol;Acc:MGI:99677]
Fam129c	1.60	1.2E-05	family with sequence similarity 129, member C [Source:MGI Symbol;Acc:MGI:3686743]
#N/A	1.60	3.3E-06	#N/A
Jph3	1.60	1.9E-02	junctophilin 3 [Source:MGI Symbol;Acc:MGI:1891497]
Mab21l1	1.60	3.1E-02	mab-21-like 1 (C. elegans) [Source:MGI Symbol;Acc:MGI:1333773]
Lrrtm3	1.60	3.3E-02	leucine rich repeat transmembrane neuronal 3 [Source:MGI Symbol;Acc:MGI:2389177]
Acss3	1.60	9.0E-03	acyl-CoA synthetase short-chain family member 3 [Source:MGI Symbol;Acc:MGI:2685720]
Slc6a17	1.60	2.0E-02	solute carrier family 6 (neurotransmitter transporter), member 17 [Source:MGI Symbol;Acc:MGI:2442535]
Gabrb3	1.59	1.0E-02	gamma-aminobutyric acid (GABA) A receptor, subunit beta 3 [Source:MGI Symbol;Acc:MGI:95621]
Fam49a	1.59	5.4E-08	family with sequence similarity 49, member A [Source:MGI Symbol;Acc:MGI:1261783]
Ttll6	1.59	4.2E-02	tubulin tyrosine ligase-like family, member 6 [Source:MGI Symbol;Acc:MGI:2683461]
Adora3	1.59	9.7E-04	adenosine A3 receptor [Source:MGI Symbol;Acc:MGI:104847]
Flt4	1.59	1.1E-04	FMS-like tyrosine kinase 4 [Source:MGI Symbol;Acc:MGI:95561]
Fam163a	1.59	2.3E-02	family with sequence similarity 163, member A [Source:MGI Symbol;Acc:MGI:3618859]
Crtac1	1.58	5.3E-03	cartilage acidic protein 1 [Source:MGI Symbol;Acc:MGI:1920082]
Cep85l	1.58	4.4E-03	centrosomal protein 85-like [Source:MGI Symbol;Acc:MGI:3642684]
Nell2	1.58	4.0E-02	NEL-like 2 [Source:MGI Symbol;Acc:MGI:1858510]
Lppr5	1.58	1.3E-02	lipid phosphate phosphatase-related protein type 5 [Source:MGI Symbol;Acc:MGI:1923019]
Lsamp	1.58	3.9E-03	limbic system-associated membrane protein [Source:MGI Symbol;Acc:MGI:1261760]
Nudt11	1.58	2.1E-02	nudix (nucleoside diphosphate linked moiety X)-type motif 11 [Source:MGI Symbol;Acc:MGI:1930957]
Pdzrn4	1.58	1.6E-02	PDZ domain containing RING finger 4 [Source:MGI Symbol;Acc:MGI:3056996]
Rassf9	1.58	8.5E-04	Ras association (RalGDS/AF-6) domain family (N-terminal) member 9 [Source:MGI Symbol;Acc:MGI:2384307]
Ccbe1	1.58	5.8E-03	collagen and calcium binding EGF domains 1 [Source:MGI Symbol;Acc:MGI:2445053]
Zeb2os	1.58	3.9E-03	zinc finger E-box binding homeobox 2, opposite strand [Source:MGI Symbol;Acc:MGI:3652108]
Hcls1	1.58	2.6E-13	hematopoietic cell specific Lyn substrate 1 [Source:MGI Symbol;Acc:MGI:104568]
Grip2	1.57	1.4E-03	glutamate receptor interacting protein 2 [Source:MGI Symbol;Acc:MGI:2681173]
Clmp	1.57	1.6E-05	CXADR-like membrane protein [Source:MGI Symbol;Acc:MGI:1918816]
Fstl4	1.57	2.6E-02	follistatin-like 4 [Source:MGI Symbol;Acc:MGI:2443199]
Mmp11	1.57	7.9E-05	matrix metallopeptidase 11 [Source:MGI Symbol;Acc:MGI:97008]
Pcdhb10	1.57	4.5E-03	protocadherin beta 10 [Source:MGI Symbol;Acc:MGI:2136745]
Npy4r	1.57	2.2E-02	neuropeptide Y receptor Y4 [Source:MGI Symbol;Acc:MGI:105374]
Il27	1.57	2.7E-02	interleukin 27 [Source:MGI Symbol;Acc:MGI:2384409]
Hmcn2	1.57	3.9E-05	hemicentin 2 [Source:MGI Symbol;Acc:MGI:2677838]
Gpr149	1.56	2.7E-02	G protein-coupled receptor 149 [Source:MGI Symbol;Acc:MGI:2443628]
Paqr6	1.56	3.2E-03	progestin and adipoQ receptor family member VI [Source:MGI Symbol;Acc:MGI:1916207]
Cldn10	1.56	4.8E-03	claudin 10 [Source:MGI Symbol;Acc:MGI:1913101]
Col14a1	1.56	2.2E-04	collagen, type XIV, alpha 1 [Source:MGI Symbol;Acc:MGI:1341272]
B630005N14Rik	1.56	7.9E-18	RIKEN cDNA B630005N14 gene [Source:MGI Symbol;Acc:MGI:2141466]

Acaa1b	1.56	3.6E-03	acetyl-Coenzyme A acyltransferase 1B [Source:MGI Symbol;Acc:MGI:3605455]
Cd200r4	1.56	3.7E-04	CD200 receptor 4 [Source:MGI Symbol;Acc:MGI:3036289]
Chrm1	1.56	1.4E-02	cholinergic receptor, muscarinic 1, CNS [Source:MGI Symbol;Acc:MGI:88396]
Fam124a	1.56	1.5E-04	family with sequence similarity 124, member A [Source:MGI Symbol;Acc:MGI:3645930]
Orm2	1.56	2.9E-03	orosomucoid 2 [Source:MGI Symbol;Acc:MGI:97444]
Adrbk2	1.56	2.6E-10	adrenergic receptor kinase, beta 2 [Source:MGI Symbol;Acc:MGI:87941]
Soga1	1.55	8.2E-08	suppressor of glucose, autophagy associated 1 [Source:MGI Symbol;Acc:MGI:2444575]
Kcna1	1.55	4.0E-04	potassium voltage-gated channel, shaker-related subfamily, member 1 [Source:MGI Symbol;Acc:MGI:96654]
Ogn	1.55	3.6E-06	osteoglycin [Source:MGI Symbol;Acc:MGI:109278]
Tenm4	1.55	6.4E-04	teneurin transmembrane protein 4 [Source:MGI Symbol;Acc:MGI:2447063]
Prima1	1.55	8.3E-03	proline rich membrane anchor 1 [Source:MGI Symbol;Acc:MGI:1926097]
Ms4a6d	1.55	1.2E-04	membrane-spanning 4-domains, subfamily A, member 6D [Source:MGI Symbol;Acc:MGI:1916024]
Stac	1.55	2.6E-02	src homology three (SH3) and cysteine rich domain [Source:MGI Symbol;Acc:MGI:1201400]
Gper1	1.55	2.5E-03	G protein-coupled estrogen receptor 1 [Source:MGI Symbol;Acc:MGI:1924104]
Ackr4	1.54	1.8E-03	atypical chemokine receptor 4 [Source:MGI Symbol;Acc:MGI:2181676]
Ndst3	1.54	6.8E-03	N-deacetylase/N-sulfotransferase (heparan glucosaminyl) 3 [Source:MGI Symbol;Acc:MGI:1932544]
A730017C20Rik	1.54	3.3E-02	RIKEN cDNA A730017C20 gene [Source:MGI Symbol;Acc:MGI:2442934]
Ryr2	1.54	5.3E-03	ryanodine receptor 2, cardiac [Source:MGI Symbol;Acc:MGI:99685]
Gng7	1.54	5.6E-04	guanine nucleotide binding protein (G protein), gamma 7 [Source:MGI Symbol;Acc:MGI:95787]
Tlr8	1.54	2.7E-03	toll-like receptor 8 [Source:MGI Symbol;Acc:MGI:2176887]
Sobp	1.54	1.4E-05	sine oculis-binding protein homolog (Drosophila) [Source:MGI Symbol;Acc:MGI:1924427]
Ly86	1.54	3.3E-08	lymphocyte antigen 86 [Source:MGI Symbol;Acc:MGI:1321404]
Gm12473	1.54	7.0E-03	predicted gene 12473 [Source:MGI Symbol;Acc:MGI:3652337]
Jph4	1.54	3.7E-03	junctophilin 4 [Source:MGI Symbol;Acc:MGI:2443113]
Prelp	1.54	1.4E-05	proline arginine-rich end leucine-rich repeat [Source:MGI Symbol;Acc:MGI:2151110]
Dpp6	1.54	2.4E-02	dipeptidylpeptidase 6 [Source:MGI Symbol;Acc:MGI:94921]
Arrb2	1.54	1.2E-15	arrestin, beta 2 [Source:MGI Symbol;Acc:MGI:99474]
Zkscan4	1.53	4.0E-03	zinc finger with KRAB and SCAN domains 4 [Source:MGI Symbol;Acc:MGI:3649412]
Kcnq2	1.53	2.5E-02	potassium voltage-gated channel, subfamily Q, member 2 [Source:MGI Symbol;Acc:MGI:1309503]
Stab1	1.53	1.1E-09	stabilin 1 [Source:MGI Symbol;Acc:MGI:2178742]
Klhl4	1.53	4.2E-03	kelch-like 4 [Source:MGI Symbol;Acc:MGI:2442829]
Slc22a3	1.53	3.0E-04	solute carrier family 22 (organic cation transporter), member 3 [Source:MGI Symbol;Acc:MGI:1333817]
Igfbp6	1.53	1.6E-03	insulin-like growth factor binding protein 6 [Source:MGI Symbol;Acc:MGI:96441]
Ppp2r2b	1.52	1.3E-02	protein phosphatase 2, regulatory subunit B, beta [Source:MGI Symbol;Acc:MGI:1920180]
Il10	1.52	1.2E-05	interleukin 10 [Source:MGI Symbol;Acc:MGI:96537]
Pgm5	1.52	2.2E-03	phosphoglucomutase 5 [Source:MGI Symbol;Acc:MGI:1925668]
Lrrn4cl	1.52	9.4E-04	LRRN4 C-terminal like [Source:MGI Symbol;Acc:MGI:1916102]
Fgl2	1.52	2.3E-10	fibrinogen-like protein 2 [Source:MGI Symbol;Acc:MGI:103266]
Ptgfr	1.52	4.6E-03	prostaglandin F receptor [Source:MGI Symbol;Acc:MGI:97796]
Bai3	1.52	2.8E-02	brain-specific angiogenesis inhibitor 3 [Source:MGI Symbol;Acc:MGI:2441837]
Kcna6	1.52	9.7E-03	potassium voltage-gated channel, shaker-related, subfamily, member 6 [Source:MGI Symbol;Acc:MGI:96663]
Cmtm5	1.52	8.5E-03	CKLF-like MARVEL transmembrane domain containing 5 [Source:MGI Symbol;Acc:MGI:2447164]
Iqsec3	1.51	4.8E-02	IQ motif and Sec7 domain 3 [Source:MGI Symbol;Acc:MGI:2677208]
Grm7	1.51	4.4E-02	glutamate receptor, metabotropic 7 [Source:MGI Symbol;Acc:MGI:1351344]
Musk	1.51	2.8E-03	muscle, skeletal, receptor tyrosine kinase [Source:MGI Symbol;Acc:MGI:103581]
Scml2	1.51	1.6E-03	sex comb on midleg-like 2 (Drosophila) [Source:MGI Symbol;Acc:MGI:1340042]
Opcml	1.51	3.7E-02	opioid binding protein/cell adhesion molecule-like [Source:MGI Symbol;Acc:MGI:97397]
Cadm2	1.51	1.2E-03	cell adhesion molecule 2 [Source:MGI Symbol;Acc:MGI:2442722]
Cyp26b1	1.51	2.2E-04	cytochrome P450, family 26, subfamily b, polypeptide 1 [Source:MGI Symbol;Acc:MGI:2176159]

Siglec1	1.51	2.5E-02	sialic acid binding Ig-like lectin 1, sialoadhesin [Source:MGI Symbol;Acc:MGI:99668]
Soga3	1.51	3.7E-02	SOGA family member 3 [Source:MGI Symbol;Acc:MGI:1914662]
Maf	1.51	2.2E-07	avian musculoaponeurotic fibrosarcoma (v-maf) AS42 oncogene homolog [Source:MGI Symbol;Acc:MGI:96909]
Abca8b	1.51	5.3E-04	ATP-binding cassette, sub-family A (ABC1), member 8b [Source:MGI Symbol;Acc:MGI:1351668]
Scn7a	1.51	4.5E-05	sodium channel, voltage-gated, type VII, alpha [Source:MGI Symbol;Acc:MGI:102965]
Fam110b	1.51	3.8E-04	family with sequence similarity 110, member B [Source:MGI Symbol;Acc:MGI:1916593]
Prr18	1.50	2.2E-02	proline rich 18 [Source:MGI Symbol;Acc:MGI:2443403]
Tmem200a	1.50	3.2E-02	transmembrane protein 200A [Source:MGI Symbol;Acc:MGI:1924470]
Zfpm2	1.50	2.3E-03	zinc finger protein, multitype 2 [Source:MGI Symbol;Acc:MGI:1334444]
Enpp2	1.50	2.5E-07	ectonucleotide pyrophosphatase/phosphodiesterase 2 [Source:MGI Symbol;Acc:MGI:1321390]
Hao2	1.50	1.0E-03	hydroxyacid oxidase 2 [Source:MGI Symbol;Acc:MGI:96012]
Wbscr17	1.50	6.0E-03	Williams-Beuren syndrome chromosome region 17 homolog (human) [Source:MGI Symbol;Acc:MGI:2137594]
Adamts19	1.49	1.0E-02	a disintegrin-like and metallopeptidase (reprolysin type) with thrombospondin type 1 motif, 19 [Source:MGI Symbol;Acc:MGI:2442875]
Ctnna2	1.49	4.4E-02	catenin (cadherin associated protein), alpha 2 [Source:MGI Symbol;Acc:MGI:88275]
Klf15	1.49	8.0E-04	Kruppel-like factor 15 [Source:MGI Symbol;Acc:MGI:1929988]
Tex14	1.49	8.4E-03	testis expressed gene 14 [Source:MGI Symbol;Acc:MGI:1933227]
Tmod2	1.49	4.3E-04	tropomodulin 2 [Source:MGI Symbol;Acc:MGI:1355335]
Col11a1	1.49	3.3E-02	collagen, type XI, alpha 1 [Source:MGI Symbol;Acc:MGI:88446]
Fzd10	1.49	1.5E-02	frizzled homolog 10 (Drosophila) [Source:MGI Symbol;Acc:MGI:2136761]
Brsk2	1.49	4.7E-03	BR serine/threonine kinase 2 [Source:MGI Symbol;Acc:MGI:1923020]
Slc16a2	1.49	1.1E-03	solute carrier family 16 (monocarboxylic acid transporters), member 2 [Source:MGI Symbol;Acc:MGI:1203732]
Msrb2	1.49	1.2E-03	methionine sulfoxide reductase B2 [Source:MGI Symbol;Acc:MGI:1923717]
Ap1s2	1.49	4.9E-07	adaptor-related protein complex 1, sigma 2 subunit [Source:MGI Symbol;Acc:MGI:1889383]
Mamdc2	1.48	4.0E-05	MAM domain containing 2 [Source:MGI Symbol;Acc:MGI:1918988]
Lum	1.48	2.2E-03	lumican [Source:MGI Symbol;Acc:MGI:109347]
Pcdhb6	1.48	7.1E-03	protocadherin beta 6 [Source:MGI Symbol;Acc:MGI:2136740]
Cdh19	1.48	2.2E-03	cadherin 19, type 2 [Source:MGI Symbol;Acc:MGI:3588198]
Sdc3	1.48	2.1E-04	syndecan 3 [Source:MGI Symbol;Acc:MGI:1349163]
Ccl21a	1.48	2.1E-04	chemokine (C-C motif) ligand 21A (serine) [Source:MGI Symbol;Acc:MGI:1349183]
Dner	1.48	2.1E-02	delta/notch-like EGF-related receptor [Source:MGI Symbol;Acc:MGI:2152889]
Gpr37l1	1.48	3.0E-03	G protein-coupled receptor 37-like 1 [Source:MGI Symbol;Acc:MGI:1928503]
Cd79b	1.48	9.5E-06	CD79B antigen [Source:MGI Symbol;Acc:MGI:96431]
Gucy1a3	1.48	6.1E-04	guanylate cyclase 1, soluble, alpha 3 [Source:MGI Symbol;Acc:MGI:1926562]
Ncf1	1.48	9.0E-13	neutrophil cytosolic factor 1 [Source:MGI Symbol;Acc:MGI:97283]
Smad9	1.47	1.4E-03	SMAD family member 9 [Source:MGI Symbol;Acc:MGI:1859993]
Fam212a	1.47	2.0E-03	family with sequence similarity 212, member A [Source:MGI Symbol;Acc:MGI:1915426]
Mrgprf	1.47	9.4E-04	MAS-related GPR, member F [Source:MGI Symbol;Acc:MGI:2384823]
Efna5	1.47	2.8E-02	ephrin A5 [Source:MGI Symbol;Acc:MGI:107444]
Calb2	1.47	3.3E-03	calbindin 2 [Source:MGI Symbol;Acc:MGI:101914]
Siah2	1.47	2.5E-09	seven in absentia 2 [Source:MGI Symbol;Acc:MGI:108062]
Tmem255a	1.47	4.0E-02	transmembrane protein 255A [Source:MGI Symbol;Acc:MGI:3045722]
Camk2a	1.47	5.2E-04	calcium/calmodulin-dependent protein kinase II alpha [Source:MGI Symbol;Acc:MGI:88256]
St3gal2	1.47	3.9E-05	ST3 beta-galactoside alpha-2,3-sialyltransferase 2 [Source:MGI Symbol;Acc:MGI:99427]
Il6ra	1.47	6.6E-06	interleukin 6 receptor, alpha [Source:MGI Symbol;Acc:MGI:105304]
Fndc7	1.46	2.3E-03	fibronectin type III domain containing 7 [Source:MGI Symbol;Acc:MGI:2443535]
Kcnj12	1.46	2.6E-02	potassium inwardly-rectifying channel, subfamily J, member 12 [Source:MGI Symbol;Acc:MGI:108495]
Apc2	1.46	2.7E-02	adenomatosis polyposis coli 2 [Source:MGI Symbol;Acc:MGI:1346052]
Lrmp	1.46	2.0E-09	lymphoid-restricted membrane protein [Source:MGI Symbol;Acc:MGI:108424]
9330182L06Rik	1.46	1.6E-02	RIKEN cDNA 9330182L06 gene [Source:MGI Symbol;Acc:MGI:2443264]
Plin4	1.46	6.3E-04	perilipin 4 [Source:MGI Symbol;Acc:MGI:1929709]
Tmem200b	1.46	3.4E-03	transmembrane protein 200B [Source:MGI Symbol;Acc:MGI:3646343]

Myo7a	1.46	3.4E-05	myosin VIIA [Source:MGI Symbol;Acc:MGI:104510]
Sorbs3	1.46	6.3E-08	sorbin and SH3 domain containing 3 [Source:MGI Symbol;Acc:MGI:700013]
Hhatl	1.46	1.2E-02	hedgehog acyltransferase-like [Source:MGI Symbol;Acc:MGI:1922020]
Diras2	1.45	1.3E-02	DIRAS family, GTP-binding RAS-like 2 [Source:MGI Symbol;Acc:MGI:1915453]
Thsd7a	1.45	3.4E-03	thrombospondin, type I, domain containing 7A [Source:MGI Symbol;Acc:MGI:2685683]
Nsg1	1.45	3.5E-03	neuron specific gene family member 1 [Source:MGI Symbol;Acc:MGI:109149]
Crabp1	1.45	2.0E-03	cellular retinoic acid binding protein I [Source:MGI Symbol;Acc:MGI:88490]
Apod	1.45	2.9E-02	apolipoprotein D [Source:MGI Symbol;Acc:MGI:88056]
Prrg3	1.45	6.4E-03	proline rich Gla (G-carboxyglutamic acid) 3 (transmembrane) [Source:MGI Symbol;Acc:MGI:2685214]
Itln1	1.45	2.4E-02	intelectin 1 (galactofuranose binding) [Source:MGI Symbol;Acc:MGI:1333831]
Rnf112	1.45	4.7E-02	ring finger protein 112 [Source:MGI Symbol;Acc:MGI:106611]
Atp2b2	1.45	4.3E-03	ATPase, Ca++ transporting, plasma membrane 2 [Source:MGI Symbol;Acc:MGI:105368]
Ppapdc3	1.45	6.3E-03	phosphatidic acid phosphatase type 2 domain containing 3 [Source:MGI Symbol;Acc:MGI:2445183]
Maml2	1.45	2.4E-06	mastermind like 2 (Drosophila) [Source:MGI Symbol;Acc:MGI:2389460]
Thsd4	1.45	7.2E-05	thrombospondin, type I, domain containing 4 [Source:MGI Symbol;Acc:MGI:2672033]
Evi2a	1.44	5.7E-10	ecotropic viral integration site 2a [Source:MGI Symbol;Acc:MGI:95458]
Mgll	1.44	3.3E-17	monoglyceride lipase [Source:MGI Symbol;Acc:MGI:1346042]
Astn2	1.44	3.5E-03	astrotactin 2 [Source:MGI Symbol;Acc:MGI:1889277]
Shank1	1.44	1.6E-03	SH3/ankyrin domain gene 1 [Source:MGI Symbol;Acc:MGI:3613677]
Pou3f3	1.44	4.0E-02	POU domain, class 3, transcription factor 3 [Source:MGI Symbol;Acc:MGI:102564]
Sox5	1.44	2.6E-04	SRY (sex determining region Y)-box 5 [Source:MGI Symbol;Acc:MGI:98367]
Wdfy3	1.44	1.1E-10	WD repeat and FYVE domain containing 3 [Source:MGI Symbol;Acc:MGI:1096875]
Spib	1.44	2.8E-05	Spi-B transcription factor (Spi-1/PU.1 related) [Source:MGI Symbol;Acc:MGI:892986]
Nckap1l	1.44	3.5E-11	NCK associated protein 1 like [Source:MGI Symbol;Acc:MGI:1926063]
Nynrin	1.44	5.8E-03	NYN domain and retroviral integrase containing [Source:MGI Symbol;Acc:MGI:2652872]
Kcnb1	1.44	6.9E-03	potassium voltage gated channel, Shab-related subfamily, member 1 [Source:MGI Symbol;Acc:MGI:96666]
Bnc2	1.43	3.7E-02	basonuclin 2 [Source:MGI Symbol;Acc:MGI:2443805]
Trnp1	1.43	4.3E-03	TMF1-regulated nuclear protein 1 [Source:MGI Symbol;Acc:MGI:1916789]
Chrdl1	1.43	3.1E-03	chordin-like 1 [Source:MGI Symbol;Acc:MGI:1933172]
Sfrp5	1.43	1.4E-02	secreted frizzled-related sequence protein 5 [Source:MGI Symbol;Acc:MGI:1860298]
Gm17029	1.43	2.4E-02	predicted gene 17029 [Source:MGI Symbol;Acc:MGI:4937856]
Pln	1.43	2.4E-03	phospholamban [Source:MGI Symbol;Acc:MGI:97622]
Cacnb2	1.43	7.0E-03	calcium channel, voltage-dependent, beta 2 subunit [Source:MGI Symbol;Acc:MGI:894644]
Tmcc2	1.43	1.8E-04	transmembrane and coiled-coil domains 2 [Source:MGI Symbol;Acc:MGI:1916125]
Ntng1	1.43	5.8E-03	netrin G1 [Source:MGI Symbol;Acc:MGI:1934028]
Syt2	1.43	1.2E-02	synaptotagmin II [Source:MGI Symbol;Acc:MGI:99666]
Lrrc4c	1.43	1.5E-02	leucine rich repeat containing 4C [Source:MGI Symbol;Acc:MGI:2442636]
Chrnb4	1.42	9.8E-03	cholinergic receptor, nicotinic, beta polypeptide 4 [Source:MGI Symbol;Acc:MGI:87892]
Penk	1.42	3.0E-03	preproenkephalin [Source:MGI Symbol;Acc:MGI:104629]
Hspb7	1.42	2.7E-03	heat shock protein family, member 7 (cardiovascular) [Source:MGI Symbol;Acc:MGI:1352494]
Arhgef9	1.42	1.4E-03	CDC42 guanine nucleotide exchange factor (GEF) 9 [Source:MGI Symbol;Acc:MGI:2442233]
Apba2	1.42	1.4E-02	amyloid beta (A4) precursor protein-binding, family A, member 2 [Source:MGI Symbol;Acc:MGI:1261791]
Kcnt2	1.42	1.8E-02	potassium channel, subfamily T, member 2 [Source:MGI Symbol;Acc:MGI:3036273]
Ptpn6	1.42	4.6E-16	protein tyrosine phosphatase, non-receptor type 6 [Source:MGI Symbol;Acc:MGI:96055]
Insl5	1.41	4.7E-02	insulin-like 5 [Source:MGI Symbol;Acc:MGI:1346085]
Trerf1	1.41	3.2E-07	transcriptional regulating factor 1 [Source:MGI Symbol;Acc:MGI:2442086]
Dlgap1	1.41	2.8E-03	discs, large (Drosophila) homolog-associated protein 1 [Source:MGI Symbol;Acc:MGI:1346065]

Ap3b2	1.41	2.2E-02	adaptor-related protein complex 3, beta 2 subunit [Source:MGI Symbol;Acc:MGI:1100869]
Dock2	1.41	8.5E-10	dedicator of cyto-kinesis 2 [Source:MGI Symbol;Acc:MGI:2149010]
Hpcal4	1.41	4.1E-02	hippocalcin-like 4 [Source:MGI Symbol;Acc:MGI:2157521]
Eda	1.41	9.7E-03	ectodysplasin-A [Source:MGI Symbol;Acc:MGI:1195272]
Ces1g	1.41	2.2E-04	carboxylesterase 1G [Source:MGI Symbol;Acc:MGI:88378]
Bank1	1.41	7.1E-04	B cell scaffold protein with ankyrin repeats 1 [Source:MGI Symbol;Acc:MGI:2442120]
Osr1	1.41	1.4E-02	odd-skipped related 1 (Drosophila) [Source:MGI Symbol;Acc:MGI:1344424]
Lcp2	1.40	1.4E-08	lymphocyte cytosolic protein 2 [Source:MGI Symbol;Acc:MGI:1321402]
Scarf2	1.40	1.6E-06	scavenger receptor class F, member 2 [Source:MGI Symbol;Acc:MGI:1858430]
Rit2	1.40	4.2E-02	Ras-like without CAAX 2 [Source:MGI Symbol;Acc:MGI:108054]
Phox2b	1.40	1.5E-02	paired-like homeobox 2b [Source:MGI Symbol;Acc:MGI:1100882]
Eef1a2	1.40	1.5E-02	eukaryotic translation elongation factor 1 alpha 2 [Source:MGI Symbol;Acc:MGI:1096317]
Slc2a5	1.40	4.4E-02	solute carrier family 2 (facilitated glucose transporter), member 5 [Source:MGI Symbol;Acc:MGI:1928369]
Ccl11	1.40	4.8E-04	chemokine (C-C motif) ligand 11 [Source:MGI Symbol;Acc:MGI:103576]
Synpo2	1.40	3.8E-03	synaptopodin 2 [Source:MGI Symbol;Acc:MGI:2153070]
Figf	1.40	2.2E-03	c-fos induced growth factor [Source:MGI Symbol;Acc:MGI:108037]
Cadps	1.40	5.5E-03	Ca2+-dependent secretion activator [Source:MGI Symbol;Acc:MGI:1350922]
AW551984	1.40	4.6E-02	expressed sequence AW551984 [Source:MGI Symbol;Acc:MGI:2143322]
Tbc1d2b	1.40	5.5E-12	TBC1 domain family, member 2B [Source:MGI Symbol;Acc:MGI:1914266]
Laptm5	1.39	1.0E-11	lysosomal-associated protein transmembrane 5 [Source:MGI Symbol;Acc:MGI:108046]
Galnt16	1.39	7.1E-03	UDP-N-acetyl-alpha-D-galactosamine:polypeptide N-acetylgalactosaminyltransferase 16 [Source:MGI Symbol;Acc:MGI:1917754]
Gpc6	1.39	6.8E-04	glypican 6 [Source:MGI Symbol;Acc:MGI:1346322]
Nr1d1	1.39	9.8E-06	nuclear receptor subfamily 1, group D, member 1 [Source:MGI Symbol;Acc:MGI:2444210]
Pcdhb4	1.39	8.2E-03	protocadherin beta 4 [Source:MGI Symbol;Acc:MGI:2136738]
Ccdc106	1.39	6.1E-03	coiled-coil domain containing 106 [Source:MGI Symbol;Acc:MGI:2385900]
Kcnd3	1.39	1.1E-03	potassium voltage-gated channel, Shal-related family, member 3 [Source:MGI Symbol;Acc:MGI:1928743]
Meis2	1.39	4.4E-03	Meis homeobox 2 [Source:MGI Symbol;Acc:MGI:108564]
S1pr2	1.39	1.1E-05	sphingosine-1-phosphate receptor 2 [Source:MGI Symbol;Acc:MGI:99569]
Ptplad2	1.39	1.8E-04	protein tyrosine phosphatase-like A domain containing 2 [Source:MGI Symbol;Acc:MGI:1914025]
Lgi4	1.39	5.9E-03	leucine-rich repeat LGI family, member 4 [Source:MGI Symbol;Acc:MGI:2180197]
Tmem130	1.39	2.3E-02	transmembrane protein 130 [Source:MGI Symbol;Acc:MGI:3607706]
Foxp2	1.39	5.7E-03	forkhead box P2 [Source:MGI Symbol;Acc:MGI:2148705]
B3galt2	1.39	9.1E-03	UDP-Gal:betaGlcNAc beta 1,3-galactosyltransferase, polypeptide 2 [Source:MGI Symbol;Acc:MGI:1349461]
Ptpn14	1.39	1.5E-05	protein tyrosine phosphatase, non-receptor type 14 [Source:MGI Symbol;Acc:MGI:102467]
Gpr22	1.39	2.6E-02	G protein-coupled receptor 22 [Source:MGI Symbol;Acc:MGI:1920260]
Fam46b	1.38	4.1E-02	family with sequence similarity 46, member B [Source:MGI Symbol;Acc:MGI:2140500]
Sgca	1.38	1.4E-02	sarcoglycan, alpha (dystrophin-associated glycoprotein) [Source:MGI Symbol;Acc:MGI:894698]
Coro2b	1.38	7.3E-04	coronin, actin binding protein, 2B [Source:MGI Symbol;Acc:MGI:2444283]
Aqp1	1.38	6.7E-06	aquaporin 1 [Source:MGI Symbol;Acc:MGI:103201]
Bves	1.38	4.8E-02	blood vessel epicardial substance [Source:MGI Symbol;Acc:MGI:1346013]
Apbb1ip	1.38	1.0E-11	amyloid beta (A4) precursor protein-binding, family B, member 1 interacting protein [Source:MGI Symbol;Acc:MGI:1861354]
Lrrc3	1.38	7.6E-03	leucine rich repeat containing 3 [Source:MGI Symbol;Acc:MGI:2447899]
3632451O06Rik	1.38	3.8E-03	RIKEN cDNA 3632451O06 gene [Source:MGI Symbol;Acc:MGI:1914669]
Limch1	1.38	5.2E-04	LIM and calponin homology domains 1 [Source:MGI Symbol;Acc:MGI:1924819]
Syndig1	1.38	2.8E-02	synapse differentiation inducing 1 [Source:MGI Symbol;Acc:MGI:3702158]
Klhl13	1.38	4.6E-03	kelch-like 13 [Source:MGI Symbol;Acc:MGI:1914705]
Peg3	1.38	4.9E-04	paternally expressed 3 [Source:MGI Symbol;Acc:MGI:104748]
Afm	1.38	1.9E-02	afamin [Source:MGI Symbol;Acc:MGI:2429409]
Npas4	1.38	9.0E-03	neuronal PAS domain protein 4 [Source:MGI Symbol;Acc:MGI:2664186]
Aoc3	1.38	1.3E-03	amine oxidase, copper containing 3 [Source:MGI Symbol;Acc:MGI:1306797]
St8sia6	1.38	5.9E-05	ST8 alpha-N-acetyl-neuraminide alpha-2,8-sialyltransferase 6 [Source:MGI Symbol;Acc:MGI:2386797]

Srpx	1.38	2.1E-02	sushi-repeat-containing protein [Source:MGI Symbol;Acc:MGI:1858306]
Tbx1	1.38	8.1E-04	T-box 1 [Source:MGI Symbol;Acc:MGI:98493]
G0s2	1.38	1.3E-03	G0/G1 switch gene 2 [Source:MGI Symbol;Acc:MGI:1316737]
Lypd6	1.38	1.5E-03	LY6/PLAUR domain containing 6 [Source:MGI Symbol;Acc:MGI:2443848]
Rab6b	1.38	1.0E-02	RAB6B, member RAS oncogene family [Source:MGI Symbol;Acc:MGI:107283]
Jade2	1.38	7.4E-09	jade family PHD finger 2 [Source:MGI Symbol;Acc:MGI:1924151]
Bcl2a1b	1.38	7.1E-04	B cell leukemia/lymphoma 2 related protein A1b [Source:MGI Symbol;Acc:MGI:1278326]
Fzd4	1.37	3.0E-04	frizzled homolog 4 (Drosophila) [Source:MGI Symbol;Acc:MGI:108520]
Pex5l	1.37	2.0E-02	peroxisomal biogenesis factor 5-like [Source:MGI Symbol;Acc:MGI:1916672]
Cd248	1.37	3.5E-03	CD248 antigen, endosialin [Source:MGI Symbol;Acc:MGI:1917695]
Gprc5b	1.37	1.6E-03	G protein-coupled receptor, family C, group 5, member B [Source:MGI Symbol;Acc:MGI:1927596]
Shc2	1.37	2.7E-02	SHC (Src homology 2 domain containing) transforming protein 2 [Source:MGI Symbol;Acc:MGI:106180]
Unc79	1.37	1.2E-02	unc-79 homolog (C. elegans) [Source:MGI Symbol;Acc:MGI:2684729]
Agtr1a	1.37	8.7E-03	angiotensin II receptor, type 1a [Source:MGI Symbol;Acc:MGI:87964]
Dixdc1	1.37	2.3E-03	DIX domain containing 1 [Source:MGI Symbol;Acc:MGI:2679721]
Plscr4	1.37	3.4E-04	phospholipid scramblase 4 [Source:MGI Symbol;Acc:MGI:2143267]
Ppil6	1.37	2.4E-02	peptidylprolyl isomerase (cyclophilin)-like 6 [Source:MGI Symbol;Acc:MGI:1920325]
Pea15a	1.37	8.2E-10	phosphoprotein enriched in astrocytes 15A [Source:MGI Symbol;Acc:MGI:104799]
Serpinb8	1.37	7.8E-04	serine (or cysteine) peptidase inhibitor, clade B, member 8 [Source:MGI Symbol;Acc:MGI:894657]
Lin7a	1.37	1.8E-03	lin-7 homolog A (C. elegans) [Source:MGI Symbol;Acc:MGI:2135609]
Car11	1.37	5.3E-04	carbonic anhydrase 11 [Source:MGI Symbol;Acc:MGI:1336193]
Myocd	1.37	3.4E-02	myocardin [Source:MGI Symbol;Acc:MGI:2137495]
Meox2	1.37	1.3E-02	mesenchyme homeobox 2 [Source:MGI Symbol;Acc:MGI:103219]
Hoxd11	1.37	5.2E-04	homeobox D11 [Source:MGI Symbol;Acc:MGI:96203]
Lrfn1	1.37	6.9E-03	leucine rich repeat and fibronectin type III domain containing 1 [Source:MGI Symbol;Acc:MGI:2136810]
Sdk2	1.37	6.1E-03	sidekick homolog 2 (chicken) [Source:MGI Symbol;Acc:MGI:2443847]
Ahnak2	1.36	2.2E-04	AHNAK nucleoprotein 2 [Source:MGI Symbol;Acc:MGI:2144831]
Zeb2	1.36	3.3E-07	zinc finger E-box binding homeobox 2 [Source:MGI Symbol;Acc:MGI:1344407]
Chrna3	1.36	3.6E-02	cholinergic receptor, nicotinic, alpha polypeptide 3 [Source:MGI Symbol;Acc:MGI:87887]
Snx32	1.36	1.3E-03	sorting nexin 32 [Source:MGI Symbol;Acc:MGI:2444704]
Scg2	1.36	9.0E-03	secretogranin II [Source:MGI Symbol;Acc:MGI:103033]
Nell1	1.36	3.8E-02	NEL-like 1 [Source:MGI Symbol;Acc:MGI:2443902]
Fn3k	1.36	1.1E-02	fructosamine 3 kinase [Source:MGI Symbol;Acc:MGI:1926834]
Adcy5	1.36	6.8E-04	adenylate cyclase 5 [Source:MGI Symbol;Acc:MGI:99673]
Meis1	1.36	8.0E-04	Meis homeobox 1 [Source:MGI Symbol;Acc:MGI:104717]
Retnla	1.36	2.3E-03	resistin like alpha [Source:MGI Symbol;Acc:MGI:1888504]
Sned1	1.36	4.4E-06	sushi, nidogen and EGF-like domains 1 [Source:MGI Symbol;Acc:MGI:3045960]
Ina	1.36	3.7E-02	internexin neuronal intermediate filament protein, alpha [Source:MGI Symbol;Acc:MGI:96568]
Ntn1	1.36	1.1E-03	netrin 1 [Source:MGI Symbol;Acc:MGI:105088]
Unc119	1.36	1.6E-09	unc-119 homolog (C. elegans) [Source:MGI Symbol;Acc:MGI:1328357]
Zfhx4	1.36	2.4E-02	zinc finger homeodomain 4 [Source:MGI Symbol;Acc:MGI:2137668]
Iglon5	1.36	4.0E-02	IgLON family member 5 [Source:MGI Symbol;Acc:MGI:2686277]
Tagap	1.36	9.4E-05	T cell activation Rho GTPase activating protein [Source:MGI Symbol;Acc:MGI:3615484]
AF251705	1.35	2.0E-04	cDNA sequence AF251705 [Source:MGI Symbol;Acc:MGI:2153249]
Pecam1	1.35	1.2E-09	platelet/endothelial cell adhesion molecule 1 [Source:MGI Symbol;Acc:MGI:97537]
Tmem178b	1.35	6.3E-03	transmembrane protein 178B [Source:MGI Symbol;Acc:MGI:3647581]
Negr1	1.35	5.4E-03	neuronal growth regulator 1 [Source:MGI Symbol;Acc:MGI:2444846]
Adamts2	1.35	1.9E-03	a disintegrin-like and metallopeptidase (reprolysin type) with thrombospondin type 1 motif, 2 [Source:MGI Symbol;Acc:MGI:1347356]
Bmper	1.35	4.9E-04	BMP-binding endothelial regulator [Source:MGI Symbol;Acc:MGI:1920480]
Plagl1	1.35	2.1E-03	pleiomorphic adenoma gene-like 1 [Source:MGI Symbol;Acc:MGI:1100874]
Plcl1	1.35	8.3E-03	phospholipase C-like 1 [Source:MGI Symbol;Acc:MGI:3036262]
Hand1	1.35	3.1E-02	heart and neural crest derivatives expressed transcript 1 [Source:MGI Symbol;Acc:MGI:103577]
Pcdha10	1.35	1.3E-02	protocadherin alpha 10 [Source:MGI Symbol;Acc:MGI:1298408]
Dse	1.35	3.4E-08	dermatan sulfate epimerase [Source:MGI Symbol;Acc:MGI:2443455]
Igsf11	1.35	1.7E-02	immunoglobulin superfamily, member 11 [Source:MGI Symbol;Acc:MGI:2388477]

Tef	1.35	2.3E-03	thyrotroph embryonic factor [Source:MGI Symbol;Acc:MGI:98663]
Tmtc4	1.35	3.4E-12	transmembrane and tetratricopeptide repeat containing 4 [Source:MGI Symbol;Acc:MGI:1921050]
St6galnac5	1.35	4.6E-03	ST6 (alpha-N-acetyl-neuraminyl-2,3-beta-galactosyl-1,3)-N-acetylgalactosaminide alpha-2,6-sialyltransferase 5 [Source:MGI Symbol;Acc:MGI:1349471]
Cdh13	1.34	3.6E-06	cadherin 13 [Source:MGI Symbol;Acc:MGI:99551]
Atp13a2	1.34	4.7E-13	ATPase type 13A2 [Source:MGI Symbol;Acc:MGI:1922022]
Ndn	1.34	3.6E-03	necdin [Source:MGI Symbol;Acc:MGI:97290]
Akap12	1.34	9.7E-04	A kinase (PRKA) anchor protein (gravin) 12 [Source:MGI Symbol;Acc:MGI:1932576]
Map7d2	1.34	4.5E-02	MAP7 domain containing 2 [Source:MGI Symbol;Acc:MGI:1917474]
Tmem119	1.34	1.9E-04	transmembrane protein 119 [Source:MGI Symbol;Acc:MGI:2385228]
Tdrkh	1.34	3.4E-03	tudor and KH domain containing protein [Source:MGI Symbol;Acc:MGI:1919884]
Sncb	1.34	4.3E-02	synuclein, beta [Source:MGI Symbol;Acc:MGI:1889011]
Camkk2	1.34	4.9E-08	calcium/calmodulin-dependent protein kinase kinase 2, beta [Source:MGI Symbol;Acc:MGI:2444812]
Irs1	1.34	1.5E-03	insulin receptor substrate 1 [Source:MGI Symbol;Acc:MGI:99454]
Itsn1	1.34	8.3E-15	intersectin 1 (SH3 domain protein 1A) [Source:MGI Symbol;Acc:MGI:1338069]
Gprasp2	1.34	1.1E-02	G protein-coupled receptor associated sorting protein 2 [Source:MGI Symbol;Acc:MGI:2442071]
Sez6l	1.34	3.2E-02	seizure related 6 homolog like [Source:MGI Symbol;Acc:MGI:1935121]
Hmcn1	1.34	4.1E-03	hemicentin 1 [Source:MGI Symbol;Acc:MGI:2685047]
Sptb	1.34	3.5E-03	spectrin beta, erythrocytic [Source:MGI Symbol;Acc:MGI:98387]
Mapk10	1.33	4.7E-02	mitogen-activated protein kinase 10 [Source:MGI Symbol;Acc:MGI:1346863]
Grik2	1.33	4.0E-02	glutamate receptor, ionotropic, kainate 2 (beta 2) [Source:MGI Symbol;Acc:MGI:95815]
Rasgrp1	1.33	1.4E-05	RAS guanyl releasing protein 1 [Source:MGI Symbol;Acc:MGI:1314635]
Faim2	1.33	4.8E-02	Fas apoptotic inhibitory molecule 2 [Source:MGI Symbol;Acc:MGI:1919643]
Tal1	1.33	5.3E-03	T cell acute lymphocytic leukemia 1 [Source:MGI Symbol;Acc:MGI:98480]
Dpysl5	1.33	3.0E-02	dihydropyrimidinase-like 5 [Source:MGI Symbol;Acc:MGI:1929772]
Robo2	1.33	5.1E-03	roundabout homolog 2 (Drosophila) [Source:MGI Symbol;Acc:MGI:1890110]
Akap6	1.33	4.5E-03	A kinase (PRKA) anchor protein 6 [Source:MGI Symbol;Acc:MGI:3050566]
Ccdc8	1.33	1.6E-03	coiled-coil domain containing 8 [Source:MGI Symbol;Acc:MGI:3612184]
Ctxn3	1.33	6.0E-03	cortexin 3 [Source:MGI Symbol;Acc:MGI:3642816]
Gfap	1.33	3.7E-02	glial fibrillary acidic protein [Source:MGI Symbol;Acc:MGI:95697]
Piezo2	1.33	5.8E-04	piezo-type mechanosensitive ion channel component 2 [Source:MGI Symbol;Acc:MGI:1918781]
Pla2g15	1.33	2.4E-06	phospholipase A2, group XV [Source:MGI Symbol;Acc:MGI:2178076]
Trpm6	1.32	1.9E-06	transient receptor potential cation channel, subfamily M, member 6 [Source:MGI Symbol;Acc:MGI:2675603]
P2rx2	1.32	1.3E-02	purinergic receptor P2X, ligand-gated ion channel, 2 [Source:MGI Symbol;Acc:MGI:2665170]
Ugt1a1	1.32	4.6E-02	UDP glucuronosyltransferase 1 family, polypeptide A1 [Source:MGI Symbol;Acc:MGI:98898]
Lrg1	1.32	1.5E-02	leucine-rich alpha-2-glycoprotein 1 [Source:MGI Symbol;Acc:MGI:1924155]
Actc1	1.32	6.1E-03	actin, alpha, cardiac muscle 1 [Source:MGI Symbol;Acc:MGI:87905]
4632428N05Rik	1.32	1.1E-10	RIKEN cDNA 4632428N05 gene [Source:MGI Symbol;Acc:MGI:1921298]
Itga11	1.32	2.8E-04	integrin alpha 11 [Source:MGI Symbol;Acc:MGI:2442114]
Spin4	1.32	1.3E-02	spindlin family, member 4 [Source:MGI Symbol;Acc:MGI:2444925]
Gap43	1.32	1.3E-02	growth associated protein 43 [Source:MGI Symbol;Acc:MGI:95639]
Pamr1	1.32	9.4E-04	peptidase domain containing associated with muscle regeneration 1 [Source:MGI Symbol;Acc:MGI:2445082]
Npy2r	1.32	2.2E-02	neuropeptide Y receptor Y2 [Source:MGI Symbol;Acc:MGI:108418]
Elmod1	1.32	1.4E-02	ELMO/CED-12 domain containing 1 [Source:MGI Symbol;Acc:MGI:3583900]
Gatsl2	1.32	2.7E-08	GATS protein-like 2 [Source:MGI Symbol;Acc:MGI:1933384]
Cyp2c55	1.32	2.4E-02	cytochrome P450, family 2, subfamily c, polypeptide 55 [Source:MGI Symbol;Acc:MGI:1919332]
Dpp10	1.32	8.3E-03	dipeptidylpeptidase 10 [Source:MGI Symbol;Acc:MGI:2442409]
Pcmtd1	1.31	8.9E-15	protein-L-isoaspartate (D-aspartate) O-methyltransferase domain containing 1 [Source:MGI Symbol;Acc:MGI:2441773]
Kazald1	1.31	5.4E-03	Kazal-type serine peptidase inhibitor domain 1 [Source:MGI Symbol;Acc:MGI:2147606]
Adrb1	1.31	2.0E-02	adrenergic receptor, beta 1 [Source:MGI Symbol;Acc:MGI:87937]
Fndc1	1.31	8.1E-04	fibronectin type III domain containing 1 [Source:MGI Symbol;Acc:MGI:1915905]
March1	1.31	9.4E-05	membrane-associated ring finger (C3HC4) 1 [Source:MGI Symbol;Acc:MGI:1920175]

Npy1r	1.31	2.9E-02	neuropeptide Y receptor Y1 [Source:MGI Symbol;Acc:MGI:104963]
Olfml2a	1.31	1.5E-03	olfactomedin-like 2A [Source:MGI Symbol;Acc:MGI:2444741]
Cxx1c	1.31	1.0E-04	CAAX box 1C [Source:MGI Symbol;Acc:MGI:1920115]
Mpdz	1.31	6.2E-04	multiple PDZ domain protein [Source:MGI Symbol;Acc:MGI:1343489]
Loxl3	1.31	2.1E-03	lysyl oxidase-like 3 [Source:MGI Symbol;Acc:MGI:1337004]
Rnf207	1.31	2.2E-02	ring finger protein 207 [Source:MGI Symbol;Acc:MGI:2684989]
Dnah10	1.31	8.9E-03	dynein, axonemal, heavy chain 10 [Source:MGI Symbol;Acc:MGI:1860299]
Ank2	1.31	3.5E-03	ankyrin 2, brain [Source:MGI Symbol;Acc:MGI:88025]
Dact3	1.31	2.8E-03	dapper homolog 3, antagonist of beta-catenin (xenopus) [Source:MGI Symbol;Acc:MGI:3654828]
Cldn5	1.31	7.7E-05	claudin 5 [Source:MGI Symbol;Acc:MGI:1276112]
Mir143hg	1.31	4.3E-02	Mir143 and Mir145 host gene (non-coding RNA) [Source:MGI Symbol;Acc:MGI:4439832]
Gpr133	1.31	7.2E-06	G protein-coupled receptor 133 [Source:MGI Symbol;Acc:MGI:3041203]
Ccdc60	1.31	2.7E-02	coiled-coil domain containing 60 [Source:MGI Symbol;Acc:MGI:2141043]
Rab3il1	1.31	5.2E-04	RAB3A interacting protein (rabin3)-like 1 [Source:MGI Symbol;Acc:MGI:1922010]
Sncg	1.31	1.0E-02	synuclein, gamma [Source:MGI Symbol;Acc:MGI:1298397]
Epha7	1.30	2.7E-02	Eph receptor A7 [Source:MGI Symbol;Acc:MGI:95276]
Rgma	1.30	3.0E-03	repulsive guidance molecule family member A [Source:MGI Symbol;Acc:MGI:2679262]
Ebf4	1.30	2.4E-02	early B cell factor 4 [Source:MGI Symbol;Acc:MGI:2385972]
Gna15	1.30	5.0E-04	guanine nucleotide binding protein, alpha 15 [Source:MGI Symbol;Acc:MGI:95770]
Clip3	1.30	7.9E-04	CAP-GLY domain containing linker protein 3 [Source:MGI Symbol;Acc:MGI:1923936]
Cyp2d22	1.30	1.3E-05	cytochrome P450, family 2, subfamily d, polypeptide 22 [Source:MGI Symbol;Acc:MGI:1929474]
Mapk8ip2	1.30	3.3E-02	mitogen-activated protein kinase 8 interacting protein 2 [Source:MGI Symbol;Acc:MGI:1926555]
Boc	1.30	5.0E-04	biregional cell adhesion molecule-related/down-regulated by oncogenes (Cdon) binding protein [Source:MGI Symbol;Acc:MGI:2151153]
Sstr1	1.30	2.2E-02	somatostatin receptor 1 [Source:MGI Symbol;Acc:MGI:98327]
Timp4	1.30	4.0E-02	tissue inhibitor of metalloproteinase 4 [Source:MGI Symbol;Acc:MGI:109125]
Foxd3	1.29	1.4E-02	forkhead box D3 [Source:MGI Symbol;Acc:MGI:1347473]
Fam198b	1.29	1.7E-05	family with sequence similarity 198, member B [Source:MGI Symbol;Acc:MGI:1915909]
Myh11	1.29	2.0E-02	myosin, heavy polypeptide 11, smooth muscle [Source:MGI Symbol;Acc:MGI:102643]
Kif5a	1.29	2.1E-02	kinesin family member 5A [Source:MGI Symbol;Acc:MGI:109564]
Otud1	1.29	3.4E-04	OTU domain containing 1 [Source:MGI Symbol;Acc:MGI:1918448]
Jam2	1.29	5.7E-05	junction adhesion molecule 2 [Source:MGI Symbol;Acc:MGI:1933820]
Dlc1	1.29	5.8E-07	deleted in liver cancer 1 [Source:MGI Symbol;Acc:MGI:1354949]
Syt1	1.29	1.8E-02	synaptotagmin I [Source:MGI Symbol;Acc:MGI:99667]
Pcbp3	1.29	5.8E-03	poly(rC) binding protein 3 [Source:MGI Symbol;Acc:MGI:1890470]
Rassf8	1.29	1.7E-03	Ras association (RalGDS/AF-6) domain family (N-terminal) member 8 [Source:MGI Symbol;Acc:MGI:1918573]
Sdpr	1.29	6.2E-05	serum deprivation response [Source:MGI Symbol;Acc:MGI:99513]
Nptx2	1.29	3.5E-03	neuronal pentraxin 2 [Source:MGI Symbol;Acc:MGI:1858209]
Adamts5	1.29	8.0E-03	a disintegrin-like and metallopeptidase (reprolysin type) with thrombospondin type 1 motif, 5 (aggrecanase-2) [Source:MGI Symbol;Acc:MGI:1346321]
Pcp4l1	1.28	5.5E-03	Purkinje cell protein 4-like 1 [Source:MGI Symbol;Acc:MGI:1913675]
Ptpdc1	1.28	9.0E-03	protein tyrosine phosphatase domain containing 1 [Source:MGI Symbol;Acc:MGI:2145430]
Zc3h12c	1.28	2.7E-03	zinc finger CCCH type containing 12C [Source:MGI Symbol;Acc:MGI:3026959]
Gria3	1.28	8.7E-03	glutamate receptor, ionotropic, AMPA3 (alpha 3) [Source:MGI Symbol;Acc:MGI:95810]
Kcnip3	1.28	3.7E-03	Kv channel interacting protein 3, calsenilin [Source:MGI Symbol;Acc:MGI:1929258]
Lamb2	1.28	5.9E-04	laminin, beta 2 [Source:MGI Symbol;Acc:MGI:99916]
Pdgfrl	1.28	2.7E-02	platelet-derived growth factor receptor-like [Source:MGI Symbol;Acc:MGI:1916047]
Tlr7	1.28	2.3E-03	toll-like receptor 7 [Source:MGI Symbol;Acc:MGI:2176882]
Ptprn	1.28	1.9E-02	protein tyrosine phosphatase, receptor type, N [Source:MGI Symbol;Acc:MGI:102765]
Hnmt	1.28	4.5E-03	histamine N-methyltransferase [Source:MGI Symbol;Acc:MGI:2153181]
Acsl6	1.28	4.6E-02	acyl-CoA synthetase long-chain family member 6 [Source:MGI Symbol;Acc:MGI:894291]
Xylt2	1.28	1.1E-09	xylosyltransferase II [Source:MGI Symbol;Acc:MGI:2444797]
Olfm1	1.28	1.3E-05	olfactomedin 1 [Source:MGI Symbol;Acc:MGI:1860437]

Ncam1	1.28	4.4E-03	neural cell adhesion molecule 1 [Source:MGI Symbol;Acc:MGI:97281]
Efcc1	1.28	3.9E-04	EF hand and coiled-coil domain containing 1 [Source:MGI Symbol;Acc:MGI:3611451]
Tspan18	1.28	5.8E-03	tetraspanin 18 [Source:MGI Symbol;Acc:MGI:1917186]
Sox10	1.28	3.7E-03	SRY (sex determining region Y)-box 10 [Source:MGI Symbol;Acc:MGI:98358]
Ntn4	1.28	1.6E-04	netrin 4 [Source:MGI Symbol;Acc:MGI:1888978]
Lrrtm1	1.28	2.0E-02	leucine rich repeat transmembrane neuronal 1 [Source:MGI Symbol;Acc:MGI:2389173]
Slc35f1	1.28	1.6E-02	solute carrier family 35, member F1 [Source:MGI Symbol;Acc:MGI:2139810]
Arhgef4	1.28	1.1E-02	Rho guanine nucleotide exchange factor (GEF) 4 [Source:MGI Symbol;Acc:MGI:2442507]
Ntn3	1.28	2.9E-02	netrin 3 [Source:MGI Symbol;Acc:MGI:1341188]
6330403A02Rik	1.28	3.5E-03	RIKEN cDNA 6330403A02 gene [Source:MGI Symbol;Acc:MGI:2138735]
Wscd1	1.28	5.6E-03	WSC domain containing 1 [Source:MGI Symbol;Acc:MGI:2448493]
Scn5a	1.27	4.9E-02	sodium channel, voltage-gated, type V, alpha [Source:MGI Symbol;Acc:MGI:98251]
Ryr3	1.27	7.2E-03	ryanodine receptor 3 [Source:MGI Symbol;Acc:MGI:99684]
Sardh	1.27	5.5E-03	sarcosine dehydrogenase [Source:MGI Symbol;Acc:MGI:2183102]
Lmod1	1.27	3.3E-03	leiomodin 1 (smooth muscle) [Source:MGI Symbol;Acc:MGI:2135671]
Ptpru	1.27	2.7E-04	protein tyrosine phosphatase, receptor type, U [Source:MGI Symbol;Acc:MGI:1321151]
Hpse2	1.27	2.9E-02	heparanase 2 [Source:MGI Symbol;Acc:MGI:2685814]
Ttc28	1.27	9.9E-06	tetratricopeptide repeat domain 28 [Source:MGI Symbol;Acc:MGI:2140873]
Dcn	1.27	2.2E-05	decorin [Source:MGI Symbol;Acc:MGI:94872]
Tmem26	1.27	2.4E-02	transmembrane protein 26 [Source:MGI Symbol;Acc:MGI:2143537]
Gfra2	1.27	3.0E-03	glial cell line derived neurotrophic factor family receptor alpha 2 [Source:MGI Symbol;Acc:MGI:1195462]
Comp	1.27	6.2E-03	cartilage oligomeric matrix protein [Source:MGI Symbol;Acc:MGI:88469]
Cplx1	1.27	2.6E-02	complexin 1 [Source:MGI Symbol;Acc:MGI:104727]
Scg3	1.27	3.3E-03	secretogranin III [Source:MGI Symbol;Acc:MGI:103032]
Ly9	1.27	4.1E-05	lymphocyte antigen 9 [Source:MGI Symbol;Acc:MGI:96885]
Fam227a	1.27	2.3E-02	family with sequence similarity 227, member A [Source:MGI Symbol;Acc:MGI:1922979]
Coch	1.27	1.2E-02	coagulation factor C homolog (Limulus polyphemus) [Source:MGI Symbol;Acc:MGI:1278313]
Egflam	1.27	2.7E-03	EGF-like, fibronectin type III and laminin G domains [Source:MGI Symbol;Acc:MGI:2146149]
Rasgef1c	1.27	2.2E-02	RasGEF domain family, member 1C [Source:MGI Symbol;Acc:MGI:1921813]
Cd300ld	1.26	7.4E-03	CD300 molecule-like family member d [Source:MGI Symbol;Acc:MGI:2442358]
Tnfaip6	1.26	2.0E-02	tumor necrosis factor alpha induced protein 6 [Source:MGI Symbol;Acc:MGI:1195266]
Slc24a3	1.26	6.8E-03	solute carrier family 24 (sodium/potassium/calcium exchanger), member 3 [Source:MGI Symbol;Acc:MGI:2137513]
Slc36a4	1.26	1.2E-05	solute carrier family 36 (proton/amino acid symporter), member 4 [Source:MGI Symbol;Acc:MGI:2442595]
Slc2a4	1.26	2.0E-03	solute carrier family 2 (facilitated glucose transporter), member 4 [Source:MGI Symbol;Acc:MGI:95758]
Xkr8	1.26	5.7E-04	X Kell blood group precursor related family member 8 homolog [Source:MGI Symbol;Acc:MGI:2685877]
Fibin	1.26	4.9E-02	fin bud initiation factor homolog (zebrafish) [Source:MGI Symbol;Acc:MGI:1914856]
Arhgap15	1.26	7.7E-04	Rho GTPase activating protein 15 [Source:MGI Symbol;Acc:MGI:1923367]
Wasf3	1.26	1.3E-02	WAS protein family, member 3 [Source:MGI Symbol;Acc:MGI:2658986]
Fam155a	1.26	1.1E-02	family with sequence similarity 155, member A [Source:MGI Symbol;Acc:MGI:2142765]
Cacna1h	1.26	2.3E-06	calcium channel, voltage-dependent, T type, alpha 1H subunit [Source:MGI Symbol;Acc:MGI:1928842]
Tcp11l1	1.26	8.4E-03	t-complex 11 like 1 [Source:MGI Symbol;Acc:MGI:2444263]
Vnn3	1.26	2.1E-02	vanin 3 [Source:MGI Symbol;Acc:MGI:1347055]
Gnai1	1.26	2.4E-06	guanine nucleotide binding protein (G protein), alpha inhibiting 1 [Source:MGI Symbol;Acc:MGI:95771]
S100b	1.26	1.3E-02	S100 protein, beta polypeptide, neural [Source:MGI Symbol;Acc:MGI:98217]
Etnppl	1.26	4.6E-02	ethanolamine phosphate phospholyase [Source:MGI Symbol;Acc:MGI:1919010]
Tbxa2r	1.26	9.8E-03	thromboxane A2 receptor [Source:MGI Symbol;Acc:MGI:98496]
Egf	1.25	3.8E-02	epidermal growth factor [Source:MGI Symbol;Acc:MGI:95290]
Lair1	1.25	3.1E-05	leukocyte-associated Ig-like receptor 1 [Source:MGI Symbol;Acc:MGI:105492]

8430408G22Rik	1.25	4.7E-02	RIKEN cDNA 8430408G22 gene [Source:MGI Symbol;Acc:MGI:1918730]
Map6	1.25	4.2E-03	microtubule-associated protein 6 [Source:MGI Symbol;Acc:MGI:1201690]
Adora1	1.25	4.6E-03	adenosine A1 receptor [Source:MGI Symbol;Acc:MGI:99401]
Tceal1	1.25	2.2E-03	transcription elongation factor A (SII)-like 1 [Source:MGI Symbol;Acc:MGI:2385317]
Ttc8	1.25	3.7E-03	tetratricopeptide repeat domain 8 [Source:MGI Symbol;Acc:MGI:1923510]
Aldh1a2	1.25	8.2E-03	aldehyde dehydrogenase family 1, subfamily A2 [Source:MGI Symbol;Acc:MGI:107928]
Tmem132c	1.25	4.0E-02	transmembrane protein 132C [Source:MGI Symbol;Acc:MGI:2443061]
Lhx6	1.25	2.0E-02	LIM homeobox protein 6 [Source:MGI Symbol;Acc:MGI:1306803]
Slc22a17	1.25	2.7E-03	solute carrier family 22 (organic cation transporter), member 17 [Source:MGI Symbol;Acc:MGI:1926225]
Samd4	1.25	7.1E-03	sterile alpha motif domain containing 4 [Source:MGI Symbol;Acc:MGI:1921730]
Kctd15	1.25	1.7E-03	potassium channel tetramerisation domain containing 15 [Source:MGI Symbol;Acc:MGI:2385276]
Tspan2	1.25	6.8E-04	tetraspanin 2 [Source:MGI Symbol;Acc:MGI:1917997]
Cercam	1.24	2.1E-02	cerebral endothelial cell adhesion molecule [Source:MGI Symbol;Acc:MGI:2139134]
Peli3	1.24	6.9E-03	pellino 3 [Source:MGI Symbol;Acc:MGI:1924963]
Flna	1.24	5.1E-03	filamin, alpha [Source:MGI Symbol;Acc:MGI:95556]
Cacna1e	1.24	5.9E-03	calcium channel, voltage-dependent, R type, alpha 1E subunit [Source:MGI Symbol;Acc:MGI:106217]
Pvrl4	1.24	1.9E-03	poliovirus receptor-related 4 [Source:MGI Symbol;Acc:MGI:1918990]
Acp5	1.24	3.7E-07	acid phosphatase 5, tartrate resistant [Source:MGI Symbol;Acc:MGI:87883]
Arhgef6	1.24	5.0E-07	Rac/Cdc42 guanine nucleotide exchange factor (GEF) 6 [Source:MGI Symbol;Acc:MGI:1920591]
Ttn	1.24	3.9E-02	titin [Source:MGI Symbol;Acc:MGI:98864]
Hsd3b3	1.24	3.2E-03	hydroxy-delta-5-steroid dehydrogenase, 3 beta- and steroid delta-isomerase 3 [Source:MGI Symbol;Acc:MGI:96235]
Tex15	1.24	2.3E-02	testis expressed gene 15 [Source:MGI Symbol;Acc:MGI:1934816]
Rtn1	1.24	5.5E-03	reticulon 1 [Source:MGI Symbol;Acc:MGI:1933947]
M1ap	1.24	2.9E-02	meiosis 1 associated protein [Source:MGI Symbol;Acc:MGI:1315200]
Myo1g	1.24	1.4E-09	myosin IG [Source:MGI Symbol;Acc:MGI:1927091]
Eno2	1.24	4.8E-03	enolase 2, gamma neuronal [Source:MGI Symbol;Acc:MGI:95394]
Arhgef10	1.24	3.7E-04	Rho guanine nucleotide exchange factor (GEF) 10 [Source:MGI Symbol;Acc:MGI:2444453]
Slc8a2	1.24	3.4E-02	solute carrier family 8 (sodium/calcium exchanger), member 2 [Source:MGI Symbol;Acc:MGI:107996]
Chd5	1.24	2.2E-02	chromodomain helicase DNA binding protein 5 [Source:MGI Symbol;Acc:MGI:3036258]
Lims2	1.24	3.2E-03	LIM and senescent cell antigen like domains 2 [Source:MGI Symbol;Acc:MGI:2385067]
Synm	1.24	7.6E-03	synemin, intermediate filament protein [Source:MGI Symbol;Acc:MGI:2661187]
Adamtsl2	1.24	4.3E-02	ADAMTS-like 2 [Source:MGI Symbol;Acc:MGI:1925044]
Tmtc1	1.24	1.4E-03	transmembrane and tetratricopeptide repeat containing 1 [Source:MGI Symbol;Acc:MGI:3039590]
Ebf3	1.24	1.6E-02	early B cell factor 3 [Source:MGI Symbol;Acc:MGI:894289]
Zfp532	1.24	9.9E-05	zinc finger protein 532 [Source:MGI Symbol;Acc:MGI:3036282]
Hpgds	1.23	6.3E-03	hematopoietic prostaglandin D synthase [Source:MGI Symbol;Acc:MGI:1859384]
Setbp1	1.23	4.8E-03	SET binding protein 1 [Source:MGI Symbol;Acc:MGI:1933199]
Cul9	1.23	1.3E-04	cullin 9 [Source:MGI Symbol;Acc:MGI:1925559]
Kcnq4	1.23	1.4E-02	potassium voltage-gated channel, subfamily Q, member 4 [Source:MGI Symbol;Acc:MGI:1926803]
Cep83os	1.23	8.4E-03	centrosomal protein 83, opposite strand [Source:MGI Symbol;Acc:MGI:1914973]
Slc39a13	1.23	1.4E-06	solute carrier family 39 (metal ion transporter), member 13 [Source:MGI Symbol;Acc:MGI:1915677]
9230105E05Rik	1.23	1.8E-02	RIKEN cDNA 9230105E05 gene [Source:MGI Symbol;Acc:MGI:2444388]
Gramd1b	1.23	5.3E-06	GRAM domain containing 1B [Source:MGI Symbol;Acc:MGI:1925037]
Mkx	1.23	2.1E-04	mohawk homeobox [Source:MGI Symbol;Acc:MGI:2687286]
Sparcl1	1.23	2.4E-04	SPARC-like 1 [Source:MGI Symbol;Acc:MGI:108110]
Bai1	1.23	3.7E-02	brain-specific angiogenesis inhibitor 1 [Source:MGI Symbol;Acc:MGI:1933736]
Islr2	1.23	4.6E-02	immunoglobulin superfamily containing leucine-rich repeat 2 [Source:MGI Symbol;Acc:MGI:2444277]
Cd109	1.23	1.9E-03	CD109 antigen [Source:MGI Symbol;Acc:MGI:2445221]
Scrn1	1.23	1.3E-02	secernin 1 [Source:MGI Symbol;Acc:MGI:1917188]
Fbp1	1.23	2.3E-02	fructose bisphosphatase 1 [Source:MGI Symbol;Acc:MGI:95492]
Tgfb2	1.23	9.7E-03	transforming growth factor, beta 2 [Source:MGI Symbol;Acc:MGI:98726]

Pik3c2g	1.22	4.4E-02	phosphatidylinositol 3-kinase, C2 domain containing, gamma polypeptide [Source:MGI Symbol;Acc:MGI:1203730]
Cdh2	1.22	2.3E-02	cadherin 2 [Source:MGI Symbol;Acc:MGI:88355]
Kcnn2	1.22	4.9E-02	potassium intermediate/small conductance calcium-activated channel, subfamily N, member 2 [Source:MGI Symbol;Acc:MGI:2153182]
Ccdc88b	1.22	6.5E-09	coiled-coil domain containing 88B [Source:MGI Symbol;Acc:MGI:1925567]
Xlr	1.22	7.2E-03	X-linked lymphocyte-regulated [Source:MGI Symbol;Acc:MGI:98976]
Gpr124	1.22	1.6E-03	G protein-coupled receptor 124 [Source:MGI Symbol;Acc:MGI:1925810]
Fign	1.22	3.0E-02	fidgetin [Source:MGI Symbol;Acc:MGI:1890647]
Aoah	1.22	3.9E-02	acyloxyacyl hydrolase [Source:MGI Symbol;Acc:MGI:1350928]
Stmn2	1.22	1.4E-02	stathmin-like 2 [Source:MGI Symbol;Acc:MGI:98241]
Spock2	1.22	9.9E-03	sparc/osteonectin, cwcv and kazal-like domains proteoglycan 2 [Source:MGI Symbol;Acc:MGI:1891351]
Samsn1	1.22	1.5E-04	SAM domain, SH3 domain and nuclear localization signals, 1 [Source:MGI Symbol;Acc:MGI:1914992]
Speg	1.22	1.9E-03	SPEG complex locus [Source:MGI Symbol;Acc:MGI:109282]
Vav2	1.22	1.3E-03	vav 2 oncogene [Source:MGI Symbol;Acc:MGI:102718]
Cdkl1	1.22	3.2E-02	cyclin-dependent kinase-like 1 (CDC2-related kinase) [Source:MGI Symbol;Acc:MGI:1918341]
Cep170	1.22	2.0E-05	centrosomal protein 170 [Source:MGI Symbol;Acc:MGI:1918348]
Btnl9	1.21	4.3E-02	butyrophilin-like 9 [Source:MGI Symbol;Acc:MGI:2442439]
Ak1	1.21	2.7E-03	adenylate kinase 1 [Source:MGI Symbol;Acc:MGI:87977]
Abcg4	1.21	1.6E-02	ATP-binding cassette, sub-family G (WHITE), member 4 [Source:MGI Symbol;Acc:MGI:1890594]
Col4a6	1.21	9.8E-05	collagen, type IV, alpha 6 [Source:MGI Symbol;Acc:MGI:2152695]
Kcnh1	1.21	1.8E-02	potassium voltage-gated channel, subfamily H (eag-related), member 1 [Source:MGI Symbol;Acc:MGI:1341721]
Dnajc6	1.21	4.2E-02	DnaJ (Hsp40) homolog, subfamily C, member 6 [Source:MGI Symbol;Acc:MGI:1919935]
Dstyk	1.21	1.3E-07	dual serine/threonine and tyrosine protein kinase [Source:MGI Symbol;Acc:MGI:1925064]
Fez1	1.21	2.7E-02	fasciculation and elongation protein zeta 1 (zygin I) [Source:MGI Symbol;Acc:MGI:2670976]
Flnc	1.21	1.3E-02	filamin C, gamma [Source:MGI Symbol;Acc:MGI:95557]
Mfap2	1.21	1.2E-02	microfibrillar-associated protein 2 [Source:MGI Symbol;Acc:MGI:99559]
Cd53	1.21	7.1E-07	CD53 antigen [Source:MGI Symbol;Acc:MGI:88341]
Pnmal2	1.21	8.3E-04	PNMA-like 2 [Source:MGI Symbol;Acc:MGI:3645856]
Dact1	1.21	2.4E-03	dapper homolog 1, antagonist of beta-catenin (xenopus) [Source:MGI Symbol;Acc:MGI:1891740]
Ltbp4	1.21	8.4E-06	latent transforming growth factor beta binding protein 4 [Source:MGI Symbol;Acc:MGI:1321395]
Wnk1	1.21	5.9E-10	WNK lysine deficient protein kinase 1 [Source:MGI Symbol;Acc:MGI:2442092]
Npr1	1.21	9.5E-04	natriuretic peptide receptor 1 [Source:MGI Symbol;Acc:MGI:97371]
St3gal6	1.21	6.6E-03	ST3 beta-galactoside alpha-2,3-sialyltransferase 6 [Source:MGI Symbol;Acc:MGI:1888707]
Mrvi1	1.21	2.3E-03	MRV integration site 1 [Source:MGI Symbol;Acc:MGI:1338023]
Zfp618	1.21	2.7E-02	zinc finger protein 618 [Source:MGI Symbol;Acc:MGI:1919950]
Cgnl1	1.21	1.0E-04	cingulin-like 1 [Source:MGI Symbol;Acc:MGI:1915428]
Mamld1	1.21	9.2E-03	mastermind-like domain containing 1 [Source:MGI Symbol;Acc:MGI:3045303]
Mfap5	1.20	1.8E-02	microfibrillar associated protein 5 [Source:MGI Symbol;Acc:MGI:1354387]
Slc25a45	1.20	2.3E-05	solute carrier family 25, member 45 [Source:MGI Symbol;Acc:MGI:2147731]
Wscd2	1.20	3.5E-02	WSC domain containing 2 [Source:MGI Symbol;Acc:MGI:2445030]
Grik3	1.20	1.4E-03	glutamate receptor, ionotropic, kainate 3 [Source:MGI Symbol;Acc:MGI:95816]
Adam33	1.20	7.4E-03	a disintegrin and metallopeptidase domain 33 [Source:MGI Symbol;Acc:MGI:1341813]
Fam13c	1.20	3.8E-02	family with sequence similarity 13, member C [Source:MGI Symbol;Acc:MGI:1918971]
Gpr97	1.20	7.4E-03	G protein-coupled receptor 97 [Source:MGI Symbol;Acc:MGI:1859670]
Frem2	1.20	2.5E-02	Fras1 related extracellular matrix protein 2 [Source:MGI Symbol;Acc:MGI:2444465]
Snap25	1.20	3.8E-02	synaptosomal-associated protein 25 [Source:MGI Symbol;Acc:MGI:98331]
Nkd2	1.20	2.2E-02	naked cuticle 2 homolog (Drosophila) [Source:MGI Symbol;Acc:MGI:1919543]
Ccdc80	1.20	7.3E-03	coiled-coil domain containing 80 [Source:MGI Symbol;Acc:MGI:1915146]
Krt222	1.20	2.1E-03	keratin 222 [Source:MGI Symbol;Acc:MGI:2442728]
Aldh1a3	1.20	9.5E-04	aldehyde dehydrogenase family 1, subfamily A3 [Source:MGI Symbol;Acc:MGI:1861722]
Tgfbr3	1.20	3.4E-04	transforming growth factor, beta receptor III [Source:MGI Symbol;Acc:MGI:104637]

Map1a	1.20	2.6E-03	microtubule-associated protein 1 A [Source:MGI Symbol;Acc:MGI:1306776]
Cacna2d1	1.19	2.3E-03	calcium channel, voltage-dependent, alpha2/delta subunit 1 [Source:MGI Symbol;Acc:MGI:88295]
Dennd4b	1.19	3.0E-08	DENN/MADD domain containing 4B [Source:MGI Symbol;Acc:MGI:2446201]
Fpr2	1.19	9.9E-03	formyl peptide receptor 2 [Source:MGI Symbol;Acc:MGI:1278319]
Atp1a2	1.19	9.2E-04	ATPase, Na+/K+ transporting, alpha 2 polypeptide [Source:MGI Symbol;Acc:MGI:88106]
Zfp354c	1.19	8.2E-03	zinc finger protein 354C [Source:MGI Symbol;Acc:MGI:1353621]
Jph2	1.19	1.0E-02	junctophilin 2 [Source:MGI Symbol;Acc:MGI:1891496]
Gabra3	1.19	2.6E-02	gamma-aminobutyric acid (GABA) A receptor, subunit alpha 3 [Source:MGI Symbol;Acc:MGI:95615]
Dync2li1	1.19	1.1E-02	dynein cytoplasmic 2 light intermediate chain 1 [Source:MGI Symbol;Acc:MGI:1913996]
Shroom4	1.19	4.4E-03	shroom family member 4 [Source:MGI Symbol;Acc:MGI:2685570]
Susd4	1.19	9.2E-03	sushi domain containing 4 [Source:MGI Symbol;Acc:MGI:2138351]
Col23a1	1.19	1.9E-02	collagen, type XXIII, alpha 1 [Source:MGI Symbol;Acc:MGI:2653243]
Sema3a	1.19	2.0E-02	sema domain, immunoglobulin domain (Ig), short basic domain, secreted, (semaphorin) 3A [Source:MGI Symbol;Acc:MGI:107558]
Tbxas1	1.19	3.3E-05	thromboxane A synthase 1, platelet [Source:MGI Symbol;Acc:MGI:98497]
Tmem8b	1.19	1.9E-03	transmembrane protein 8B [Source:MGI Symbol;Acc:MGI:2441680]
Scgn	1.19	8.4E-03	secretagogin, EF-hand calcium binding protein [Source:MGI Symbol;Acc:MGI:2384873]
Cacna1c	1.19	4.1E-02	calcium channel, voltage-dependent, L type, alpha 1C subunit [Source:MGI Symbol;Acc:MGI:103013]
Anxa9	1.19	9.2E-03	annexin A9 [Source:MGI Symbol;Acc:MGI:1923711]
Myl9	1.19	1.2E-02	myosin, light polypeptide 9, regulatory [Source:MGI Symbol;Acc:MGI:2138915]
Ar	1.18	3.5E-03	androgen receptor [Source:MGI Symbol;Acc:MGI:88064]
A430105I19Rik	1.18	3.8E-04	RIKEN cDNA A430105I19 gene [Source:MGI Symbol;Acc:MGI:2685199]
Rian	1.18	1.1E-02	RNA imprinted and accumulated in nucleus [Source:MGI Symbol;Acc:MGI:1922995]
Ust	1.18	2.7E-03	uronyl-2-sulfotransferase [Source:MGI Symbol;Acc:MGI:2442406]
Rusc2	1.18	1.1E-04	RUN and SH3 domain containing 2 [Source:MGI Symbol;Acc:MGI:2140371]
Cd83	1.18	4.7E-05	CD83 antigen [Source:MGI Symbol;Acc:MGI:1328316]
Nfam1	1.18	5.2E-04	Nfat activating molecule with ITAM motif 1 [Source:MGI Symbol;Acc:MGI:1921289]
Bmp1	1.18	5.4E-05	bone morphogenetic protein 1 [Source:MGI Symbol;Acc:MGI:88176]
Rab3c	1.18	3.7E-02	RAB3C, member RAS oncogene family [Source:MGI Symbol;Acc:MGI:1914545]
Lama4	1.18	5.5E-04	laminin, alpha 4 [Source:MGI Symbol;Acc:MGI:109321]
Pdzrn3	1.18	3.8E-04	PDZ domain containing RING finger 3 [Source:MGI Symbol;Acc:MGI:1933157]
Pard6g	1.18	1.2E-03	par-6 family cell polarity regulator gamma [Source:MGI Symbol;Acc:MGI:2135606]
BC026585	1.18	9.8E-04	cDNA sequence BC026585 [Source:MGI Symbol;Acc:MGI:2448516]
Bbx	1.18	6.0E-08	bobby sox homolog (Drosophila) [Source:MGI Symbol;Acc:MGI:1917758]
Tgm3	1.18	2.9E-02	transglutaminase 3, E polypeptide [Source:MGI Symbol;Acc:MGI:98732]
Fgd5	1.18	3.5E-03	FYVE, RhoGEF and PH domain containing 5 [Source:MGI Symbol;Acc:MGI:2443369]
Msn	1.18	5.6E-07	moesin [Source:MGI Symbol;Acc:MGI:97167]
Serpinf1	1.18	5.8E-03	serine (or cysteine) peptidase inhibitor, clade F, member 1 [Source:MGI Symbol;Acc:MGI:108080]
Adamts1	1.18	2.9E-03	a disintegrin-like and metallopeptidase (reprolysin type) with thrombospondin type 1 motif, 1 [Source:MGI Symbol;Acc:MGI:109249]
Fbxl16	1.18	2.6E-02	F-box and leucine-rich repeat protein 16 [Source:MGI Symbol;Acc:MGI:2448488]
Sntg2	1.17	3.3E-02	syntrophin, gamma 2 [Source:MGI Symbol;Acc:MGI:1919541]
Sec14l1	1.17	3.5E-09	SEC14-like 1 (S. cerevisiae) [Source:MGI Symbol;Acc:MGI:1921386]
Tgfb3	1.17	1.6E-03	transforming growth factor, beta 3 [Source:MGI Symbol;Acc:MGI:98727]
Tmem255b	1.17	7.4E-03	transmembrane protein 255B [Source:MGI Symbol;Acc:MGI:2685533]
Itm2a	1.17	2.2E-02	integral membrane protein 2A [Source:MGI Symbol;Acc:MGI:107706]
Pde10a	1.17	3.9E-02	phosphodiesterase 10A [Source:MGI Symbol;Acc:MGI:1345143]
Cadm3	1.17	4.6E-03	cell adhesion molecule 3 [Source:MGI Symbol;Acc:MGI:2137858]
Fgfr1	1.17	3.3E-05	fibroblast growth factor receptor 1 [Source:MGI Symbol;Acc:MGI:95522]
Rgs7bp	1.17	2.4E-02	regulator of G-protein signalling 7 binding protein [Source:MGI Symbol;Acc:MGI:106334]
Dennd2a	1.17	3.7E-04	DENN/MADD domain containing 2A [Source:MGI Symbol;Acc:MGI:2444961]
Mxra8	1.17	3.0E-05	matrix-remodelling associated 8 [Source:MGI Symbol;Acc:MGI:1922011]
Stmn3	1.17	3.9E-02	stathmin-like 3 [Source:MGI Symbol;Acc:MGI:1277137]
Fam171a2	1.17	1.1E-02	family with sequence similarity 171, member A2 [Source:MGI Symbol;Acc:MGI:2448496]
Plp1	1.17	1.5E-03	proteolipid protein (myelin) 1 [Source:MGI Symbol;Acc:MGI:97623]

Smim1	1.17	2.6E-02	small integral membrane protein 1 [Source:MGI Symbol;Acc:MGI:1916109]
Gm11946	1.17	2.8E-02	predicted gene 11946 [Source:MGI Symbol;Acc:MGI:3650071]
Mgmt	1.17	1.2E-02	O-6-methylguanine-DNA methyltransferase [Source:MGI Symbol;Acc:MGI:96977]
Ndrg4	1.16	2.2E-02	N-myc downstream regulated gene 4 [Source:MGI Symbol;Acc:MGI:2384590]
Edn1	1.16	1.9E-02	endothelin 1 [Source:MGI Symbol;Acc:MGI:95283]
AI118078	1.16	5.3E-03	expressed sequence AI118078 [Source:MGI Symbol;Acc:MGI:2142980]
Napb	1.16	1.3E-02	N-ethylmaleimide sensitive fusion protein attachment protein beta [Source:MGI Symbol;Acc:MGI:104562]
Mab21l2	1.16	2.4E-03	mab-21-like 2 (C. elegans) [Source:MGI Symbol;Acc:MGI:1346022]
Gdap1l1	1.16	3.5E-02	ganglioside-induced differentiation-associated protein 1-like 1 [Source:MGI Symbol;Acc:MGI:2385163]
D430019H16Rik	1.16	3.4E-02	RIKEN cDNA D430019H16 gene [Source:MGI Symbol;Acc:MGI:2443127]
Rftn2	1.16	9.6E-04	raftlin family member 2 [Source:MGI Symbol;Acc:MGI:1921263]
Gm6377	1.16	5.2E-03	predicted gene 6377 [Source:MGI Symbol;Acc:MGI:3647255]
Kcnh2	1.16	2.3E-03	potassium voltage-gated channel, subfamily H (eag-related), member 2 [Source:MGI Symbol;Acc:MGI:1341722]
Plek	1.16	4.4E-06	pleckstrin [Source:MGI Symbol;Acc:MGI:1860485]
Mag	1.16	2.6E-02	myelin-associated glycoprotein [Source:MGI Symbol;Acc:MGI:96912]
Cpe	1.16	3.6E-03	carboxypeptidase E [Source:MGI Symbol;Acc:MGI:101932]
Ppm1l	1.16	3.0E-04	protein phosphatase 1 (formerly 2C)-like [Source:MGI Symbol;Acc:MGI:2139740]
Prph	1.16	3.9E-02	peripherin [Source:MGI Symbol;Acc:MGI:97774]
Was	1.16	2.3E-05	Wiskott-Aldrich syndrome homolog (human) [Source:MGI Symbol;Acc:MGI:105059]
Atp2b4	1.16	4.9E-03	ATPase, Ca++ transporting, plasma membrane 4 [Source:MGI Symbol;Acc:MGI:88111]
Nova2	1.16	6.3E-03	neuro-oncological ventral antigen 2 [Source:MGI Symbol;Acc:MGI:104296]
Cnn1	1.16	2.3E-02	calponin 1 [Source:MGI Symbol;Acc:MGI:104979]
Fcna	1.16	2.7E-02	ficolin A [Source:MGI Symbol;Acc:MGI:1340905]
Abcc9	1.16	2.6E-02	ATP-binding cassette, sub-family C (CFTR/MRP), member 9 [Source:MGI Symbol;Acc:MGI:1352630]
Ppp1r3c	1.15	2.3E-03	protein phosphatase 1, regulatory (inhibitor) subunit 3C [Source:MGI Symbol;Acc:MGI:1858229]
Pdlim3	1.15	4.9E-03	PDZ and LIM domain 3 [Source:MGI Symbol;Acc:MGI:1859274]
Fam131b	1.15	2.5E-02	family with sequence similarity 131, member B [Source:MGI Symbol;Acc:MGI:1923406]
N28178	1.15	3.1E-02	expressed sequence N28178 [Source:MGI Symbol;Acc:MGI:2140712]
Myrip	1.15	3.9E-03	myosin VIIA and Rab interacting protein [Source:MGI Symbol;Acc:MGI:2384407]
Mtss1l	1.15	9.2E-04	metastasis suppressor 1-like [Source:MGI Symbol;Acc:MGI:3039591]
Pkib	1.15	2.8E-05	protein kinase inhibitor beta, cAMP dependent, testis specific [Source:MGI Symbol;Acc:MGI:101937]
Themis2	1.15	1.9E-05	thymocyte selection associated family member 2 [Source:MGI Symbol;Acc:MGI:2446213]
Cacna2d3	1.15	4.8E-02	calcium channel, voltage-dependent, alpha2/delta subunit 3 [Source:MGI Symbol;Acc:MGI:1338890]
Syn2	1.15	1.8E-02	synapsin II [Source:MGI Symbol;Acc:MGI:103020]
Tmem44	1.15	8.8E-03	transmembrane protein 44 [Source:MGI Symbol;Acc:MGI:1924489]
Snhg11	1.15	4.5E-02	small nucleolar RNA host gene 11 [Source:MGI Symbol;Acc:MGI:2441845]
Ubxn11	1.15	1.1E-03	UBX domain protein 11 [Source:MGI Symbol;Acc:MGI:1914836]
Camkk1	1.15	1.0E-03	calcium/calmodulin-dependent protein kinase kinase 1, alpha [Source:MGI Symbol;Acc:MGI:1891766]
#N/A	1.15	3.6E-02	#N/A
Gramd1a	1.15	3.3E-09	GRAM domain containing 1A [Source:MGI Symbol;Acc:MGI:105490]
Npr3	1.15	6.6E-03	natriuretic peptide receptor 3 [Source:MGI Symbol;Acc:MGI:97373]
Nova1	1.15	2.1E-02	neuro-oncological ventral antigen 1 [Source:MGI Symbol;Acc:MGI:104297]
Sfxn4	1.15	2.5E-02	sideroflexin 4 [Source:MGI Symbol;Acc:MGI:2137680]
Per3	1.15	1.9E-02	period circadian clock 3 [Source:MGI Symbol;Acc:MGI:1277134]
Timp2	1.15	1.7E-05	tissue inhibitor of metalloproteinase 2 [Source:MGI Symbol;Acc:MGI:98753]
Fndc4	1.15	1.9E-02	fibronectin type III domain containing 4 [Source:MGI Symbol;Acc:MGI:1917195]
Nsg2	1.15	2.1E-02	neuron specific gene family member 2 [Source:MGI Symbol;Acc:MGI:1202070]
Gnao1	1.14	2.2E-03	guanine nucleotide binding protein, alpha O [Source:MGI Symbol;Acc:MGI:95775]
Ccdc50	1.14	6.5E-12	coiled-coil domain containing 50 [Source:MGI Symbol;Acc:MGI:1914751]
Ston1	1.14	5.7E-04	stonin 1 [Source:MGI Symbol;Acc:MGI:1924307]
Parvb	1.14	6.6E-04	parvin, beta [Source:MGI Symbol;Acc:MGI:2153063]
Agmo	1.14	2.5E-02	alkylglycerol monooxygenase [Source:MGI Symbol;Acc:MGI:2442495]
Cnksr3	1.14	9.8E-06	Cnksr family member 3 [Source:MGI Symbol;Acc:MGI:2674130]
Tll1	1.14	1.4E-02	tolloid-like [Source:MGI Symbol;Acc:MGI:106923]

AI429214	1.14	7.9E-03	expressed sequence AI429214 [Source:MGI Symbol;Acc:MGI:2142538]
Neurl1a	1.14	6.1E-03	neuralized homolog 1A (Drosophila) [Source:MGI Symbol;Acc:MGI:1334263]
Ptp4a3	1.14	1.2E-05	protein tyrosine phosphatase 4a3 [Source:MGI Symbol;Acc:MGI:1277098]
Rtn4rl2	1.14	2.9E-02	reticulon 4 receptor-like 2 [Source:MGI Symbol;Acc:MGI:2669796]
Dhdh	1.14	1.0E-03	dihydrodiol dehydrogenase (dimeric) [Source:MGI Symbol;Acc:MGI:1919005]
Ldhb	1.14	7.1E-03	lactate dehydrogenase B [Source:MGI Symbol;Acc:MGI:96763]
Syt3	1.14	4.0E-02	synaptotagmin III [Source:MGI Symbol;Acc:MGI:99665]
Itih5	1.14	5.5E-06	inter-alpha (globulin) inhibitor H5 [Source:MGI Symbol;Acc:MGI:1925751]
Arhgap24	1.14	1.0E-03	Rho GTPase activating protein 24 [Source:MGI Symbol;Acc:MGI:1922647]
Adam11	1.14	2.8E-03	a disintegrin and metallopeptidase domain 11 [Source:MGI Symbol;Acc:MGI:1098667]
Gulp1	1.14	2.2E-03	GULP, engulfment adaptor PTB domain containing 1 [Source:MGI Symbol;Acc:MGI:1920407]
Nxph3	1.13	5.0E-02	neurexophilin 3 [Source:MGI Symbol;Acc:MGI:1336188]
Dgkg	1.13	2.5E-02	diacylglycerol kinase, gamma [Source:MGI Symbol;Acc:MGI:105060]
Tubb4a	1.13	3.0E-02	tubulin, beta 4A class IVA [Source:MGI Symbol;Acc:MGI:107848]
Ackr2	1.13	2.4E-02	atypical chemokine receptor 2 [Source:MGI Symbol;Acc:MGI:1891697]
Aatk	1.13	1.8E-03	apoptosis-associated tyrosine kinase [Source:MGI Symbol;Acc:MGI:1197518]
P2rx1	1.13	7.5E-03	purinergic receptor P2X, ligand-gated ion channel, 1 [Source:MGI Symbol;Acc:MGI:1098235]
C77370	1.13	3.3E-02	expressed sequence C77370 [Source:MGI Symbol;Acc:MGI:2148050]
Nkd1	1.13	7.0E-03	naked cuticle 1 homolog (Drosophila) [Source:MGI Symbol;Acc:MGI:2135954]
Tmem35	1.13	2.3E-02	transmembrane protein 35 [Source:MGI Symbol;Acc:MGI:1914814]
Fam131a	1.13	1.2E-02	family with sequence similarity 131, member A [Source:MGI Symbol;Acc:MGI:1925658]
Dclk3	1.13	7.6E-03	doublecortin-like kinase 3 [Source:MGI Symbol;Acc:MGI:3039580]
Larp6	1.13	3.5E-03	La ribonucleoprotein domain family, member 6 [Source:MGI Symbol;Acc:MGI:1914807]
Car4	1.13	3.6E-02	carbonic anhydrase 4 [Source:MGI Symbol;Acc:MGI:1096574]
Gpr161	1.13	1.0E-02	G protein-coupled receptor 161 [Source:MGI Symbol;Acc:MGI:2685054]
Nlrp1b	1.13	1.7E-02	NLR family, pyrin domain containing 1B [Source:MGI Symbol;Acc:MGI:3582959]
Sh3bp1	1.13	6.3E-09	SH3-domain binding protein 1 [Source:MGI Symbol;Acc:MGI:104603]
Eva1c	1.13	3.2E-02	eva-1 homolog C (C. elegans) [Source:MGI Symbol;Acc:MGI:1918217]
Slc8a3	1.13	3.5E-02	solute carrier family 8 (sodium/calcium exchanger), member 3 [Source:MGI Symbol;Acc:MGI:107976]
Frmd6	1.12	1.3E-04	FERM domain containing 6 [Source:MGI Symbol;Acc:MGI:2442579]
Postn	1.12	4.9E-04	periostin, osteoblast specific factor [Source:MGI Symbol;Acc:MGI:1926321]
Prr5l	1.12	4.2E-03	proline rich 5 like [Source:MGI Symbol;Acc:MGI:1919696]
Aadac	1.12	4.2E-03	arylacetamide deacetylase (esterase) [Source:MGI Symbol;Acc:MGI:1915008]
Plekhb1	1.12	2.0E-02	pleckstrin homology domain containing, family B (evectins) member 1 [Source:MGI Symbol;Acc:MGI:1351469]
Fbln2	1.12	1.4E-03	fibulin 2 [Source:MGI Symbol;Acc:MGI:95488]
Cd37	1.12	2.8E-05	CD37 antigen [Source:MGI Symbol;Acc:MGI:88330]
Lrp3	1.12	8.9E-03	low density lipoprotein receptor-related protein 3 [Source:MGI Symbol;Acc:MGI:3584516]
Sh3bgr	1.12	1.5E-02	SH3-binding domain glutamic acid-rich protein [Source:MGI Symbol;Acc:MGI:1354740]
P4htm	1.12	7.5E-03	prolyl 4-hydroxylase, transmembrane (endoplasmic reticulum) [Source:MGI Symbol;Acc:MGI:1921693]
Dmpk	1.12	3.4E-03	dystrophia myotonica-protein kinase [Source:MGI Symbol;Acc:MGI:94906]
C130050O18Rik	1.12	7.2E-03	RIKEN cDNA C130050O18 gene [Source:MGI Symbol;Acc:MGI:2442694]
Nat8l	1.12	2.9E-02	N-acetyltransferase 8-like [Source:MGI Symbol;Acc:MGI:2447776]
Greb1	1.12	2.1E-04	gene regulated by estrogen in breast cancer protein [Source:MGI Symbol;Acc:MGI:2149712]
Ebf1	1.12	6.1E-04	early B cell factor 1 [Source:MGI Symbol;Acc:MGI:95275]
Tubb3	1.12	4.6E-02	tubulin, beta 3 class III [Source:MGI Symbol;Acc:MGI:107813]
Bex2	1.12	1.9E-02	brain expressed X-linked 2 [Source:MGI Symbol;Acc:MGI:1338017]
Tnfrsf19	1.12	1.4E-02	tumor necrosis factor receptor superfamily, member 19 [Source:MGI Symbol;Acc:MGI:1352474]
Spock3	1.12	3.1E-02	sparc/osteonectin, cwcv and kazal-like domains proteoglycan 3 [Source:MGI Symbol;Acc:MGI:1920152]
Olfml1	1.12	3.7E-02	olfactomedin-like 1 [Source:MGI Symbol;Acc:MGI:2679264]
Acta2	1.12	3.4E-02	actin, alpha 2, smooth muscle, aorta [Source:MGI Symbol;Acc:MGI:87909]
Myo5a	1.11	1.9E-06	myosin VA [Source:MGI Symbol;Acc:MGI:105976]
Gpr20	1.11	1.8E-02	G protein-coupled receptor 20 [Source:MGI Symbol;Acc:MGI:2441803]
D630045J12Rik	1.11	1.9E-02	RIKEN cDNA D630045J12 gene [Source:MGI Symbol;Acc:MGI:2669829]

Meg3	1.11	1.3E-02	maternally expressed 3 [Source:MGI Symbol;Acc:MGI:1202886]
Aspn	1.11	2.8E-03	asporin [Source:MGI Symbol;Acc:MGI:1913945]
Syngap1	1.11	3.7E-03	synaptic Ras GTPase activating protein 1 homolog (rat) [Source:MGI Symbol;Acc:MGI:3039785]
Eml1	1.11	1.0E-03	echinoderm microtubule associated protein like 1 [Source:MGI Symbol;Acc:MGI:1915769]
She	1.11	6.9E-04	src homology 2 domain-containing transforming protein E [Source:MGI Symbol;Acc:MGI:1099462]
Disp2	1.11	1.8E-02	dispatched homolog 2 (Drosophila) [Source:MGI Symbol;Acc:MGI:2388733]
Sox17	1.11	1.4E-02	SRY (sex determining region Y)-box 17 [Source:MGI Symbol;Acc:MGI:107543]
Podn	1.11	4.2E-06	podocan [Source:MGI Symbol;Acc:MGI:2674939]
Dock10	1.11	1.5E-07	dedicator of cytokinesis 10 [Source:MGI Symbol;Acc:MGI:2146320]
Stard9	1.11	2.4E-03	START domain containing 9 [Source:MGI Symbol;Acc:MGI:3045258]
Zfp521	1.11	8.4E-03	zinc finger protein 521 [Source:MGI Symbol;Acc:MGI:95459]
Grb14	1.10	2.8E-02	growth factor receptor bound protein 14 [Source:MGI Symbol;Acc:MGI:1355324]
Dchs1	1.10	2.8E-03	dachsous 1 (Drosophila) [Source:MGI Symbol;Acc:MGI:2685011]
Plekhh2	1.10	9.0E-04	pleckstrin homology domain containing, family H (with MyTH4 domain) member 2 [Source:MGI Symbol;Acc:MGI:2146813]
Rab11fip5	1.10	4.0E-04	RAB11 family interacting protein 5 (class I) [Source:MGI Symbol;Acc:MGI:1098586]
Slc25a23	1.10	2.5E-04	solute carrier family 25 (mitochondrial carrier; phosphate carrier), member 23 [Source:MGI Symbol;Acc:MGI:1914222]
Tyrobp	1.10	1.5E-07	TYRO protein tyrosine kinase binding protein [Source:MGI Symbol;Acc:MGI:1277211]
Unc93b1	1.10	5.2E-09	unc-93 homolog B1 (C. elegans) [Source:MGI Symbol;Acc:MGI:1859307]
Arhgap30	1.10	2.3E-08	Rho GTPase activating protein 30 [Source:MGI Symbol;Acc:MGI:2684948]
Plxna4	1.10	2.3E-02	plexin A4 [Source:MGI Symbol;Acc:MGI:2179061]
Galnt18	1.10	1.6E-02	UDP-N-acetyl-alpha-D-galactosamine:polypeptide N-acetylgalactosaminyltransferase 18 [Source:MGI Symbol;Acc:MGI:2446239]
Slc7a14	1.10	4.2E-02	solute carrier family 7 (cationic amino acid transporter, y+ system), member 14 [Source:MGI Symbol;Acc:MGI:3040688]
Lrrc32	1.10	1.9E-04	leucine rich repeat containing 32 [Source:MGI Symbol;Acc:MGI:93882]
Nptxr	1.10	1.6E-02	neuronal pentraxin receptor [Source:MGI Symbol;Acc:MGI:1920590]
Fxyd6	1.10	4.2E-03	FXYD domain-containing ion transport regulator 6 [Source:MGI Symbol;Acc:MGI:1890226]
Sycn	1.10	3.5E-02	syncollin [Source:MGI Symbol;Acc:MGI:1915666]
Fam212b	1.10	1.2E-02	family with sequence similarity 212, member B [Source:MGI Symbol;Acc:MGI:1923497]
Rtn2	1.10	7.5E-03	reticulon 2 (Z-band associated protein) [Source:MGI Symbol;Acc:MGI:107612]
Me3	1.09	1.5E-02	malic enzyme 3, NADP(+)-dependent, mitochondrial [Source:MGI Symbol;Acc:MGI:1916679]
Cux2	1.09	3.4E-02	cut-like homeobox 2 [Source:MGI Symbol;Acc:MGI:107321]
Fkbp9	1.09	1.1E-04	FK506 binding protein 9 [Source:MGI Symbol;Acc:MGI:1350921]
Chrm2	1.09	4.8E-02	cholinergic receptor, muscarinic 2, cardiac [Source:MGI Symbol;Acc:MGI:88397]
Mdk	1.09	2.1E-02	midkine [Source:MGI Symbol;Acc:MGI:96949]
Fer	1.09	7.2E-06	fer (fms/fps related) protein kinase [Source:MGI Symbol;Acc:MGI:105917]
Ldb2	1.09	1.3E-02	LIM domain binding 2 [Source:MGI Symbol;Acc:MGI:894670]
Apoe	1.09	1.1E-03	apolipoprotein E [Source:MGI Symbol;Acc:MGI:88057]
Dpt	1.09	7.6E-05	dermatopontin [Source:MGI Symbol;Acc:MGI:1928392]
Tmem179	1.09	4.6E-02	transmembrane protein 179 [Source:MGI Symbol;Acc:MGI:2144891]
Gmpr	1.09	1.1E-02	guanosine monophosphate reductase [Source:MGI Symbol;Acc:MGI:1913605]
Nxpe5	1.09	1.9E-02	neurexophilin and PC-esterase domain family, member 5 [Source:MGI Symbol;Acc:MGI:3584036]
Adcy7	1.09	1.2E-04	adenylate cyclase 7 [Source:MGI Symbol;Acc:MGI:102891]
Ppfia4	1.09	4.7E-03	protein tyrosine phosphatase, receptor type, f polypeptide (PTPRF), interacting protein (liprin), alpha 4 [Source:MGI Symbol;Acc:MGI:1915757]
Maob	1.09	1.1E-05	monoamine oxidase B [Source:MGI Symbol;Acc:MGI:96916]
Cpxm1	1.09	4.2E-02	carboxypeptidase X 1 (M14 family) [Source:MGI Symbol;Acc:MGI:1934569]
Cp	1.09	2.3E-05	ceruloplasmin [Source:MGI Symbol;Acc:MGI:88476]
Dlg4	1.08	2.9E-03	discs, large homolog 4 (Drosophila) [Source:MGI Symbol;Acc:MGI:1277959]
Nnat	1.08	1.3E-02	neuronatin [Source:MGI Symbol;Acc:MGI:104716]
Adam8	1.08	3.1E-04	a disintegrin and metallopeptidase domain 8 [Source:MGI Symbol;Acc:MGI:107825]
Gsn	1.08	9.3E-06	gelsolin [Source:MGI Symbol;Acc:MGI:95851]
Col4a5	1.08	1.0E-03	collagen, type IV, alpha 5 [Source:MGI Symbol;Acc:MGI:88456]
Vill	1.08	1.8E-02	villin-like [Source:MGI Symbol;Acc:MGI:1201781]

Armcx1	1.08	7.5E-03	armadillo repeat containing, X-linked 1 [Source:MGI Symbol;Acc:MGI:1925498]
Plekho2	1.08	1.5E-05	pleckstrin homology domain containing, family O member 2 [Source:MGI Symbol;Acc:MGI:2143132]
Tmem117	1.08	1.0E-02	transmembrane protein 117 [Source:MGI Symbol;Acc:MGI:2444580]
Sspn	1.08	6.2E-03	sarcospan [Source:MGI Symbol;Acc:MGI:1353511]
Col28a1	1.08	4.0E-02	collagen, type XXVIII, alpha 1 [Source:MGI Symbol;Acc:MGI:2685312]
Limd2	1.08	4.6E-10	LIM domain containing 2 [Source:MGI Symbol;Acc:MGI:1915053]
Gli2	1.08	1.5E-02	GLI-Kruppel family member GLI2 [Source:MGI Symbol;Acc:MGI:95728]
Obsl1	1.08	8.2E-03	obscurin-like 1 [Source:MGI Symbol;Acc:MGI:2138628]
Csmd1	1.08	4.6E-02	CUB and Sushi multiple domains 1 [Source:MGI Symbol;Acc:MGI:2137383]
Card11	1.08	7.0E-06	caspase recruitment domain family, member 11 [Source:MGI Symbol;Acc:MGI:1916978]
Colec12	1.08	9.4E-03	collectin sub-family member 12 [Source:MGI Symbol;Acc:MGI:2152907]
Vegfc	1.07	1.2E-02	vascular endothelial growth factor C [Source:MGI Symbol;Acc:MGI:109124]
Fmnl3	1.07	2.9E-03	formin-like 3 [Source:MGI Symbol;Acc:MGI:109569]
Fam168a	1.07	2.7E-06	family with sequence similarity 168, member A [Source:MGI Symbol;Acc:MGI:2442372]
Sema6c	1.07	1.7E-02	sema domain, transmembrane domain (TM), and cytoplasmic domain, (semaphorin) 6C [Source:MGI Symbol;Acc:MGI:1338032]
Reck	1.07	1.8E-02	reversion-inducing-cysteine-rich protein with kazal motifs [Source:MGI Symbol;Acc:MGI:1855698]
Bmx	1.07	3.4E-02	BMX non-receptor tyrosine kinase [Source:MGI Symbol;Acc:MGI:1101778]
Foxred2	1.07	7.3E-03	FAD-dependent oxidoreductase domain containing 2 [Source:MGI Symbol;Acc:MGI:106315]
Klhl23	1.07	6.0E-03	kelch-like 23 [Source:MGI Symbol;Acc:MGI:2683536]
Tenc1	1.07	9.0E-06	tensin like C1 domain-containing phosphatase [Source:MGI Symbol;Acc:MGI:2387586]
Ppp1r18	1.07	1.5E-07	protein phosphatase 1, regulatory subunit 18 [Source:MGI Symbol;Acc:MGI:1923698]
Slfn3	1.07	3.6E-02	schlafen 3 [Source:MGI Symbol;Acc:MGI:1329005]
Cacna1d	1.07	1.2E-02	calcium channel, voltage-dependent, L type, alpha 1D subunit [Source:MGI Symbol;Acc:MGI:88293]
Adam22	1.07	5.1E-04	a disintegrin and metallopeptidase domain 22 [Source:MGI Symbol;Acc:MGI:1340046]
Sgce	1.06	1.7E-03	sarcoglycan, epsilon [Source:MGI Symbol;Acc:MGI:1329042]
Lox	1.06	7.0E-03	lysyl oxidase [Source:MGI Symbol;Acc:MGI:96817]
Hoxb2	1.06	4.3E-02	homeobox B2 [Source:MGI Symbol;Acc:MGI:96183]
Gm15922	1.06	4.4E-02	predicted gene 15922 [Source:MGI Symbol;Acc:MGI:3802148]
Igfbp3	1.06	7.0E-06	insulin-like growth factor binding protein 3 [Source:MGI Symbol;Acc:MGI:96438]
C1qa	1.06	1.3E-07	complement component 1, q subcomponent, alpha polypeptide [Source:MGI Symbol;Acc:MGI:88223]
Hmgcs2	1.06	2.2E-02	3-hydroxy-3-methylglutaryl-Coenzyme A synthase 2 [Source:MGI Symbol;Acc:MGI:101939]
Bbs1	1.06	5.2E-03	Bardet-Biedl syndrome 1 (human) [Source:MGI Symbol;Acc:MGI:1277215]
Rassf10	1.06	1.9E-02	Ras association (RalGDS/AF-6) domain family (N-terminal) member 10 [Source:MGI Symbol;Acc:MGI:1925998]
Thbs3	1.06	3.7E-03	thrombospondin 3 [Source:MGI Symbol;Acc:MGI:98739]
Dtna	1.06	3.0E-02	dystrobrevin alpha [Source:MGI Symbol;Acc:MGI:106039]
Syp	1.06	1.7E-02	synaptophysin [Source:MGI Symbol;Acc:MGI:98467]
Fn1	1.06	1.4E-02	fibronectin 1 [Source:MGI Symbol;Acc:MGI:95566]
Apba1	1.06	4.1E-03	amyloid beta (A4) precursor protein binding, family A, member 1 [Source:MGI Symbol;Acc:MGI:1860297]
Fgf1	1.06	1.4E-03	fibroblast growth factor 1 [Source:MGI Symbol;Acc:MGI:95515]
Ccdc109b	1.06	8.9E-03	coiled-coil domain containing 109B [Source:MGI Symbol;Acc:MGI:1914065]
BC068157	1.06	4.7E-02	cDNA sequence BC068157 [Source:MGI Symbol;Acc:MGI:3605626]
Slc4a3	1.06	2.4E-02	solute carrier family 4 (anion exchanger), member 3 [Source:MGI Symbol;Acc:MGI:109350]
Il33	1.05	3.8E-02	interleukin 33 [Source:MGI Symbol;Acc:MGI:1924375]
Etv1	1.05	1.5E-02	ets variant 1 [Source:MGI Symbol;Acc:MGI:99254]
Zfp651	1.05	4.6E-03	zinc finger protein 651 [Source:MGI Symbol;Acc:MGI:2670992]
Rcsd1	1.05	7.9E-05	RCSD domain containing 1 [Source:MGI Symbol;Acc:MGI:2676394]
Car2	1.05	6.9E-04	carbonic anhydrase 2 [Source:MGI Symbol;Acc:MGI:88269]
Dzip1	1.05	2.2E-02	DAZ interacting protein 1 [Source:MGI Symbol;Acc:MGI:1914311]
Cacna1b	1.05	3.7E-02	calcium channel, voltage-dependent, N type, alpha 1B subunit [Source:MGI Symbol;Acc:MGI:88296]
9430020K01Rik	1.05	1.5E-03	RIKEN cDNA 9430020K01 gene [Source:MGI Symbol;Acc:MGI:2685174]

Ppp1r12b	1.05	1.7E-02	protein phosphatase 1, regulatory (inhibitor) subunit 12B [Source:MGI Symbol;Acc:MGI:1916417]
Rspo3	1.05	3.9E-02	R-spondin 3 homolog (Xenopus laevis) [Source:MGI Symbol;Acc:MGI:1920030]
Reep1	1.05	3.4E-02	receptor accessory protein 1 [Source:MGI Symbol;Acc:MGI:1098827]
Cntln	1.05	2.1E-03	centlein, centrosomal protein [Source:MGI Symbol;Acc:MGI:2443104]
Morc4	1.05	2.2E-03	microrchidia 4 [Source:MGI Symbol;Acc:MGI:1922996]
Trip6	1.05	4.7E-03	thyroid hormone receptor interactor 6 [Source:MGI Symbol;Acc:MGI:1343458]
Apbb1	1.05	9.8E-03	amyloid beta (A4) precursor protein-binding, family B, member 1 [Source:MGI Symbol;Acc:MGI:107765]
Kif26a	1.05	5.4E-03	kinesin family member 26A [Source:MGI Symbol;Acc:MGI:2447072]
Ssu2	1.05	2.9E-02	ssu-2 homolog (C. elegans) [Source:MGI Symbol;Acc:MGI:2443733]
Phtf2	1.05	1.9E-04	putative homeodomain transcription factor 2 [Source:MGI Symbol;Acc:MGI:1916020]
Mapk8ip1	1.05	1.0E-02	mitogen-activated protein kinase 8 interacting protein 1 [Source:MGI Symbol;Acc:MGI:1309464]
Hhipl1	1.04	2.5E-02	hedgehog interacting protein-like 1 [Source:MGI Symbol;Acc:MGI:1919265]
Rem1	1.04	6.0E-03	rad and gem related GTP binding protein 1 [Source:MGI Symbol;Acc:MGI:1097696]
Tubb6	1.04	7.5E-03	tubulin, beta 6 class V [Source:MGI Symbol;Acc:MGI:1915201]
AI854703	1.04	2.0E-02	expressed sequence AI854703 [Source:MGI Symbol;Acc:MGI:2141510]
Upk1b	1.04	9.2E-03	uroplakin 1B [Source:MGI Symbol;Acc:MGI:98912]
Zfp658	1.04	5.3E-03	zinc finger protein 658 [Source:MGI Symbol;Acc:MGI:2652821]
Itgb2	1.04	1.2E-05	integrin beta 2 [Source:MGI Symbol;Acc:MGI:96611]
Islr	1.04	5.9E-03	immunoglobulin superfamily containing leucine-rich repeat [Source:MGI Symbol;Acc:MGI:1349645]
Nrp2	1.04	4.3E-05	neuropilin 2 [Source:MGI Symbol;Acc:MGI:1100492]
Atl1	1.04	4.6E-02	atlastin GTPase 1 [Source:MGI Symbol;Acc:MGI:1921241]
Mapk12	1.04	1.2E-02	mitogen-activated protein kinase 12 [Source:MGI Symbol;Acc:MGI:1353438]
Myom1	1.04	4.7E-03	myomesin 1 [Source:MGI Symbol;Acc:MGI:1341430]
Cpeb1	1.04	1.9E-02	cytoplasmic polyadenylation element binding protein 1 [Source:MGI Symbol;Acc:MGI:108442]
Rassf2	1.04	3.0E-04	Ras association (RalGDS/AF-6) domain family member 2 [Source:MGI Symbol;Acc:MGI:2442060]
Layn	1.04	3.8E-02	layilin [Source:MGI Symbol;Acc:MGI:2685357]
Sirpa	1.04	5.2E-07	signal-regulatory protein alpha [Source:MGI Symbol;Acc:MGI:108563]
Gm26572	1.04	2.2E-02	predicted gene, 26572 [Source:MGI Symbol;Acc:MGI:5477066]
Tmem45a	1.04	3.0E-02	transmembrane protein 45a [Source:MGI Symbol;Acc:MGI:1913122]
Tagap1	1.04	1.7E-03	T cell activation GTPase activating protein 1 [Source:MGI Symbol;Acc:MGI:1919786]
Pde8a	1.04	5.7E-04	phosphodiesterase 8A [Source:MGI Symbol;Acc:MGI:1277116]
Cd55	1.04	1.9E-04	CD55 antigen [Source:MGI Symbol;Acc:MGI:104850]
Vstm4	1.04	8.6E-03	V-set and transmembrane domain containing 4 [Source:MGI Symbol;Acc:MGI:2444633]
Hspb8	1.04	2.1E-02	heat shock protein 8 [Source:MGI Symbol;Acc:MGI:2135756]
Zfp467	1.04	3.3E-04	zinc finger protein 467 [Source:MGI Symbol;Acc:MGI:1916160]
Dzank1	1.04	3.1E-02	double zinc ribbon and ankyrin repeat domains 1 [Source:MGI Symbol;Acc:MGI:2139080]
Peli2	1.03	9.2E-04	pellino 2 [Source:MGI Symbol;Acc:MGI:1891445]
Thsd1	1.03	2.2E-02	thrombospondin, type I, domain 1 [Source:MGI Symbol;Acc:MGI:1929096]
Prickle2	1.03	6.8E-03	prickle homolog 2 (Drosophila) [Source:MGI Symbol;Acc:MGI:1925144]
Sesn1	1.03	1.6E-06	sestrin 1 [Source:MGI Symbol;Acc:MGI:2155278]
Tgfb1i1	1.03	9.4E-04	transforming growth factor beta 1 induced transcript 1 [Source:MGI Symbol;Acc:MGI:102784]
Hoxd10	1.03	6.8E-03	homeobox D10 [Source:MGI Symbol;Acc:MGI:96202]
Pygo1	1.03	3.3E-02	pygopus 1 [Source:MGI Symbol;Acc:MGI:1919385]
Syt15	1.03	1.1E-02	synaptotagmin XV [Source:MGI Symbol;Acc:MGI:2442166]
Gpr137b	1.03	3.2E-04	G protein-coupled receptor 137B [Source:MGI Symbol;Acc:MGI:1891463]
Wwox	1.03	5.7E-04	WW domain-containing oxidoreductase [Source:MGI Symbol;Acc:MGI:1931237]
Fcrl1	1.03	1.9E-02	Fc receptor-like 1 [Source:MGI Symbol;Acc:MGI:2442862]
Evc	1.03	1.1E-02	Ellis van Creveld gene syndrome [Source:MGI Symbol;Acc:MGI:1890596]
Itga9	1.03	1.2E-03	integrin alpha 9 [Source:MGI Symbol;Acc:MGI:104756]
Ptrf	1.03	6.9E-05	polymerase I and transcript release factor [Source:MGI Symbol;Acc:MGI:1277968]
Lgmn	1.03	4.4E-06	legumain [Source:MGI Symbol;Acc:MGI:1330838]
Aff3	1.03	1.6E-02	AF4/FMR2 family, member 3 [Source:MGI Symbol;Acc:MGI:106927]
Wdr35	1.03	9.2E-03	WD repeat domain 35 [Source:MGI Symbol;Acc:MGI:1921932]
Nacad	1.03	4.3E-02	NAC alpha domain containing [Source:MGI Symbol;Acc:MGI:3603030]

Cep112	1.03	2.4E-02	centrosomal protein 112 [Source:MGI Symbol;Acc:MGI:1923673]
Rgs5	1.03	1.0E-02	regulator of G-protein signaling 5 [Source:MGI Symbol;Acc:MGI:1098434]
Fbn1	1.03	1.3E-03	fibrillin 1 [Source:MGI Symbol;Acc:MGI:95489]
Calcb	1.02	3.4E-02	calcitonin-related polypeptide, beta [Source:MGI Symbol;Acc:MGI:2151254]
Nrn1	1.02	3.8E-02	neuritin 1 [Source:MGI Symbol;Acc:MGI:1915654]
Hfe	1.02	6.7E-04	hemochromatosis [Source:MGI Symbol;Acc:MGI:109191]
Hoxa11	1.02	1.9E-02	homeobox A11 [Source:MGI Symbol;Acc:MGI:96172]
Igsf6	1.02	7.1E-03	immunoglobulin superfamily, member 6 [Source:MGI Symbol;Acc:MGI:1891393]
Wasf1	1.02	3.2E-02	WAS protein family, member 1 [Source:MGI Symbol;Acc:MGI:1890563]
#N/A	1.02	4.9E-03	#N/A
Trpm5	1.02	3.9E-02	transient receptor potential cation channel, subfamily M, member 5 [Source:MGI Symbol;Acc:MGI:1861718]
Myh10	1.02	4.6E-03	myosin, heavy polypeptide 10, non-muscle [Source:MGI Symbol;Acc:MGI:1930780]
Cx3cr1	1.02	1.6E-03	chemokine (C-X3-C motif) receptor 1 [Source:MGI Symbol;Acc:MGI:1333815]
Zc3h12b	1.02	1.6E-02	zinc finger CCCH-type containing 12B [Source:MGI Symbol;Acc:MGI:2442133]
Prox1	1.02	3.7E-03	prospero homeobox 1 [Source:MGI Symbol;Acc:MGI:97772]
Jdp2	1.02	4.0E-03	Jun dimerization protein 2 [Source:MGI Symbol;Acc:MGI:1932093]
Mmp17	1.02	2.8E-02	matrix metallopeptidase 17 [Source:MGI Symbol;Acc:MGI:1346076]
Igf2	1.02	3.8E-04	insulin-like growth factor 2 [Source:MGI Symbol;Acc:MGI:96434]
Zfp423	1.02	1.4E-02	zinc finger protein 423 [Source:MGI Symbol;Acc:MGI:1891217]
Loxl1	1.02	3.3E-02	lysyl oxidase-like 1 [Source:MGI Symbol;Acc:MGI:106096]
Sybu	1.02	4.3E-02	syntabulin (syntaxin-interacting) [Source:MGI Symbol;Acc:MGI:2442392]
Rragb	1.02	4.2E-02	Ras-related GTP binding B [Source:MGI Symbol;Acc:MGI:3038613]
Gli3	1.02	2.0E-02	GLI-Kruppel family member GLI3 [Source:MGI Symbol;Acc:MGI:95729]
Lrrn2	1.01	3.7E-02	leucine rich repeat protein 2, neuronal [Source:MGI Symbol;Acc:MGI:106037]
Il10ra	1.01	2.3E-05	interleukin 10 receptor, alpha [Source:MGI Symbol;Acc:MGI:96538]
Amt	1.01	5.3E-03	aminomethyltransferase [Source:MGI Symbol;Acc:MGI:3646700]
St3gal1	1.01	2.5E-06	ST3 beta-galactoside alpha-2,3-sialyltransferase 1 [Source:MGI Symbol;Acc:MGI:98304]
Kcnk3	1.01	2.8E-02	potassium channel, subfamily K, member 3 [Source:MGI Symbol;Acc:MGI:1100509]
Cxxc5	1.01	8.9E-03	CXXC finger 5 [Source:MGI Symbol;Acc:MGI:1914643]
Fbln7	1.01	1.2E-02	fibulin 7 [Source:MGI Symbol;Acc:MGI:1917620]
Gm26724	1.01	4.0E-02	predicted gene, 26724 [Source:MGI Symbol;Acc:MGI:5477218]
Ltbp3	1.01	2.2E-04	latent transforming growth factor beta binding protein 3 [Source:MGI Symbol;Acc:MGI:1101355]
Tagln	1.01	4.1E-02	transgelin [Source:MGI Symbol;Acc:MGI:106012]
Gucy1b3	1.01	1.0E-02	guanylate cyclase 1, soluble, beta 3 [Source:MGI Symbol;Acc:MGI:1860604]
Ckb	1.01	6.1E-03	creatine kinase, brain [Source:MGI Symbol;Acc:MGI:88407]
Gpr27	1.01	4.8E-02	G protein-coupled receptor 27 [Source:MGI Symbol;Acc:MGI:1202299]
Ppic	1.01	3.5E-03	peptidylprolyl isomerase C [Source:MGI Symbol;Acc:MGI:97751]
Hspa1a	1.01	4.6E-03	heat shock protein 1A [Source:MGI Symbol;Acc:MGI:96244]
Arrb1	1.01	8.6E-06	arrestin, beta 1 [Source:MGI Symbol;Acc:MGI:99473]
Prkar1b	1.01	3.1E-02	protein kinase, cAMP dependent regulatory, type I beta [Source:MGI Symbol;Acc:MGI:97759]
Crispld2	1.01	4.3E-03	cysteine-rich secretory protein LCCL domain containing 2 [Source:MGI Symbol;Acc:MGI:1926142]
Adamts12	1.01	4.0E-02	a disintegrin-like and metallopeptidase (reprolysin type) with thrombospondin type 1 motif, 12 [Source:MGI Symbol;Acc:MGI:2146046]
Slc9a7	1.00	4.9E-02	solute carrier family 9 (sodium/hydrogen exchanger), member 7 [Source:MGI Symbol;Acc:MGI:2444530]
Ano2	1.00	1.4E-02	anoctamin 2 [Source:MGI Symbol;Acc:MGI:2387214]
Arap1	1.00	2.2E-09	ArfGAP with RhoGAP domain, ankyrin repeat and PH domain 1 [Source:MGI Symbol;Acc:MGI:1916960]
Rnf144a	1.00	1.1E-02	ring finger protein 144A [Source:MGI Symbol;Acc:MGI:1344401]
Spaca6	1.00	1.2E-02	sperm acrosome associated 6 [Source:MGI Symbol;Acc:MGI:1922452]
Atp1b2	1.00	1.6E-02	ATPase, Na+/K+ transporting, beta 2 polypeptide [Source:MGI Symbol;Acc:MGI:88109]
Sncaip	1.00	2.0E-02	synuclein, alpha interacting protein (synphilin) [Source:MGI Symbol;Acc:MGI:1915097]
Rbms3	1.00	1.5E-03	RNA binding motif, single stranded interacting protein [Source:MGI Symbol;Acc:MGI:2444477]
Atf3	1.00	4.1E-03	activating transcription factor 3 [Source:MGI Symbol;Acc:MGI:109384]
Fam171b	1.00	1.6E-02	family with sequence similarity 171, member B [Source:MGI Symbol;Acc:MGI:2444579]

Gm13375	1.00	2.0E-02	predicted gene 13375 [Source:MGI Symbol;Acc:MGI:3649913]
Timp3	1.00	2.6E-02	tissue inhibitor of metalloproteinase 3 [Source:MGI Symbol;Acc:MGI:98754]
Fbxl22	1.00	3.8E-02	F-box and leucine-rich repeat protein 22 [Source:MGI Symbol;Acc:MGI:1921415]
Wwc2	1.00	1.4E-04	WW, C2 and coiled-coil domain containing 2 [Source:MGI Symbol;Acc:MGI:1261872]
Ntrk2	1.00	4.8E-02	neurotrophic tyrosine kinase, receptor, type 2 [Source:MGI Symbol;Acc:MGI:97384]
Dmxl2	1.00	5.4E-03	Dmx-like 2 [Source:MGI Symbol;Acc:MGI:2444630]
Cav1	1.00	1.8E-04	caveolin 1, caveolae protein [Source:MGI Symbol;Acc:MGI:102709]
Abi3bp	1.00	2.9E-03	ABI gene family, member 3 (NESH) binding protein [Source:MGI Symbol;Acc:MGI:2444583]
Inpp5d	1.00	6.2E-07	inositol polyphosphate-5-phosphatase D [Source:MGI Symbol;Acc:MGI:107357]
Fzd2	1.00	1.9E-02	frizzled homolog 2 (Drosophila) [Source:MGI Symbol;Acc:MGI:1888513]
Arsj	1.00	1.4E-02	arylsulfatase J [Source:MGI Symbol;Acc:MGI:2443513]
Porcn	1.00	2.0E-02	porcupine homolog (Drosophila) [Source:MGI Symbol;Acc:MGI:1890212]
Npr2	0.99	2.0E-03	natriuretic peptide receptor 2 [Source:MGI Symbol;Acc:MGI:97372]
Sspo	0.99	2.2E-02	SCO-spondin [Source:MGI Symbol;Acc:MGI:2674311]
Pirb	0.99	1.0E-02	paired Ig-like receptor B [Source:MGI Symbol;Acc:MGI:894311]
Zbtb4	0.99	1.8E-06	zinc finger and BTB domain containing 4 [Source:MGI Symbol;Acc:MGI:1922830]
Adck3	0.99	7.6E-03	aarF domain containing kinase 3 [Source:MGI Symbol;Acc:MGI:1914676]
Gatm	0.99	1.8E-05	glycine amidinotransferase (L-arginine:glycine amidinotransferase) [Source:MGI Symbol;Acc:MGI:1914342]
Zc2hc1c	0.99	4.9E-02	zinc finger, C2HC-type containing 1C [Source:MGI Symbol;Acc:MGI:1919600]
Nfatc4	0.99	5.8E-03	nuclear factor of activated T cells, cytoplasmic, calcineurin dependent 4 [Source:MGI Symbol;Acc:MGI:1920431]
Zfp281	0.99	9.5E-06	zinc finger protein 281 [Source:MGI Symbol;Acc:MGI:3029290]
Fgd1	0.99	2.9E-02	FYVE, RhoGEF and PH domain containing 1 [Source:MGI Symbol;Acc:MGI:104566]
Zc4h2	0.99	1.8E-02	zinc finger, C4H2 domain containing [Source:MGI Symbol;Acc:MGI:2679294]
Matn2	0.99	2.3E-04	matrilin 2 [Source:MGI Symbol;Acc:MGI:109613]
Wwtr1	0.99	7.0E-03	WW domain containing transcription regulator 1 [Source:MGI Symbol;Acc:MGI:1917649]
Gpr182	0.99	8.2E-03	G protein-coupled receptor 182 [Source:MGI Symbol;Acc:MGI:109545]
Olfml3	0.99	2.4E-02	olfactomedin-like 3 [Source:MGI Symbol;Acc:MGI:1914877]
Fhad1	0.99	7.6E-03	forkhead-associated (FHA) phosphopeptide binding domain 1 [Source:MGI Symbol;Acc:MGI:1920323]
BC064078	0.99	8.0E-04	cDNA sequence BC064078 [Source:MGI Symbol;Acc:MGI:3040692]
2610203C20Rik	0.99	3.3E-02	RIKEN cDNA 2610203C20 gene [Source:MGI Symbol;Acc:MGI:1917705]
Rai2	0.99	4.7E-03	retinoic acid induced 2 [Source:MGI Symbol;Acc:MGI:1344378]
Spn	0.99	6.8E-04	sialophorin [Source:MGI Symbol;Acc:MGI:98384]
Fstl3	0.99	4.3E-02	follistatin-like 3 [Source:MGI Symbol;Acc:MGI:1890391]
Fhl1	0.99	5.5E-05	four and a half LIM domains 1 [Source:MGI Symbol;Acc:MGI:1298387]
Ubash3b	0.99	4.1E-04	ubiquitin associated and SH3 domain containing, B [Source:MGI Symbol;Acc:MGI:1920078]
Lbp	0.99	1.8E-02	lipopolysaccharide binding protein [Source:MGI Symbol;Acc:MGI:1098776]
Sv2b	0.99	4.6E-02	synaptic vesicle glycoprotein 2 b [Source:MGI Symbol;Acc:MGI:1927338]
Evl	0.99	2.5E-04	Ena-vasodilator stimulated phosphoprotein [Source:MGI Symbol;Acc:MGI:1194884]
Clec3b	0.98	1.7E-03	C-type lectin domain family 3, member b [Source:MGI Symbol;Acc:MGI:104540]
C1qtnf2	0.98	2.7E-02	C1q and tumor necrosis factor related protein 2 [Source:MGI Symbol;Acc:MGI:1916433]
Klhdc1	0.98	2.2E-02	kelch domain containing 1 [Source:MGI Symbol;Acc:MGI:2672853]
Gab3	0.98	2.1E-02	growth factor receptor bound protein 2-associated protein 3 [Source:MGI Symbol;Acc:MGI:2387324]
Armcx4	0.98	1.3E-02	armadillo repeat containing, X-linked 4 [Source:MGI Symbol;Acc:MGI:2147887]
Tcf21	0.98	5.9E-04	transcription factor 21 [Source:MGI Symbol;Acc:MGI:1202715]
Tppp3	0.98	1.4E-04	tubulin polymerization-promoting protein family member 3 [Source:MGI Symbol;Acc:MGI:1915221]
Kirrel	0.98	1.1E-02	kin of IRRE like (Drosophila) [Source:MGI Symbol;Acc:MGI:1891396]
Efhc1	0.98	4.3E-02	EF-hand domain (C-terminal) containing 1 [Source:MGI Symbol;Acc:MGI:1919127]
Rogdi	0.98	2.5E-05	rogdi homolog (Drosophila) [Source:MGI Symbol;Acc:MGI:1913299]
Tbc1d16	0.98	1.5E-03	TBC1 domain family, member 16 [Source:MGI Symbol;Acc:MGI:2652878]
N4bp2l1	0.98	7.0E-04	NEDD4 binding protein 2-like 1 [Source:MGI Symbol;Acc:MGI:2140872]
Pdxk	0.98	9.2E-05	pyridoxal (pyridoxine, vitamin B6) kinase [Source:MGI Symbol;Acc:MGI:1351869]
Fam189a1	0.98	4.6E-02	family with sequence similarity 189, member A1 [Source:MGI Symbol;Acc:MGI:1917888]

Dennd1a	0.98	7.2E-06	DENN/MADD domain containing 1A [Source:MGI Symbol;Acc:MGI:2442794]
Zfhx3	0.98	2.8E-05	zinc finger homeobox 3 [Source:MGI Symbol;Acc:MGI:99948]
Gata2	0.98	7.4E-03	GATA binding protein 2 [Source:MGI Symbol;Acc:MGI:95662]
Bcam	0.97	8.1E-04	basal cell adhesion molecule [Source:MGI Symbol;Acc:MGI:1929940]
Vim	0.97	5.1E-04	vimentin [Source:MGI Symbol;Acc:MGI:98932]
Tmem100	0.97	1.8E-02	transmembrane protein 100 [Source:MGI Symbol;Acc:MGI:1915138]
Gpsm3	0.97	5.2E-05	G-protein signalling modulator 3 (AGS3-like, C. elegans) [Source:MGI Symbol;Acc:MGI:2146785]
Gdnf	0.97	2.7E-02	glial cell line derived neurotrophic factor [Source:MGI Symbol;Acc:MGI:107430]
Csgalnact1	0.97	3.0E-02	chondroitin sulfate N-acetylgalactosaminyltransferase 1 [Source:MGI Symbol;Acc:MGI:2442354]
Bambi	0.97	1.9E-02	BMP and activin membrane-bound inhibitor [Source:MGI Symbol;Acc:MGI:1915260]
Rslcan18	0.97	4.8E-02	regulator of sex-limitation candidate 18 [Source:MGI Symbol;Acc:MGI:5433745]
Pcsk6	0.97	1.4E-03	proprotein convertase subtilisin/kexin type 6 [Source:MGI Symbol;Acc:MGI:102897]
Srcin1	0.97	4.8E-02	SRC kinase signaling inhibitor 1 [Source:MGI Symbol;Acc:MGI:1933179]
Zcchc3	0.97	3.0E-02	zinc finger, CCHC domain containing 3 [Source:MGI Symbol;Acc:MGI:1915167]
Fxyd1	0.97	9.0E-03	FXYD domain-containing ion transport regulator 1 [Source:MGI Symbol;Acc:MGI:1889273]
Sdk1	0.97	3.7E-03	sidekick homolog 1 (chicken) [Source:MGI Symbol;Acc:MGI:2444413]
Tpm2	0.97	3.3E-02	tropomyosin 2, beta [Source:MGI Symbol;Acc:MGI:98810]
Cnrip1	0.97	2.3E-02	cannabinoid receptor interacting protein 1 [Source:MGI Symbol;Acc:MGI:1917505]
Rbpms2	0.97	3.2E-02	RNA binding protein with multiple splicing 2 [Source:MGI Symbol;Acc:MGI:1919223]
Fbln5	0.97	9.6E-04	fibulin 5 [Source:MGI Symbol;Acc:MGI:1346091]
Clip4	0.96	4.6E-02	CAP-GLY domain containing linker protein family, member 4 [Source:MGI Symbol;Acc:MGI:1919100]
Calcrl	0.96	3.8E-03	calcitonin receptor-like [Source:MGI Symbol;Acc:MGI:1926944]
Tle2	0.96	8.7E-03	transducin-like enhancer of split 2, homolog of Drosophila E(spl) [Source:MGI Symbol;Acc:MGI:104635]
Amotl1	0.96	6.4E-04	angiomotin-like 1 [Source:MGI Symbol;Acc:MGI:1922973]
Rapgef4	0.96	2.6E-02	Rap guanine nucleotide exchange factor (GEF) 4 [Source:MGI Symbol;Acc:MGI:1917723]
Psd	0.96	2.5E-02	pleckstrin and Sec7 domain containing [Source:MGI Symbol;Acc:MGI:1920978]
Mmrn2	0.96	2.9E-03	multimerin 2 [Source:MGI Symbol;Acc:MGI:2385618]
Tlr6	0.96	1.5E-02	toll-like receptor 6 [Source:MGI Symbol;Acc:MGI:1341296]
Adarb1	0.96	3.1E-02	adenosine deaminase, RNA-specific, B1 [Source:MGI Symbol;Acc:MGI:891999]
Igdcc4	0.96	4.6E-02	immunoglobulin superfamily, DCC subclass, member 4 [Source:MGI Symbol;Acc:MGI:1858497]
Col20a1	0.96	1.1E-02	collagen, type XX, alpha 1 [Source:MGI Symbol;Acc:MGI:1920618]
Rerg	0.96	2.1E-02	RAS-like, estrogen-regulated, growth-inhibitor [Source:MGI Symbol;Acc:MGI:2665139]
Trabd2b	0.96	4.6E-02	TraB domain containing 2B [Source:MGI Symbol;Acc:MGI:3650152]
Epdr1	0.96	1.4E-02	ependymin related protein 1 (zebrafish) [Source:MGI Symbol;Acc:MGI:2145369]
Tceal8	0.96	4.6E-03	transcription elongation factor A (SII)-like 8 [Source:MGI Symbol;Acc:MGI:1913934]
Hspa2	0.95	1.3E-02	heat shock protein 2 [Source:MGI Symbol;Acc:MGI:96243]
Rhobtb1	0.95	1.9E-03	Rho-related BTB domain containing 1 [Source:MGI Symbol;Acc:MGI:1916538]
Fbln1	0.95	1.3E-02	fibulin 1 [Source:MGI Symbol;Acc:MGI:95487]
Ltbp1	0.95	9.8E-05	latent transforming growth factor beta binding protein 1 [Source:MGI Symbol;Acc:MGI:109151]
Efs	0.95	2.3E-02	embryonal Fyn-associated substrate [Source:MGI Symbol;Acc:MGI:105311]
Otub2	0.95	2.3E-02	OTU domain, ubiquitin aldehyde binding 2 [Source:MGI Symbol;Acc:MGI:1915399]
Stard8	0.95	2.7E-03	START domain containing 8 [Source:MGI Symbol;Acc:MGI:2448556]
Prickle1	0.95	1.1E-03	prickle homolog 1 (Drosophila) [Source:MGI Symbol;Acc:MGI:1916034]
Ophn1	0.95	5.9E-04	oligophrenin 1 [Source:MGI Symbol;Acc:MGI:2151070]
Alox5	0.95	1.5E-02	arachidonate 5-lipoxygenase [Source:MGI Symbol;Acc:MGI:87999]
Cyp27a1	0.95	8.4E-03	cytochrome P450, family 27, subfamily a, polypeptide 1 [Source:MGI Symbol;Acc:MGI:88594]
Ehd1	0.95	9.9E-07	EH-domain containing 1 [Source:MGI Symbol;Acc:MGI:1341878]
Prex1	0.95	9.2E-06	phosphatidylinositol-3,4,5-trisphosphate-dependent Rac exchange factor 1 [Source:MGI Symbol;Acc:MGI:3040696]
Zfp503	0.95	4.5E-03	zinc finger protein 503 [Source:MGI Symbol;Acc:MGI:1353644]
Cmtm3	0.95	1.6E-03	CKLF-like MARVEL transmembrane domain containing 3 [Source:MGI Symbol;Acc:MGI:2447162]

Pck1	0.95	3.5E-02	phosphoenolpyruvate carboxykinase 1, cytosolic [Source:MGI Symbol;Acc:MGI:97501]
Mill2	0.95	1.8E-02	MHC I like leukocyte 2 [Source:MGI Symbol;Acc:MGI:2179989]
Kank2	0.95	1.2E-04	KN motif and ankyrin repeat domains 2 [Source:MGI Symbol;Acc:MGI:2384568]
Pfn2	0.94	3.2E-02	profilin 2 [Source:MGI Symbol;Acc:MGI:97550]
Tdrp	0.94	3.8E-02	testis development related protein [Source:MGI Symbol;Acc:MGI:1919398]
Cd22	0.94	2.9E-02	CD22 antigen [Source:MGI Symbol;Acc:MGI:88322]
Agt	0.94	1.8E-02	angiotensinogen (serpin peptidase inhibitor, clade A, member 8) [Source:MGI Symbol;Acc:MGI:87963]
Arhgef25	0.94	6.0E-03	Rho guanine nucleotide exchange factor (GEF) 25 [Source:MGI Symbol;Acc:MGI:1277173]
Snta1	0.94	6.9E-03	syntrophin, acidic 1 [Source:MGI Symbol;Acc:MGI:101772]
Tmem86a	0.94	1.2E-02	transmembrane protein 86A [Source:MGI Symbol;Acc:MGI:1915143]
Tanc2	0.94	3.3E-03	tetratricopeptide repeat, ankyrin repeat and coiled-coil containing 2 [Source:MGI Symbol;Acc:MGI:2444121]
Cytl1	0.94	3.2E-02	cytokine-like 1 [Source:MGI Symbol;Acc:MGI:2684993]
Sepp1	0.94	3.8E-02	selenoprotein P, plasma, 1 [Source:MGI Symbol;Acc:MGI:894288]
Ror2	0.94	1.6E-02	receptor tyrosine kinase-like orphan receptor 2 [Source:MGI Symbol;Acc:MGI:1347521]
Cryab	0.94	2.2E-02	crystallin, alpha B [Source:MGI Symbol;Acc:MGI:88516]
Renbp	0.94	4.1E-03	renin binding protein [Source:MGI Symbol;Acc:MGI:105940]
Marveld1	0.94	4.9E-03	MARVEL (membrane-associating) domain containing 1 [Source:MGI Symbol;Acc:MGI:2147570]
Il16	0.94	3.2E-03	interleukin 16 [Source:MGI Symbol;Acc:MGI:1270855]
Alox12	0.94	4.4E-02	arachidonate 12-lipoxygenase [Source:MGI Symbol;Acc:MGI:87998]
Isx	0.94	2.3E-02	intestine specific homeobox [Source:MGI Symbol;Acc:MGI:1918847]
Anxa6	0.93	1.9E-04	annexin A6 [Source:MGI Symbol;Acc:MGI:88255]
Ddr2	0.93	7.0E-03	discoidin domain receptor family, member 2 [Source:MGI Symbol;Acc:MGI:1345277]
Cdk19	0.93	9.8E-06	cyclin-dependent kinase 19 [Source:MGI Symbol;Acc:MGI:1925584]
Dapk1	0.93	8.0E-04	death associated protein kinase 1 [Source:MGI Symbol;Acc:MGI:1916885]
Rfx2	0.93	2.7E-02	regulatory factor X, 2 (influences HLA class II expression) [Source:MGI Symbol;Acc:MGI:106583]
Plekha4	0.93	4.6E-02	pleckstrin homology domain containing, family A (phosphoinositide binding specific) member 4 [Source:MGI Symbol;Acc:MGI:1916467]
Lat2	0.93	1.7E-03	linker for activation of T cells family, member 2 [Source:MGI Symbol;Acc:MGI:1926479]
Kcnma1	0.93	2.4E-02	potassium large conductance calcium-activated channel, subfamily M, alpha member 1 [Source:MGI Symbol;Acc:MGI:99923]
Ascl2	0.93	9.6E-03	achaete-scute complex homolog 2 (Drosophila) [Source:MGI Symbol;Acc:MGI:96920]
Lyl1	0.93	1.9E-02	lymphoblastomic leukemia 1 [Source:MGI Symbol;Acc:MGI:96891]
Itga8	0.93	4.3E-02	integrin alpha 8 [Source:MGI Symbol;Acc:MGI:109442]
Ssbp2	0.93	5.4E-04	single-stranded DNA binding protein 2 [Source:MGI Symbol;Acc:MGI:1914220]
Tmem47	0.93	2.0E-02	transmembrane protein 47 [Source:MGI Symbol;Acc:MGI:2177570]
Zscan2	0.93	3.7E-02	zinc finger and SCAN domain containing 2 [Source:MGI Symbol;Acc:MGI:99176]
Camk1	0.92	2.6E-04	calcium/calmodulin-dependent protein kinase I [Source:MGI Symbol;Acc:MGI:1098535]
Sorcs2	0.92	2.8E-02	sortilin-related VPS10 domain containing receptor 2 [Source:MGI Symbol;Acc:MGI:1932289]
D630003M21Rik	0.92	2.8E-02	RIKEN cDNA D630003M21 gene [Source:MGI Symbol;Acc:MGI:3606579]
Celf2	0.92	4.9E-07	CUGBP, Elav-like family member 2 [Source:MGI Symbol;Acc:MGI:1338822]
Zfp385a	0.92	7.7E-04	zinc finger protein 385A [Source:MGI Symbol;Acc:MGI:1352495]
Hspg2	0.92	5.1E-03	perlecan (heparan sulfate proteoglycan 2) [Source:MGI Symbol;Acc:MGI:96257]
Smtn	0.92	1.8E-02	smoothelin [Source:MGI Symbol;Acc:MGI:1354727]
Ikzf1	0.92	7.0E-05	IKAROS family zinc finger 1 [Source:MGI Symbol;Acc:MGI:1342540]
Tspan9	0.92	3.1E-03	tetraspanin 9 [Source:MGI Symbol;Acc:MGI:1924558]
Ephx1	0.92	1.7E-02	epoxide hydrolase 1, microsomal [Source:MGI Symbol;Acc:MGI:95405]
Mras	0.92	4.3E-03	muscle and microspikes RAS [Source:MGI Symbol;Acc:MGI:1100856]
Tgfb1	0.92	1.1E-06	transforming growth factor, beta 1 [Source:MGI Symbol;Acc:MGI:98725]
Slc43a3	0.91	5.5E-04	solute carrier family 43, member 3 [Source:MGI Symbol;Acc:MGI:1931054]
Hivep3	0.91	5.4E-05	human immunodeficiency virus type I enhancer binding protein 3 [Source:MGI Symbol;Acc:MGI:106589]
Maged2	0.91	8.7E-03	melanoma antigen, family D, 2 [Source:MGI Symbol;Acc:MGI:1933391]
Afap1l1	0.91	3.2E-02	actin filament associated protein 1-like 1 [Source:MGI Symbol;Acc:MGI:2147199]

Cped1	0.91	6.3E-04	cadherin-like and PC-esterase domain containing 1 [Source:MGI Symbol;Acc:MGI:2444814]
Ephx2	0.91	3.8E-03	epoxide hydrolase 2, cytoplasmic [Source:MGI Symbol;Acc:MGI:99500]
Jam3	0.91	6.9E-03	junction adhesion molecule 3 [Source:MGI Symbol;Acc:MGI:1933825]
Col16a1	0.91	1.1E-03	collagen, type XVI, alpha 1 [Source:MGI Symbol;Acc:MGI:1095396]
Arid3b	0.91	1.7E-04	AT rich interactive domain 3B (BRIGHT-like) [Source:MGI Symbol;Acc:MGI:1930768]
Rgs19	0.91	3.8E-03	regulator of G-protein signaling 19 [Source:MGI Symbol;Acc:MGI:1915153]
Cxcl12	0.91	3.7E-03	chemokine (C-X-C motif) ligand 12 [Source:MGI Symbol;Acc:MGI:103556]
Ncf4	0.91	2.0E-05	neutrophil cytosolic factor 4 [Source:MGI Symbol;Acc:MGI:109186]
Mxra7	0.91	7.2E-03	matrix-remodelling associated 7 [Source:MGI Symbol;Acc:MGI:1914872]
Ppap2b	0.91	8.7E-05	phosphatidic acid phosphatase type 2B [Source:MGI Symbol;Acc:MGI:1915166]
Bicc1	0.91	3.4E-03	bicaudal C homolog 1 (Drosophila) [Source:MGI Symbol;Acc:MGI:1933388]
Scara3	0.91	2.2E-02	scavenger receptor class A, member 3 [Source:MGI Symbol;Acc:MGI:2444418]
Snn	0.91	2.0E-02	stannin [Source:MGI Symbol;Acc:MGI:1276549]
Art3	0.91	1.3E-02	ADP-ribosyltransferase 3 [Source:MGI Symbol;Acc:MGI:1202729]
Glul	0.91	5.2E-05	glutamate-ammonia ligase (glutamine synthetase) [Source:MGI Symbol;Acc:MGI:95739]
Tek	0.91	5.9E-03	endothelial-specific receptor tyrosine kinase [Source:MGI Symbol;Acc:MGI:98664]
Grb10	0.90	1.8E-02	growth factor receptor bound protein 10 [Source:MGI Symbol;Acc:MGI:103232]
Rnase4	0.90	4.8E-03	ribonuclease, RNase A family 4 [Source:MGI Symbol;Acc:MGI:1926217]
Rhoq	0.90	3.3E-05	ras homolog gene family, member Q [Source:MGI Symbol;Acc:MGI:1931553]
Gadd45b	0.90	9.9E-05	growth arrest and DNA-damage-inducible 45 beta [Source:MGI Symbol;Acc:MGI:107776]
Elmo1	0.90	9.7E-06	engulfment and cell motility 1 [Source:MGI Symbol;Acc:MGI:2153044]
Trps1	0.90	7.6E-03	trichorhinophalangeal syndrome I (human) [Source:MGI Symbol;Acc:MGI:1927616]
Rab31	0.90	1.0E-04	RAB31, member RAS oncogene family [Source:MGI Symbol;Acc:MGI:1914603]
Suco	0.90	2.8E-07	SUN domain containing ossification factor [Source:MGI Symbol;Acc:MGI:2138346]
Ctsh	0.90	3.9E-07	cathepsin H [Source:MGI Symbol;Acc:MGI:107285]
Cav2	0.90	4.5E-03	caveolin 2 [Source:MGI Symbol;Acc:MGI:107571]
Cacng7	0.90	3.1E-02	calcium channel, voltage-dependent, gamma subunit 7 [Source:MGI Symbol;Acc:MGI:1932374]
Ssc5d	0.90	7.4E-03	scavenger receptor cysteine rich family, 5 domains [Source:MGI Symbol;Acc:MGI:3606211]
Nfkbiz	0.90	1.4E-03	nuclear factor of kappa light polypeptide gene enhancer in B cells inhibitor, zeta [Source:MGI Symbol;Acc:MGI:1931595]
Wdfy2	0.89	3.6E-02	WD repeat and FYVE domain containing 2 [Source:MGI Symbol;Acc:MGI:2442811]
Ms4a6b	0.89	5.2E-03	membrane-spanning 4-domains, subfamily A, member 6B [Source:MGI Symbol;Acc:MGI:1917024]
Tln1	0.89	1.5E-05	talin 1 [Source:MGI Symbol;Acc:MGI:1099832]
Qk	0.89	1.7E-04	quaking [Source:MGI Symbol;Acc:MGI:97837]
Irf5	0.89	9.9E-05	interferon regulatory factor 5 [Source:MGI Symbol;Acc:MGI:1350924]
Fat4	0.89	7.5E-03	FAT tumor suppressor homolog 4 (Drosophila) [Source:MGI Symbol;Acc:MGI:3045256]
Mtr	0.89	3.6E-03	5-methyltetrahydrofolate-homocysteine methyltransferase [Source:MGI Symbol;Acc:MGI:894292]
Zscan26	0.89	4.1E-04	zinc finger and SCAN domain containing 26 [Source:MGI Symbol;Acc:MGI:3531417]
Gli1	0.89	1.2E-02	GLI-Kruppel family member GLI1 [Source:MGI Symbol;Acc:MGI:95727]
Klhdc8b	0.89	2.0E-02	kelch domain containing 8B [Source:MGI Symbol;Acc:MGI:1925517]
Tspan4	0.89	1.5E-02	tetraspanin 4 [Source:MGI Symbol;Acc:MGI:1928097]
Numbl	0.89	6.2E-03	numb-like [Source:MGI Symbol;Acc:MGI:894702]
Mtcl1	0.89	9.1E-03	microtubule crosslinking factor 1 [Source:MGI Symbol;Acc:MGI:1915867]
Asrgl1	0.89	1.4E-02	asparaginase like 1 [Source:MGI Symbol;Acc:MGI:1913764]
Lyn	0.89	1.2E-08	Yamaguchi sarcoma viral (v-yes-1) oncogene homolog [Source:MGI Symbol;Acc:MGI:96892]
Zcchc24	0.89	9.3E-04	zinc finger, CCHC domain containing 24 [Source:MGI Symbol;Acc:MGI:1919168]
Plekho1	0.89	4.4E-04	pleckstrin homology domain containing, family O member 1 [Source:MGI Symbol;Acc:MGI:1914470]
Man1c1	0.89	1.5E-03	mannosidase, alpha, class 1C, member 1 [Source:MGI Symbol;Acc:MGI:2446214]
Srgap2	0.89	6.6E-04	SLIT-ROBO Rho GTPase activating protein 2 [Source:MGI Symbol;Acc:MGI:109605]
Zeb1	0.89	3.9E-03	zinc finger E-box binding homeobox 1 [Source:MGI Symbol;Acc:MGI:1344313]
Hspb1	0.89	3.8E-02	heat shock protein 1 [Source:MGI Symbol;Acc:MGI:96240]
Ptpn13	0.89	1.3E-03	protein tyrosine phosphatase, non-receptor type 13 [Source:MGI Symbol;Acc:MGI:103293]

Aox1	0.89	2.4E-02	aldehyde oxidase 1 [Source:MGI Symbol;Acc:MGI:88035]
Smarcd3	0.89	4.3E-02	SWI/SNF related, matrix associated, actin dependent regulator of chromatin, subfamily d, member 3 [Source:MGI Symbol;Acc:MGI:1914243]
Alox5ap	0.88	1.3E-03	arachidonate 5-lipoxygenase activating protein [Source:MGI Symbol;Acc:MGI:107505]
Cdkn1c	0.88	1.7E-02	cyclin-dependent kinase inhibitor 1C (P57) [Source:MGI Symbol;Acc:MGI:104564]
Xpo4	0.88	4.7E-03	exportin 4 [Source:MGI Symbol;Acc:MGI:1888526]
Fam222a	0.88	4.1E-02	family with sequence similarity 222, member A [Source:MGI Symbol;Acc:MGI:3605543]
Tfpi	0.88	1.2E-02	tissue factor pathway inhibitor [Source:MGI Symbol;Acc:MGI:1095418]
Katnal1	0.88	1.1E-02	katanin p60 subunit A-like 1 [Source:MGI Symbol;Acc:MGI:2387638]
Lepr	0.88	1.5E-03	leptin receptor [Source:MGI Symbol;Acc:MGI:104993]
Armcx2	0.88	4.0E-02	armadillo repeat containing, X-linked 2 [Source:MGI Symbol;Acc:MGI:1914666]
Nbl1	0.88	2.4E-04	neuroblastoma, suppression of tumorigenicity 1 [Source:MGI Symbol;Acc:MGI:104591]
Crip2	0.88	1.9E-04	cysteine rich protein 2 [Source:MGI Symbol;Acc:MGI:1915587]
Arhgap23	0.88	1.2E-02	Rho GTPase activating protein 23 [Source:MGI Symbol;Acc:MGI:3697726]
Olfr78	0.88	4.5E-02	olfactory receptor 78 [Source:MGI Symbol;Acc:MGI:2157548]
Grin2d	0.88	1.4E-02	glutamate receptor, ionotropic, NMDA2D (epsilon 4) [Source:MGI Symbol;Acc:MGI:95823]
Eml6	0.88	2.6E-02	echinoderm microtubule associated protein like 6 [Source:MGI Symbol;Acc:MGI:2442895]
Msl3l2	0.88	3.2E-02	male-specific lethal 3-like 2 (Drosophila) [Source:MGI Symbol;Acc:MGI:1920640]
C4b	0.88	8.2E-03	complement component 4B (Chido blood group) [Source:MGI Symbol;Acc:MGI:88228]
Antxr1	0.88	7.4E-04	anthrax toxin receptor 1 [Source:MGI Symbol;Acc:MGI:1916788]
Rdx	0.88	2.7E-04	radixin [Source:MGI Symbol;Acc:MGI:97887]
Sh3bgrl3	0.88	1.2E-07	SH3 domain binding glutamic acid-rich protein-like 3 [Source:MGI Symbol;Acc:MGI:1920973]
Pam	0.88	1.1E-03	peptidylglycine alpha-amidating monooxygenase [Source:MGI Symbol;Acc:MGI:97475]
Tenm3	0.88	4.4E-02	teneurin transmembrane protein 3 [Source:MGI Symbol;Acc:MGI:1345183]
Srgap3	0.88	3.4E-03	SLIT-ROBO Rho GTPase activating protein 3 [Source:MGI Symbol;Acc:MGI:2152938]
Arhgap25	0.87	2.2E-03	Rho GTPase activating protein 25 [Source:MGI Symbol;Acc:MGI:2443687]
Tbc1d19	0.87	1.3E-02	TBC1 domain family, member 19 [Source:MGI Symbol;Acc:MGI:1914499]
Shank3	0.87	5.7E-03	SH3/ankyrin domain gene 3 [Source:MGI Symbol;Acc:MGI:1930016]
Gjc1	0.87	1.9E-02	gap junction protein, gamma 1 [Source:MGI Symbol;Acc:MGI:95718]
Mrc2	0.87	1.9E-02	mannose receptor, C type 2 [Source:MGI Symbol;Acc:MGI:107818]
Cfh	0.87	4.3E-02	complement component factor h [Source:MGI Symbol;Acc:MGI:88385]
Hspa12b	0.87	1.6E-02	heat shock protein 12B [Source:MGI Symbol;Acc:MGI:1919880]
Mapt	0.87	5.0E-02	microtubule-associated protein tau [Source:MGI Symbol;Acc:MGI:97180]
Chst14	0.87	8.9E-03	carbohydrate (N-acetylgalactosamine 4-0) sulfotransferase 14 [Source:MGI Symbol;Acc:MGI:1919386]
Ehd2	0.87	4.0E-03	EH-domain containing 2 [Source:MGI Symbol;Acc:MGI:2154274]
Slc9a3r2	0.87	1.2E-03	solute carrier family 9 (sodium/hydrogen exchanger), member 3 regulator 2 [Source:MGI Symbol;Acc:MGI:1890662]
Il18	0.87	1.4E-03	interleukin 18 [Source:MGI Symbol;Acc:MGI:107936]
Tmem204	0.87	9.4E-03	transmembrane protein 204 [Source:MGI Symbol;Acc:MGI:3039635]
Pla2r1	0.87	1.4E-02	phospholipase A2 receptor 1 [Source:MGI Symbol;Acc:MGI:102468]
Ahi1	0.87	4.0E-02	Abelson helper integration site 1 [Source:MGI Symbol;Acc:MGI:87971]
Filip1	0.87	4.8E-02	filamin A interacting protein 1 [Source:MGI Symbol;Acc:MGI:1917848]
Bend4	0.87	3.8E-02	BEN domain containing 4 [Source:MGI Symbol;Acc:MGI:3648414]
Tie1	0.87	9.4E-04	tyrosine kinase with immunoglobulin-like and EGF-like domains 1 [Source:MGI Symbol;Acc:MGI:99906]
Amph	0.87	2.9E-02	amphiphysin [Source:MGI Symbol;Acc:MGI:103574]
Dclre1c	0.86	4.6E-03	DNA cross-link repair 1C, PSO2 homolog (S. cerevisiae) [Source:MGI Symbol;Acc:MGI:2441769]
Nmnat2	0.86	4.0E-02	nicotinamide nucleotide adenylyltransferase 2 [Source:MGI Symbol;Acc:MGI:2444155]
Cd34	0.86	1.1E-02	CD34 antigen [Source:MGI Symbol;Acc:MGI:88329]
Notch3	0.86	3.2E-02	notch 3 [Source:MGI Symbol;Acc:MGI:99460]
Cacna1a	0.86	6.6E-03	calcium channel, voltage-dependent, P/Q type, alpha 1A subunit [Source:MGI Symbol;Acc:MGI:109482]
Slc8a1	0.86	2.8E-03	solute carrier family 8 (sodium/calcium exchanger), member 1 [Source:MGI Symbol;Acc:MGI:107956]

Kank3	0.86	1.2E-02	KN motif and ankyrin repeat domains 3 [Source:MGI Symbol;Acc:MGI:1098615]
Klhl24	0.86	1.8E-02	kelch-like 24 [Source:MGI Symbol;Acc:MGI:1923035]
B230118H07Rik	0.86	6.2E-03	RIKEN cDNA B230118H07 gene [Source:MGI Symbol;Acc:MGI:1915420]
Prkcdbp	0.86	1.3E-02	protein kinase C, delta binding protein [Source:MGI Symbol;Acc:MGI:1923422]
Lphn1	0.86	2.8E-03	latrophilin 1 [Source:MGI Symbol;Acc:MGI:1929461]
Leprel1	0.86	1.6E-02	leprecan-like 1 [Source:MGI Symbol;Acc:MGI:2146663]
Prkacb	0.86	5.2E-04	protein kinase, cAMP dependent, catalytic, beta [Source:MGI Symbol;Acc:MGI:97594]
Rbpms	0.86	1.0E-02	RNA binding protein gene with multiple splicing [Source:MGI Symbol;Acc:MGI:1334446]
Sphk1	0.86	3.9E-02	sphingosine kinase 1 [Source:MGI Symbol;Acc:MGI:1316649]
Itgb8	0.86	3.4E-02	integrin beta 8 [Source:MGI Symbol;Acc:MGI:1338035]
Leprel2	0.86	9.7E-03	leprecan-like 2 [Source:MGI Symbol;Acc:MGI:1315208]
Efemp1	0.86	2.9E-05	epidermal growth factor-containing fibulin-like extracellular matrix protein 1 [Source:MGI Symbol;Acc:MGI:1339998]
Leprel4	0.86	2.2E-02	leprecan-like 4 [Source:MGI Symbol;Acc:MGI:1913430]
Trim47	0.85	3.3E-03	tripartite motif-containing 47 [Source:MGI Symbol;Acc:MGI:1917374]
Ghr	0.85	3.3E-04	growth hormone receptor [Source:MGI Symbol;Acc:MGI:95708]
Mageh1	0.85	2.4E-02	melanoma antigen, family H, 1 [Source:MGI Symbol;Acc:MGI:1922875]
Rgs4	0.85	4.6E-02	regulator of G-protein signaling 4 [Source:MGI Symbol;Acc:MGI:108409]
Tuba1a	0.85	1.7E-02	tubulin, alpha 1A [Source:MGI Symbol;Acc:MGI:98869]
Il1rap	0.85	8.5E-03	interleukin 1 receptor accessory protein [Source:MGI Symbol;Acc:MGI:104975]
Podxl	0.85	3.0E-03	podocalyxin-like [Source:MGI Symbol;Acc:MGI:1351317]
Fzd1	0.85	1.1E-02	frizzled homolog 1 (Drosophila) [Source:MGI Symbol;Acc:MGI:1196625]
Kcnab2	0.85	3.8E-03	potassium voltage-gated channel, shaker-related subfamily, beta member 2 [Source:MGI Symbol;Acc:MGI:109239]
Coro1a	0.85	4.8E-06	coronin, actin binding protein 1A [Source:MGI Symbol;Acc:MGI:1345961]
Cxx1a	0.85	4.1E-02	CAAX box 1A [Source:MGI Symbol;Acc:MGI:1913408]
Zfp874a	0.85	4.7E-02	zinc finger protein 874a [Source:MGI Symbol;Acc:MGI:3040703]
Trim44	0.85	9.2E-04	tripartite motif-containing 44 [Source:MGI Symbol;Acc:MGI:1931835]
Itga7	0.85	1.2E-04	integrin alpha 7 [Source:MGI Symbol;Acc:MGI:102700]
Tnfaip8l2	0.84	1.5E-02	tumor necrosis factor, alpha-induced protein 8-like 2 [Source:MGI Symbol;Acc:MGI:1917019]
Glis2	0.84	1.8E-02	GLIS family zinc finger 2 [Source:MGI Symbol;Acc:MGI:1932535]
Hdac5	0.84	4.7E-03	histone deacetylase 5 [Source:MGI Symbol;Acc:MGI:1333784]
Ttll7	0.84	2.8E-02	tubulin tyrosine ligase-like family, member 7 [Source:MGI Symbol;Acc:MGI:1918142]
Dysf	0.84	3.3E-02	dysferlin [Source:MGI Symbol;Acc:MGI:1349385]
Cry2	0.84	2.0E-02	cryptochrome 2 (photolyase-like) [Source:MGI Symbol;Acc:MGI:1270859]
Cbx6	0.84	2.2E-03	chromobox 6 [Source:MGI Symbol;Acc:MGI:3512628]
Zfr2	0.84	2.7E-02	zinc finger RNA binding protein 2 [Source:MGI Symbol;Acc:MGI:2143792]
Hoxd9	0.84	1.5E-02	homeobox D9 [Source:MGI Symbol;Acc:MGI:96210]
Map1b	0.84	4.7E-02	microtubule-associated protein 1B [Source:MGI Symbol;Acc:MGI:1306778]
Cpm	0.84	4.3E-02	carboxypeptidase M [Source:MGI Symbol;Acc:MGI:1917824]
Usp35	0.84	1.7E-02	ubiquitin specific peptidase 35 [Source:MGI Symbol;Acc:MGI:2685339]
Ikbip	0.84	3.2E-03	IKBKB interacting protein [Source:MGI Symbol;Acc:MGI:1914704]
Dab2	0.84	1.6E-02	disabled 2, mitogen-responsive phosphoprotein [Source:MGI Symbol;Acc:MGI:109175]
Notch2	0.84	3.0E-04	notch 2 [Source:MGI Symbol;Acc:MGI:97364]
Epm2a	0.84	3.9E-02	epilepsy, progressive myoclonic epilepsy, type 2 gene alpha [Source:MGI Symbol;Acc:MGI:1341085]
Sfxn5	0.84	8.6E-03	sideroflexin 5 [Source:MGI Symbol;Acc:MGI:2137681]
Gm16576	0.84	4.7E-02	predicted gene 16576 [Source:MGI Symbol;Acc:MGI:4414996]
Col6a1	0.83	1.9E-05	collagen, type VI, alpha 1 [Source:MGI Symbol;Acc:MGI:88459]
Nfatc2	0.83	8.6E-04	nuclear factor of activated T cells, cytoplasmic, calcineurin dependent 2 [Source:MGI Symbol;Acc:MGI:102463]
Pde2a	0.83	4.0E-02	phosphodiesterase 2A, cGMP-stimulated [Source:MGI Symbol;Acc:MGI:2446107]
Csf3r	0.83	2.3E-02	colony stimulating factor 3 receptor (granulocyte) [Source:MGI Symbol;Acc:MGI:1339755]
Mylip	0.83	3.4E-03	myosin regulatory light chain interacting protein [Source:MGI Symbol;Acc:MGI:2388271]
Ramp2	0.83	8.9E-03	receptor (calcitonin) activity modifying protein 2 [Source:MGI Symbol;Acc:MGI:1859650]
Mmp23	0.83	2.9E-02	matrix metallopeptidase 23 [Source:MGI Symbol;Acc:MGI:1347361]
Plxna3	0.83	2.2E-02	plexin A3 [Source:MGI Symbol;Acc:MGI:107683]

Nr1d2	0.83	9.6E-03	nuclear receptor subfamily 1, group D, member 2 [Source:MGI Symbol;Acc:MGI:2449205]
Ms4a4d	0.83	1.9E-02	membrane-spanning 4-domains, subfamily A, member 4D [Source:MGI Symbol;Acc:MGI:1913857]
Plcg2	0.83	1.3E-04	phospholipase C, gamma 2 [Source:MGI Symbol;Acc:MGI:97616]
Zfp69	0.83	5.0E-02	zinc finger protein 69 [Source:MGI Symbol;Acc:MGI:107794]
Tril	0.83	4.2E-03	TLR4 interactor with leucine-rich repeats [Source:MGI Symbol;Acc:MGI:1914123]
Col5a3	0.83	1.4E-02	collagen, type V, alpha 3 [Source:MGI Symbol;Acc:MGI:1858212]
Hsdl1	0.83	4.0E-04	hydroxysteroid dehydrogenase like 1 [Source:MGI Symbol;Acc:MGI:1919802]
Fam43a	0.83	1.4E-02	family with sequence similarity 43, member A [Source:MGI Symbol;Acc:MGI:2676309]
Arhgef17	0.83	2.0E-02	Rho guanine nucleotide exchange factor (GEF) 17 [Source:MGI Symbol;Acc:MGI:2673002]
1700021K19Rik	0.82	2.5E-05	RIKEN cDNA 1700021K19 gene [Source:MGI Symbol;Acc:MGI:1915160]
Dnal1	0.82	3.7E-02	dynein, axonemal, light chain 1 [Source:MGI Symbol;Acc:MGI:1921462]
Rab34	0.82	1.3E-02	RAB34, member RAS oncogene family [Source:MGI Symbol;Acc:MGI:104606]
Mark1	0.82	1.3E-02	MAP/microtubule affinity-regulating kinase 1 [Source:MGI Symbol;Acc:MGI:2664902]
Msrb3	0.82	9.3E-03	methionine sulfoxide reductase B3 [Source:MGI Symbol;Acc:MGI:2443538]
Lcp1	0.82	8.7E-05	lymphocyte cytosolic protein 1 [Source:MGI Symbol;Acc:MGI:104808]
Ogfrl1	0.82	6.4E-03	opioid growth factor receptor-like 1 [Source:MGI Symbol;Acc:MGI:1917405]
Sepn1	0.82	8.3E-03	selenoprotein N, 1 [Source:MGI Symbol;Acc:MGI:2151208]
Tshz3	0.82	2.2E-02	teashirt zinc finger family member 3 [Source:MGI Symbol;Acc:MGI:2442819]
Bcl2	0.82	1.3E-04	B cell leukemia/lymphoma 2 [Source:MGI Symbol;Acc:MGI:88138]
Camsap2	0.82	1.2E-02	calmodulin regulated spectrin-associated protein family, member 2 [Source:MGI Symbol;Acc:MGI:1922434]
Palld	0.81	5.2E-03	palladin, cytoskeletal associated protein [Source:MGI Symbol;Acc:MGI:1919583]
Col12a1	0.81	1.0E-02	collagen, type XII, alpha 1 [Source:MGI Symbol;Acc:MGI:88448]
Ano1	0.81	9.6E-03	anoctamin 1, calcium activated chloride channel [Source:MGI Symbol;Acc:MGI:2142149]
Fscn1	0.81	1.3E-03	fascin homolog 1, actin bundling protein (Strongylocentrotus purpuratus) [Source:MGI Symbol;Acc:MGI:1352745]
Fermt2	0.81	2.0E-02	fermitin family homolog 2 (Drosophila) [Source:MGI Symbol;Acc:MGI:2385001]
Tmem132a	0.81	2.7E-02	transmembrane protein 132A [Source:MGI Symbol;Acc:MGI:2147810]
5730409E04Rik	0.81	2.0E-02	RIKEN cDNA 5730409E04Rik gene [Source:MGI Symbol;Acc:MGI:3609248]
Ppm1f	0.81	7.9E-03	protein phosphatase 1F (PP2C domain containing) [Source:MGI Symbol;Acc:MGI:1918464]
Erg	0.81	2.2E-02	avian erythroblastosis virus E-26 (v-ets) oncogene related [Source:MGI Symbol;Acc:MGI:95415]
Kcnj8	0.81	4.4E-02	potassium inwardly-rectifying channel, subfamily J, member 8 [Source:MGI Symbol;Acc:MGI:1100508]
Dzip1l	0.81	4.2E-02	DAZ interacting protein 1-like [Source:MGI Symbol;Acc:MGI:1919757]
Mcam	0.81	1.4E-02	melanoma cell adhesion molecule [Source:MGI Symbol;Acc:MGI:1933966]
Sh2b3	0.81	8.9E-04	SH2B adaptor protein 3 [Source:MGI Symbol;Acc:MGI:893598]
Fmnl1	0.80	9.7E-04	formin-like 1 [Source:MGI Symbol;Acc:MGI:1888994]
S1pr1	0.80	2.1E-02	sphingosine-1-phosphate receptor 1 [Source:MGI Symbol;Acc:MGI:1096355]
Id4	0.80	2.1E-02	inhibitor of DNA binding 4 [Source:MGI Symbol;Acc:MGI:99414]
Hsd11b1	0.80	4.4E-02	hydroxysteroid 11-beta dehydrogenase 1 [Source:MGI Symbol;Acc:MGI:103562]
Stx2	0.80	3.3E-02	syntaxin 2 [Source:MGI Symbol;Acc:MGI:108059]
Cacna2d2	0.80	4.9E-02	calcium channel, voltage-dependent, alpha 2/delta subunit 2 [Source:MGI Symbol;Acc:MGI:1929813]
Aldh6a1	0.80	2.5E-03	aldehyde dehydrogenase family 6, subfamily A1 [Source:MGI Symbol;Acc:MGI:1915077]
Nrros	0.80	1.7E-03	negative regulator of reactive oxygen species [Source:MGI Symbol;Acc:MGI:2445095]
Lppr2	0.80	4.1E-02	lipid phosphate phosphatase-related protein type 2 [Source:MGI Symbol;Acc:MGI:2384575]
Hoxd3	0.80	4.1E-02	homeobox D3 [Source:MGI Symbol;Acc:MGI:96207]
Phldb2	0.80	6.7E-03	pleckstrin homology-like domain, family B, member 2 [Source:MGI Symbol;Acc:MGI:2444981]
Cat	0.80	5.4E-04	catalase [Source:MGI Symbol;Acc:MGI:88271]
Ajuba	0.80	4.1E-02	ajuba LIM protein [Source:MGI Symbol;Acc:MGI:1341886]
Hcar1	0.80	1.4E-02	hydrocarboxylic acid receptor 1 [Source:MGI Symbol;Acc:MGI:2441671]
Dennd5a	0.80	2.0E-03	DENN/MADD domain containing 5A [Source:MGI Symbol;Acc:MGI:1201681]
Zbtb20	0.80	6.6E-04	zinc finger and BTB domain containing 20 [Source:MGI Symbol;Acc:MGI:1929213]
Mylk	0.80	4.4E-02	myosin, light polypeptide kinase [Source:MGI Symbol;Acc:MGI:894806]

Ctsk	0.80	2.4E-02	cathepsin K [Source:MGI Symbol;Acc:MGI:107823]
Pdlim7	0.80	3.7E-02	PDZ and LIM domain 7 [Source:MGI Symbol;Acc:MGI:1914649]
Rapgef5	0.80	1.8E-02	Rap guanine nucleotide exchange factor (GEF) 5 [Source:MGI Symbol;Acc:MGI:2444365]
Pfkfb3	0.80	3.3E-03	6-phosphofructo-2-kinase/fructose-2,6-biphosphatase 3 [Source:MGI Symbol;Acc:MGI:2181202]
Grik5	0.79	2.6E-02	glutamate receptor, ionotropic, kainate 5 (gamma 2) [Source:MGI Symbol;Acc:MGI:95818]
Ndrg1	0.79	2.3E-04	N-myc downstream regulated gene 1 [Source:MGI Symbol;Acc:MGI:1341799]
Ablim2	0.79	2.6E-02	actin-binding LIM protein 2 [Source:MGI Symbol;Acc:MGI:2385758]
Hlx	0.79	2.2E-02	H2.0-like homeobox [Source:MGI Symbol;Acc:MGI:96109]
Dmwd	0.79	3.7E-02	dystrophia myotonica-containing WD repeat motif [Source:MGI Symbol;Acc:MGI:94907]
Stc2	0.79	1.2E-02	stanniocalcin 2 [Source:MGI Symbol;Acc:MGI:1316731]
Iffo1	0.79	1.2E-02	intermediate filament family orphan 1 [Source:MGI Symbol;Acc:MGI:2444516]
Cda	0.79	2.1E-02	cytidine deaminase [Source:MGI Symbol;Acc:MGI:1919519]
Cygb	0.79	8.8E-04	cytoglobin [Source:MGI Symbol;Acc:MGI:2149481]
A330023F24Rik	0.79	4.5E-02	RIKEN cDNA A330023F24 gene [Source:MGI Symbol;Acc:MGI:2443958]
Ptpn1	0.79	2.0E-06	protein tyrosine phosphatase, non-receptor type 1 [Source:MGI Symbol;Acc:MGI:97805]
Syde1	0.79	1.5E-02	synapse defective 1, Rho GTPase, homolog 1 (C. elegans) [Source:MGI Symbol;Acc:MGI:1918959]
Pid1	0.79	3.9E-02	phosphotyrosine interaction domain containing 1 [Source:MGI Symbol;Acc:MGI:2138391]
Mr1	0.79	4.4E-02	major histocompatibility complex, class I-related [Source:MGI Symbol;Acc:MGI:1195463]
Hyi	0.79	2.0E-02	hydroxypyruvate isomerase homolog (E. coli) [Source:MGI Symbol;Acc:MGI:1915430]
Sfxn3	0.79	5.8E-03	sideroflexin 3 [Source:MGI Symbol;Acc:MGI:2137679]
Per1	0.78	6.9E-03	period circadian clock 1 [Source:MGI Symbol;Acc:MGI:1098283]
Akna	0.78	6.1E-04	AT-hook transcription factor [Source:MGI Symbol;Acc:MGI:2140340]
Atoh8	0.78	2.2E-02	atonal homolog 8 (Drosophila) [Source:MGI Symbol;Acc:MGI:1918343]
Hexb	0.78	8.5E-04	hexosaminidase B [Source:MGI Symbol;Acc:MGI:96074]
Cdr2	0.78	1.2E-02	cerebellar degeneration-related 2 [Source:MGI Symbol;Acc:MGI:1100885]
Tacc1	0.78	3.3E-03	transforming, acidic coiled-coil containing protein 1 [Source:MGI Symbol;Acc:MGI:2443510]
Cbr3	0.78	3.0E-02	carbonyl reductase 3 [Source:MGI Symbol;Acc:MGI:1309992]
Lifr	0.78	8.4E-03	leukemia inhibitory factor receptor [Source:MGI Symbol;Acc:MGI:96788]
Slc16a9	0.78	4.4E-02	solute carrier family 16 (monocarboxylic acid transporters), member 9 [Source:MGI Symbol;Acc:MGI:1914109]
Hdac11	0.78	4.6E-02	histone deacetylase 11 [Source:MGI Symbol;Acc:MGI:2385252]
Pald1	0.78	2.9E-02	phosphatase domain containing, paladin 1 [Source:MGI Symbol;Acc:MGI:1351623]
Sun2	0.77	6.6E-04	Sad1 and UNC84 domain containing 2 [Source:MGI Symbol;Acc:MGI:2443011]
Pgap2	0.77	1.5E-06	post-GPI attachment to proteins 2 [Source:MGI Symbol;Acc:MGI:2385286]
Dip2c	0.77	5.2E-03	DIP2 disco-interacting protein 2 homolog C (Drosophila) [Source:MGI Symbol;Acc:MGI:1920179]
Abca9	0.77	3.2E-02	ATP-binding cassette, sub-family A (ABC1), member 9 [Source:MGI Symbol;Acc:MGI:2386796]
Tnc	0.77	2.0E-04	tenascin C [Source:MGI Symbol;Acc:MGI:101922]
Tbc1d8	0.77	3.7E-03	TBC1 domain family, member 8 [Source:MGI Symbol;Acc:MGI:1927225]
Acer2	0.77	2.6E-02	alkaline ceramidase 2 [Source:MGI Symbol;Acc:MGI:1920932]
Pacsin1	0.77	4.8E-02	protein kinase C and casein kinase substrate in neurons 1 [Source:MGI Symbol;Acc:MGI:1345181]
1110051M20Rik	0.77	2.7E-02	RIKEN cDNA 1110051M20 gene [Source:MGI Symbol;Acc:MGI:1915079]
Tnfrsf13b	0.77	1.2E-02	tumor necrosis factor receptor superfamily, member 13b [Source:MGI Symbol;Acc:MGI:1889411]
Gpam	0.76	1.4E-02	glycerol-3-phosphate acyltransferase, mitochondrial [Source:MGI Symbol;Acc:MGI:109162]
Klhl5	0.76	6.7E-04	kelch-like 5 [Source:MGI Symbol;Acc:MGI:1919028]
Pik3cd	0.76	1.5E-03	phosphatidylinositol 3-kinase catalytic delta polypeptide [Source:MGI Symbol;Acc:MGI:1098211]
P2ry6	0.76	2.9E-03	pyrimidinergic receptor P2Y, G-protein coupled, 6 [Source:MGI Symbol;Acc:MGI:2673874]
Hspa1b	0.76	3.2E-03	heat shock protein 1B [Source:MGI Symbol;Acc:MGI:99517]
Tspan33	0.76	4.4E-02	tetraspanin 33 [Source:MGI Symbol;Acc:MGI:1919012]
Zdhhc8	0.76	9.8E-03	zinc finger, DHHC domain containing 8 [Source:MGI Symbol;Acc:MGI:1338012]

Col6a3	0.76	2.4E-03	collagen, type VI, alpha 3 [Source:MGI Symbol;Acc:MGI:88461]
Eva1b	0.76	2.1E-02	eva-1 homolog B (C. elegans) [Source:MGI Symbol;Acc:MGI:1922063]
Cdh11	0.76	1.3E-02	cadherin 11 [Source:MGI Symbol;Acc:MGI:99217]
Pcolce	0.76	2.4E-02	procollagen C-endopeptidase enhancer protein [Source:MGI Symbol;Acc:MGI:105099]
Amotl2	0.76	4.6E-03	angiomotin-like 2 [Source:MGI Symbol;Acc:MGI:1929286]
Pkd2	0.76	1.2E-03	polycystic kidney disease 2 [Source:MGI Symbol;Acc:MGI:1099818]
Lphn3	0.76	4.0E-02	latrophilin 3 [Source:MGI Symbol;Acc:MGI:2441950]
Cbfa2t3	0.75	1.8E-02	core-binding factor, runt domain, alpha subunit 2, translocated to, 3 (human) [Source:MGI Symbol;Acc:MGI:1338013]
Slc35e3	0.75	7.5E-03	solute carrier family 35, member E3 [Source:MGI Symbol;Acc:MGI:2448489]
Dhrs3	0.75	2.5E-05	dehydrogenase/reductase (SDR family) member 3 [Source:MGI Symbol;Acc:MGI:1315215]
Dync2h1	0.75	3.4E-02	dynein cytoplasmic 2 heavy chain 1 [Source:MGI Symbol;Acc:MGI:107736]
Itpkb	0.75	1.8E-03	inositol 1,4,5-trisphosphate 3-kinase B [Source:MGI Symbol;Acc:MGI:109235]
Setd7	0.75	5.9E-03	SET domain containing (lysine methyltransferase) 7 [Source:MGI Symbol;Acc:MGI:1920501]
Gpr116	0.75	9.8E-03	G protein-coupled receptor 116 [Source:MGI Symbol;Acc:MGI:2182928]
Klhl6	0.75	4.6E-02	kelch-like 6 [Source:MGI Symbol;Acc:MGI:2686922]
Prune2	0.75	3.7E-02	prune homolog 2 (Drosophila) [Source:MGI Symbol;Acc:MGI:1925004]
Reep5	0.75	9.7E-04	receptor accessory protein 5 [Source:MGI Symbol;Acc:MGI:1270152]
Nlgn2	0.75	2.2E-02	neuroligin 2 [Source:MGI Symbol;Acc:MGI:2681835]
Aldh3b1	0.75	7.7E-04	aldehyde dehydrogenase 3 family, member B1 [Source:MGI Symbol;Acc:MGI:1914939]
Slc48a1	0.74	1.5E-04	solute carrier family 48 (heme transporter), member 1 [Source:MGI Symbol;Acc:MGI:1914989]
Pcdh7	0.74	2.9E-02	protocadherin 7 [Source:MGI Symbol;Acc:MGI:1860487]
Anks6	0.74	2.2E-02	ankyrin repeat and sterile alpha motif domain containing 6 [Source:MGI Symbol;Acc:MGI:1922941]
Il17re	0.74	2.7E-02	interleukin 17 receptor E [Source:MGI Symbol;Acc:MGI:1889371]
Tgfbr2	0.74	4.4E-03	transforming growth factor, beta receptor II [Source:MGI Symbol;Acc:MGI:98729]
Cdyl2	0.74	3.3E-02	chromodomain protein, Y chromosome-like 2 [Source:MGI Symbol;Acc:MGI:1923046]
Ralgps1	0.74	8.1E-03	Ral GEF with PH domain and SH3 binding motif 1 [Source:MGI Symbol;Acc:MGI:1922008]
Rarg	0.74	6.2E-03	retinoic acid receptor, gamma [Source:MGI Symbol;Acc:MGI:97858]
Lancl1	0.74	2.3E-03	LanC (bacterial lantibiotic synthetase component C)-like 1 [Source:MGI Symbol;Acc:MGI:1336997]
Ptger3	0.74	3.5E-02	prostaglandin E receptor 3 (subtype EP3) [Source:MGI Symbol;Acc:MGI:97795]
Ptprb	0.74	6.2E-03	protein tyrosine phosphatase, receptor type, B [Source:MGI Symbol;Acc:MGI:97809]
Ccdc157	0.74	1.9E-02	coiled-coil domain containing 157 [Source:MGI Symbol;Acc:MGI:3041210]
Prex2	0.74	7.7E-03	phosphatidylinositol-3,4,5-trisphosphate-dependent Rac exchange factor 2 [Source:MGI Symbol;Acc:MGI:1923385]
Emilin1	0.73	1.1E-02	elastin microfibril interfacer 1 [Source:MGI Symbol;Acc:MGI:1926189]
Klhl36	0.73	1.7E-02	kelch-like 36 [Source:MGI Symbol;Acc:MGI:2385305]
Zfp395	0.73	2.6E-02	zinc finger protein 395 [Source:MGI Symbol;Acc:MGI:2682318]
Nipa1	0.73	4.8E-02	non imprinted in Prader-Willi/Angelman syndrome 1 homolog (human) [Source:MGI Symbol;Acc:MGI:2442058]
Rftn1	0.73	2.8E-03	raftlin lipid raft linker 1 [Source:MGI Symbol;Acc:MGI:1923688]
Tmem176a	0.73	2.0E-05	transmembrane protein 176A [Source:MGI Symbol;Acc:MGI:1913308]
Palm	0.73	1.4E-02	paralemmin [Source:MGI Symbol;Acc:MGI:1261814]
Cd48	0.73	9.8E-03	CD48 antigen [Source:MGI Symbol;Acc:MGI:88339]
Klf2	0.73	2.9E-02	Kruppel-like factor 2 (lung) [Source:MGI Symbol;Acc:MGI:1342772]
Trim32	0.73	1.4E-02	tripartite motif-containing 32 [Source:MGI Symbol;Acc:MGI:1917057]
Prkab2	0.73	1.7E-02	protein kinase, AMP-activated, beta 2 non-catalytic subunit [Source:MGI Symbol;Acc:MGI:1336185]
Slc45a4	0.73	9.0E-04	solute carrier family 45, member 4 [Source:MGI Symbol;Acc:MGI:2146236]
Armcx3	0.73	1.9E-02	armadillo repeat containing, X-linked 3 [Source:MGI Symbol;Acc:MGI:1918953]
Itm2b	0.73	7.8E-03	integral membrane protein 2B [Source:MGI Symbol;Acc:MGI:1309517]
Gmip	0.73	5.4E-04	Gem-interacting protein [Source:MGI Symbol;Acc:MGI:1926066]
Fzd8	0.72	2.6E-02	frizzled homolog 8 (Drosophila) [Source:MGI Symbol;Acc:MGI:108460]
2410089E03Rik	0.72	6.0E-03	RIKEN cDNA 2410089E03 gene [Source:MGI Symbol;Acc:MGI:1920942]
Plxnc1	0.72	4.0E-02	plexin C1 [Source:MGI Symbol;Acc:MGI:1890127]
Hoxd8	0.72	2.5E-02	homeobox D8 [Source:MGI Symbol;Acc:MGI:96209]
Dkk3	0.72	1.8E-02	dickkopf homolog 3 (Xenopus laevis) [Source:MGI Symbol;Acc:MGI:1354952]

Lix1l	0.72	3.6E-02	Lix1-like [Source:MGI Symbol;Acc:MGI:3036267]
Bcl6	0.72	7.8E-03	B cell leukemia/lymphoma 6 [Source:MGI Symbol;Acc:MGI:107187]
Col27a1	0.72	2.4E-02	collagen, type XXVII, alpha 1 [Source:MGI Symbol;Acc:MGI:2672118]
Fkbp10	0.72	4.5E-02	FK506 binding protein 10 [Source:MGI Symbol;Acc:MGI:104769]
Proser2	0.72	3.4E-02	proline and serine rich 2 [Source:MGI Symbol;Acc:MGI:2442238]
Sorbs2	0.72	8.2E-03	sorbin and SH3 domain containing 2 [Source:MGI Symbol;Acc:MGI:1924574]
Mmp2	0.71	9.3E-04	matrix metallopeptidase 2 [Source:MGI Symbol;Acc:MGI:97009]
Pxdn	0.71	9.8E-03	peroxidasin homolog (Drosophila) [Source:MGI Symbol;Acc:MGI:1916925]
Six5	0.71	3.3E-02	sine oculis-related homeobox 5 [Source:MGI Symbol;Acc:MGI:106220]
Dclk1	0.71	1.1E-02	doublecortin-like kinase 1 [Source:MGI Symbol;Acc:MGI:1330861]
Emcn	0.71	3.8E-02	endomucin [Source:MGI Symbol;Acc:MGI:1891716]
Pbx3	0.71	3.5E-02	pre B cell leukemia homeobox 3 [Source:MGI Symbol;Acc:MGI:97496]
Cotl1	0.71	8.6E-05	coactosin-like 1 (Dictyostelium) [Source:MGI Symbol;Acc:MGI:1919292]
Cc2d2a	0.71	1.6E-02	coiled-coil and C2 domain containing 2A [Source:MGI Symbol;Acc:MGI:1924487]
Bace1	0.71	2.9E-02	beta-site APP cleaving enzyme 1 [Source:MGI Symbol;Acc:MGI:1346542]
Xrcc1	0.71	9.3E-04	X-ray repair complementing defective repair in Chinese hamster cells 1 [Source:MGI Symbol;Acc:MGI:99137]
Lats2	0.71	1.8E-04	large tumor suppressor 2 [Source:MGI Symbol;Acc:MGI:1354386]
Dpysl3	0.71	1.1E-02	dihydropyrimidinase-like 3 [Source:MGI Symbol;Acc:MGI:1349762]
Rcn3	0.70	4.4E-02	reticulocalbin 3, EF-hand calcium binding domain [Source:MGI Symbol;Acc:MGI:1277122]
Traf5	0.70	2.7E-02	TNF receptor-associated factor 5 [Source:MGI Symbol;Acc:MGI:107548]
Adam15	0.70	5.5E-04	a disintegrin and metallopeptidase domain 15 (metargidin) [Source:MGI Symbol;Acc:MGI:1333882]
Nrp1	0.70	5.2E-03	neuropilin 1 [Source:MGI Symbol;Acc:MGI:106206]
Cdh5	0.70	1.8E-02	cadherin 5 [Source:MGI Symbol;Acc:MGI:105057]
Dusp3	0.70	4.9E-03	dual specificity phosphatase 3 (vaccinia virus phosphatase VH1-related) [Source:MGI Symbol;Acc:MGI:1919599]
Sdc2	0.70	2.0E-03	syndecan 2 [Source:MGI Symbol;Acc:MGI:1349165]
Rsu1	0.70	1.6E-03	Ras suppressor protein 1 [Source:MGI Symbol;Acc:MGI:103040]
Pros1	0.70	2.0E-03	protein S (alpha) [Source:MGI Symbol;Acc:MGI:1095733]
Tlr1	0.70	2.9E-04	toll-like receptor 1 [Source:MGI Symbol;Acc:MGI:1341295]
Invs	0.70	2.6E-02	inversin [Source:MGI Symbol;Acc:MGI:1335082]
Ulk2	0.70	1.4E-03	unc-51 like kinase 2 [Source:MGI Symbol;Acc:MGI:1352758]
Ano6	0.69	5.5E-03	anoctamin 6 [Source:MGI Symbol;Acc:MGI:2145890]
Sema3f	0.69	2.8E-03	sema domain, immunoglobulin domain (Ig), short basic domain, secreted, (semaphorin) 3F [Source:MGI Symbol;Acc:MGI:1096347]
Nav1	0.69	1.9E-03	neuron navigator 1 [Source:MGI Symbol;Acc:MGI:2183683]
Pold4	0.69	4.4E-03	polymerase (DNA-directed), delta 4 [Source:MGI Symbol;Acc:MGI:1916995]
Syk	0.69	5.4E-04	spleen tyrosine kinase [Source:MGI Symbol;Acc:MGI:99515]
Ago4	0.69	4.0E-02	argonaute RISC catalytic subunit 4 [Source:MGI Symbol;Acc:MGI:1924100]
Tsc22d3	0.69	1.1E-02	TSC22 domain family, member 3 [Source:MGI Symbol;Acc:MGI:1196284]
Bsn	0.69	4.5E-02	bassoon [Source:MGI Symbol;Acc:MGI:1277955]
Sh3bgrl	0.69	1.2E-03	SH3-binding domain glutamic acid-rich protein like [Source:MGI Symbol;Acc:MGI:1930849]
Plod1	0.69	7.2E-03	procollagen-lysine, 2-oxoglutarate 5-dioxygenase 1 [Source:MGI Symbol;Acc:MGI:99907]
Blvrb	0.69	5.2E-04	biliverdin reductase B (flavin reductase (NADPH)) [Source:MGI Symbol;Acc:MGI:2385271]
Rps6ka2	0.69	4.8E-02	ribosomal protein S6 kinase, polypeptide 2 [Source:MGI Symbol;Acc:MGI:1342290]
Fendrr	0.69	1.2E-02	Foxf1 adjacent non-coding developmental regulatory RNA [Source:MGI Symbol;Acc:MGI:1916040]
D930015E06Rik	0.69	2.0E-02	RIKEN cDNA D930015E06 gene [Source:MGI Symbol;Acc:MGI:2443399]
Grn	0.69	4.5E-04	granulin [Source:MGI Symbol;Acc:MGI:95832]
Elk1	0.69	2.2E-02	ELK1, member of ETS oncogene family [Source:MGI Symbol;Acc:MGI:101833]
Pdzd2	0.69	8.2E-03	PDZ domain containing 2 [Source:MGI Symbol;Acc:MGI:1922394]
Arl3	0.69	4.6E-02	ADP-ribosylation factor-like 3 [Source:MGI Symbol;Acc:MGI:1929699]
Ddit3	0.69	2.3E-02	DNA-damage inducible transcript 3 [Source:MGI Symbol;Acc:MGI:109247]
Col6a2	0.69	6.8E-04	collagen, type VI, alpha 2 [Source:MGI Symbol;Acc:MGI:88460]
Tmem134	0.69	3.7E-04	transmembrane protein 134 [Source:MGI Symbol;Acc:MGI:1914240]
Icam1	0.68	1.6E-02	intercellular adhesion molecule 1 [Source:MGI Symbol;Acc:MGI:96392]
Slc12a9	0.68	7.3E-03	solute carrier family 12 (potassium/chloride transporters), member 9 [Source:MGI Symbol;Acc:MGI:1933532]
Rarres2	0.68	1.4E-02	retinoic acid receptor responder (tazarotene induced) 2 [Source:MGI Symbol;Acc:MGI:1918910]

Igfbp4	0.68	2.2E-02	insulin-like growth factor binding protein 4 [Source:MGI Symbol;Acc:MGI:96439]
Rhoj	0.68	2.1E-02	ras homolog gene family, member J [Source:MGI Symbol;Acc:MGI:1931551]
Gmfg	0.68	1.8E-03	glia maturation factor, gamma [Source:MGI Symbol;Acc:MGI:1927135]
Igsf10	0.68	1.4E-02	immunoglobulin superfamily, member 10 [Source:MGI Symbol;Acc:MGI:1923481]
Ggta1	0.68	3.5E-02	glycoprotein galactosyltransferase alpha 1, 3 [Source:MGI Symbol;Acc:MGI:95704]
Impact	0.68	2.8E-03	impact, RWD domain protein [Source:MGI Symbol;Acc:MGI:1098233]
Bdkrb2	0.68	2.7E-02	bradykinin receptor, beta 2 [Source:MGI Symbol;Acc:MGI:102845]
Vasn	0.68	5.0E-02	vasorin [Source:MGI Symbol;Acc:MGI:2177651]
Plbd2	0.68	4.6E-04	phospholipase B domain containing 2 [Source:MGI Symbol;Acc:MGI:1919022]
Calcoco1	0.68	1.4E-02	calcium binding and coiled coil domain 1 [Source:MGI Symbol;Acc:MGI:1914738]
Fam129a	0.68	3.2E-02	family with sequence similarity 129, member A [Source:MGI Symbol;Acc:MGI:2137237]
Marcks	0.68	7.5E-05	myristoylated alanine rich protein kinase C substrate [Source:MGI Symbol;Acc:MGI:96907]
Slc9a9	0.68	3.1E-02	solute carrier family 9 (sodium/hydrogen exchanger), member 9 [Source:MGI Symbol;Acc:MGI:2679732]
Ppp1r12c	0.68	1.2E-03	protein phosphatase 1, regulatory (inhibitor) subunit 12C [Source:MGI Symbol;Acc:MGI:1924258]
Gbe1	0.67	4.1E-02	glucan (1,4-alpha-), branching enzyme 1 [Source:MGI Symbol;Acc:MGI:1921435]
Rgs2	0.67	6.0E-03	regulator of G-protein signaling 2 [Source:MGI Symbol;Acc:MGI:1098271]
Pappa	0.67	3.4E-02	pregnancy-associated plasma protein A [Source:MGI Symbol;Acc:MGI:97479]
Gng2	0.67	1.7E-02	guanine nucleotide binding protein (G protein), gamma 2 [Source:MGI Symbol;Acc:MGI:102705]
Zrsr1	0.67	1.7E-02	zinc finger (CCCH type), RNA binding motif and serine/arginine rich 1 [Source:MGI Symbol;Acc:MGI:98885]
Dtx2	0.67	7.0E-03	deltex 2 homolog (Drosophila) [Source:MGI Symbol;Acc:MGI:1921448]
Tirap	0.67	7.9E-03	toll-interleukin 1 receptor (TIR) domain-containing adaptor protein [Source:MGI Symbol;Acc:MGI:2152213]
Nfic	0.67	1.4E-02	nuclear factor I/C [Source:MGI Symbol;Acc:MGI:109591]
BC018473	0.67	1.4E-02	cDNA sequence BC018473 [Source:MGI Symbol;Acc:MGI:3039625]
Ypel3	0.67	4.1E-02	yippee-like 3 (Drosophila) [Source:MGI Symbol;Acc:MGI:1913340]
Ptk7	0.67	4.3E-02	PTK7 protein tyrosine kinase 7 [Source:MGI Symbol;Acc:MGI:1918711]
Spire1	0.67	4.9E-02	spire homolog 1 (Drosophila) [Source:MGI Symbol;Acc:MGI:1915416]
Frmd4a	0.66	3.1E-02	FERM domain containing 4A [Source:MGI Symbol;Acc:MGI:1919850]
Foxf2	0.66	2.2E-02	forkhead box F2 [Source:MGI Symbol;Acc:MGI:1347479]
Rap1gap2	0.66	5.2E-03	RAP1 GTPase activating protein 2 [Source:MGI Symbol;Acc:MGI:3028623]
Lamc1	0.66	9.3E-03	laminin, gamma 1 [Source:MGI Symbol;Acc:MGI:99914]
Ncor2	0.66	4.1E-04	nuclear receptor co-repressor 2 [Source:MGI Symbol;Acc:MGI:1337080]
Cul7	0.66	1.5E-02	cullin 7 [Source:MGI Symbol;Acc:MGI:1913765]
Thbd	0.66	1.9E-02	thrombomodulin [Source:MGI Symbol;Acc:MGI:98736]
Fam219a	0.66	3.0E-02	family with sequence similarity 219, member A [Source:MGI Symbol;Acc:MGI:1919151]
Sall1	0.66	4.1E-02	sal-like 1 (Drosophila) [Source:MGI Symbol;Acc:MGI:1889585]
Atxn7l1	0.66	9.8E-03	ataxin 7-like 1 [Source:MGI Symbol;Acc:MGI:3584458]
Fli1	0.66	2.1E-02	Friend leukemia integration 1 [Source:MGI Symbol;Acc:MGI:95554]
Pde4a	0.66	4.8E-02	phosphodiesterase 4A, cAMP specific [Source:MGI Symbol;Acc:MGI:99558]
2510009E07Rik	0.66	5.9E-03	RIKEN cDNA 2510009E07 gene [Source:MGI Symbol;Acc:MGI:1919440]
Pkd1	0.66	1.4E-03	polycystic kidney disease 1 homolog [Source:MGI Symbol;Acc:MGI:97603]
Cd97	0.65	1.4E-03	CD97 antigen [Source:MGI Symbol;Acc:MGI:1347095]
Flcn	0.65	2.1E-02	folliculin [Source:MGI Symbol;Acc:MGI:2442184]
Oaz2	0.65	4.9E-03	ornithine decarboxylase antizyme 2 [Source:MGI Symbol;Acc:MGI:109492]
Sh3pxd2b	0.65	2.9E-02	SH3 and PX domains 2B [Source:MGI Symbol;Acc:MGI:2442062]
Pip4k2b	0.65	4.4E-03	phosphatidylinositol-5-phosphate 4-kinase, type II, beta [Source:MGI Symbol;Acc:MGI:1934234]
Ptprj	0.65	8.9E-03	protein tyrosine phosphatase, receptor type, J [Source:MGI Symbol;Acc:MGI:104574]
Kifc3	0.65	2.9E-02	kinesin family member C3 [Source:MGI Symbol;Acc:MGI:109202]
Gprasp1	0.65	4.0E-02	G protein-coupled receptor associated sorting protein 1 [Source:MGI Symbol;Acc:MGI:1917418]
Twf2	0.65	5.9E-04	twinfilin, actin-binding protein, homolog 2 (Drosophila) [Source:MGI Symbol;Acc:MGI:1346078]
Gstm1	0.65	3.1E-02	glutathione S-transferase, mu 1 [Source:MGI Symbol;Acc:MGI:95860]
#N/A	0.65	1.2E-02	#N/A
Tmem9	0.64	2.2E-02	transmembrane protein 9 [Source:MGI Symbol;Acc:MGI:1913491]
Rasgef1b	0.64	2.9E-02	RasGEF domain family, member 1B [Source:MGI Symbol;Acc:MGI:2443755]
Pde4b	0.64	4.2E-02	phosphodiesterase 4B, cAMP specific [Source:MGI Symbol;Acc:MGI:99557]

Lyst	0.64	3.1E-02	lysosomal trafficking regulator [Source:MGI Symbol;Acc:MGI:107448]
Acvrl1	0.64	4.1E-03	activin A receptor, type II-like 1 [Source:MGI Symbol;Acc:MGI:1338946]
Samd14	0.64	4.9E-02	sterile alpha motif domain containing 14 [Source:MGI Symbol;Acc:MGI:2384945]
Tmsb4x	0.64	1.3E-02	thymosin, beta 4, X chromosome [Source:MGI Symbol;Acc:MGI:99510]
Itm2c	0.64	1.2E-03	integral membrane protein 2C [Source:MGI Symbol;Acc:MGI:1927594]
Txnip	0.63	2.6E-02	thioredoxin interacting protein [Source:MGI Symbol;Acc:MGI:1889549]
Zdhhc1	0.63	1.8E-02	zinc finger, DHHC domain containing 1 [Source:MGI Symbol;Acc:MGI:1918046]
Rbpj	0.63	9.8E-05	recombination signal binding protein for immunoglobulin kappa J region [Source:MGI Symbol;Acc:MGI:96522]
Rrm2b	0.63	3.3E-02	ribonucleotide reductase M2 B (TP53 inducible) [Source:MGI Symbol;Acc:MGI:2155865]
Snx8	0.63	2.9E-02	sorting nexin 8 [Source:MGI Symbol;Acc:MGI:2443816]
Nid1	0.63	1.6E-02	nidogen 1 [Source:MGI Symbol;Acc:MGI:97342]
Ddx26b	0.63	7.3E-03	DEAD/H (Asp-Glu-Ala-Asp/His) box polypeptide 26B [Source:MGI Symbol;Acc:MGI:2442593]
Dapp1	0.63	4.8E-02	dual adaptor for phosphotyrosine and 3-phosphoinositides 1 [Source:MGI Symbol;Acc:MGI:1347063]
Cyfip2	0.63	2.1E-02	cytoplasmic FMR1 interacting protein 2 [Source:MGI Symbol;Acc:MGI:1924134]
Eng	0.63	2.2E-02	endoglin [Source:MGI Symbol;Acc:MGI:95392]
Zbtb34	0.63	2.3E-02	zinc finger and BTB domain containing 34 [Source:MGI Symbol;Acc:MGI:2685195]
Lacc1	0.63	1.9E-02	laccase (multicopper oxidoreductase) domain containing 1 [Source:MGI Symbol;Acc:MGI:2445077]
Fgfr3	0.63	3.4E-02	fibroblast growth factor receptor 3 [Source:MGI Symbol;Acc:MGI:95524]
Pdgfra	0.63	3.7E-04	platelet derived growth factor receptor, alpha polypeptide [Source:MGI Symbol;Acc:MGI:97530]
Tmx4	0.63	3.3E-02	thioredoxin-related transmembrane protein 4 [Source:MGI Symbol;Acc:MGI:106558]
Amigo1	0.63	2.3E-02	adhesion molecule with Ig like domain 1 [Source:MGI Symbol;Acc:MGI:2653612]
Mllt6	0.63	2.4E-03	myeloid/lymphoid or mixed-lineage leukemia (trithorax homolog, Drosophila); translocated to, 6 [Source:MGI Symbol;Acc:MGI:1935145]
Mpeg1	0.62	2.0E-02	macrophage expressed gene 1 [Source:MGI Symbol;Acc:MGI:1333743]
Bin2	0.62	4.2E-02	bridging integrator 2 [Source:MGI Symbol;Acc:MGI:3611448]
Fgd3	0.62	1.4E-02	FYVE, RhoGEF and PH domain containing 3 [Source:MGI Symbol;Acc:MGI:1353657]
Gaa	0.62	7.4E-03	glucosidase, alpha, acid [Source:MGI Symbol;Acc:MGI:95609]
Ctsf	0.62	1.4E-02	cathepsin F [Source:MGI Symbol;Acc:MGI:1861434]
Fstl1	0.62	4.4E-02	follistatin-like 1 [Source:MGI Symbol;Acc:MGI:102793]
Elk3	0.62	1.1E-02	ELK3, member of ETS oncogene family [Source:MGI Symbol;Acc:MGI:101762]
Sipa1	0.62	8.9E-03	signal-induced proliferation associated gene 1 [Source:MGI Symbol;Acc:MGI:107576]
Col5a1	0.62	7.9E-03	collagen, type V, alpha 1 [Source:MGI Symbol;Acc:MGI:88457]
Ids	0.62	1.8E-03	iduronate 2-sulfatase [Source:MGI Symbol;Acc:MGI:96417]
Gpx3	0.62	3.3E-03	glutathione peroxidase 3 [Source:MGI Symbol;Acc:MGI:105102]
Fam171a1	0.62	3.7E-02	family with sequence similarity 171, member A1 [Source:MGI Symbol;Acc:MGI:2442917]
Col15a1	0.62	3.2E-02	collagen, type XV, alpha 1 [Source:MGI Symbol;Acc:MGI:88449]
Cd81	0.61	6.1E-03	CD81 antigen [Source:MGI Symbol;Acc:MGI:1096398]
Stox2	0.61	2.7E-02	storkhead box 2 [Source:MGI Symbol;Acc:MGI:1918319]
Ift122	0.61	5.0E-02	intraflagellar transport 122 [Source:MGI Symbol;Acc:MGI:1932386]
Notch1	0.61	2.1E-02	notch 1 [Source:MGI Symbol;Acc:MGI:97363]
Cln8	0.61	4.5E-03	ceroid-lipofuscinosis, neuronal 8 [Source:MGI Symbol;Acc:MGI:1349447]
Lhfp	0.61	1.9E-02	lipoma HMGIC fusion partner [Source:MGI Symbol;Acc:MGI:1920048]
Jmy	0.61	1.4E-02	junction-mediating and regulatory protein [Source:MGI Symbol;Acc:MGI:1913096]
Ifi27	0.61	5.9E-04	interferon, alpha-inducible protein 27 [Source:MGI Symbol;Acc:MGI:1277180]
Acadsb	0.60	6.3E-03	acyl-Coenzyme A dehydrogenase, short/branched chain [Source:MGI Symbol;Acc:MGI:1914135]
Ehbp1l1	0.60	1.3E-03	EH domain binding protein 1-like 1 [Source:MGI Symbol;Acc:MGI:3612340]
H6pd	0.60	2.8E-02	hexose-6-phosphate dehydrogenase (glucose 1-dehydrogenase) [Source:MGI Symbol;Acc:MGI:2140356]
Slc16a10	0.60	3.4E-02	solute carrier family 16 (monocarboxylic acid transporters), member 10 [Source:MGI Symbol;Acc:MGI:1919722]
Ggt5	0.60	4.0E-02	gamma-glutamyltransferase 5 [Source:MGI Symbol;Acc:MGI:1346063]
Adamdec1	0.60	4.3E-02	ADAM-like, decysin 1 [Source:MGI Symbol;Acc:MGI:1917650]
Rgs10	0.60	2.3E-02	regulator of G-protein signalling 10 [Source:MGI Symbol;Acc:MGI:1915115]
Ldb1	0.60	1.0E-02	LIM domain binding 1 [Source:MGI Symbol;Acc:MGI:894762]
Ncf2	0.60	2.3E-03	neutrophil cytosolic factor 2 [Source:MGI Symbol;Acc:MGI:97284]

Mef2c	0.60	3.6E-02	myocyte enhancer factor 2C [Source:MGI Symbol;Acc:MGI:99458]
Foxf1	0.60	8.4E-03	forkhead box F1 [Source:MGI Symbol;Acc:MGI:1347470]
Tcp11l2	0.60	4.1E-02	t-complex 11 (mouse) like 2 [Source:MGI Symbol;Acc:MGI:2444679]
Fbrsl1	0.60	6.4E-03	fibrosin-like 1 [Source:MGI Symbol;Acc:MGI:1920907]
Tbc1d5	0.59	1.4E-02	TBC1 domain family, member 5 [Source:MGI Symbol;Acc:MGI:1919488]
Psd3	0.59	1.8E-03	pleckstrin and Sec7 domain containing 3 [Source:MGI Symbol;Acc:MGI:1918215]
Asph	0.59	1.1E-03	aspartate-beta-hydroxylase [Source:MGI Symbol;Acc:MGI:1914186]
Ank	0.59	3.3E-02	progressive ankylosis [Source:MGI Symbol;Acc:MGI:3045421]
Zhx3	0.59	4.3E-02	zinc fingers and homeoboxes 3 [Source:MGI Symbol;Acc:MGI:2444772]
Gabarapl1	0.59	1.2E-02	gamma-aminobutyric acid (GABA) A receptor-associated protein-like 1 [Source:MGI Symbol;Acc:MGI:1914980]
5031439G07Rik	0.59	7.3E-04	RIKEN cDNA 5031439G07 gene [Source:MGI Symbol;Acc:MGI:2444899]
Zfp36l1	0.59	2.5E-03	zinc finger protein 36, C3H type-like 1 [Source:MGI Symbol;Acc:MGI:107946]
Crlf2	0.59	4.0E-02	cytokine receptor-like factor 2 [Source:MGI Symbol;Acc:MGI:1889506]
Mkl1	0.59	3.6E-03	MKL (megakaryoblastic leukemia)/myocardin-like 1 [Source:MGI Symbol;Acc:MGI:2384495]
Ttc7b	0.59	3.7E-02	tetratricopeptide repeat domain 7B [Source:MGI Symbol;Acc:MGI:2144724]
Cuedc1	0.58	2.5E-02	CUE domain containing 1 [Source:MGI Symbol;Acc:MGI:2144281]
P2rx4	0.58	1.9E-02	purinergic receptor P2X, ligand-gated ion channel 4 [Source:MGI Symbol;Acc:MGI:1338859]
Spns2	0.58	4.8E-02	spinster homolog 2 [Source:MGI Symbol;Acc:MGI:2384936]
L3mbtl3	0.58	2.3E-02	l(3)mbt-like 3 (Drosophila) [Source:MGI Symbol;Acc:MGI:2143628]
Bnip2	0.58	2.6E-03	BCL2/adenovirus E1B interacting protein 2 [Source:MGI Symbol;Acc:MGI:109327]
Bcl9l	0.58	2.2E-02	B cell CLL/lymphoma 9-like [Source:MGI Symbol;Acc:MGI:1933114]
Zfp275	0.57	3.8E-02	zinc finger protein 275 [Source:MGI Symbol;Acc:MGI:1350985]
Man2b2	0.57	1.4E-02	mannosidase 2, alpha B2 [Source:MGI Symbol;Acc:MGI:1195262]
Slc12a4	0.57	4.8E-02	solute carrier family 12, member 4 [Source:MGI Symbol;Acc:MGI:1309465]
BC029722	0.57	4.8E-02	cDNA sequence BC029722 [Source:MGI Symbol;Acc:MGI:3584273]
Crlf3	0.57	9.2E-03	cytokine receptor-like factor 3 [Source:MGI Symbol;Acc:MGI:1860086]
Rapgef1	0.57	4.5E-03	Rap guanine nucleotide exchange factor (GEF) 1 [Source:MGI Symbol;Acc:MGI:104580]
Fth1	0.57	4.9E-02	ferritin heavy chain 1 [Source:MGI Symbol;Acc:MGI:95588]
Sh3pxd2a	0.57	5.2E-03	SH3 and PX domains 2A [Source:MGI Symbol;Acc:MGI:1298393]
Egln2	0.57	3.1E-03	egl-9 family hypoxia-inducible factor 2 [Source:MGI Symbol;Acc:MGI:1932287]
Zfp362	0.56	3.6E-02	zinc finger protein 362 [Source:MGI Symbol;Acc:MGI:2652839]
Eid1	0.56	1.9E-02	EP300 interacting inhibitor of differentiation 1 [Source:MGI Symbol;Acc:MGI:1889651]
Sh3kbp1	0.56	1.4E-02	SH3-domain kinase binding protein 1 [Source:MGI Symbol;Acc:MGI:1889583]
Tmem176b	0.56	2.7E-03	transmembrane protein 176B [Source:MGI Symbol;Acc:MGI:1916348]
Txndc16	0.56	4.4E-02	thioredoxin domain containing 16 [Source:MGI Symbol;Acc:MGI:1917811]
Phf20	0.56	1.7E-02	PHD finger protein 20 [Source:MGI Symbol;Acc:MGI:2444148]
Pcdhgc4	0.56	2.1E-02	protocadherin gamma subfamily C, 4 [Source:MGI Symbol;Acc:MGI:1935203]
Man2a2	0.56	3.2E-02	mannosidase 2, alpha 2 [Source:MGI Symbol;Acc:MGI:2150656]
Ptprc	0.56	2.8E-02	protein tyrosine phosphatase, receptor type, C [Source:MGI Symbol;Acc:MGI:97810]
Wdfy4	0.56	2.9E-02	WD repeat and FYVE domain containing 4 [Source:MGI Symbol;Acc:MGI:3584510]
Tk2	0.56	2.4E-02	thymidine kinase 2, mitochondrial [Source:MGI Symbol;Acc:MGI:1913266]
Chst15	0.56	3.8E-02	carbohydrate (N-acetylgalactosamine 4-sulfate 6-O) sulfotransferase 15 [Source:MGI Symbol;Acc:MGI:1924840]
Csf2ra	0.56	3.1E-03	colony stimulating factor 2 receptor, alpha, low-affinity (granulocyte-macrophage) [Source:MGI Symbol;Acc:MGI:1339754]
Pdgfrb	0.55	2.4E-02	platelet derived growth factor receptor, beta polypeptide [Source:MGI Symbol;Acc:MGI:97531]
Akap8l	0.55	3.7E-02	A kinase (PRKA) anchor protein 8-like [Source:MGI Symbol;Acc:MGI:1860606]
Abcc5	0.54	3.7E-02	ATP-binding cassette, sub-family C (CFTR/MRP), member 5 [Source:MGI Symbol;Acc:MGI:1351644]
Lamb1	0.54	5.4E-03	laminin B1 [Source:MGI Symbol;Acc:MGI:96743]
Arhgap29	0.54	3.9E-02	Rho GTPase activating protein 29 [Source:MGI Symbol;Acc:MGI:2443818]
Tln2	0.54	4.6E-02	talin 2 [Source:MGI Symbol;Acc:MGI:1917799]
Bicd2	0.53	3.1E-02	bicaudal D homolog 2 (Drosophila) [Source:MGI Symbol;Acc:MGI:1924145]
Lrp1	0.53	1.6E-02	low density lipoprotein receptor-related protein 1 [Source:MGI Symbol;Acc:MGI:96828]
Tmem104	0.53	2.4E-02	transmembrane protein 104 [Source:MGI Symbol;Acc:MGI:2444222]
Pitpnm1	0.53	2.3E-02	phosphatidylinositol transfer protein, membrane-associated 1 [Source:MGI Symbol;Acc:MGI:1197524]

Tppp	0.53	2.9E-02	tubulin polymerization promoting protein [Source:MGI Symbol;Acc:MGI:1920198]
Mknk1	0.53	2.5E-02	MAP kinase-interacting serine/threonine kinase 1 [Source:MGI Symbol;Acc:MGI:894316]
Bmp4	0.53	2.0E-02	bone morphogenetic protein 4 [Source:MGI Symbol;Acc:MGI:88180]
Tlr2	0.52	4.8E-02	toll-like receptor 2 [Source:MGI Symbol;Acc:MGI:1346060]
Hook3	0.52	3.5E-02	hook homolog 3 (Drosophila) [Source:MGI Symbol;Acc:MGI:2443554]
Sipa1l2	0.52	4.1E-02	signal-induced proliferation-associated 1 like 2 [Source:MGI Symbol;Acc:MGI:2676970]
Bin1	0.52	4.2E-02	bridging integrator 1 [Source:MGI Symbol;Acc:MGI:108092]
Acp2	0.52	5.6E-03	acid phosphatase 2, lysosomal [Source:MGI Symbol;Acc:MGI:87882]
Lrpap1	0.52	1.5E-02	low density lipoprotein receptor-related protein associated protein 1 [Source:MGI Symbol;Acc:MGI:96829]
Trim35	0.52	4.3E-02	tripartite motif-containing 35 [Source:MGI Symbol;Acc:MGI:1914104]
Lama5	0.52	2.0E-02	laminin, alpha 5 [Source:MGI Symbol;Acc:MGI:105382]
Slco2b1	0.52	4.0E-02	solute carrier organic anion transporter family, member 2b1 [Source:MGI Symbol;Acc:MGI:1351872]
Dock9	0.52	4.4E-02	dedicator of cytokinesis 9 [Source:MGI Symbol;Acc:MGI:106321]
Maged1	0.51	3.5E-02	melanoma antigen, family D, 1 [Source:MGI Symbol;Acc:MGI:1930187]
Tyk2	0.51	4.7E-03	tyrosine kinase 2 [Source:MGI Symbol;Acc:MGI:1929470]
Nrbp2	0.51	4.5E-02	nuclear receptor binding protein 2 [Source:MGI Symbol;Acc:MGI:2385017]
Cnn2	0.51	1.4E-02	calponin 2 [Source:MGI Symbol;Acc:MGI:105093]
Ubald1	0.51	1.4E-02	UBA-like domain containing 1 [Source:MGI Symbol;Acc:MGI:1916255]
Daam2	0.51	2.9E-02	dishevelled associated activator of morphogenesis 2 [Source:MGI Symbol;Acc:MGI:1923691]
Inpp4a	0.51	2.0E-02	inositol polyphosphate-4-phosphatase, type I [Source:MGI Symbol;Acc:MGI:1931123]
Gna12	0.51	7.3E-03	guanine nucleotide binding protein, alpha 12 [Source:MGI Symbol;Acc:MGI:95767]
Orai3	0.51	4.6E-02	ORAI calcium release-activated calcium modulator 3 [Source:MGI Symbol;Acc:MGI:3039586]
Akr1b3	0.51	8.9E-03	aldo-keto reductase family 1, member B3 (aldose reductase) [Source:MGI Symbol;Acc:MGI:1353494]
Macf1	0.51	9.2E-03	microtubule-actin crosslinking factor 1 [Source:MGI Symbol;Acc:MGI:108559]
Filip1l	0.50	2.1E-02	filamin A interacting protein 1-like [Source:MGI Symbol;Acc:MGI:1925999]
Tpcn1	0.50	1.4E-02	two pore channel 1 [Source:MGI Symbol;Acc:MGI:2182472]
Setd1b	0.50	9.8E-03	SET domain containing 1B [Source:MGI Symbol;Acc:MGI:2652820]
Lpar1	0.50	9.2E-03	lysophosphatidic acid receptor 1 [Source:MGI Symbol;Acc:MGI:108429]
Myo9a	0.49	4.3E-02	myosin IXa [Source:MGI Symbol;Acc:MGI:107735]
Gorasp1	0.49	2.9E-02	golgi reassembly stacking protein 1 [Source:MGI Symbol;Acc:MGI:1921748]
Abhd12	0.49	1.4E-02	abhydrolase domain containing 12 [Source:MGI Symbol;Acc:MGI:1923442]
Rnf130	0.49	7.5E-03	ring finger protein 130 [Source:MGI Symbol;Acc:MGI:1891717]
Map7d1	0.49	4.7E-02	MAP7 domain containing 1 [Source:MGI Symbol;Acc:MGI:2384297]
Fnbp1	0.49	4.9E-02	formin binding protein 1 [Source:MGI Symbol;Acc:MGI:109606]
Vwf	0.49	4.8E-02	Von Willebrand factor homolog [Source:MGI Symbol;Acc:MGI:98941]
Heg1	0.49	3.2E-02	HEG homolog 1 (zebrafish) [Source:MGI Symbol;Acc:MGI:1924696]
Ptch1	0.48	3.7E-02	patched homolog 1 [Source:MGI Symbol;Acc:MGI:105373]
Slc8b1	0.48	2.1E-02	solute carrier family 8 (sodium/lithium/calcium exchanger), member B1 [Source:MGI Symbol;Acc:MGI:2180781]
Anxa5	0.48	4.6E-02	annexin A5 [Source:MGI Symbol;Acc:MGI:106008]
Camk1d	0.48	3.3E-02	calcium/calmodulin-dependent protein kinase ID [Source:MGI Symbol;Acc:MGI:2442190]
Dnajb4	0.48	3.5E-02	DnaJ (Hsp40) homolog, subfamily B, member 4 [Source:MGI Symbol;Acc:MGI:1914285]
Nfix	0.48	2.9E-02	nuclear factor I/X [Source:MGI Symbol;Acc:MGI:97311]
Atf7	0.48	4.5E-02	activating transcription factor 7 [Source:MGI Symbol;Acc:MGI:2443472]
Fnip2	0.47	4.8E-02	folliculin interacting protein 2 [Source:MGI Symbol;Acc:MGI:2683054]
Ppp1r21	0.47	2.4E-02	protein phosphatase 1, regulatory subunit 21 [Source:MGI Symbol;Acc:MGI:1921075]
Nfia	0.47	2.1E-02	nuclear factor I/A [Source:MGI Symbol;Acc:MGI:108056]
Fam65a	0.47	3.2E-02	family with sequence similarity 65, member A [Source:MGI Symbol;Acc:MGI:1922937]
Prr12	0.47	2.9E-02	proline rich 12 [Source:MGI Symbol;Acc:MGI:2679002]
Ctso	0.47	4.1E-02	cathepsin O [Source:MGI Symbol;Acc:MGI:2139628]
Creg1	0.46	2.2E-02	cellular repressor of E1A-stimulated genes 1 [Source:MGI Symbol;Acc:MGI:1344382]
Thra	0.46	2.3E-02	thyroid hormone receptor alpha [Source:MGI Symbol;Acc:MGI:98742]

Bcat2	0.46	4.4E-02	branched chain aminotransferase 2, mitochondrial [Source:MGI Symbol;Acc:MGI:1276534]
Zyx	0.46	1.5E-02	zyxin [Source:MGI Symbol;Acc:MGI:103072]
Extl3	0.45	1.8E-02	exostoses (multiple)-like 3 [Source:MGI Symbol;Acc:MGI:1860765]
Stxbp3a	0.45	1.8E-02	syntaxin binding protein 3A [Source:MGI Symbol;Acc:MGI:107362]
Ocrl	0.45	4.4E-02	oculocerebrorenal syndrome of Lowe [Source:MGI Symbol;Acc:MGI:109589]
Ppfibp2	0.45	2.7E-02	PTPRF interacting protein, binding protein 2 (liprin beta 2) [Source:MGI Symbol;Acc:MGI:894649]
Smarca2	0.45	2.6E-02	SWI/SNF related, matrix associated, actin dependent regulator of chromatin, subfamily a, member 2 [Source:MGI Symbol;Acc:MGI:99603]
Mtss1	0.45	1.7E-02	metastasis suppressor 1 [Source:MGI Symbol;Acc:MGI:2384818]
Osbpl8	0.45	4.6E-02	oxysterol binding protein-like 8 [Source:MGI Symbol;Acc:MGI:2443807]
Fam49b	0.44	9.8E-03	family with sequence similarity 49, member B [Source:MGI Symbol;Acc:MGI:1923520]
Pdrg1	0.44	4.8E-02	p53 and DNA damage regulated 1 [Source:MGI Symbol;Acc:MGI:1915809]
Fnbp1l	0.44	2.2E-02	formin binding protein 1-like [Source:MGI Symbol;Acc:MGI:1925642]
Nceh1	0.44	4.8E-02	neutral cholesterol ester hydrolase 1 [Source:MGI Symbol;Acc:MGI:2443191]
Rhog	0.43	1.5E-02	ras homolog gene family, member G [Source:MGI Symbol;Acc:MGI:1928370]
Slc25a36	0.43	4.9E-02	solute carrier family 25, member 36 [Source:MGI Symbol;Acc:MGI:1924909]
Trim8	0.43	4.3E-02	tripartite motif-containing 8 [Source:MGI Symbol;Acc:MGI:1933302]
Tcirg1	0.42	2.3E-02	T cell, immune regulator 1, ATPase, H+ transporting, lysosomal V0 protein A3 [Source:MGI Symbol;Acc:MGI:1350931]
Itpripl2	0.42	3.0E-02	inositol 1,4,5-triphosphate receptor interacting protein-like 2 [Source:MGI Symbol;Acc:MGI:2442416]
Pip5k1c	0.42	4.5E-02	phosphatidylinositol-4-phosphate 5-kinase, type 1 gamma [Source:MGI Symbol;Acc:MGI:1298224]
Tfe3	0.42	2.4E-02	transcription factor E3 [Source:MGI Symbol;Acc:MGI:98511]
Tcf3	0.41	2.3E-02	transcription factor 3 [Source:MGI Symbol;Acc:MGI:98510]
Megf8	0.41	3.4E-02	multiple EGF-like-domains 8 [Source:MGI Symbol;Acc:MGI:2446294]
Rap1b	0.41	3.4E-02	RAS related protein 1b [Source:MGI Symbol;Acc:MGI:894315]
Dnmt3a	0.41	2.8E-02	DNA methyltransferase 3A [Source:MGI Symbol;Acc:MGI:1261827]
Hexa	0.41	4.6E-02	hexosaminidase A [Source:MGI Symbol;Acc:MGI:96073]
Ppp1r9b	0.41	3.2E-02	protein phosphatase 1, regulatory subunit 9B [Source:MGI Symbol;Acc:MGI:2387581]
Irak2	0.40	3.8E-02	interleukin-1 receptor-associated kinase 2 [Source:MGI Symbol;Acc:MGI:2429603]
Ltn1	0.40	3.8E-02	listerin E3 ubiquitin protein ligase 1 [Source:MGI Symbol;Acc:MGI:1926163]
Tbc1d14	0.40	4.5E-02	TBC1 domain family, member 14 [Source:MGI Symbol;Acc:MGI:1098708]
Tmem123	0.40	3.3E-02	transmembrane protein 123 [Source:MGI Symbol;Acc:MGI:1919179]
Dock8	0.38	5.0E-02	dedicator of cytokinesis 8 [Source:MGI Symbol;Acc:MGI:1921396]
Foxp1	0.38	4.2E-02	forkhead box P1 [Source:MGI Symbol;Acc:MGI:1914004]
Kmt2a	0.37	4.3E-02	lysine (K)-specific methyltransferase 2A [Source:MGI Symbol;Acc:MGI:96995]
Prpf38b	0.36	5.0E-02	PRP38 pre-mRNA processing factor 38 (yeast) domain containing B [Source:MGI Symbol;Acc:MGI:1914171]
Jarid2	-0.36	4.6E-02	jumonji, AT rich interactive domain 2 [Source:MGI Symbol;Acc:MGI:104813]
Ogfr	-0.37	3.5E-02	opioid growth factor receptor [Source:MGI Symbol;Acc:MGI:1919325]
Dnpep	-0.37	4.3E-02	aspartyl aminopeptidase [Source:MGI Symbol;Acc:MGI:1278328]
Arap2	-0.38	4.0E-02	ArfGAP with RhoGAP domain, ankyrin repeat and PH domain 2 [Source:MGI Symbol;Acc:MGI:2684416]
Pof1b	-0.38	4.4E-02	premature ovarian failure 1B [Source:MGI Symbol;Acc:MGI:1916943]
Tmem9b	-0.38	4.8E-02	TMEM9 domain family, member B [Source:MGI Symbol;Acc:MGI:1915254]
Scfd1	-0.38	4.8E-02	Sec1 family domain containing 1 [Source:MGI Symbol;Acc:MGI:1924233]
Pdk1	-0.38	2.9E-02	pyruvate dehydrogenase kinase, isoenzyme 1 [Source:MGI Symbol;Acc:MGI:1926119]
Suz12	-0.38	4.0E-02	suppressor of zeste 12 homolog (Drosophila) [Source:MGI Symbol;Acc:MGI:1261758]
Scpep1	-0.39	4.7E-02	serine carboxypeptidase 1 [Source:MGI Symbol;Acc:MGI:1921867]
Bspry	-0.39	4.8E-02	B-box and SPRY domain containing [Source:MGI Symbol;Acc:MGI:2177191]
Eny2	-0.39	4.9E-02	enhancer of yellow 2 homolog (Drosophila) [Source:MGI Symbol;Acc:MGI:1919286]
Dhx32	-0.39	3.5E-02	DEAH (Asp-Glu-Ala-His) box polypeptide 32 [Source:MGI Symbol;Acc:MGI:2141813]
Nr2f6	-0.39	3.6E-02	nuclear receptor subfamily 2, group F, member 6 [Source:MGI Symbol;Acc:MGI:1352453]
Cyth2	-0.39	4.3E-02	cytohesin 2 [Source:MGI Symbol;Acc:MGI:1334255]
Tmco4	-0.40	5.0E-02	transmembrane and coiled-coil domains 4 [Source:MGI Symbol;Acc:MGI:1924306]
Nhsl1	-0.40	3.5E-02	NHS-like 1 [Source:MGI Symbol;Acc:MGI:106390]

Plekha2	-0.40	4.9E-02	pleckstrin homology domain-containing, family A (phosphoinositide binding specific) member 2 [Source:MGI Symbol;Acc:MGI:1928144]
Sptlc1	-0.40	3.6E-02	serine palmitoyltransferase, long chain base subunit 1 [Source:MGI Symbol;Acc:MGI:1099431]
Aifm1	-0.40	3.6E-02	apoptosis-inducing factor, mitochondrion-associated 1 [Source:MGI Symbol;Acc:MGI:1349419]
Mob3b	-0.41	3.3E-02	MOB kinase activator 3B [Source:MGI Symbol;Acc:MGI:2664539]
Csnk1g3	-0.41	2.9E-02	casein kinase 1, gamma 3 [Source:MGI Symbol;Acc:MGI:1917675]
Dcp2	-0.41	3.2E-02	DCP2 decapping enzyme homolog (S. cerevisiae) [Source:MGI Symbol;Acc:MGI:1917890]
Sgms1	-0.41	4.6E-02	sphingomyelin synthase 1 [Source:MGI Symbol;Acc:MGI:2444110]
Txndc9	-0.41	4.3E-02	thioredoxin domain containing 9 [Source:MGI Symbol;Acc:MGI:2138153]
Mtch2	-0.41	4.4E-02	mitochondrial carrier homolog 2 (C. elegans) [Source:MGI Symbol;Acc:MGI:1929260]
Arhgef5	-0.41	4.0E-02	Rho guanine nucleotide exchange factor (GEF) 5 [Source:MGI Symbol;Acc:MGI:1858952]
Bcar3	-0.41	2.6E-02	breast cancer anti-estrogen resistance 3 [Source:MGI Symbol;Acc:MGI:1352501]
Chchd3	-0.42	3.9E-02	coiled-coil-helix-coiled-coil-helix domain containing 3 [Source:MGI Symbol;Acc:MGI:1913325]
Fam135a	-0.42	2.4E-02	family with sequence similarity 135, member A [Source:MGI Symbol;Acc:MGI:1915437]
Fam83g	-0.42	3.2E-02	family with sequence similarity 83, member G [Source:MGI Symbol;Acc:MGI:1916890]
Camsap3	-0.42	3.1E-02	calmodulin regulated spectrin-associated protein family, member 3 [Source:MGI Symbol;Acc:MGI:1916947]
Fam84b	-0.42	1.5E-02	family with sequence similarity 84, member B [Source:MGI Symbol;Acc:MGI:3026924]
Zc3h15	-0.42	3.7E-02	zinc finger CCCH-type containing 15 [Source:MGI Symbol;Acc:MGI:1919747]
Atg4d	-0.42	3.5E-02	autophagy related 4D, cysteine peptidase [Source:MGI Symbol;Acc:MGI:2444308]
Afg3l2	-0.42	3.9E-02	AFG3-like AAA ATPase 2 [Source:MGI Symbol;Acc:MGI:1916847]
Stk38l	-0.42	1.5E-02	serine/threonine kinase 38 like [Source:MGI Symbol;Acc:MGI:1922250]
Hmgn5	-0.42	3.4E-02	high-mobility group nucleosome binding domain 5 [Source:MGI Symbol;Acc:MGI:1355295]
Cisd1	-0.42	4.8E-02	CDGSH iron sulfur domain 1 [Source:MGI Symbol;Acc:MGI:1261855]
Met	-0.42	4.3E-02	met proto-oncogene [Source:MGI Symbol;Acc:MGI:96969]
Nudt19	-0.42	3.7E-02	nudix (nucleoside diphosphate linked moiety X)-type motif 19 [Source:MGI Symbol;Acc:MGI:94203]
Lpin3	-0.42	4.6E-02	lipin 3 [Source:MGI Symbol;Acc:MGI:1891342]
Pdhb	-0.42	3.8E-02	pyruvate dehydrogenase (lipoamide) beta [Source:MGI Symbol;Acc:MGI:1915513]
Fam160a2	-0.43	3.4E-02	family with sequence similarity 160, member A2 [Source:MGI Symbol;Acc:MGI:1921599]
Chuk	-0.43	2.9E-02	conserved helix-loop-helix ubiquitous kinase [Source:MGI Symbol;Acc:MGI:99484]
Ndufs6	-0.43	3.1E-02	NADH dehydrogenase (ubiquinone) Fe-S protein 6 [Source:MGI Symbol;Acc:MGI:107932]
Dcbld1	-0.43	2.9E-02	discoidin, CUB and LCCL domain containing 1 [Source:MGI Symbol;Acc:MGI:1913936]
Aldh16a1	-0.43	4.2E-02	aldehyde dehydrogenase 16 family, member A1 [Source:MGI Symbol;Acc:MGI:1916998]
Atp11b	-0.43	4.4E-02	ATPase, class VI, type 11B [Source:MGI Symbol;Acc:MGI:1923545]
Eea1	-0.43	4.3E-02	early endosome antigen 1 [Source:MGI Symbol;Acc:MGI:2442192]
Arhgef16	-0.43	3.2E-02	Rho guanine nucleotide exchange factor (GEF) 16 [Source:MGI Symbol;Acc:MGI:2446219]
Bcl10	-0.43	1.6E-02	B cell leukemia/lymphoma 10 [Source:MGI Symbol;Acc:MGI:1337994]
Cblc	-0.43	2.8E-02	Casitas B-lineage lymphoma c [Source:MGI Symbol;Acc:MGI:1931457]
6330416G13Rik	-0.43	4.6E-02	RIKEN cDNA 6330416G13 gene [Source:MGI Symbol;Acc:MGI:1913920]
Slc33a1	-0.43	2.7E-02	solute carrier family 33 (acetyl-CoA transporter), member 1 [Source:MGI Symbol;Acc:MGI:1332247]
Ap3s1	-0.43	2.9E-02	adaptor-related protein complex 3, sigma 1 subunit [Source:MGI Symbol;Acc:MGI:1337062]
Smad7	-0.43	4.9E-02	SMAD family member 7 [Source:MGI Symbol;Acc:MGI:1100518]
Pim1	-0.43	3.1E-02	proviral integration site 1 [Source:MGI Symbol;Acc:MGI:97584]
Rab24	-0.44	2.9E-02	RAB24, member RAS oncogene family [Source:MGI Symbol;Acc:MGI:105065]
Pi4k2b	-0.44	2.5E-02	phosphatidylinositol 4-kinase type 2 beta [Source:MGI Symbol;Acc:MGI:1914323]
Coa3	-0.44	4.2E-02	cytochrome C oxidase assembly factor 3 [Source:MGI Symbol;Acc:MGI:1098757]
Sh3bp5l	-0.44	3.1E-02	SH3 binding domain protein 5 like [Source:MGI Symbol;Acc:MGI:1933124]
Mlx	-0.44	4.1E-02	MAX-like protein X [Source:MGI Symbol;Acc:MGI:108398]

Igsf5	-0.44	2.8E-02	immunoglobulin superfamily, member 5 [Source:MGI Symbol;Acc:MGI:1919308]
2610528J11Rik	-0.44	3.7E-02	RIKEN cDNA 2610528J11 gene [Source:MGI Symbol;Acc:MGI:1913701]
Apaf1	-0.44	1.7E-02	apoptotic peptidase activating factor 1 [Source:MGI Symbol;Acc:MGI:1306796]
Myo10	-0.44	2.4E-02	myosin X [Source:MGI Symbol;Acc:MGI:107716]
Rala	-0.44	3.6E-02	v-ral simian leukemia viral oncogene homolog A (ras related) [Source:MGI Symbol;Acc:MGI:1927243]
Ugcg	-0.44	3.5E-02	UDP-glucose ceramide glucosyltransferase [Source:MGI Symbol;Acc:MGI:1332243]
Slc30a4	-0.44	4.3E-02	solute carrier family 30 (zinc transporter), member 4 [Source:MGI Symbol;Acc:MGI:1345282]
Cmas	-0.44	2.9E-02	cytidine monophospho-N-acetylneuraminic acid synthetase [Source:MGI Symbol;Acc:MGI:1337124]
Ankrd50	-0.44	4.0E-02	ankyrin repeat domain 50 [Source:MGI Symbol;Acc:MGI:2139777]
Ifih1	-0.45	3.7E-02	interferon induced with helicase C domain 1 [Source:MGI Symbol;Acc:MGI:1918836]
Casz1	-0.45	1.9E-02	castor zinc finger 1 [Source:MGI Symbol;Acc:MGI:1196251]
B4galt3	-0.45	4.2E-02	UDP-Gal:betaGlcNAc beta 1,4-galactosyltransferase, polypeptide 3 [Source:MGI Symbol;Acc:MGI:1928767]
Parp12	-0.45	3.7E-02	poly (ADP-ribose) polymerase family, member 12 [Source:MGI Symbol;Acc:MGI:2143990]
Atpif1	-0.45	2.9E-02	ATPase inhibitory factor 1 [Source:MGI Symbol;Acc:MGI:1196457]
Tor1aip2	-0.45	3.7E-02	torsin A interacting protein 2 [Source:MGI Symbol;Acc:MGI:3582695]
Psma1	-0.45	4.8E-02	proteasome (prosome, macropain) subunit, alpha type 1 [Source:MGI Symbol;Acc:MGI:1347005]
Reep4	-0.45	1.6E-02	receptor accessory protein 4 [Source:MGI Symbol;Acc:MGI:1919799]
Casp1	-0.45	3.3E-02	caspase 1 [Source:MGI Symbol;Acc:MGI:96544]
Plscr1	-0.45	4.2E-02	phospholipid scramblase 1 [Source:MGI Symbol;Acc:MGI:893575]
Dock5	-0.45	4.0E-02	dedicator of cytokinesis 5 [Source:MGI Symbol;Acc:MGI:2652871]
Cox6c	-0.45	4.9E-02	cytochrome c oxidase subunit VIc [Source:MGI Symbol;Acc:MGI:104614]
Fam83h	-0.46	4.4E-02	family with sequence similarity 83, member H [Source:MGI Symbol;Acc:MGI:2145900]
Atp5j	-0.46	3.6E-02	ATP synthase, H+ transporting, mitochondrial F0 complex, subunit F [Source:MGI Symbol;Acc:MGI:107777]
Tmem238	-0.46	3.6E-02	transmembrane protein 238 [Source:MGI Symbol;Acc:MGI:1922935]
Psma4	-0.46	4.3E-02	proteasome (prosome, macropain) subunit, alpha type 4 [Source:MGI Symbol;Acc:MGI:1347060]
Map2k3	-0.46	4.0E-02	mitogen-activated protein kinase kinase 3 [Source:MGI Symbol;Acc:MGI:1346868]
Sdhc	-0.46	4.4E-02	succinate dehydrogenase complex, subunit C, integral membrane protein [Source:MGI Symbol;Acc:MGI:1913302]
Ndufab1	-0.46	4.8E-02	NADH dehydrogenase (ubiquinone) 1, alpha/beta subcomplex, 1 [Source:MGI Symbol;Acc:MGI:1917566]
Sh3yl1	-0.46	2.9E-02	Sh3 domain YSC-like 1 [Source:MGI Symbol;Acc:MGI:1346118]
Cast	-0.46	3.8E-02	calpastatin [Source:MGI Symbol;Acc:MGI:1098236]
Krtcap3	-0.46	1.7E-02	keratinocyte associated protein 3 [Source:MGI Symbol;Acc:MGI:1917065]
Sec23b	-0.46	2.2E-02	SEC23B (S. cerevisiae) [Source:MGI Symbol;Acc:MGI:1350925]
Optn	-0.46	3.6E-02	optineurin [Source:MGI Symbol;Acc:MGI:1918898]
Uqcr10	-0.46	4.5E-02	ubiquinol-cytochrome c reductase, complex III subunit X [Source:MGI Symbol;Acc:MGI:1913402]
F11r	-0.47	4.8E-02	F11 receptor [Source:MGI Symbol;Acc:MGI:1321398]
Letm1	-0.47	4.0E-02	leucine zipper-EF-hand containing transmembrane protein 1 [Source:MGI Symbol;Acc:MGI:1932557]
Stk39	-0.47	2.1E-02	serine/threonine kinase 39 [Source:MGI Symbol;Acc:MGI:1858416]
Agpat4	-0.47	4.6E-02	1-acylglycerol-3-phosphate O-acyltransferase 4 (lysophosphatidic acid acyltransferase, delta) [Source:MGI Symbol;Acc:MGI:1915512]
Ndufc1	-0.47	4.7E-02	NADH dehydrogenase (ubiquinone) 1, subcomplex unknown, 1 [Source:MGI Symbol;Acc:MGI:1913627]
Snx25	-0.47	1.7E-02	sorting nexin 25 [Source:MGI Symbol;Acc:MGI:2142610]
Chn2	-0.47	2.8E-02	chimerin 2 [Source:MGI Symbol;Acc:MGI:1917243]
Hook1	-0.47	3.2E-02	hook homolog 1 (Drosophila) [Source:MGI Symbol;Acc:MGI:1925213]
Dok4	-0.47	4.5E-02	docking protein 4 [Source:MGI Symbol;Acc:MGI:2148865]
Rps27l	-0.47	3.3E-02	ribosomal protein S27-like [Source:MGI Symbol;Acc:MGI:1915191]
Btnl4	-0.47	2.2E-02	butyrophilin-like 4 [Source:MGI Symbol;Acc:MGI:1932036]
Cyp4f40	-0.47	4.9E-02	cytochrome P450, family 4, subfamily f, polypeptide 40 [Source:MGI Symbol;Acc:MGI:3645508]
Gmcl1	-0.47	1.3E-02	germ cell-less homolog 1 (Drosophila) [Source:MGI Symbol;Acc:MGI:1345156]

Rnpep	-0.47	4.1E-02	arginyl aminopeptidase (aminopeptidase B) [Source:MGI Symbol;Acc:MGI:2384902]
Cox7b	-0.47	4.4E-02	cytochrome c oxidase subunit VIIb [Source:MGI Symbol;Acc:MGI:1913392]
Pklr	-0.47	3.6E-02	pyruvate kinase liver and red blood cell [Source:MGI Symbol;Acc:MGI:97604]
Srebf2	-0.47	2.2E-02	sterol regulatory element binding factor 2 [Source:MGI Symbol;Acc:MGI:107585]
Nab1	-0.47	1.4E-02	Ngfi-A binding protein 1 [Source:MGI Symbol;Acc:MGI:107564]
Cox7a2	-0.47	2.4E-02	cytochrome c oxidase subunit VIIa 2 [Source:MGI Symbol;Acc:MGI:1316715]
Txndc17	-0.47	3.0E-02	thioredoxin domain containing 17 [Source:MGI Symbol;Acc:MGI:1289248]
Ifrd1	-0.47	5.0E-02	interferon-related developmental regulator 1 [Source:MGI Symbol;Acc:MGI:1316717]
Igsf23	-0.47	4.4E-02	immunoglobulin superfamily, member 23 [Source:MGI Symbol;Acc:MGI:1917330]
Myo5b	-0.48	3.0E-02	myosin VB [Source:MGI Symbol;Acc:MGI:106598]
Epb4.1l4b	-0.48	3.5E-02	erythrocyte protein band 4.1-like 4b [Source:MGI Symbol;Acc:MGI:1859149]
Tjp2	-0.48	2.8E-02	tight junction protein 2 [Source:MGI Symbol;Acc:MGI:1341872]
Ssx2ip	-0.48	1.6E-02	synovial sarcoma, X breakpoint 2 interacting protein [Source:MGI Symbol;Acc:MGI:2139150]
Dera	-0.48	3.2E-02	2-deoxyribose-5-phosphate aldolase homolog (C. elegans) [Source:MGI Symbol;Acc:MGI:1913762]
Mcu	-0.48	3.4E-02	mitochondrial calcium uniporter [Source:MGI Symbol;Acc:MGI:3026965]
Plcl2	-0.48	5.0E-03	phospholipase C-like 2 [Source:MGI Symbol;Acc:MGI:1352756]
Ywhah	-0.48	4.0E-02	tyrosine 3-monooxygenase/tryptophan 5-monooxygenase activation protein, eta polypeptide [Source:MGI Symbol;Acc:MGI:109194]
Map2k4	-0.48	7.7E-03	mitogen-activated protein kinase kinase 4 [Source:MGI Symbol;Acc:MGI:1346869]
Fbp2	-0.48	2.3E-02	fructose bisphosphatase 2 [Source:MGI Symbol;Acc:MGI:95491]
Mrpl51	-0.48	2.6E-02	mitochondrial ribosomal protein L51 [Source:MGI Symbol;Acc:MGI:1913743]
Gpc4	-0.48	1.6E-02	glypican 4 [Source:MGI Symbol;Acc:MGI:104902]
Mad2l1bp	-0.49	4.4E-02	MAD2L1 binding protein [Source:MGI Symbol;Acc:MGI:1913841]
Unc5b	-0.49	3.4E-02	unc-5 homolog B (C. elegans) [Source:MGI Symbol;Acc:MGI:894703]
#N/A	-0.49	4.0E-02	#N/A
Dnajc22	-0.49	1.0E-02	DnaJ (Hsp40) homolog, subfamily C, member 22 [Source:MGI Symbol;Acc:MGI:1920028]
Tmem41b	-0.49	2.2E-02	transmembrane protein 41B [Source:MGI Symbol;Acc:MGI:1289225]
Lig1	-0.49	4.7E-02	ligase I, DNA, ATP-dependent [Source:MGI Symbol;Acc:MGI:101789]
Rdh11	-0.49	1.7E-02	retinol dehydrogenase 11 [Source:MGI Symbol;Acc:MGI:102581]
Ebp	-0.49	6.3E-03	phenylalkylamine Ca2+ antagonist (emopamil) binding protein [Source:MGI Symbol;Acc:MGI:107822]
Fam129b	-0.49	1.4E-02	family with sequence similarity 129, member B [Source:MGI Symbol;Acc:MGI:2442910]
Prelid1	-0.49	3.9E-02	PRELI domain containing 1 [Source:MGI Symbol;Acc:MGI:1913744]
Sema4b	-0.49	9.2E-03	sema domain, immunoglobulin domain (Ig), transmembrane domain (TM) and short cytoplasmic domain, (semaphorin) 4B [Source:MGI Symbol;Acc:MGI:107559]
Ldlr	-0.49	4.7E-02	low density lipoprotein receptor [Source:MGI Symbol;Acc:MGI:96765]
Cox7c	-0.49	2.9E-02	cytochrome c oxidase subunit VIIc [Source:MGI Symbol;Acc:MGI:103226]
Mesdc1	-0.49	7.2E-03	mesoderm development candidate 1 [Source:MGI Symbol;Acc:MGI:1891420]
Sema6d	-0.49	1.5E-02	sema domain, transmembrane domain (TM), and cytoplasmic domain, (semaphorin) 6D [Source:MGI Symbol;Acc:MGI:2387661]
Tprn	-0.49	3.8E-02	taperin [Source:MGI Symbol;Acc:MGI:2139535]
Slc25a22	-0.49	4.5E-02	solute carrier family 25 (mitochondrial carrier, glutamate), member 22 [Source:MGI Symbol;Acc:MGI:1915517]
Pip5k1b	-0.50	8.1E-03	phosphatidylinositol-4-phosphate 5-kinase, type 1 beta [Source:MGI Symbol;Acc:MGI:107930]
Irf6	-0.50	1.1E-02	interferon regulatory factor 6 [Source:MGI Symbol;Acc:MGI:1859211]
Cox5a	-0.50	3.8E-02	cytochrome c oxidase subunit Va [Source:MGI Symbol;Acc:MGI:88474]
Ap1m2	-0.50	6.1E-03	adaptor protein complex AP-1, mu 2 subunit [Source:MGI Symbol;Acc:MGI:1336974]
Styk1	-0.50	4.9E-03	serine/threonine/tyrosine kinase 1 [Source:MGI Symbol;Acc:MGI:2141396]
Cox5b	-0.50	3.5E-02	cytochrome c oxidase subunit Vb [Source:MGI Symbol;Acc:MGI:88475]
Smox	-0.50	3.8E-02	spermine oxidase [Source:MGI Symbol;Acc:MGI:2445356]
9130409J20Rik	-0.50	9.3E-03	RIKEN cDNA 9130409J20 gene [Source:MGI Symbol;Acc:MGI:1918826]
Nostrin	-0.50	4.2E-02	nitric oxide synthase trafficker [Source:MGI Symbol;Acc:MGI:3606242]
Atp5g1	-0.50	4.0E-02	ATP synthase, H+ transporting, mitochondrial F0 complex, subunit C1 (subunit 9) [Source:MGI Symbol;Acc:MGI:107653]
Mtx2	-0.50	1.7E-02	metaxin 2 [Source:MGI Symbol;Acc:MGI:1859652]
Lpcat3	-0.50	1.2E-02	lysophosphatidylcholine acyltransferase 3 [Source:MGI Symbol;Acc:MGI:1315211]
Itga3	-0.50	2.8E-02	integrin alpha 3 [Source:MGI Symbol;Acc:MGI:96602]

Plgrkt	-0.50	1.1E-02	plasminogen receptor, C-terminal lysine transmembrane protein [Source:MGI Symbol;Acc:MGI:1915009]
Ttc39b	-0.51	1.7E-02	tetratricopeptide repeat domain 39B [Source:MGI Symbol;Acc:MGI:1917113]
Rasef	-0.51	4.0E-03	RAS and EF hand domain containing [Source:MGI Symbol;Acc:MGI:2448565]
Lrrc16a	-0.51	4.3E-03	leucine rich repeat containing 16A [Source:MGI Symbol;Acc:MGI:1915982]
Ero1l	-0.51	1.3E-02	ERO1-like (S. cerevisiae) [Source:MGI Symbol;Acc:MGI:1354385]
Elovl6	-0.51	3.5E-02	ELOVL family member 6, elongation of long chain fatty acids (yeast) [Source:MGI Symbol;Acc:MGI:2156528]
Ept1	-0.51	2.2E-02	ethanolaminephosphotransferase 1 (CDP-ethanolamine-specific) [Source:MGI Symbol;Acc:MGI:107898]
Sertad1	-0.51	4.7E-02	SERTA domain containing 1 [Source:MGI Symbol;Acc:MGI:1913438]
Pnp	-0.51	3.3E-02	purine-nucleoside phosphorylase [Source:MGI Symbol;Acc:MGI:97365]
Prpsap1	-0.51	3.3E-02	phosphoribosyl pyrophosphate synthetase-associated protein 1 [Source:MGI Symbol;Acc:MGI:1915013]
Dtx3l	-0.51	3.9E-02	deltex 3-like (Drosophila) [Source:MGI Symbol;Acc:MGI:2656973]
Tmprss2	-0.51	1.9E-02	transmembrane protease, serine 2 [Source:MGI Symbol;Acc:MGI:1354381]
Fas	-0.52	3.5E-02	Fas (TNF receptor superfamily member 6) [Source:MGI Symbol;Acc:MGI:95484]
Tuft1	-0.52	8.9E-03	tuftelin 1 [Source:MGI Symbol;Acc:MGI:109572]
Taldo1	-0.52	2.6E-02	transaldolase 1 [Source:MGI Symbol;Acc:MGI:1274789]
Dhfr	-0.52	5.0E-02	dihydrofolate reductase [Source:MGI Symbol;Acc:MGI:94890]
Gipc2	-0.52	3.6E-02	GIPC PDZ domain containing family, member 2 [Source:MGI Symbol;Acc:MGI:1889209]
Helz2	-0.52	2.4E-02	helicase with zinc finger 2, transcriptional coactivator [Source:MGI Symbol;Acc:MGI:2385169]
Abcb10	-0.52	7.6E-03	ATP-binding cassette, sub-family B (MDR/TAP), member 10 [Source:MGI Symbol;Acc:MGI:1860508]
Cks2	-0.52	4.1E-02	CDC28 protein kinase regulatory subunit 2 [Source:MGI Symbol;Acc:MGI:1913447]
Edf1	-0.52	2.9E-02	endothelial differentiation-related factor 1 [Source:MGI Symbol;Acc:MGI:1891227]
Coq7	-0.52	4.0E-02	demethyl-Q 7 [Source:MGI Symbol;Acc:MGI:107207]
Elf3	-0.52	2.3E-02	E74-like factor 3 [Source:MGI Symbol;Acc:MGI:1101781]
Eif2ak2	-0.52	7.5E-03	eukaryotic translation initiation factor 2-alpha kinase 2 [Source:MGI Symbol;Acc:MGI:1353449]
Usmg5	-0.52	2.6E-02	upregulated during skeletal muscle growth 5 [Source:MGI Symbol;Acc:MGI:1891435]
#N/A	-0.52	6.3E-03	#N/A
Sowahb	-0.52	2.3E-02	sosondowah ankyrin repeat domain family member B [Source:MGI Symbol;Acc:MGI:1925338]
Klf6	-0.52	2.1E-02	Kruppel-like factor 6 [Source:MGI Symbol;Acc:MGI:1346318]
Akr1c12	-0.52	2.5E-02	aldo-keto reductase family 1, member C12 [Source:MGI Symbol;Acc:MGI:1351661]
Mal2	-0.52	1.5E-02	mal, T cell differentiation protein 2 [Source:MGI Symbol;Acc:MGI:2146021]
Hmgb3	-0.52	4.1E-02	high mobility group box 3 [Source:MGI Symbol;Acc:MGI:1098219]
4930539E08Rik	-0.53	3.4E-02	RIKEN cDNA 4930539E08 gene [Source:MGI Symbol;Acc:MGI:1925441]
Cnksr1	-0.53	6.1E-03	connector enhancer of kinase suppressor of Ras 1 [Source:MGI Symbol;Acc:MGI:2670958]
Gpd2	-0.53	2.0E-02	glycerol phosphate dehydrogenase 2, mitochondrial [Source:MGI Symbol;Acc:MGI:99778]
Tpi1	-0.53	4.4E-02	triosephosphate isomerase 1 [Source:MGI Symbol;Acc:MGI:98797]
Dhcr7	-0.53	2.8E-03	7-dehydrocholesterol reductase [Source:MGI Symbol;Acc:MGI:1298378]
Stap2	-0.53	1.2E-02	signal transducing adaptor family member 2 [Source:MGI Symbol;Acc:MGI:2147039]
Gca	-0.53	3.4E-02	grancalcin [Source:MGI Symbol;Acc:MGI:1918521]
Cdh1	-0.53	3.7E-02	cadherin 1 [Source:MGI Symbol;Acc:MGI:88354]
Eif2s2	-0.53	4.6E-02	eukaryotic translation initiation factor 2, subunit 2 (beta) [Source:MGI Symbol;Acc:MGI:1914454]
Nampt	-0.53	1.1E-02	nicotinamide phosphoribosyltransferase [Source:MGI Symbol;Acc:MGI:1929865]
Krt7	-0.53	3.3E-02	keratin 7 [Source:MGI Symbol;Acc:MGI:96704]
Atp5k	-0.53	2.6E-02	ATP synthase, H+ transporting, mitochondrial F1F0 complex, subunit E [Source:MGI Symbol;Acc:MGI:106636]
Ccdc25	-0.53	1.2E-02	coiled-coil domain containing 25 [Source:MGI Symbol;Acc:MGI:1914429]
Pdk3	-0.53	3.4E-02	pyruvate dehydrogenase kinase, isoenzyme 3 [Source:MGI Symbol;Acc:MGI:2384308]
Dsc2	-0.54	3.1E-02	desmocollin 2 [Source:MGI Symbol;Acc:MGI:103221]
Itpk1	-0.54	3.6E-02	inositol 1,3,4-triphosphate 5/6 kinase [Source:MGI Symbol;Acc:MGI:2446159]

Uqcrq	-0.54	3.5E-02	ubiquinol-cytochrome c reductase, complex III subunit VII [Source:MGI Symbol;Acc:MGI:107807]
Eif4e3	-0.54	1.2E-02	eukaryotic translation initiation factor 4E member 3 [Source:MGI Symbol;Acc:MGI:1914142]
Ly75	-0.54	8.7E-03	lymphocyte antigen 75 [Source:MGI Symbol;Acc:MGI:106662]
Pdlim1	-0.54	1.4E-02	PDZ and LIM domain 1 (elfin) [Source:MGI Symbol;Acc:MGI:1860611]
Tapbpl	-0.54	9.8E-03	TAP binding protein-like [Source:MGI Symbol;Acc:MGI:2384853]
Rmdn3	-0.54	1.5E-02	regulator of microtubule dynamics 3 [Source:MGI Symbol;Acc:MGI:1915059]
Rpia	-0.54	2.5E-02	ribose 5-phosphate isomerase A [Source:MGI Symbol;Acc:MGI:103254]
Akr1c13	-0.54	1.8E-02	aldo-keto reductase family 1, member C13 [Source:MGI Symbol;Acc:MGI:1351662]
Abhd17c	-0.54	1.7E-02	abhydrolase domain containing 17C [Source:MGI Symbol;Acc:MGI:1917428]
Ceacam20	-0.54	2.2E-02	carcinoembryonic antigen-related cell adhesion molecule 20 [Source:MGI Symbol;Acc:MGI:1918851]
Got1	-0.54	2.9E-02	glutamic-oxaloacetic transaminase 1, soluble [Source:MGI Symbol;Acc:MGI:95791]
Sec61b	-0.54	2.3E-02	Sec61 beta subunit [Source:MGI Symbol;Acc:MGI:1913462]
Pkp2	-0.54	2.0E-03	plakophilin 2 [Source:MGI Symbol;Acc:MGI:1914701]
Tmem79	-0.54	4.0E-02	transmembrane protein 79 [Source:MGI Symbol;Acc:MGI:1919163]
Mtf1	-0.55	5.8E-03	metal response element binding transcription factor 1 [Source:MGI Symbol;Acc:MGI:101786]
Ccrn4l	-0.55	1.4E-02	CCR4 carbon catabolite repression 4-like (S. cerevisiae) [Source:MGI Symbol;Acc:MGI:109382]
Map7	-0.55	2.1E-03	microtubule-associated protein 7 [Source:MGI Symbol;Acc:MGI:1328328]
Atp5j2	-0.55	2.5E-02	ATP synthase, H+ transporting, mitochondrial F0 complex, subunit F2 [Source:MGI Symbol;Acc:MGI:1927558]
Acap1	-0.55	4.6E-02	ArfGAP with coiled-coil, ankyrin repeat and PH domains 1 [Source:MGI Symbol;Acc:MGI:2388270]
Nsdhl	-0.55	1.2E-02	NAD(P) dependent steroid dehydrogenase-like [Source:MGI Symbol;Acc:MGI:1099438]
Pafah1b3	-0.55	3.2E-03	platelet-activating factor acetylhydrolase, isoform 1b, subunit 3 [Source:MGI Symbol;Acc:MGI:108414]
Lrrc1	-0.55	3.6E-03	leucine rich repeat containing 1 [Source:MGI Symbol;Acc:MGI:2442313]
Fam160a1	-0.55	4.7E-02	family with sequence similarity 160, member A1 [Source:MGI Symbol;Acc:MGI:2444746]
Sept5	-0.55	2.2E-02	septin 5 [Source:MGI Symbol;Acc:MGI:1195461]
Ccdc34	-0.55	4.6E-02	coiled-coil domain containing 34 [Source:MGI Symbol;Acc:MGI:1915451]
Exosc9	-0.55	4.6E-02	exosome component 9 [Source:MGI Symbol;Acc:MGI:1355319]
Casp3	-0.55	4.1E-03	caspase 3 [Source:MGI Symbol;Acc:MGI:107739]
Hn1	-0.55	2.1E-02	hematological and neurological expressed sequence 1 [Source:MGI Symbol;Acc:MGI:1096361]
Ndufs4	-0.55	8.8E-03	NADH dehydrogenase (ubiquinone) Fe-S protein 4 [Source:MGI Symbol;Acc:MGI:1343135]
Gm11223	-0.55	4.7E-02	predicted gene 11223 [Source:MGI Symbol;Acc:MGI:3651631]
Hmgb2	-0.56	3.1E-02	high mobility group box 2 [Source:MGI Symbol;Acc:MGI:96157]
Scnn1a	-0.56	1.4E-03	sodium channel, nonvoltage-gated 1 alpha [Source:MGI Symbol;Acc:MGI:101782]
Clrn3	-0.56	2.2E-02	clarin 3 [Source:MGI Symbol;Acc:MGI:2142022]
Gmnn	-0.56	4.2E-02	geminin [Source:MGI Symbol;Acc:MGI:1927344]
Irf1	-0.56	1.7E-02	interferon regulatory factor 1 [Source:MGI Symbol;Acc:MGI:96590]
Mapk6	-0.56	6.7E-03	mitogen-activated protein kinase 6 [Source:MGI Symbol;Acc:MGI:1354946]
Tmsb10	-0.56	1.9E-02	thymosin, beta 10 [Source:MGI Symbol;Acc:MGI:109146]
Eno1	-0.56	3.9E-02	enolase 1, alpha non-neuron [Source:MGI Symbol;Acc:MGI:95393]
Ptp4a1	-0.56	3.1E-02	protein tyrosine phosphatase 4a1 [Source:MGI Symbol;Acc:MGI:1277096]
Car13	-0.56	1.5E-02	carbonic anhydrase 13 [Source:MGI Symbol;Acc:MGI:1931322]
Tm4sf20	-0.56	1.4E-02	transmembrane 4 L six family member 20 [Source:MGI Symbol;Acc:MGI:1913511]
Sdcbp2	-0.56	4.5E-02	syndecan binding protein (syntenin) 2 [Source:MGI Symbol;Acc:MGI:2385156]
Bak1	-0.56	4.7E-02	BCL2-antagonist/killer 1 [Source:MGI Symbol;Acc:MGI:1097161]
2010107E04Rik	-0.57	8.4E-03	RIKEN cDNA 2010107E04 gene [Source:MGI Symbol;Acc:MGI:1917507]
Bpgm	-0.57	3.8E-03	2,3-bisphosphoglycerate mutase [Source:MGI Symbol;Acc:MGI:1098242]
Elovl7	-0.57	2.1E-03	ELOVL family member 7, elongation of long chain fatty acids (yeast) [Source:MGI Symbol;Acc:MGI:1921809]
Cdkl2	-0.57	2.2E-02	cyclin-dependent kinase-like 2 (CDC2-related kinase) [Source:MGI Symbol;Acc:MGI:1858227]
Calml4	-0.57	1.9E-02	calmodulin-like 4 [Source:MGI Symbol;Acc:MGI:1922850]
Crb3	-0.57	7.6E-03	crumbs homolog 3 (Drosophila) [Source:MGI Symbol;Acc:MGI:2670904]
Srxn1	-0.57	3.1E-02	sulfiredoxin 1 homolog (S. cerevisiae) [Source:MGI Symbol;Acc:MGI:104971]
Net1	-0.57	5.9E-03	neuroepithelial cell transforming gene 1 [Source:MGI Symbol;Acc:MGI:1927138]

Nipal2	-0.57	6.7E-03	NIPA-like domain containing 2 [Source:MGI Symbol;Acc:MGI:1924488]
Hoxa7	-0.57	4.0E-02	homeobox A7 [Source:MGI Symbol;Acc:MGI:96179]
Lrrc66	-0.57	2.8E-03	leucine rich repeat containing 66 [Source:MGI Symbol;Acc:MGI:2387634]
Ovol1	-0.57	5.0E-02	OVO homolog-like 1 (Drosophila) [Source:MGI Symbol;Acc:MGI:1330290]
Acy1	-0.57	3.2E-02	aminoacylase 1 [Source:MGI Symbol;Acc:MGI:87913]
Apol6	-0.58	7.0E-03	apolipoprotein L 6 [Source:MGI Symbol;Acc:MGI:1919189]
Kctd5	-0.58	2.3E-02	potassium channel tetramerisation domain containing 5 [Source:MGI Symbol;Acc:MGI:1916509]
Dut	-0.58	2.9E-02	deoxyuridine triphosphatase [Source:MGI Symbol;Acc:MGI:1346051]
Cisd3	-0.58	2.5E-03	CDGSH iron sulfur domain 3 [Source:MGI Symbol;Acc:MGI:101788]
Rnf128	-0.58	1.1E-02	ring finger protein 128 [Source:MGI Symbol;Acc:MGI:1914139]
Slc25a24	-0.58	3.1E-02	solute carrier family 25 (mitochondrial carrier, phosphate carrier), member 24 [Source:MGI Symbol;Acc:MGI:1917160]
Cldn7	-0.58	3.7E-02	claudin 7 [Source:MGI Symbol;Acc:MGI:1859285]
Racgap1	-0.59	3.7E-02	Rac GTPase-activating protein 1 [Source:MGI Symbol;Acc:MGI:1349423]
Lpcat4	-0.59	2.8E-02	lysophosphatidylcholine acyltransferase 4 [Source:MGI Symbol;Acc:MGI:2138993]
Slc25a1	-0.59	3.4E-03	solute carrier family 25 (mitochondrial carrier, citrate transporter), member 1 [Source:MGI Symbol;Acc:MGI:1345283]
Ppfia3	-0.59	2.3E-02	protein tyrosine phosphatase, receptor type, f polypeptide (PTPRF), interacting protein (liprin), alpha 3 [Source:MGI Symbol;Acc:MGI:1924037]
Cib1	-0.59	8.8E-03	calcium and integrin binding 1 (calmyrin) [Source:MGI Symbol;Acc:MGI:1344418]
Epcam	-0.59	4.6E-02	epithelial cell adhesion molecule [Source:MGI Symbol;Acc:MGI:106653]
Dusp6	-0.59	3.1E-02	dual specificity phosphatase 6 [Source:MGI Symbol;Acc:MGI:1914853]
Rpa3	-0.59	4.1E-02	replication protein A3 [Source:MGI Symbol;Acc:MGI:1915490]
Ubald2	-0.59	8.0E-04	UBA-like domain containing 2 [Source:MGI Symbol;Acc:MGI:1914635]
Gmds	-0.59	1.0E-02	GDP-mannose 4, 6-dehydratase [Source:MGI Symbol;Acc:MGI:1891112]
Lap3	-0.60	1.7E-02	leucine aminopeptidase 3 [Source:MGI Symbol;Acc:MGI:1914238]
Tk1	-0.60	3.1E-02	thymidine kinase 1 [Source:MGI Symbol;Acc:MGI:98763]
Perp	-0.60	1.3E-02	PERP, TP53 apoptosis effector [Source:MGI Symbol;Acc:MGI:1929938]
Gm9855	-0.60	7.5E-03	predicted pseudogene 9855 [Source:MGI Symbol;Acc:MGI:3704357]
Alas1	-0.60	3.1E-02	aminolevulinic acid synthase 1 [Source:MGI Symbol;Acc:MGI:87989]
Serinc2	-0.60	3.6E-03	serine incorporator 2 [Source:MGI Symbol;Acc:MGI:1919132]
Ripk3	-0.60	2.5E-02	receptor-interacting serine-threonine kinase 3 [Source:MGI Symbol;Acc:MGI:2154952]
Tmem82	-0.61	1.9E-02	transmembrane protein 82 [Source:MGI Symbol;Acc:MGI:2384869]
Baiap2l1	-0.61	3.7E-03	BAI1-associated protein 2-like 1 [Source:MGI Symbol;Acc:MGI:1914148]
S100a10	-0.61	4.5E-02	S100 calcium binding protein A10 (calpactin) [Source:MGI Symbol;Acc:MGI:1339468]
Dbi	-0.61	1.4E-02	diazepam binding inhibitor [Source:MGI Symbol;Acc:MGI:94865]
Fa2h	-0.61	3.5E-02	fatty acid 2-hydroxylase [Source:MGI Symbol;Acc:MGI:2443327]
B3gnt3	-0.61	7.9E-03	UDP-GlcNAc:betaGal beta-1,3-N-acetylglucosaminyltransferase 3 [Source:MGI Symbol;Acc:MGI:2152535]
Pkp3	-0.61	9.4E-03	plakophilin 3 [Source:MGI Symbol;Acc:MGI:1891830]
Btnl1	-0.61	2.4E-02	butyrophilin-like 1 [Source:MGI Symbol;Acc:MGI:1932027]
Irgm1	-0.61	2.5E-02	immunity-related GTPase family M member 1 [Source:MGI Symbol;Acc:MGI:107567]
Me2	-0.62	4.8E-02	malic enzyme 2, NAD(+)-dependent, mitochondrial [Source:MGI Symbol;Acc:MGI:2147351]
Il13ra1	-0.62	4.9E-04	interleukin 13 receptor, alpha 1 [Source:MGI Symbol;Acc:MGI:105052]
Pde9a	-0.62	2.3E-02	phosphodiesterase 9A [Source:MGI Symbol;Acc:MGI:1277179]
Cldn23	-0.62	4.2E-03	claudin 23 [Source:MGI Symbol;Acc:MGI:1919158]
Chchd10	-0.62	7.1E-03	coiled-coil-helix-coiled-coil-helix domain containing 10 [Source:MGI Symbol;Acc:MGI:2143558]
Tmem170	-0.62	2.7E-02	transmembrane protein 170 [Source:MGI Symbol;Acc:MGI:106426]
Tm7sf2	-0.62	1.2E-02	transmembrane 7 superfamily member 2 [Source:MGI Symbol;Acc:MGI:1920416]
Mlkl	-0.62	4.6E-02	mixed lineage kinase domain-like [Source:MGI Symbol;Acc:MGI:1921818]
Rhpn2	-0.62	3.1E-03	rhophilin, Rho GTPase binding protein 2 [Source:MGI Symbol;Acc:MGI:1289234]
Mvk	-0.62	7.2E-03	mevalonate kinase [Source:MGI Symbol;Acc:MGI:107624]
Cdk1	-0.62	4.9E-02	cyclin-dependent kinase 1 [Source:MGI Symbol;Acc:MGI:88351]
Ccdc68	-0.62	1.9E-02	coiled-coil domain containing 68 [Source:MGI Symbol;Acc:MGI:3612676]
Tmem97	-0.62	1.3E-03	transmembrane protein 97 [Source:MGI Symbol;Acc:MGI:1916321]
Dak	-0.63	4.2E-02	dihydroxyacetone kinase 2 homolog (yeast) [Source:MGI Symbol;Acc:MGI:2385084]
Casp8	-0.63	1.4E-02	caspase 8 [Source:MGI Symbol;Acc:MGI:1261423]
Lss	-0.63	4.5E-03	lanosterol synthase [Source:MGI Symbol;Acc:MGI:1336155]

Slc39a5	-0.63	6.4E-03	solute carrier family 39 (metal ion transporter), member 5 [Source:MGI Symbol;Acc:MGI:1919336]
Mtmr11	-0.63	9.1E-04	myotubularin related protein 11 [Source:MGI Symbol;Acc:MGI:2652817]
0610007P14Rik	-0.63	1.5E-03	RIKEN cDNA 0610007P14 gene [Source:MGI Symbol;Acc:MGI:1915571]
Tmem150b	-0.63	1.6E-02	transmembrane protein 150B [Source:MGI Symbol;Acc:MGI:2679718]
Hsd17b7	-0.63	1.1E-02	hydroxysteroid (17-beta) dehydrogenase 7 [Source:MGI Symbol;Acc:MGI:1330808]
Pdss1	-0.63	7.1E-03	prenyl (solanesyl) diphosphate synthase, subunit 1 [Source:MGI Symbol;Acc:MGI:1889278]
Eno1b	-0.64	5.3E-03	enolase 1B, retrotransposed [Source:MGI Symbol;Acc:MGI:3648653]
Ripk4	-0.64	3.4E-03	receptor-interacting serine-threonine kinase 4 [Source:MGI Symbol;Acc:MGI:1919638]
Ccdc71l	-0.64	4.3E-02	coiled-coil domain containing 71 like [Source:MGI Symbol;Acc:MGI:1919373]
Cystm1	-0.64	7.2E-03	cysteine-rich transmembrane module containing 1 [Source:MGI Symbol;Acc:MGI:1913310]
Cmpk2	-0.64	4.8E-02	cytidine monophosphate (UMP-CMP) kinase 2, mitochondrial [Source:MGI Symbol;Acc:MGI:99830]
Dedd2	-0.64	8.9E-04	death effector domain-containing DNA binding protein 2 [Source:MGI Symbol;Acc:MGI:1914629]
Ccdc64b	-0.64	2.7E-02	coiled-coil domain containing 64B [Source:MGI Symbol;Acc:MGI:2388267]
Parp9	-0.64	1.8E-02	poly (ADP-ribose) polymerase family, member 9 [Source:MGI Symbol;Acc:MGI:1933117]
Insig1	-0.64	9.5E-03	insulin induced gene 1 [Source:MGI Symbol;Acc:MGI:1916289]
Gmppb	-0.64	7.0E-03	GDP-mannose pyrophosphorylase B [Source:MGI Symbol;Acc:MGI:2660880]
Hdhd3	-0.65	4.0E-02	haloacid dehalogenase-like hydrolase domain containing 3 [Source:MGI Symbol;Acc:MGI:1919998]
Vil1	-0.65	4.0E-02	villin 1 [Source:MGI Symbol;Acc:MGI:98930]
Stx3	-0.65	1.4E-03	syntaxin 3 [Source:MGI Symbol;Acc:MGI:103077]
Gm26880	-0.65	4.0E-02	predicted gene, 26880 [Source:MGI Symbol;Acc:MGI:5477374]
Egln3	-0.65	3.5E-03	egl-9 family hypoxia-inducible factor 3 [Source:MGI Symbol;Acc:MGI:1932288]
Ldha	-0.65	2.3E-02	lactate dehydrogenase A [Source:MGI Symbol;Acc:MGI:96759]
Cxcr6	-0.65	3.0E-02	chemokine (C-X-C motif) receptor 6 [Source:MGI Symbol;Acc:MGI:1934582]
Dfna5	-0.65	2.7E-02	deafness, autosomal dominant 5 (human) [Source:MGI Symbol;Acc:MGI:1889850]
Dusp8	-0.66	1.0E-02	dual specificity phosphatase 8 [Source:MGI Symbol;Acc:MGI:106626]
Arl4a	-0.66	4.5E-04	ADP-ribosylation factor-like 4A [Source:MGI Symbol;Acc:MGI:99437]
Tnfsf10	-0.66	9.3E-03	tumor necrosis factor (ligand) superfamily, member 10 [Source:MGI Symbol;Acc:MGI:107414]
Pgk1	-0.66	1.4E-02	phosphoglycerate kinase 1 [Source:MGI Symbol;Acc:MGI:97555]
Lck	-0.66	3.1E-02	lymphocyte protein tyrosine kinase [Source:MGI Symbol;Acc:MGI:96756]
Cenpv	-0.66	9.8E-03	centromere protein V [Source:MGI Symbol;Acc:MGI:1920389]
Serpinb1a	-0.66	9.5E-03	serine (or cysteine) peptidase inhibitor, clade B, member 1a [Source:MGI Symbol;Acc:MGI:1913472]
Xpnpep1	-0.66	1.6E-03	X-prolyl aminopeptidase (aminopeptidase P) 1, soluble [Source:MGI Symbol;Acc:MGI:2180003]
Nipal1	-0.66	5.3E-04	NIPA-like domain containing 1 [Source:MGI Symbol;Acc:MGI:1917951]
Fads2	-0.67	2.8E-03	fatty acid desaturase 2 [Source:MGI Symbol;Acc:MGI:1930079]
Ikzf2	-0.67	1.2E-02	IKAROS family zinc finger 2 [Source:MGI Symbol;Acc:MGI:1342541]
Anxa2	-0.67	2.8E-02	annexin A2 [Source:MGI Symbol;Acc:MGI:88246]
Marcksl1	-0.67	5.7E-03	MARCKS-like 1 [Source:MGI Symbol;Acc:MGI:97143]
Gstp2	-0.67	3.5E-02	glutathione S-transferase, pi 2 [Source:MGI Symbol;Acc:MGI:95864]
Ccng2	-0.67	3.5E-03	cyclin G2 [Source:MGI Symbol;Acc:MGI:1095734]
Uqcrfs1	-0.67	3.5E-03	ubiquinol-cytochrome c reductase, Rieske iron-sulfur polypeptide 1 [Source:MGI Symbol;Acc:MGI:1913944]
Ube2c	-0.67	4.7E-02	ubiquitin-conjugating enzyme E2C [Source:MGI Symbol;Acc:MGI:1915862]
Mxi1	-0.67	1.4E-03	Max interacting protein 1 [Source:MGI Symbol;Acc:MGI:97245]
Cycs	-0.67	3.3E-02	cytochrome c, somatic [Source:MGI Symbol;Acc:MGI:88578]
Agpat2	-0.67	4.2E-03	1-acylglycerol-3-phosphate O-acyltransferase 2 (lysophosphatidic acid acyltransferase, beta) [Source:MGI Symbol;Acc:MGI:1914762]
Prdm1	-0.67	1.2E-02	PR domain containing 1, with ZNF domain [Source:MGI Symbol;Acc:MGI:99655]
Ano9	-0.68	1.9E-04	anoctamin 9 [Source:MGI Symbol;Acc:MGI:1918595]
Psmb9	-0.68	3.7E-02	proteasome (prosome, macropain) subunit, beta type 9 (large multifunctional peptidase 2) [Source:MGI Symbol;Acc:MGI:1346526]
Pgd	-0.68	1.2E-02	phosphogluconate dehydrogenase [Source:MGI Symbol;Acc:MGI:97553]
Skil	-0.68	1.1E-02	SKI-like [Source:MGI Symbol;Acc:MGI:106203]
Trim31	-0.68	1.1E-02	tripartite motif-containing 31 [Source:MGI Symbol;Acc:MGI:2385051]
Gimap3	-0.68	3.6E-02	GTPase, IMAP family member 3 [Source:MGI Symbol;Acc:MGI:1932723]

Ppa1	-0.69	2.2E-02	pyrophosphatase (inorganic) 1 [Source:MGI Symbol;Acc:MGI:97831]
Slc25a15	-0.69	2.5E-03	solute carrier family 25 (mitochondrial carrier ornithine transporter), member 15 [Source:MGI Symbol;Acc:MGI:1342274]
Dusp5	-0.69	2.6E-02	dual specificity phosphatase 5 [Source:MGI Symbol;Acc:MGI:2685183]
Myo1a	-0.69	1.7E-02	myosin IA [Source:MGI Symbol;Acc:MGI:107732]
Aacs	-0.69	1.3E-03	acetoacetyl-CoA synthetase [Source:MGI Symbol;Acc:MGI:1926144]
Gale	-0.70	7.8E-03	galactose-4-epimerase, UDP [Source:MGI Symbol;Acc:MGI:1921496]
Duox2	-0.70	9.8E-03	dual oxidase 2 [Source:MGI Symbol;Acc:MGI:3036280]
Birc5	-0.70	4.4E-02	baculoviral IAP repeat-containing 5 [Source:MGI Symbol;Acc:MGI:1203517]
Panx1	-0.70	1.5E-03	pannexin 1 [Source:MGI Symbol;Acc:MGI:1860055]
Tap1	-0.71	3.9E-02	transporter 1, ATP-binding cassette, sub-family B (MDR/TAP) [Source:MGI Symbol;Acc:MGI:98483]
Senp3	-0.71	3.7E-06	SUMO/sentrin specific peptidase 3 [Source:MGI Symbol;Acc:MGI:2158736]
Tfrc	-0.71	3.5E-02	transferrin receptor [Source:MGI Symbol;Acc:MGI:98822]
Tmem236	-0.71	8.6E-03	transmembrane protein 236 [Source:MGI Symbol;Acc:MGI:1919309]
Agpat9	-0.71	9.7E-03	1-acylglycerol-3-phosphate O-acyltransferase 9 [Source:MGI Symbol;Acc:MGI:3603816]
Fam162a	-0.71	2.3E-02	family with sequence similarity 162, member A [Source:MGI Symbol;Acc:MGI:1917436]
Gcat	-0.71	2.8E-02	glycine C-acetyltransferase (2-amino-3-ketobutyrate-coenzyme A ligase) [Source:MGI Symbol;Acc:MGI:1349389]
Igkc	-0.72	2.2E-02	immunoglobulin kappa constant [Source:MGI Symbol;Acc:MGI:96495]
Gm5431	-0.72	4.0E-02	predicted gene 5431 [Source:MGI Symbol;Acc:MGI:3645205]
S100a16	-0.72	1.2E-03	S100 calcium binding protein A16 [Source:MGI Symbol;Acc:MGI:1915110]
Nckap5	-0.72	1.1E-02	NCK-associated protein 5 [Source:MGI Symbol;Acc:MGI:2686394]
Pcyt2	-0.72	6.3E-04	phosphate cytidylyltransferase 2, ethanolamine [Source:MGI Symbol;Acc:MGI:1915921]
Gpr160	-0.72	2.4E-03	G protein-coupled receptor 160 [Source:MGI Symbol;Acc:MGI:1919112]
Hk2	-0.73	4.4E-02	hexokinase 2 [Source:MGI Symbol;Acc:MGI:1315197]
Anxa4	-0.73	5.9E-04	annexin A4 [Source:MGI Symbol;Acc:MGI:88030]
Abhd11os	-0.73	4.8E-03	abhydrolase domain containing 11, opposite strand [Source:MGI Symbol;Acc:MGI:1917062]
Zap70	-0.73	4.3E-02	zeta-chain (TCR) associated protein kinase [Source:MGI Symbol;Acc:MGI:99613]
Ppp1r14d	-0.73	8.8E-03	protein phosphatase 1, regulatory (inhibitor) subunit 14D [Source:MGI Symbol;Acc:MGI:1919362]
Ovol2	-0.73	2.4E-02	ovo-like 2 (Drosophila) [Source:MGI Symbol;Acc:MGI:1338039]
1700066B19Rik	-0.74	3.1E-02	RIKEN cDNA 1700066B19 gene [Source:MGI Symbol;Acc:MGI:1920699]
Trim40	-0.74	3.9E-02	tripartite motif-containing 40 [Source:MGI Symbol;Acc:MGI:2684881]
Hspa12a	-0.74	2.2E-03	heat shock protein 12A [Source:MGI Symbol;Acc:MGI:1920692]
Gch1	-0.74	3.8E-02	GTP cyclohydrolase 1 [Source:MGI Symbol;Acc:MGI:95675]
Cideb	-0.74	1.3E-02	cell death-inducing DNA fragmentation factor, alpha subunit-like effector B [Source:MGI Symbol;Acc:MGI:1270844]
BC016579	-0.74	3.5E-03	cDNA sequence, BC016579 [Source:MGI Symbol;Acc:MGI:2384848]
Nos2	-0.75	2.7E-02	nitric oxide synthase 2, inducible [Source:MGI Symbol;Acc:MGI:97361]
Pfkp	-0.75	1.4E-03	phosphofructokinase, platelet [Source:MGI Symbol;Acc:MGI:1891833]
Nrap	-0.75	3.4E-02	nebulin-related anchoring protein [Source:MGI Symbol;Acc:MGI:1098765]
Casp7	-0.75	1.3E-03	caspase 7 [Source:MGI Symbol;Acc:MGI:109383]
Aprt	-0.75	3.4E-03	adenine phosphoribosyl transferase [Source:MGI Symbol;Acc:MGI:88061]
Spc25	-0.75	2.6E-02	SPC25, NDC80 kinetochore complex component, homolog (S. cerevisiae) [Source:MGI Symbol;Acc:MGI:1913692]
Cdkn1a	-0.75	8.2E-03	cyclin-dependent kinase inhibitor 1A (P21) [Source:MGI Symbol;Acc:MGI:104556]
Gm11545	-0.75	1.2E-02	predicted gene 11545 [Source:MGI Symbol;Acc:MGI:2144683]
Sc5d	-0.75	2.7E-05	sterol-C5-desaturase (fungal ERG3, delta-5-desaturase) homolog (S. cerevisae) [Source:MGI Symbol;Acc:MGI:1353611]
Tmcc3	-0.76	8.8E-03	transmembrane and coiled coil domains 3 [Source:MGI Symbol;Acc:MGI:2442900]
Pxdc1	-0.76	1.7E-02	PX domain containing 1 [Source:MGI Symbol;Acc:MGI:1914145]
Plekhn1	-0.76	9.7E-03	pleckstrin homology domain containing, family N member 1 [Source:MGI Symbol;Acc:MGI:2387630]
Sqle	-0.76	2.2E-03	squalene epoxidase [Source:MGI Symbol;Acc:MGI:109296]
#N/A	-0.77	3.5E-02	#N/A
Batf2	-0.77	1.7E-02	basic leucine zipper transcription factor, ATF-like 2 [Source:MGI Symbol;Acc:MGI:1921731]
Fads1	-0.77	5.2E-03	fatty acid desaturase 1 [Source:MGI Symbol;Acc:MGI:1923517]
Rhou	-0.78	7.1E-03	ras homolog gene family, member U [Source:MGI Symbol;Acc:MGI:1916831]
Fam64a	-0.78	5.0E-02	family with sequence similarity 64, member A [Source:MGI Symbol;Acc:MGI:1924434]

Cwh43	-0.78	4.3E-02	cell wall biogenesis 43 C-terminal homolog (S. cerevisiae) [Source:MGI Symbol;Acc:MGI:2444131]
Eci3	-0.78	4.9E-02	enoyl-Coenzyme A delta isomerase 3 [Source:MGI Symbol;Acc:MGI:1916373]
Baiap2l2	-0.78	5.5E-04	BAI1-associated protein 2-like 2 [Source:MGI Symbol;Acc:MGI:2652819]
Dpf3	-0.78	4.7E-02	D4, zinc and double PHD fingers, family 3 [Source:MGI Symbol;Acc:MGI:1917377]
Zc3h12d	-0.79	3.3E-03	zinc finger CCCH type containing 12D [Source:MGI Symbol;Acc:MGI:3045313]
Socs1	-0.79	9.9E-03	suppressor of cytokine signaling 1 [Source:MGI Symbol;Acc:MGI:1354910]
Aldh3b2	-0.80	1.8E-02	aldehyde dehydrogenase 3 family, member B2 [Source:MGI Symbol;Acc:MGI:2147613]
Sprr2a3	-0.80	3.4E-02	small proline-rich protein 2A3 [Source:MGI Symbol;Acc:MGI:3845028]
Apol7a	-0.80	4.6E-02	apolipoprotein L 7a [Source:MGI Symbol;Acc:MGI:1923011]
Wars	-0.81	2.2E-03	tryptophanyl-tRNA synthetase [Source:MGI Symbol;Acc:MGI:104630]
Msmo1	-0.81	3.9E-03	methylsterol monoxygenase 1 [Source:MGI Symbol;Acc:MGI:1913484]
Il1rn	-0.81	2.2E-02	interleukin 1 receptor antagonist [Source:MGI Symbol;Acc:MGI:96547]
Muc3	-0.81	4.3E-02	mucin 3, intestinal [Source:MGI Symbol;Acc:MGI:1203527]
Pls1	-0.81	6.2E-03	plastin 1 (I-isoform) [Source:MGI Symbol;Acc:MGI:104809]
Acat2	-0.81	1.9E-04	acetyl-Coenzyme A acetyltransferase 2 [Source:MGI Symbol;Acc:MGI:87871]
Gadd45a	-0.81	4.2E-03	growth arrest and DNA-damage-inducible 45 alpha [Source:MGI Symbol;Acc:MGI:107799]
Txn1	-0.82	2.0E-03	thioredoxin 1 [Source:MGI Symbol;Acc:MGI:98874]
Lgals9	-0.82	5.2E-04	lectin, galactose binding, soluble 9 [Source:MGI Symbol;Acc:MGI:109496]
Fdft1	-0.83	3.8E-04	farnesyl diphosphate farnesyl transferase 1 [Source:MGI Symbol;Acc:MGI:102706]
AW112010	-0.83	1.8E-04	expressed sequence AW112010 [Source:MGI Symbol;Acc:MGI:2147706]
Krt20	-0.83	4.9E-02	keratin 20 [Source:MGI Symbol;Acc:MGI:1914059]
Pif1	-0.83	3.7E-02	PIF1 5'-to-3' DNA helicase homolog (S. cerevisiae) [Source:MGI Symbol;Acc:MGI:2143057]
Ptrh1	-0.83	2.2E-02	peptidyl-tRNA hydrolase 1 homolog (S. cerevisiae) [Source:MGI Symbol;Acc:MGI:1913779]
B3gnt5	-0.83	8.9E-03	UDP-GlcNAc:betaGal beta-1,3-N-acetylglucosaminyltransferase 5 [Source:MGI Symbol;Acc:MGI:2137302]
Cd3e	-0.83	1.6E-02	CD3 antigen, epsilon polypeptide [Source:MGI Symbol;Acc:MGI:88332]
Rnf125	-0.84	2.9E-02	ring finger protein 125 [Source:MGI Symbol;Acc:MGI:1914914]
Gsdmc3	-0.84	6.1E-03	gasdermin C3 [Source:MGI Symbol;Acc:MGI:3580656]
Amn	-0.84	3.3E-02	amnionless [Source:MGI Symbol;Acc:MGI:1934943]
Tmem253	-0.84	9.1E-03	transmembrane protein 253 [Source:MGI Symbol;Acc:MGI:3588246]
Mkrn2os	-0.84	3.7E-05	makorin, ring finger protein 2, opposite strand [Source:MGI Symbol;Acc:MGI:1917541]
Hmgcr	-0.85	1.7E-04	3-hydroxy-3-methylglutaryl-Coenzyme A reductase [Source:MGI Symbol;Acc:MGI:96159]
Hist1h2ap	-0.85	4.0E-02	histone cluster 1, H2ap [Source:MGI Symbol;Acc:MGI:3710573]
Pmaip1	-0.85	5.2E-03	phorbol-12-myristate-13-acetate-induced protein 1 [Source:MGI Symbol;Acc:MGI:1930146]
Spats2l	-0.85	1.3E-04	spermatogenesis associated, serine-rich 2-like [Source:MGI Symbol;Acc:MGI:1914448]
Pmvk	-0.86	9.9E-05	phosphomevalonate kinase [Source:MGI Symbol;Acc:MGI:1915853]
Cd3d	-0.86	4.4E-02	CD3 antigen, delta polypeptide [Source:MGI Symbol;Acc:MGI:88331]
Prelid2	-0.87	1.1E-02	PRELI domain containing 2 [Source:MGI Symbol;Acc:MGI:1924869]
Cenph	-0.87	2.6E-02	centromere protein H [Source:MGI Symbol;Acc:MGI:1349448]
Hnf4g	-0.87	4.7E-04	hepatocyte nuclear factor 4, gamma [Source:MGI Symbol;Acc:MGI:1353604]
Sytl3	-0.87	2.5E-02	synaptotagmin-like 3 [Source:MGI Symbol;Acc:MGI:1933367]
Mvd	-0.87	1.4E-04	mevalonate (diphospho) decarboxylase [Source:MGI Symbol;Acc:MGI:2179327]
Glod5	-0.88	4.5E-03	glyoxalase domain containing 5 [Source:MGI Symbol;Acc:MGI:1917074]
Slfn4	-0.88	2.7E-02	schlafen 4 [Source:MGI Symbol;Acc:MGI:1329010]
Sprr2a2	-0.88	3.3E-02	small proline-rich protein 2A2 [Source:MGI Symbol;Acc:MGI:3845026]
Tmc7	-0.88	1.7E-02	transmembrane channel-like gene family 7 [Source:MGI Symbol;Acc:MGI:2443317]
H2-M2	-0.89	1.3E-02	histocompatibility 2, M region locus 2 [Source:MGI Symbol;Acc:MGI:95914]
Btnl2	-0.89	6.9E-04	butyrophilin-like 2 [Source:MGI Symbol;Acc:MGI:1859549]
2810417H13Rik	-0.89	1.5E-02	RIKEN cDNA 2810417H13 gene [Source:MGI Symbol;Acc:MGI:1915276]
Abhd3	-0.89	2.8E-02	abhydrolase domain containing 3 [Source:MGI Symbol;Acc:MGI:2147183]
Dusp4	-0.90	1.9E-02	dual specificity phosphatase 4 [Source:MGI Symbol;Acc:MGI:2442191]
Spink4	-0.90	4.1E-02	serine peptidase inhibitor, Kazal type 4 [Source:MGI Symbol;Acc:MGI:1341848]
Xcl1	-0.91	4.4E-02	chemokine (C motif) ligand 1 [Source:MGI Symbol;Acc:MGI:104593]
Trim29	-0.92	3.7E-02	tripartite motif-containing 29 [Source:MGI Symbol;Acc:MGI:1919419]
Itgb6	-0.92	1.9E-03	integrin beta 6 [Source:MGI Symbol;Acc:MGI:96615]
Slpi	-0.92	8.9E-03	secretory leukocyte peptidase inhibitor [Source:MGI Symbol;Acc:MGI:109297]

Cyp51	-0.92	5.1E-05	cytochrome P450, family 51 [Source:MGI Symbol;Acc:MGI:106040]
Scd2	-0.94	2.7E-03	stearoyl-Coenzyme A desaturase 2 [Source:MGI Symbol;Acc:MGI:98240]
Apol7b	-0.94	4.3E-02	apolipoprotein L 7b [Source:MGI Symbol;Acc:MGI:3583950]
Apol7c	-0.94	2.6E-02	apolipoprotein L 7c [Source:MGI Symbol;Acc:MGI:1920912]
Apol10b	-0.95	2.5E-03	apolipoprotein L 10B [Source:MGI Symbol;Acc:MGI:3043522]
Hmgcs1	-0.95	1.6E-03	3-hydroxy-3-methylglutaryl-Coenzyme A synthase 1 [Source:MGI Symbol;Acc:MGI:107592]
Amica1	-0.95	8.5E-03	adhesion molecule, interacts with CXADR antigen 1 [Source:MGI Symbol;Acc:MGI:2685484]
Sct	-0.96	1.3E-03	secretin [Source:MGI Symbol;Acc:MGI:99466]
Itgae	-0.96	4.5E-03	integrin alpha E, epithelial-associated [Source:MGI Symbol;Acc:MGI:1298377]
Gp1bb	-0.96	2.4E-02	glycoprotein Ib, beta polypeptide [Source:MGI Symbol;Acc:MGI:107852]
Egr2	-0.96	4.9E-02	early growth response 2 [Source:MGI Symbol;Acc:MGI:95296]
Suox	-0.96	4.0E-02	sulfite oxidase [Source:MGI Symbol;Acc:MGI:2446117]
1500009L16Rik	-0.96	8.9E-03	RIKEN cDNA 1500009L16 gene [Source:MGI Symbol;Acc:MGI:1917034]
Muc4	-0.97	1.7E-05	mucin 4 [Source:MGI Symbol;Acc:MGI:2153525]
Sprr2a1	-0.97	8.0E-03	small proline-rich protein 2A1 [Source:MGI Symbol;Acc:MGI:1330350]
Idi1	-0.98	3.5E-05	isopentenyl-diphosphate delta isomerase [Source:MGI Symbol;Acc:MGI:2442264]
Cd3g	-0.99	2.6E-03	CD3 antigen, gamma polypeptide [Source:MGI Symbol;Acc:MGI:88333]
Pmp22	-0.99	2.3E-02	peripheral myelin protein 22 [Source:MGI Symbol;Acc:MGI:97631]
Gpnmb	-1.00	1.2E-02	glycoprotein (transmembrane) nmb [Source:MGI Symbol;Acc:MGI:1934765]
Sult2b1	-1.00	1.1E-02	sulfotransferase family, cytosolic, 2B, member 1 [Source:MGI Symbol;Acc:MGI:1926342]
Pla2g3	-1.00	1.9E-02	phospholipase A2, group III [Source:MGI Symbol;Acc:MGI:2444945]
Lif	-1.01	7.1E-03	leukemia inhibitory factor [Source:MGI Symbol;Acc:MGI:96787]
S100a14	-1.02	9.2E-03	S100 calcium binding protein A14 [Source:MGI Symbol;Acc:MGI:1913416]
Cxcl9	-1.02	4.6E-02	chemokine (C-X-C motif) ligand 9 [Source:MGI Symbol;Acc:MGI:1352449]
Unc5cl	-1.02	8.6E-03	unc-5 homolog C (C. elegans)-like [Source:MGI Symbol;Acc:MGI:1923839]
Mmp12	-1.02	2.9E-02	matrix metallopeptidase 12 [Source:MGI Symbol;Acc:MGI:97005]
Fdps	-1.03	2.8E-06	farnesyl diphosphate synthetase [Source:MGI Symbol;Acc:MGI:104888]
Sec14l2	-1.03	1.7E-02	SEC14-like 2 (S. cerevisiae) [Source:MGI Symbol;Acc:MGI:1915065]
Far2	-1.03	1.1E-03	fatty acyl CoA reductase 2 [Source:MGI Symbol;Acc:MGI:2687035]
Mfsd2a	-1.04	2.8E-02	major facilitator superfamily domain containing 2A [Source:MGI Symbol;Acc:MGI:1923824]
Xkr9	-1.04	2.6E-02	X Kell blood group precursor related family member 9 homolog [Source:MGI Symbol;Acc:MGI:2686466]
Fos	-1.04	2.0E-02	FBJ osteosarcoma oncogene [Source:MGI Symbol;Acc:MGI:95574]
Dcstamp	-1.04	2.8E-02	dentrocyte expressed seven transmembrane protein [Source:MGI Symbol;Acc:MGI:1923016]
Cacna1s	-1.04	1.7E-02	calcium channel, voltage-dependent, L type, alpha 1S subunit [Source:MGI Symbol;Acc:MGI:88294]
Ifit2	-1.04	1.4E-05	interferon-induced protein with tetratricopeptide repeats 2 [Source:MGI Symbol;Acc:MGI:99449]
Sectm1a	-1.05	2.9E-03	secreted and transmembrane 1A [Source:MGI Symbol;Acc:MGI:2384805]
Ido1	-1.06	2.5E-02	indoleamine 2,3-dioxygenase 1 [Source:MGI Symbol;Acc:MGI:96416]
Rnf152	-1.06	3.3E-02	ring finger protein 152 [Source:MGI Symbol;Acc:MGI:2443787]
Tcrg-C1	-1.06	9.5E-03	T cell receptor gamma, constant 1 [Source:MGI Symbol;Acc:MGI:98625]
Igkv4-91	-1.06	3.6E-02	immunoglobulin kappa chain variable 4-91 [Source:MGI Symbol;Acc:MGI:3642277]
Pgk1-rs7	-1.06	2.1E-02	phosphoglycerate kinase-1, related sequence-7 [Source:MGI Symbol;Acc:MGI:97562]
Duoxa2	-1.06	1.5E-03	dual oxidase maturation factor 2 [Source:MGI Symbol;Acc:MGI:1914061]
Myl7	-1.07	1.6E-02	myosin, light polypeptide 7, regulatory [Source:MGI Symbol;Acc:MGI:107495]
Ighv14-3	-1.07	1.4E-02	immunoglobulin heavy variable V14-3 [Source:MGI Symbol;Acc:MGI:4439764]
Gm14137	-1.07	5.5E-03	predicted gene 14137 [Source:MGI Symbol;Acc:MGI:3651144]
Tnip3	-1.08	2.5E-03	TNFAIP3 interacting protein 3 [Source:MGI Symbol;Acc:MGI:3041165]
Cldn4	-1.09	1.3E-03	claudin 4 [Source:MGI Symbol;Acc:MGI:1313314]
Igkv6-15	-1.09	4.4E-04	immunoglobulin kappa variable 6-15 [Source:MGI Symbol;Acc:MGI:1330831]
Tgtp1	-1.09	4.9E-02	T cell specific GTPase 1 [Source:MGI Symbol;Acc:MGI:98734]
Inf2	-1.09	2.1E-03	inverted formin, FH2 and WH2 domain containing [Source:MGI Symbol;Acc:MGI:1917685]
Ctsw	-1.10	5.6E-04	cathepsin W [Source:MGI Symbol;Acc:MGI:1338045]
Cox7a1	-1.11	2.2E-03	cytochrome c oxidase subunit VIIa 1 [Source:MGI Symbol;Acc:MGI:1316714]
Apob	-1.12	2.9E-02	apolipoprotein B [Source:MGI Symbol;Acc:MGI:88052]
Il1r2	-1.12	3.1E-02	interleukin 1 receptor, type II [Source:MGI Symbol;Acc:MGI:96546]
Oasl1	-1.13	1.1E-03	2'-5' oligoadenylate synthetase-like 1 [Source:MGI Symbol;Acc:MGI:2180849]
Soat2	-1.14	8.5E-03	sterol O-acyltransferase 2 [Source:MGI Symbol;Acc:MGI:1332226]

Frmd5	-1.14	9.7E-03	FERM domain containing 5 [Source:MGI Symbol;Acc:MGI:2442557]
Cd160	-1.14	2.0E-03	CD160 antigen [Source:MGI Symbol;Acc:MGI:1860383]
Apol9b	-1.15	2.6E-02	apolipoprotein L 9b [Source:MGI Symbol;Acc:MGI:1919148]
Klrd1	-1.15	4.4E-02	killer cell lectin-like receptor, subfamily D, member 1 [Source:MGI Symbol;Acc:MGI:1196275]
Tcrg-C4	-1.15	1.1E-02	T cell receptor gamma, constant 4 [Source:MGI Symbol;Acc:MGI:98628]
Plaur	-1.16	1.3E-03	plasminogen activator, urokinase receptor [Source:MGI Symbol;Acc:MGI:97612]
Cd7	-1.16	1.3E-03	CD7 antigen [Source:MGI Symbol;Acc:MGI:88344]
Ttc16	-1.17	2.2E-02	tetratricopeptide repeat domain 16 [Source:MGI Symbol;Acc:MGI:2443048]
Aldh1l1	-1.18	1.3E-03	aldehyde dehydrogenase 1 family, member L1 [Source:MGI Symbol;Acc:MGI:1340024]
Gm13237	-1.18	1.1E-02	predicted gene 13237 [Source:MGI Symbol;Acc:MGI:3649924]
Ighv9-3	-1.18	1.3E-03	immunoglobulin heavy variable V9-3 [Source:MGI Symbol;Acc:MGI:3642720]
Tnfrsf9	-1.19	2.7E-02	tumor necrosis factor receptor superfamily, member 9 [Source:MGI Symbol;Acc:MGI:1101059]
Ighv5-6	-1.20	4.9E-03	immunoglobulin heavy variable 5-6 [Source:MGI Symbol;Acc:MGI:4439815]
Clcn1	-1.20	3.2E-02	chloride channel 1 [Source:MGI Symbol;Acc:MGI:88417]
Tnfsf13	-1.20	6.9E-04	tumor necrosis factor (ligand) superfamily, member 13 [Source:MGI Symbol;Acc:MGI:1916833]
Cst7	-1.21	7.0E-03	cystatin F (leukocystatin) [Source:MGI Symbol;Acc:MGI:1298217]
9330159N05Rik	-1.25	1.7E-02	RIKEN cDNA 9330159N05 gene [Source:MGI Symbol;Acc:MGI:2441739]
Ighv6-6	-1.25	2.2E-02	immunoglobulin heavy variable 6-6 [Source:MGI Symbol;Acc:MGI:4439619]
Igkv12-44	-1.26	4.9E-03	immunoglobulin kappa variable 12-44 [Source:MGI Symbol;Acc:MGI:4439775]
Slc16a3	-1.26	4.1E-04	solute carrier family 16 (monocarboxylic acid transporters), member 3 [Source:MGI Symbol;Acc:MGI:1933438]
Ighv1-55	-1.28	2.5E-02	immunoglobulin heavy variable 1-55 [Source:MGI Symbol;Acc:MGI:4439716]
Igkv10-96	-1.29	2.0E-02	immunoglobulin kappa variable 10-96 [Source:MGI Symbol;Acc:MGI:4439561]
F10	-1.29	3.7E-02	coagulation factor X [Source:MGI Symbol;Acc:MGI:103107]
Pla2g2d	-1.29	1.3E-03	phospholipase A2, group IID [Source:MGI Symbol;Acc:MGI:1341796]
Gad2	-1.29	4.8E-03	glutamic acid decarboxylase 2 [Source:MGI Symbol;Acc:MGI:95634]
Crtam	-1.30	3.2E-02	cytotoxic and regulatory T cell molecule [Source:MGI Symbol;Acc:MGI:1859822]
Plekhs1	-1.30	1.4E-03	pleckstrin homology domain containing, family S member 1 [Source:MGI Symbol;Acc:MGI:2443041]
Tm6sf2	-1.30	1.3E-02	transmembrane 6 superfamily member 2 [Source:MGI Symbol;Acc:MGI:1933210]
Slc27a2	-1.31	5.4E-04	solute carrier family 27 (fatty acid transporter), member 2 [Source:MGI Symbol;Acc:MGI:1347099]
2210407C18Rik	-1.31	1.1E-06	RIKEN cDNA 2210407C18 gene [Source:MGI Symbol;Acc:MGI:1925604]
Tm4sf5	-1.31	1.9E-02	transmembrane 4 superfamily member 5 [Source:MGI Symbol;Acc:MGI:1922854]
Areg	-1.32	4.3E-02	amphiregulin [Source:MGI Symbol;Acc:MGI:88068]
Prap1	-1.33	3.5E-02	proline-rich acidic protein 1 [Source:MGI Symbol;Acc:MGI:893573]
Sun3	-1.33	1.1E-02	Sad1 and UNC84 domain containing 3 [Source:MGI Symbol;Acc:MGI:3041199]
Rbp2	-1.34	3.8E-03	retinol binding protein 2, cellular [Source:MGI Symbol;Acc:MGI:97877]
Klrg1	-1.34	4.2E-03	killer cell lectin-like receptor subfamily G, member 1 [Source:MGI Symbol;Acc:MGI:1355294]
Ighv1-14	-1.35	1.5E-02	immunoglobulin heavy variable 1-14 [Source:MGI Symbol;Acc:MGI:4439781]
Apoc2	-1.35	4.6E-02	apolipoprotein C-II [Source:MGI Symbol;Acc:MGI:88054]
Pcsk9	-1.35	1.2E-04	proprotein convertase subtilisin/kexin type 9 [Source:MGI Symbol;Acc:MGI:2140260]
Ocstamp	-1.35	4.5E-02	osteoclast stimulatory transmembrane protein [Source:MGI Symbol;Acc:MGI:1921864]
Prss16	-1.37	3.3E-02	protease, serine 16 (thymus) [Source:MGI Symbol;Acc:MGI:1859181]
Ighv1-77	-1.38	2.8E-02	immunoglobulin heavy variable 1-77 [Source:MGI Symbol;Acc:MGI:4439670]
Spo11	-1.38	1.7E-02	SPO11 meiotic protein covalently bound to DSB homolog (S. cerevisiae) [Source:MGI Symbol;Acc:MGI:1349669]
Creb3l3	-1.39	1.5E-02	cAMP responsive element binding protein 3-like 3 [Source:MGI Symbol;Acc:MGI:2384786]
Sprr2h	-1.41	8.5E-03	small proline-rich protein 2H [Source:MGI Symbol;Acc:MGI:1330343]
Slc26a6	-1.42	2.3E-02	solute carrier family 26, member 6 [Source:MGI Symbol;Acc:MGI:2159728]
Tnfsf12	-1.46	7.8E-07	tumor necrosis factor (ligand) superfamily, member 12 [Source:MGI Symbol;Acc:MGI:1196259]
Igkv12-46	-1.46	6.6E-03	immunoglobulin kappa variable 12-46 [Source:MGI Symbol;Acc:MGI:4439773]
Scnn1g	-1.48	2.2E-02	sodium channel, nonvoltage-gated 1 gamma [Source:MGI Symbol;Acc:MGI:104695]
Arl13a	-1.51	6.1E-03	ADP-ribosylation factor-like 13A [Source:MGI Symbol;Acc:MGI:1921698]
Cxcl11	-1.51	1.6E-03	chemokine (C-X-C motif) ligand 11 [Source:MGI Symbol;Acc:MGI:1860203]
Mt4	-1.56	4.9E-02	metallothionein 4 [Source:MGI Symbol;Acc:MGI:99692]

Sprr1a	-1.59	1.7E-04	small proline-rich protein 1A [Source:MGI Symbol;Acc:MGI:106660]
Gm11992	-1.60	5.4E-04	predicted gene 11992 [Source:MGI Symbol;Acc:MGI:3651127]
Nkg7	-1.61	4.0E-05	natural killer cell group 7 sequence [Source:MGI Symbol;Acc:MGI:1931250]
Upp1	-1.69	2.1E-07	uridine phosphorylase 1 [Source:MGI Symbol;Acc:MGI:1097668]
Trgv2	-1.70	5.0E-03	T cell receptor gamma variable 2 [Source:MGI Symbol;Acc:MGI:98632]
Erich4	-1.76	1.3E-02	glutamate rich 4 [Source:MGI Symbol;Acc:MGI:3646269]
Igkv3-12	-1.78	5.3E-06	immunoglobulin kappa variable 3-12 [Source:MGI Symbol;Acc:MGI:1330815]
Abca13	-1.78	1.8E-05	ATP-binding cassette, sub-family A (ABC1), member 13 [Source:MGI Symbol;Acc:MGI:2388707]
Gad1-ps	-1.79	2.9E-03	glutamate decarboxylase 1, pseudogene [Source:MGI Symbol;Acc:MGI:95633]
Bcl2l15	-1.82	1.6E-02	BCLl2-like 15 [Source:MGI Symbol;Acc:MGI:2685412]
Cd8b1	-1.83	4.4E-04	CD8 antigen, beta chain 1 [Source:MGI Symbol;Acc:MGI:88347]
Pla2g4c	-1.84	4.3E-02	phospholipase A2, group IVC (cytosolic, calcium-independent) [Source:MGI Symbol;Acc:MGI:1196403]
Ccl25	-1.85	4.0E-03	chemokine (C-C motif) ligand 25 [Source:MGI Symbol;Acc:MGI:1099448]
Cd8a	-1.86	5.3E-04	CD8 antigen, alpha chain [Source:MGI Symbol;Acc:MGI:88346]
Gzmb	-1.86	5.1E-03	granzyme B [Source:MGI Symbol;Acc:MGI:109267]
Mogat2	-1.87	8.4E-03	monoacylglycerol O-acyltransferase 2 [Source:MGI Symbol;Acc:MGI:2663253]
Tcrg-C2	-1.91	3.2E-05	T-cell receptor gamma, constant 2 [Source:MGI Symbol;Acc:MGI:98626]
Ms4a18	-1.92	1.0E-03	membrane-spanning 4-domains, subfamily A, member 18 [Source:MGI Symbol;Acc:MGI:1923252]
Ccl5	-1.96	5.4E-04	chemokine (C-C motif) ligand 5 [Source:MGI Symbol;Acc:MGI:98262]
Igkv8-21	-2.02	4.1E-02	immunoglobulin kappa variable 8-21 [Source:MGI Symbol;Acc:MGI:1330840]
Barx2	-2.15	1.5E-04	BarH-like homeobox 2 [Source:MGI Symbol;Acc:MGI:109617]
Igkv6-13	-2.25	4.5E-04	immunoglobulin kappa variable 6-13 [Source:MGI Symbol;Acc:MGI:1330829]
Cst6	-2.27	2.8E-06	cystatin E/M [Source:MGI Symbol;Acc:MGI:1920970]
Ggt1	-2.28	8.3E-03	gamma-glutamyltransferase 1 [Source:MGI Symbol;Acc:MGI:95706]
Igkv4-70	-2.42	1.5E-02	immunoglobulin kappa chain variable 4-70 [Source:MGI Symbol;Acc:MGI:2686348]
Sis	-2.44	4.6E-02	sucrase isomaltase (alpha-glucosidase) [Source:MGI Symbol;Acc:MGI:1917233]
Gzmk	-2.45	4.8E-03	granzyme K [Source:MGI Symbol;Acc:MGI:1298232]
Cd163l1	-2.48	1.4E-03	CD163 molecule-like 1 [Source:MGI Symbol;Acc:MGI:2443796]
Igkv4-50	-2.55	1.7E-02	immunoglobulin kappa variable 4-50 [Source:MGI Symbol;Acc:MGI:2685915]
Mttp	-2.66	1.7E-03	microsomal triglyceride transfer protein [Source:MGI Symbol;Acc:MGI:106926]
Ighv9-4	-2.78	2.9E-02	immunoglobulin heavy variable 9-4 [Source:MGI Symbol;Acc:MGI:3646379]
Anpep	-2.80	4.4E-04	alanyl (membrane) aminopeptidase [Source:MGI Symbol;Acc:MGI:5000466]
Ighv7-2	-3.14	3.3E-03	immunoglobulin heavy variable 7-2 [Source:MGI Symbol;Acc:MGI:4439623]
Ighv7-3	-3.19	5.9E-03	immunoglobulin heavy variable 7-3 [Source:MGI Symbol;Acc:MGI:4439766]
Slc6a19	-3.65	1.9E-04	solute carrier family 6 (neurotransmitter transporter), member 19 [Source:MGI Symbol;Acc:MGI:1921588]
Fabp6	-3.81	1.8E-03	fatty acid binding protein 6, ileal (gastrotropin) [Source:MGI Symbol;Acc:MGI:96565]
Lgals2	-4.14	1.1E-05	lectin, galactose-binding, soluble 2 [Source:MGI Symbol;Acc:MGI:895068]
G6pc	-4.46	2.2E-03	glucose-6-phosphatase, catalytic [Source:MGI Symbol;Acc:MGI:95607]
Cps1	-4.53	6.1E-05	carbamoyl-phosphate synthetase 1 [Source:MGI Symbol;Acc:MGI:891996]
Npc1l1	-5.43	9.0E-05	NPC1-like 1 [Source:MGI Symbol;Acc:MGI:2685089]
Apoa1	-7.22	5.6E-06	apolipoprotein A-I [Source:MGI Symbol;Acc:MGI:88049]

Table S4: List of differentially expressed KEGG pathways between colon tissue of Il10-/- mice colonized with either NC101 or △fyuA for 10 weeks

**KEGG Pathways increased in *fyuA* vs NC**	**Magnitude of perturbation**	**FDR**
mmu04080 Neuroactive ligand-receptor interaction	5.78	4.7E-20
mmu04020 Calcium signaling pathway	3.81	3.8E-09
mmu04713 Circadian entrainment	3.66	2.1E-08
mmu04512 ECM-receptor interaction	3.16	2.0E-06
mmu04724 Glutamatergic synapse	3.10	2.4E-06
mmu04510 Focal adhesion	2.96	5.8E-06
mmu04723 Retrograde endocannabinoid signaling	2.87	1.4E-05
mmu04970 Salivary secretion	2.76	3.4E-05
mmu04911 Insulin secretion	2.44	3.1E-04
mmu04725 Cholinergic synapse	2.42	3.1E-04
mmu04727 GABAergic synapse	2.23	1.2E-03
mmu04610 Complement and coagulation cascades	2.19	1.9E-03
mmu04916 Melanogenesis	1.89	8.3E-03
mmu04151 PI3K-Akt signaling pathway	1.86	8.5E-03
mmu04974 Protein digestion and absorption	1.85	1.0E-02
mmu04514 Cell adhesion molecules (CAMs)	1.82	1.0E-02
mmu04270 Vascular smooth muscle contraction	1.79	1.1E-02
mmu00532 Glycosaminoglycan biosynthesis - chondroitin sulfate / dermatan sulfate	1.84	1.1E-02
mmu04310 Wnt signaling pathway	1.73	1.4E-02
mmu04971 Gastric acid secretion	1.73	1.4E-02
mmu04540 Gap junction	1.67	1.8E-02
mmu04340 Hedgehog signaling pathway	1.66	2.0E-02
mmu04810 Regulation of actin cytoskeleton	1.61	2.2E-02
mmu04726 Serotonergic synapse	1.57	2.8E-02
mmu04728 Dopaminergic synapse	1.55	2.8E-02
mmu04670 Leukocyte transendothelial migration	1.52	3.0E-02
mmu04662 B cell receptor signaling pathway	1.52	3.0E-02
mmu04730 Long-term depression	1.46	4.0E-02
mmu04360 Axon guidance	1.42	4.5E-02

**KEGG Pathways decreased in *fyuA* vs NC**	**Magnitude of perturbation**	**FDR**
mmu00190 Oxidative phosphorylation	-4.99	2.3E-14
mmu03050 Proteasome	-3.79	8.7E-08
mmu03040 Spliceosome	-3.54	8.7E-08
mmu03010 Ribosome	-3.51	1.6E-07
mmu04110 Cell cycle	-3.40	1.6E-07
mmu03013 RNA transport	-3.23	7.1E-07
mmu00100 Steroid biosynthesis	-3.17	1.6E-05
mmu04141 Protein processing in endoplasmic reticulum	-2.83	1.6E-05

mmu04612 Antigen processing and presentation	-2.77	2.9E-05
mmu03030 DNA replication	-2.89	3.6E-05
mmu00240 Pyrimidine metabolism	-2.70	3.6E-05
mmu00900 Terpenoid backbone biosynthesis	-2.62	1.4E-04
mmu03018 RNA degradation	-2.52	1.4E-04
mmu00970 Aminoacyl-tRNA biosynthesis	-2.57	1.4E-04
mmu03460 Fanconi anemia pathway	-2.54	1.4E-04
mmu04120 Ubiquitin mediated proteolysis	-2.31	4.2E-04
mmu04623 Cytosolic DNA-sensing pathway	-2.33	4.3E-04
mmu04622 RIG-I-like receptor signaling pathway	-2.30	4.8E-04
mmu03008 Ribosome biogenesis in eukaryotes	-2.33	5.0E-04
mmu03015 mRNA surveillance pathway	-2.22	7.2E-04
mmu04668 TNF signaling pathway	-2.12	1.2E-03
mmu03420 Nucleotide excision repair	-2.05	2.3E-03
mmu00564 Glycerophospholipid metabolism	-1.99	2.7E-03
mmu04621 NOD-like receptor signaling pathway	-1.91	4.1E-03
mmu03440 Homologous recombination	-1.97	4.1E-03
mmu03022 Basal transcription factors	-1.84	6.9E-03
mmu00030 Pentose phosphate pathway	-1.80	8.5E-03
mmu03430 Mismatch repair	-1.81	9.2E-03
mmu04115 p53 signaling pathway	-1.74	9.2E-03
mmu00020 Citrate cycle (TCA cycle)	-1.77	9.2E-03
mmu00920 Sulfur metabolism	-1.89	9.3E-03
mmu00510 N-Glycan biosynthesis	-1.69	1.2E-02
mmu04146 Peroxisome	-1.65	1.4E-02
mmu03060 Protein export	-1.61	2.0E-02
mmu04660 T cell receptor signaling pathway	-1.52	2.3E-02
mmu00592 alpha-Linolenic acid metabolism	-1.54	2.6E-02
mmu03020 RNA polymerase	-1.48	3.2E-02
mmu00591 Linoleic acid metabolism	-1.46	3.5E-02
mmu00051 Fructose and mannose metabolism	-1.39	4.2E-02
mmu03410 Base excision repair	-1.40	4.2E-02
mmu04975 Fat digestion and absorption	-1.38	4.7E-02
mmu00010 Glycolysis / Gluconeogenesis	-1.35	4.7E-02

Table S5: Iron responsive genes in NC and *fyuA* colonized WT and *Il10*-/- mice

	Log FC at 5 weeks		Log FC at 10 weeks			
	NC v fyuA, Il10	FDR	NC v fyuA, Il10	FDR	NC v fyuA, WT	FDR
Fth1		NS	-0.566	4.92E-02		NS
Ftl		NS		NS		NS
Tfrc		NS	0.712	3.49E-02	1.151	9.98E-04
Alas2		NS		NS		NS
Sdhb		NS		NS		NS
Aco2		NS		NS		NS
Hao1		NS		NS		NS
Slc11a2		NS		NS		NS
Ndufs1		NS		NS		NS
Slc40a1		NS		NS		NS
Cdc42bpa		NS		NS	0.438	3.05E-02
Cdc14a		NS		NS		NS
Epas1	-0.729	3.88E-03		NS		NS
Ndrg1		NS	-0.793	2.25E-04		NS
Hamp		NS		NS		NS

Table S6: Bacterial strains and plasmids used in this study

Strain	Description	Reference
E. coli NC101	Fecal murine isolate with AIEC characteristics	(1)
NC101 Δ*fyuA*	NC101 isogenic mutant with *fyuA* deleted	This study
NC101 Δ*irp1*	NC101 isogenic mutant with *irp1* deleted	This study
NC101 Δ*fyuA irp1*	NC101 Δ*fyuA* isogenic mutant with *irp1* deleted	This study
NC101 Δ*entB*	NC101 isogenic mutant with *entB* deleted	This study
NC101 Δ*fyuA* + *fyuA*	NC101 Δ*fyuA* chromosomally complemented with *fyuA* under the regulation of its native promoter at the *attB* insertion site (2).	This study
**Plasmids**	**Description**	**Reference**
pKD46	Plasmid encoding lambda red recombinase.	(3)
pKD13	Template plasmid for generating linear PCR product for lambda red recombineering.	(4)
pCP20	Plasmid encoding FLP recombinase.	(3)
pTNS2	Plasmid encoding the *tnsABCD* transposition pathway genes.	(5)
pMCL2868-*fyuA*	pMCL2868 harboring the *fyuA* promoter and opening reading frame.	This study
pMSs201	Plasmid containing *gfp* transcriptional fusions with the *E. coli tonB*, *cusC* or *znuA* promoters.	Purchased from Dharmacon

References:

1. **Kim SC**, **Tonkonogy SL**, **Albright CA**, **Tsang J**, **Balish EJ**, **Braun J**, **Huycke MM**, **Sartor RB**. 2005. Variable phenotypes of enterocolitis in interleukin 10-deficient mice monoassociated with two different commensal bacteria. Gastroenterology **128**:891–906.

2. **Ellermann M**, **Huh EY**, **Liu B**, **Carroll IM**, **Tamayo R**, **Sartor RB**. 2015. Adherent-Invasive Escherichia coli Production of Cellulose Influences Iron-Induced Bacterial Aggregation, Phagocytosis, and Induction of Colitis. Infect Immun **83**:4068–4080.

3. **Datsenko KA**, **Wanner BL**. 2000. One-step inactivation of chromosomal genes in Escherichia coli K-12 using PCR products. Proc Natl Acad Sci USA **97**:6640–6645.

4. **Baba T**, **Ara T**, **Hasegawa M**, **Takai Y**, **Okumura Y**, **Baba M**, **Datsenko KA**, **Tomita M**, **Wanner BL**, **Mori H**. 2006. Construction of Escherichia coli K-12 in-frame, single-gene knockout mutants: the Keio collection. Mol Syst Biol **2**:473–11.

5. **Choi K-H**, **Gaynor JB**, **White KG**, **Lopez C**, **Bosio CM**, **Karkhoff-Schweizer RR**, **Schweizer HP**. 2005. A Tn7-based broad-range bacterial cloning and expression system. Nat Meth **2**:443–448.

Table S7: Oligonucleotide primers used in this study

Name	Sequence (5’ to 3’)	Reference
**Generation of bacterial mutants**		
KO_*fyuA* forward	GTATTTTTGGCGTTTCGCCGTCTTACAGGGACTCACAACAGTGTAGG CTGGAGCTGCTTCG	This study
KO_*fyuA* reverse	GTGTAAAAGGGATACCTTTTCGGTATCCCTTTTACAATAACATATGAAT ATCCTCCTTA	
fyuA upstream	GCAAACCGACCCGAAACAGGT	This study
fyuA downstream	GCGACTCTGCTGAGTTACTGGT	
KO_*irp1* forward	GCATGGCGTTCCATTGACTTTATGAACCTTAGGAAATGGGA CCGATTATGATTCCGGGGATCCGTCGACC	This study
KO_*irp1* reverse	TACGCGTTGTTTTGGGGAGGCGGACGGCATCATAACGTG TTCTCCGGTTGTGTAGGCTGGAGCTGCTTCG	
irp1 upstream	GAAAACCAAACGCTCTCGCC	This study
irp1 downstream	GCATTCAGGTACATTTCGCC	
**qRT-PCR of host genes (housekeeping gene, *Gapdh*)**		
Gapdh forward	GGTGAAGGTCGGAGTCAACGGA	(4)
Gapdh reverse	GAGGGATCTCGCTCCTGGAAGA	
Il12b forward	CGCAAGAAAGAAAAGATGAAGGAG	(1)
Il12b reverse	TTGCATTGGACTTCGGTAGATG	
Ifng forward	CTTCCTCATGGCTGTTTCTGG	(2)
Ifng reverse	ACGCTTATGTTGTTGCTGATGG	
Col1a2 forward	AGGCCCAACCTGTAAACACC	This study
Col1a2 reverse	CTGAGAAGCACGGTTGGCTA	
Fn1 forward	CCAACTCCTTGCTGGTGTCA	This study
Fn1 reverse	GAGAGCTTCCTGTCCTGTCT	
Tgfb1 forward	TCTCTGTGGAGCTGAAGCAA	(5)
Tgfb1 reverse	TGAGTGGCTGTCTTTTGACG	
Tgfbr2 forward	AGCATCACGGCCATCTGTG	This study
Tgfbr2 reverse	TGGCAAACCGTCTCCAGAGT	

**qRT-PCR of host genes (housekeeping gene, *16S rRNA gene*)**		
E. coli 16S forward	GTTAATACCTTTGCTCATTGA	(3)
E. coli 16S reverse	ACCAGGGTATCTAATCCTGTT	
fyuA forward	CAAAAGCGCTCAGGGCGGGA	This study
fyuA reverse	CGCCGTCATCAACCTGGCGT	
Irp1 forward	TGCTGAAGGACTGAAGCCAG	This study
Irp1 reverse	ACCGTATGTTGCGTACCGTT	
ybtS forward	GGAACAATGGCTACCGACGA	This study
ybtS reverse	AATGGAATCCACCGCCCATT	
ybtA forward	AACCCACTTAACGGCTCAGG	This study
ybtA reverse	AGCACCAGACTGAGCATGAC	
ybtE forward	GCAAGGGTTTTACCGCACAG	This study
ybtE reverse	ATAAACGCGCAAATCCGCTC	
tonB forward	TTTGATGTCACGCCAGATGGT	This study
tonB reverse	GGCTTACCCGGCTCATAACG	
entB forward	CGACTACGGTCTGGATTCGG	This study
entB reverse	GAGAGTAGCTTCCACCAGGC	

References:

1. **Ellermann M**, **Huh EY**, **Liu B**, **Carroll IM**, **Tamayo R**, **Sartor RB**. 2015. Adherent-Invasive Escherichia coli Production of Cellulose Influences Iron-Induced Bacterial Aggregation, Phagocytosis, and Induction of Colitis. Infect Immun **83**:4068–4080.

2. **Arthur JC**, **Gharaibeh RZ**, **Mühlbauer M**, **Perez-Chanona E**, **Uronis JM**, **McCafferty J**, **Fodor AA**, **Jobin C**. 2014. Microbial genomic analysis reveals the essential role of inflammation in bacteria-induced colorectal cancer. Nature Communications **5**:4724.

3. **Maharshak N**, **Packey CD**, **Ellermann M**, **Manick S**, **Siddle JP**, **Huh EY**, **Plevy S**, **Sartor RB**, **Carroll IM**. 2013. Altered enteric microbiota ecology in interleukin 10-deficient mice during development and progression of intestinal inflammation. Gut Microbes **4**:316–324.

4. **Sun X**, **Threadgill D**, **Jobin C**. 2012. Campylobacter jejuni induces colitis through activation of mammalian target of rapamycin signaling. Gastroenterology **142**:86-95.

5. **Kashiwagi I**, **Morita R**, **Schichita T, Komai K**, **Saeki K**, **Matsumoto M**, **Takeda K, Nomura M, Hayashi A, Kanai T**, **Yoshimura A**. 2015. Smad2 and Smad3 inversely regulate TGF-beta autoinduction in Clostridium butyricum-activated dendritic cells. Immunity **43**:65-79.
